# Tandem catalysis enables chlorine-containing waste as chlorination reagents

**DOI:** 10.1038/s41557-024-01462-8

**Published:** 2024-02-23

**Authors:** Mingyang Liu, Xinbang Wu, Paul J. Dyson

**Affiliations:** Institute of Chemical Sciences and Engineering, Ecole Polytechnique Fédérale de Lausanne (EPFL), Lausanne, Switzerland

**Keywords:** Sustainability, Organic chemistry

## Abstract

Chlorinated compounds are ubiquitous. However, accumulation of chlorine-containing waste has a negative impact on human health and the environment due to the inapplicability of common disposal methods, such as landfill and incineration. Here we report a sustainable approach to valorize chlorine-containing hydrocarbon waste, including solids (chlorinated polymers) and liquids (chlorinated solvents), based on copper and palladium catalysts with a NaNO_3_ promoter. In the process, waste is oxidized to release the chlorine in the presence of N-directing arenes to afford valuable aryl chlorides, such as the FDA-approved drug vismodegib. The remaining hydrocarbon component is mineralized to afford CO, CO_2_ and H_2_O. Moreover, the CO and CO_2_ generated could be further utilized directly. Thus, chlorine-containing hydrocarbon waste, including mixed waste, can serve as chlorination reagents that neither generate hazardous by-products nor involve specialty chlorination reagents. This tandem catalytic approach represents a promising method for the viable management of a wide and diverse range of chlorine-containing hydrocarbon wastes.

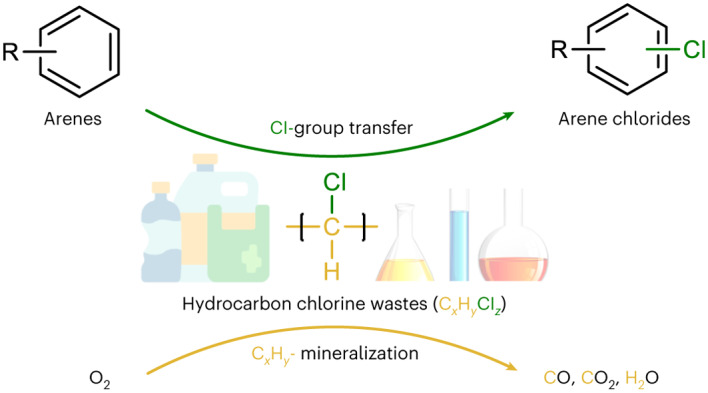

## Abstract (web summary)

While chlorinated compounds are ubiquitous in chemical synthesis, they have a negative impact on human health and the environment. Now, a sustainable tandem catalytic process has been developed that uses chlorine-containing waste as chlorination reagents. This approach represents a promising way for the viable management of chlorinated compounds.

## Main

Chlorinated compounds are ubiquitous in industrialized societies, as the physical and chemical properties of hydrocarbons are extensively modified (and improved) following chlorination. Thus, chlorine-containing hydrocarbons are widely used as plastics^1,2^, rubbers^3–5^, resins^6,7^ and solvents^8–10^, and have applications as intermediates used in the synthesis of agrochemicals and pharmaceuticals^11,12^, or are even present in agrochemical and pharmaceutical products^13–16^. Despite their enormous utility, chlorine-containing hydrocarbons are persistent in the environment or decompose to release toxic compounds, which can accumulate in the food chain. Exposure to chlorinated waste is even associated with suppression of the immune system and cancer, and yet methods to dispose of waste chlorine-containing hydrocarbons are currently inadequate^17–19^.

**Fig. 1:**
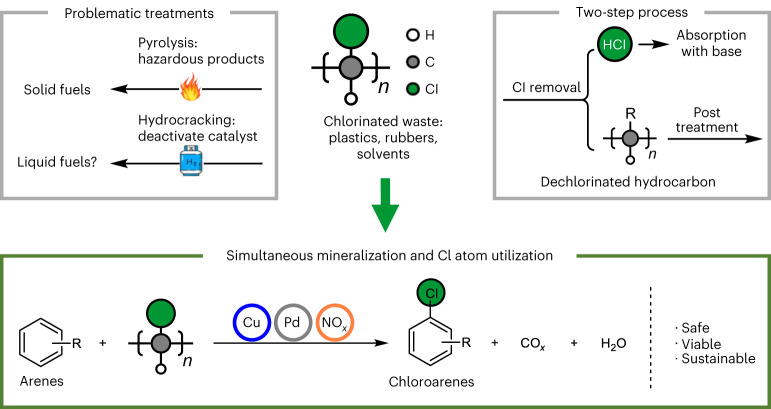
Methods development. Methods developed for the possible elimination of chlorinated wastes and the approach described in this work in which various chlorinated wastes are employed as chlorination reagents for the synthesis of fine chemicals, with concomitant mineralization of the hydrocarbon component.

Chlorine-containing hydrocarbon waste is one of the most problematic types of waste because of the large scale of such waste and the common industrial approaches used to deal with it (landfill and incineration), which lead to the formation of hazardous products, including dioxins and corrosive gases such as HCl and Cl_2_. Safe disposal of chlorinated waste is therefore highly challenging (Fig. 1), with both pyrolysis and hydrocracking being unsuitable, as they result in the same problems as industrial incineration^20–24^. Although catalytic hydrocracking has been extensively investigated for upgrading a range of polymers, when applied to chlorinated polymers, deactivation of the catalyst is usually observed^25–28^. Hence, a two-step process consisting of dechlorination followed by decomposition has been reported, but this is expensive and largely unviable^20,29–33^. Recent approaches used for the destruction of polyvinyl chloride (PVC) are summarized in Supplementary Table 1 (note that HCl is produced in the majority of cases and is typically neutralized in a second step). Recently an electrochemical dechlorination method was reported for PVC, but only partial dechlorination (<20% conversion of Cl groups) was achieved and the method might be difficult to upscale^34^. Likewise, waste chlorinated solvents are problematic, with incineration being the conventional method used to dispose of them, as bioremediation is slow^35,36^. Electrochemical dechlorination of chlorinated hydrocarbon compounds has been reported, but suffers from low reaction rates and low Faradaic efficiencies (typically <50%)^37,38^. On account of these limitations for the reported treatments (which require corrosive reagents or lead to corrosive HCl emissions), there does not appear to be a sustainable, scalable method that efficiently eliminates different types of chlorinated hydrocarbon waste.

Extending on the achievements in Pd-catalysed C–H bond chlorination^39–42^ and Cu/NO_*x*_-catalysed C–C bond oxygenation^43–46^, we integrated the two reactions to afford a safe, sustainable and economically viable strategy to use chlorinated waste as chlorination reagents (Fig. 1). The approach simultaneously generates CO/CO_2_, via Cu/NO_*x*_-catalysed oxygenation of the hydrocarbon component, and chloride, which is transferred via Pd-catalysed C–H bond chlorination, to afford valuable aryl chlorides.

## Results and discussion

### Initial investigation of the reaction conditions

**Fig. 2:**
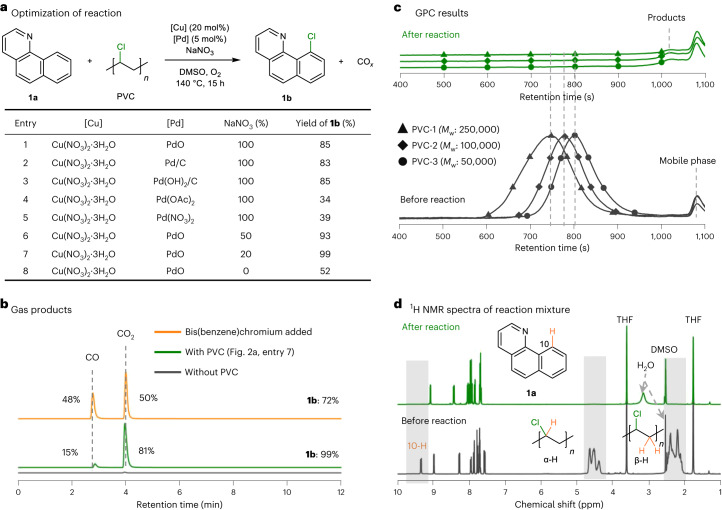
Reaction optimization and characterization of the products. **a**, Optimization of the reaction parameters. Reaction conditions: 7,8-benzoquinoline **1a** (0.25 mmol), PVC (1.5 equiv., 0.375 mmol), Cu catalyst (20 mol%, 0.05 mmol), Pd catalyst (5 mol%, 0.0125 mmol), NaNO_3_ (0–100 mol%), biphenyl (0.2 mmol, internal standard), dimethylsulfoxide (DMSO; 2 ml), O_2_ (3 bar), 140 °C, 15 h. Yield of gas and liquid products was determined by GC. **b**, GC of gas-phase products after reaction (reaction conditions correspond to those in **a**, entry 7) and the control experiment without PVC. **c**, GPC chromatograms before and after reaction. **d**, ^1^H NMR spectra of reaction mixture before and after reaction (reaction conditions correspond to those in **a**, entry 7). THF-d_8_ was used instead of DMSO-d_6_ to dissolve the reaction mixture before reaction, as PVC is insoluble in DMSO. THF-d_8_ was also added into the reaction mixture after reaction, for the ^1^H NMR spectroscopic analysis.

The high polarity of C–Cl bonds compared to C–H bonds in chlorine-containing hydrocarbon waste facilitates interactions with catalysts and can lead to their activation and functionalization^47,48^. Following C–Cl bond activation, transfer of the chlorine to a substrate, and mineralization of the hydrocarbon component of the waste, must be initiated. Commencing with conditions similar to those reported for the transfer of chlorine atoms in chlorophenol pollutants to arenes^49^, a Cu-catalysed aerobic reaction employing 7,8-benzoquinoline **1a** as substrate and PVC (molecular weight, *M*_w_: 100,000) as chlorination reagent, led to only trace quantities of the anticipated chlorinated product, namely, 10-chlorobenzoquinoline **1b** (Supplementary Fig. 1). Since Pd catalysts are widely used to form C–X bonds^42^, bimetallic systems consisting of Cu and Pd catalysts were evaluated and found to generate the chlorinated product **1b** (Fig. 2a, entries 1–5). The combination of homogeneous Cu(NO_3_)_2_ and heterogenous Pd catalysts (PdO, Pd/C, Pd(OH)_2_/C) remarkably enhances the yield of **1b** to 83–85% (Fig. 2a, entries 1–3). Notably, the homogeneous Pd complexes are less efficient catalysts than heterogenous Pd catalysts (Fig. 2a, entries 4 and 5), which may be attributed to the strong coordination of the N-directing substrate leading to stable complexes that are less active^50,51^. Optimization of the NaNO_3_ concentration afforded the chlorinated product **1b** in 99% yield (Fig. 2a, entry 7). The heterogenous PdO may be recovered by filtration and reused without loss in activity (Supplementary Fig. 11). Further details concerning the optimization of the reaction parameters are provided in Supplementary Figs. 1–10.

The gaseous products from the reaction were analysed by gas chromatography (GC) with CO and CO_2_ obtained in 15 and 81% yield, respectively, based on the carbon content of PVC (Fig. 2b), confirming that the hydrocarbon component of PVC is mineralized in near-quantitative yield (the formation of water was detected by ^1^H NMR spectroscopy; see below). Note that these gases were not observed in a control experiment without PVC. Notably, in the absence of PdO catalyst (Supplementary Table 2, entry 20), CO is obtained in 83% yield (with **1b** formed in 12% yield), suggesting that PdO promotes the oxidation of CO to CO_2_ (ref. ^52^). Consequently, it was found that in the presence of PdO, that is, under the standard reaction conditions, the selectivity to CO can be improved to 48% by adding bis(benzene)chromium (Fig. 2b and Supplementary Table 2), with the chlorinated product **1b** formed in 72% yield. The product distribution (using PVC and **1a** as substrates) was quantified, and confirms that the PVC was completely mineralized into gases (CO and CO_2_) and the Cl atoms converted into **1b** and excess HCl (Supplementary Fig. 12). Moreover, the unpurified gas stream was directly used in the carbonylation of 1-phenylethane-2,3-diol^53^ (consuming the CO generated) and in the cycloaddition of CO_2_ to styrene oxide (fixing the CO_2_ produced) affording styrene carbonate, thereby valorizing all the carbon atoms in PVC (Supplementary Fig. 13).

The depolymerization of PVC was monitored by gel permeation chromatography (GPC) and chromatograms before and after reaction (Fig. 2c) show that the characteristic peaks of PVC (with different molecular weights) completely disappear. Only signals corresponding to small molecules, that is, **1a** and **1b**, remain, confirming the total depolymerization of the polymers. ^1^H NMR spectroscopy shows that after the reaction (Fig. 2d; see caption for details of the reaction conditions), the characteristic peaks attributed to the 10-H of substrate **1a** and the α/β-Hs of PVC are no longer present, and only signals attributable to the chlorinated product **1b** and H_2_O are observed (for comparison, the ^1^H NMR spectrum of **1b** is provided in Supplementary Fig. 14). The region between 1 and 5 ppm is clear, which is where signals derived from alkyl monomers or oligomers would be expected to be observed. Thus, **1b** and water are the only liquid products obtained during the reaction.

### Scope of the chlorinated waste

Solid waste derived from commercial chlorinated plastics and rubbers is typically manufactured with other components present, including plasticizers, fillers and pigments, to provide rigidity, elasticity, colour and other properties^34^. To explore the utility of our method when applied to real waste, a range of postconsumer products were evaluated (Table 1). Using a PVC-based water pipe, electrical conduit and electric wire (**P1**; Supplementary Fig. 15), chlorinated product **1b** was obtained in yields of 80 to 99%. Transparent polyvinylidene chloride (PVDC, **P2**) is extensively used as a packaging material for food and pharmaceutical products, and in the forms of raw powder and blister affords **1b** in 99% yield. Polyepichlorohydrin (PECH, **P3**) with primary Cl groups was also tested and gave **1b** in 99% yield. Rubber has short polymeric chains and extensive cross-linkages, endowing it with greater elasticity than most plastics, but making it more challenging to depolymerize^54,55^. Nonetheless, neoprene rubber (**P4**), raw chunks and vacuum tube, both afford **1b** in a similar yield, that is, 80–82%, showing that the cross-linkages are transformed under the reaction conditions. Substrate **1a** was also chlorinated in the presence of copolymers with hetero-oxygen atoms, that is, poly(epichlorohydrin-co-ethylene oxide) (PECHEO, **P5**) and poly(epichlorohydrin-co-CO_2_) (PECHC, **P6**), with **1b** obtained in 99% yield. We simulated real waste streams by employing a mixture of several types of PVC-based and polyolefin (polyethylene (PE) and polypropylene (PP)) plastic waste in the chlorination of **1a** under the standard reaction conditions. The polyolefins remain intact after reaction, whereas the PVC was consumed to afford chlorinated product **1b** in 87% yield. The use of mixed plastics demonstrates the possibility to selectively valorize PVC from mixtures containing polyolefins and potentially other types of plastic waste, although it is likely that real waste streams would require washing before use, as is common in other plastic upcycling processes^56,57^.

**Table 1:**
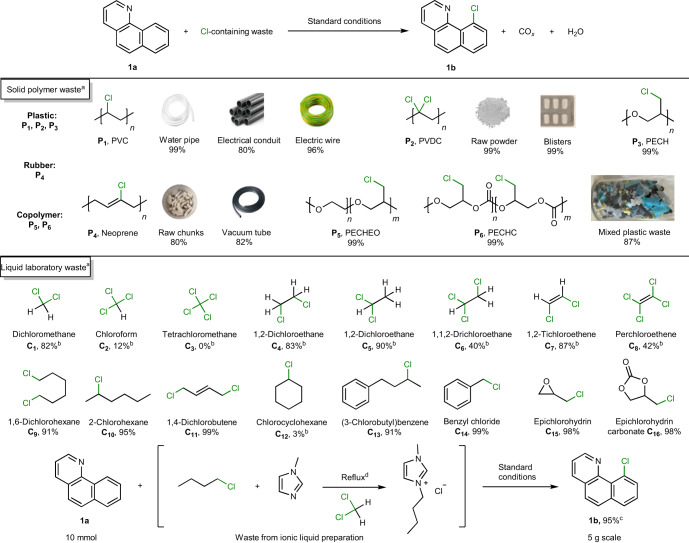
Application of chlorinated polymers, including postconsumer waste and liquid waste, in the chlorination reaction of **1a** to **1b**

Reaction conditions: ^a^7,8-benzoquinoline **1a** (0.25 mmol), chlorinated waste (1.5 equiv., 0.375 mmol, calculated on the basis of the content of chlorine—the chlorine content of postconsumer waste was determined by Schoniger combustion), Cu(NO_3_)_2_**·**3H_2_O (20 mol%, 0.05 mmol), PdO (5 mol%, 0.0125 mmol), NaNO_3_ (20 mol%, 0.05 mmol), biphenyl (0.2 mmol, internal standard), DMSO (2 ml), O_2_ (3 bar), 140 °C, 15 h. ^b^Chlorinated waste (3 equiv., 0.75 mmol). ^c^7,8-Benzoquinoline **1a** (30 mmol), 5.4 gram scale, compressed air (8 bar), 20 h. ^d^1-Methylimidazole (20 mmol), 1-chlorobutane (30 mmol) and dichloromethane (20 ml), reflux at 60 °C, 48 h. The mixed waste contained by mass: 25% PVC water pipe, 25% PVC electrical conduit, 25% PVC electric wire and 25% polyolefin plastic waste (PE and PP). Yield of the chlorinated product **1b** was determined by GC.

In addition to plastic waste, various liquid chlorine-containing hydrocarbon wastes were evaluated as chlorination reagents (Table 1). Chlorinated solvents and reagents are extensively used in chemistry and for cleaning purposes in many industries^8,9,58^. For example, dichloromethane (DCM) is the most extensively used solvent by volume in the pharmaceutical industry (~18%), and requires safe disposal to minimize damage to human health (it is a carcinogen) and damage to the environment. DCM and other commonly used chlorinated solvents (**C1** and **C4**–**C8**) are good chlorination reagents in our system (40–87%), with the exception of chloroform (**C2**) and tetrachloromethane (**C3**). Note that perchloroethene (**C8**) is the most widely used solvent in dry cleaning^58^. Other mono- and dichlorinated alkanes (**C9** and **10**), alkenes (**C11**) and alkylbenzenes (**C13** and **14**) serve as excellent sources of chlorine, to afford chlorinated product **1b** in 91–99% yield. Chlorocyclohexane (**C12**) is a poor chlorination reagent, whereas epichlorohydrin (**C15**) and epichlorohydrin carbonate (**C16**) perform chlorination well, with both affording **1b** in 98% yield.

Actual laboratory waste was used in the reaction, obtained from the synthesis of the ionic liquid 1-butyl-3-methylimidazolium chloride ([BMIM]Cl) prepared from the reaction of excess butyl chloride in DCM (Table 1). Following isolation of the [BMIM]Cl product, the chlorinated waste includes residual butyl chloride and DCM. Using this mixture as the chlorination reagent and performing the reaction on a 5 g scale with respect to **1a**, the chlorinated product **1b** was obtained in 95% (GC) yield. The pressure and temperature of the reaction were monitored online for a reaction employing 5.4 g of **1a**, implying that this is safe to perform when using the appropriate equipment (Supplementary Fig. 16). To further demonstrate the functional group compatibility of this reaction, an additive challenge was performed (Supplementary Table 3)^59^. We found that only amine additives hinder the activity of our method.

### Substrate scope of arenes

Chlorinated arenes are widely employed as pharmaceuticals, agrochemicals, dyes and solvents^13,42,60^. Chlorination of arenes typically involves toxic and corrosive reagents (sulfuryl dichloride, HCl, Cl_2_)^61,62^ or the application of expensive reagents, such as chloramines, which have a low atom economy^42,63,64^. In contrast, using chlorinated waste as chlorination reagents may be considered as an inexpensive, benign and sustainable method that alleviates problems related to the disposal of such waste. To investigate the scope of this method, a range of aromatic and heteroaromatic substrates with N-directing groups were evaluated using colourless PVC water pipe as the chlorination reagent (Table 2). Pyridine-directing arenes with various electron-withdrawing and electron-donating groups were chlorinated with high selectivity to afford regioselective aryl *ortho*-dichloride products (**2b**–**7b**) or aryl *ortho*-monochloride products (**8b**, **9b**) in 74–98% yield. The impact of functional groups on the pyridine-directing group (**10a**, **11a**) is critical to control the selectivity of *ortho*-di- or monochloride products (**10b**, **11b**). Product **12b** is an intermediate in the synthesis of vismodegib, a drug used for the treatment of basal cell carcinoma (see ‘Application to pharmaceutical synthesis’ below)^65,66^. Pyrimidine-directing arenes (**13a**–**18a**) also afford the expected chlorinated products, but in slightly lower selectivity of *ortho*-di- or monochloride products. Interestingly, arenes with pyrazol-directing groups (**19a**–**24a**) were selectively monochlorinated in good yield (72–81%). Arenes with 8-aminoquinoline (bidentate, NHQ)-directing groups (**25a**–**30a**) were also mono- or dichlorinated, but in slightly lower yields (58–80%). Compounds **25b**–**27b** are important synthetic intermediates of active EP_1_ antagonists^67^ and positive allosteric modulators^68^. In general, the excess PVC employed in the reaction is to ensure high product yields and does not affect the selectivity for *ortho*-di- or monochloride products. Employing PVC loadings containing stochiometric amounts of chlorine in the reaction slightly decrease the product yield. For example, when employing **12a** as substrate (Supplementary Fig. 17), decreasing the PVC loading from 1.5 to 0.75 equiv. decreases the yield of monochloride product from 92% (Cl atom economy 71%) to 65% (Cl atom economy 97%).

**Table 2:**
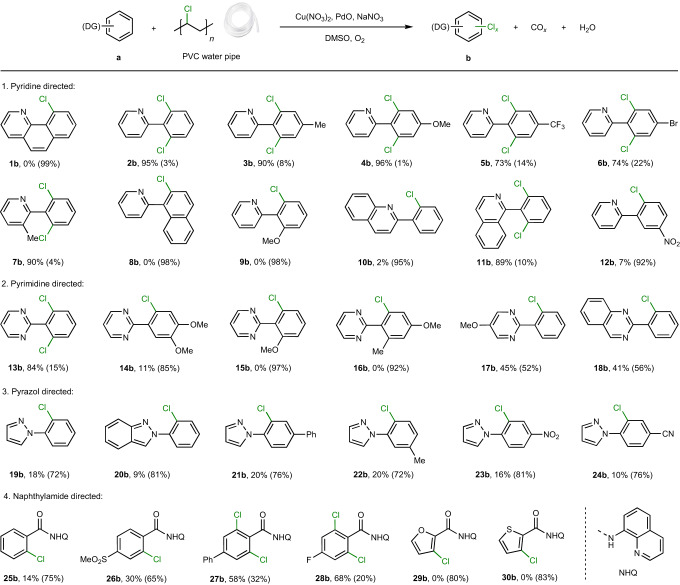
Evaluation of the substrate scope

Reaction conditions: arene **a** (0.25 mmol), PVC water pipe (1.5 equiv., 0.375 mmol), Cu(NO_3_)_2_**·**3H_2_O (20 mol%, 0.05 mmol), PdO (5 mol%, 0.0125 mmol), NaNO_3_ (20 mol%, 0.05 mmol), biphenyl (0.2 mmol), DMSO (2 ml), O_2_ (3 bar), 140 °C, 15 h. Yields of *ortho*-dichloride products are reported and yields of monochloride products are included in the brackets.

### Application to pharmaceutical synthesis

**Fig. 3:**
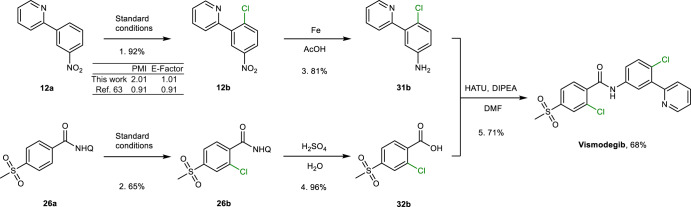
Synthesis of vismodegib. Reaction conditions: steps 1 and 2 were performed using the standard conditions, that is, **12a** or **26a** (0.25 mmol), PVC water pipe (1.5 equiv., 0.375 mmol), Cu(NO_3_)_2_**·**3H_2_O (20 mol%, 0.05 mmol), PdO (5 mol%, 0.0125 mmol), NaNO_3_ (20 mol%, 0.05 mmol), DMSO (2 ml), O_2_ (3 bar), 140 °C, 15 h; step 3, **12b** (0.50 mmol), Fe powder (5.0 mmol) and AcOH (4 ml), 80 °C, 0.5 h; step 4, **26b** (0.45 mmol) and 40% H_2_SO_4_ aqueous solution (2 ml), 120 °C, 12 h; step 5, **31b** (0.20 mmol), **32b** (0.20 mmol), 1-[bis(dimethylamino)methylene]-1*H*-1,2,3-triazolo[4,5-*b*]pyridinium 3-oxide hexafluorophosphate (HATU; 0.12 mmol), *N*,*N*-diisopropylethylamine (DIPEA; 0.21 mmol) and anhydrous DMF (2 ml), room temperature, 24 h. GC yields of each step and overall yield of vismodegib are based on **12a**. Green metrics including PMI and E-factor of step 1 are included. See Supplementary Information for details of the calculations.

This method using Cl-containing waste as a chlorination reagent was applied to the synthesis of the FDA-approved anticancer drug vismodegib (Fig. 3). On the basis of a retrosynthetic analysis of vismodegib, the pyridine-directed arene **12a** and NHQ-directed arene **26a** were selected as the starting materials^66^. *Ortho*-C–H chlorination of **12a** followed by reduction gives 4-chloro-3-pyridin-2-ylaniline, **31b**. Compound **26a** was chlorinated using the standard conditions (see caption to Fig. 3) and hydrolysed to afford 2-chloro-4-(methylsulfonyl)benzoic acid, **32b**. Condensation of **31b** and **32b** affords vismodegib, with an overall yield of 68%. The two Cl groups in vismodegib are derived from the waste PVC water pipe, and the spectroscopic and analytical data (Supplementary Figs. 18–20 and Supplementary Table 4) indicate that the route should be compatible with the stringent purity requirements of the pharmaceutical industry. Green metrics calculations of the process mass intensity (PMI) and environmental factor (E-factor) were performed (Fig. 3), demonstrating that the chlorination method has a similar efficiency and sustainability to a reported C–H chlorination method^63^.

### Mechanistic studies

**Extended Data Fig. 1:**
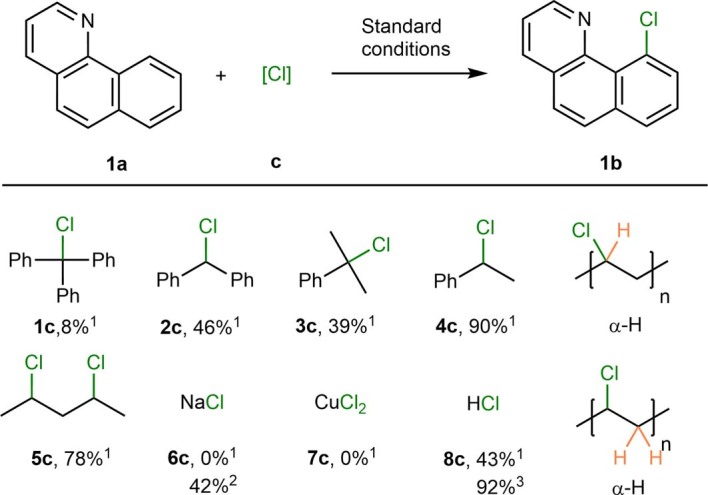
Application of model organic and inorganic chlorides as chlorination reagents. Standard conditions: (1) 7,8-benzoquinoline **1a** (0.25 mmol), chlorides (1.5 equiv., 0.375 mmol), Cu(NO_3_)_2_•3H_2_O (20 mol%, 0.05 mmol), PdO (5 mol%, 0.0125 mmol), NaNO_3_ (20 mol%, 0.05 mmol), biphenyl (0.2 mmol, internal standard), DMSO (2 mL), O_2_ (3 bar), 140 °C, 15 h. (2) with acetic acid (3 equiv., 0.75 mmol). (3) with HCl (3 equiv., 0.75 mmol).

The reaction mechanism of this reliable and robust chlorination method was probed with a view to establishing the role of the Cu(NO_3_)_2_ and PdO catalysts, the NaNO_3_ promoter and the strong solvent effect of dimethylsulfoxide (DMSO). The reaction may be divided into three parts: (1) dechlorination of chlorinated waste; (2) depolymerization/decomposition of the dechlorinated waste; and (3) chlorination of the arene substrate. Initially, several potential, model chlorination reagents were tested under the standard reaction conditions (Extended Data Fig. 1). Reagents without an α-H and β-H (**1c**), or chloride salts without H^+^ (**6c**, **7c**), were inactive or sparingly active (0–8%). Reagents with either an α-H (**2c**) or β-H (**3c**) only afforded the chlorinated product **1b** in modest yield (46% and 39%, respectively). In contrast, reagents containing both α- and β-H (**4c**, **5c**) provide **1b** in good yield (78–90%), which demonstrates that removable α- and β-H are required for chlorination. The application of acetic acid (provides excess H) and NaCl (**6c**, condition 2) or alternatively HCl (**8c**) as chlorination reagents notably improves the yield of **1b**, further confirming the importance of H (cleavable C–H bonds or H^+^). These control experiments suggest that in the initial dechlorination step HCl is generated, which acts as the chlorination reagent, and the pH of the reaction mixture confirms the formation of HCl (Supplementary Fig. 21).

**Extended Data Fig. 2:**
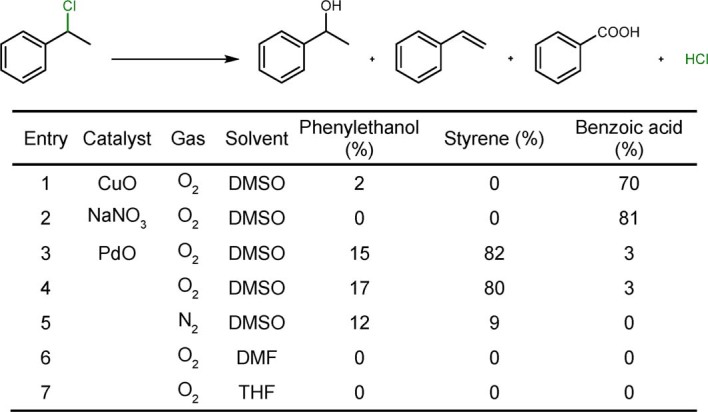
Evaluation of dechlorination and C=C/C(OH)−C bond activation using model compound **4c**. Reaction conditions: substrate **4c** (0.25 mmol), catalyst (20 mol%, 0.05 mmol), solvent (2 mL), biphenyl (0.2 mmol), gas (1 bar), 140 °C, 3 h.

Next, the dechlorination of the model reagent 1-phenylethyl chloride (**4c**) was studied (Extended Data Fig. 2). Using CuO (Extended Data Fig. 2, entry 1) or NaNO_3_ (Extended Data Fig. 2, entry 2) as the catalyst in DMSO, benzoic acid is the dominant product, revealing simultaneous dechlorination and carbon–carbon bond cleavage. With PdO (Extended Data Fig. 2, entry 3) or without catalyst (Extended Data Fig. 2, entry 4), styrene (obtained by dehydrochlorination) is the main product with 1-phenylethanol (obtained by dechlorohydroxylation) obtained in lower quantities. Dechlorination is not observed in the absence of O_2_ (Extended Data Fig. 2, entry 5) or in other polar solvents (DMF or tetrahydrofuran (THF); Extended Data Fig. 2, entries 6 and 7). ^1^H NMR spectroscopy confirms that **4c** (used as a model chlorination reagent) interacts with the DMSO solvent, activating the C–Cl bond (Supplementary Fig. 22)^69^. Hydroxyl radicals were detected by electron paramagnetic resonance spectroscopy during the DMSO/O_2_-promoted dehydrochlorination process (Supplementary Fig. 23), indicating that dehydrochlorination involves a radical-mediated pathway. Radical trapping and radical clock experiments demonstrate the formation of carbon-centred radicals through the release of Cl^•^ radicals (Supplementary Figs. 24 and 25). These results imply that DMSO/O_2_ catalyses the dehydrochlorination step to afford HCl^16,70,71^, with the formation of alkene (dominant) and alkanol (minor) intermediates (the hydroxyl oxygen may originate from O_2_; Supplementary Fig. 26). The NaNO_3_ promotes the oxidative decomposition of the dechlorinated (hydrocarbon component) polymer/solvent via C=C/C(OH)–C bond activation of the alkene and alkanol intermediates (Supplementary Figs. 27–29)^43–45,49^. In the final step, the PdO catalyses the chlorination of the arene substrate using HCl as the actual chlorination reagent^39,42,72^.

**Extended Data Fig. 3:**
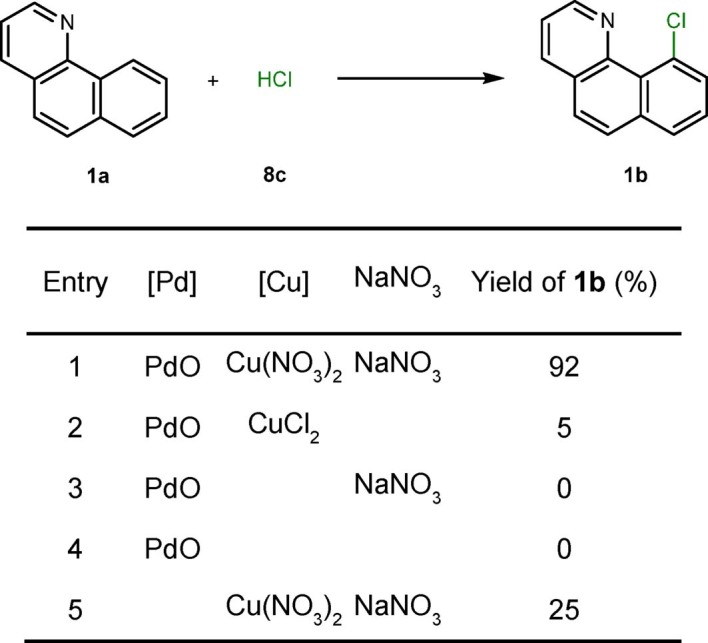
Optimization of the chlorination reaction using HCl as a chlorination reagent. Standard conditions: 7,8-benzoquinoline **1a** (0.25 mmol), HCl (3 equiv., 0.75 mmol), Cu(NO_3_)_2_•3H_2_O (20 mol%, 0.05 mmol), PdO (5 mol%, 0.0125 mmol), NaNO_3_ (20 mol%, 0.05 mmol), biphenyl (0.2 mmol, internal standard), DMSO (2 mL), O_2_ (3 bar), 140 °C, 15 h.

Pd-catalysed C–H activation/chlorination was also investigated (Extended Data Fig. 3). Using HCl as the chlorination reagent, chlorination of **1a** does not proceed well without additional Cu catalyst and NaNO_3_ (Extended Data Fig. 3, entries 1–4), which implies that the Cu catalyst and NO_3_^−^ influence the activity of the Pd catalyst during the oxidative C–H chlorination reaction^40,73–75^. The model benzo[*h*]quinolinyl Pd(II) chloride intermediate was evaluated under the standard reaction conditions and affords chlorinated product **1b** only in the presence of both Cu and NO_3_^−^ catalysts (Supplementary Fig. 30). X-ray photoelectron spectroscopy was used to confirm that the Cu and NO_3_^−^ catalysts assist in the oxidation of the Pd catalyst in the presence of O_2_ (Supplementary Fig. 31). These experiments imply that the Cu and NO_3_^−^ catalysts act as redox mediators between O_2_ and Pd(II) intermediates^73,75^, promoting the formation of Pd(IV) intermediates, followed by reductive elimination to afford chlorinated products^39–41^. A series of tests to establish the nature of the active PdO catalyst was conducted, including hot filtration, elemental analysis, mercury poisoning, Pd(II) trapping and mass spectrometry, with all indicating that the active PdO catalyst is heterogeneous (Supplementary Figs. 32 and 33), although it cannot be excluded that, to a minor extent, the heterogeneous catalyst acts as a reservoir for homogeneous Pd species stabilized by the DMSO solvent^76^. Notably, without PdO, the combination of Cu(NO_3_)_2_ and NaNO_3_ affords chlorinated product **1b** in 25% yield, presumably due to a less favourable Cu-catalysed chlorination pathway involving reduction elimination from a Cu(III) intermediate (Supplementary Fig. 34)^77,78^.

**Fig. 4:**
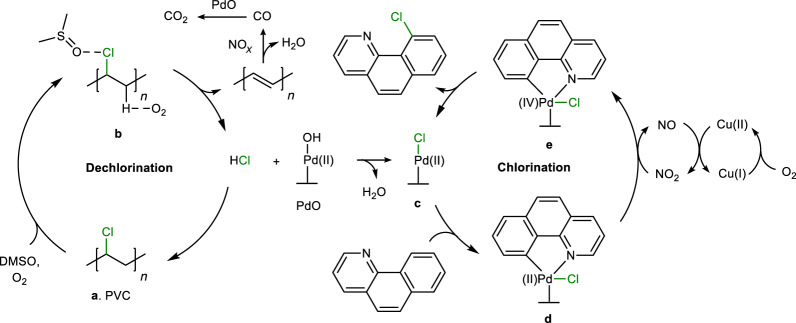
Mechanism study. Proposed reaction pathway based on PVC as the chlorination reagent.

Taken together, a tentative reaction sequence is shown in Fig. 4. Dechlorination catalysed by DMSO/O_2_ (**a** to **b**) affords alkene and HCl intermediates (Supplementary Fig. 26). The hydrocarbon component undergoes oxidization to CO and H_2_O via NaNO_3_-promoted C–C bond cleavage (Supplementary Fig. 29), followed by oxidation of CO to CO_2_ products (Pd-catalysed mainly). The HCl generated serves as the active chlorination reagent, which generates chlorinated arenes catalysed by the Pd species (**c** to **e**; Supplementary Fig. 35). Tentative reaction pathways for PVC- and PVDC-based chlorination reagents are provided in Supplementary Figs. 26, 29 and 34–36.

### Life cycle assessment

To evaluate the environmental impact of the chlorination process, life cycle assessment (LCA) modelling was performed and compared with other chlorination strategies, with each system optimized to afford 1 kg of product. Note that the equivalent of PVC is considered to be incinerated for scenarios that do not employ PVC as the chlorination reagent and the production of valuable CO is not included. Details of LCA calculations are provided in Supplementary Figs. 37–40 and Supplementary Tables 5–7. LCA of the common chlorination route^39,41^ using *N*-chlorosuccinimide as the chlorination reagent (scenario 1) for the generation of 2-(2-chloro-5-nitrophenyl)pyridine **12b**, an intermediate in the synthesis of the anticancer drug vismodegib, produces 9.18 kg of CO_2_, whereas using PVC as the chlorination reagent (scenario 2), less than six times the amount of CO_2_ is generated, that is, 1.41 kg, despite the latter route producing CO_2_ gas as a by-product. The main contribution of the CO_2_ generated from the *N*-chlorosuccinimide route consists of the production of the chlorination reagent itself and unrecoverable Pd(OAc)_2_ catalyst required, whereas using PVC waste is free of charge and the heterogeneous PdO catalyst may be recovered (Supplementary Fig. 11). The PVC route was also compared with the recently published electrochemical chlorination strategy using PVC as a chlorination reagent for the generation of 1 kg of 1-(2-chlorophenyl)-pyrazole **19b** (compare scenarios 3 and 4)^34^. In the electrochemical process, a large excess of PVC and tetrabutylammonium tetrafluoroborate (NBu_4_BF_4_) containing electrolyte are needed (the expensive bis(2-ethylhexyl) phthalate additive was not included in the analysis). The LCA indicates that electrochemical chlorination will produce 54.62 kg of CO_2_, which is over 20 times higher than the method reported here, which would produce 2.59 kg of CO_2_. The main source of CO_2_ is from the large quantity of NBu_4_BF_4_ electrolyte required. The LCA confirms that the method is more promising and less environmentally detrimental than other reported methods.

## Conclusions

More sustainable alternatives to chlorinated compounds are a key goal in green chemistry, although in some cases, for example, in pharmaceutical products^79^, it is unlikely that replacements will be identified in the near future. The choice of investing in greener chlorination approaches, or finding non-chlorinated alternatives, should be guided by a thorough assessment of the impact on both public health and the environment. Here we have shown that intractable chlorinated waste can be used as chlorination reagents to produce valuable aryl chloride products, with the hydrocarbon component being simultaneously mineralized. A new synthetic route to the FDA-approved drug, vismodegib, was developed, with the two Cl atoms in vismodegib derived from waste PVC water pipe. LCA indicates that this route has a lower environmental impact than the reported chlorination process^39,41^. Thus, this study charts a path for chlorinated wastes to be ‘decommissioned’ in a safe and sustainable way, simultaneously adding value to the process and additionally eliminating the need to manufacture unsustainable chlorination reagents. Note that chlorine production is the main electricity-consuming process in the chemical industry^80^ and is not sustainable unless the process is powered by renewable energy, hence using ‘reclaimed’ chlorine is advantageous. Future work will be focused on scaling up the reaction and extending the substrate scope, that is, to arenes without directing groups. Should it prove possible to chlorinate a broader range of arenes using this approach, then substantially larger quantities of chlorinated hydrocarbon waste could be valorized, helping to bridge the gap between chlorinated hydrocarbon waste and its application as chlorination reagents.

## Methods

### General procedures

7,8-Benzoquinoline **1a** (0.25 mmol), PVC water pipe (1.5 equiv., 0.375 mmol), Cu(NO_3_)_2_**·**3H_2_O (20%, 0.05 mmol), PdO (5%, 0.0125 mmol), NaNO_3_ (20%, 0.05 mmol), biphenyl (0.2 mmol, internal standard) and DMSO (2 ml) were added into a glass insert vial of the autoclave and charged with O_2_ (3 bar). The reactor was heated to 140 °C for 15 h. After cooling to room temperature, the gas products were collected with a Tedlar gas-sampling bag (Sigma-Aldrich) for analysis by GC. Ethyl acetate (3 × 3 ml) and an aqueous saturated brine solution (5 ml) was added to the reaction mixture to extract the organic products. The combined organic phase was used for analysis. Further purification was achieved using silica gel chromatography (hexane/ethyl acetate as eluent if not specified) when required.

### Analytical methods

Qualitative and quantitative analysis of gas-phase products was performed by GC using an Agilent 7890B instrument equipped with a hydrogen flame-ionization detector and a thermal conductivity detector. The GC yield was determined based on standard gas mixtures and integrated peak areas. Qualitative and quantitative analysis of crude liquid products was performed using an Agilent 7000C GCMS equipped with a hydrogen flame-ionization detector, electron ionization mass detector and HP-5 non-polar column. The GC yield was determined on the basis of internal standard curves and integrated peak areas.

Determination of the molecular weight and polydispersity of the polymers was performed using GPC on an Agilent 390-MDS equipped with a refractive index detector, and dual-angle light scattering detector. THF was used as eluent. For the measurement after reaction, brine (10 ml) was added to the DMSO solution (2 ml) and the organic components were extracted with THF (3 × 5 ml). The THF extract was used for GPC.

^1^H and ^13^C NMR spectra were recorded on a Bruker Avance III HD 400 instrument equipped with a 5 mm BBFO probe. DMSO-d_6_ or CDCl_3_ were used as solvent. DMSO-d_6_ was used as the reaction solvent for the reaction that was monitored by ^1^H NMR spectroscopy. After centrifuging to remove the PdO catalyst, the crude reaction mixture (0.2 ml) was diluted with DMSO-d_6_ or THF-d_8_ (0.4 ml) and directly used for the ^1^H NMR experiment.

Other methods are included in the Supplementary Information.

## Online content

Any methods, additional references, Nature Portfolio reporting summaries, source data, extended data, supplementary information, acknowledgements, peer review information; details of author contributions and competing interests; and statements of data and code availability are available at 10.1038/s41557-024-01462-8.

### Supplementary information

Supplementary Information Supplementary figures, tables, materials and methods, life cycle assessment, NMR spectra and references.

## Data Availability

All data generated or analysed during this study are included in this published paper and its Supplementary Information.
