# Supplementary material for: Tandem catalysis enables chlorine-containing waste as chlorination reagents
Source: Nat Chem. 2024 Feb 23;16(5):700–8. doi: 10.1038/s41557-024-01462-8 (PMC11087255; doi:10.1038/s41557-024-01462-8)
Supplement: Supplementary file 1 — Supplementary figures, tables, materials and methods, life cycle assessment, NMR spectra and references. [file 41557_2024_1462_MOESM1_ESM.pdf]

nature chemistry

Article

https://doi.org/10.1038/s41557-024-01462-8

Tandem catalysis enables chlorine-containing waste as chlorination reagents

In the format provided by the authors and unedited

Contents

Materials and Methods 2
- Materials 2
- Typical reaction (standard condition) 2
- Substrate preparation 8
- Analytical Methods 10

Supplementary Figures 11

Life cycle assessment (LCA) 33
- Methods 33
- Assumptions 33

NMR spectra 37
- Substrate 37
- Product 67

References 108

Materials and Methods

Materials

Inorganic salts including Cu salts, Pd salts and nitrates, solvents, alkyl chlorides and arene (if not specified) were purchased from Sigma-Aldrich and used directly unless specified otherwise. Pure polymers including polyvinylidene chloride (PVDC) M_w ~50,000/100000/250000, polychloroprene (chloroprene rubber, CR), polyepichlorohydrin (PECH) M_w ~700,000, poly(epichlorohydrin-co-ethylene oxide) (PECHEO, epichlorohydrin 64-69 wt.%) and poly(epichlorohydrin-co-CO_2) (PECHC) were purchased from Sigma-Aldrich. Compressed air, N_2 and O_2 was purchased from Carbagas. PVC or neoprene-based water pipe, electrical conduit, electric wire and vacuum tube were obtained from the chemical store at EPFL. PVDC blisters (Ibuprofen N Zentiva) was purchased from SUN STORE Pharmacies in Lausanne.

Typical reaction (standard condition)

Typical reaction using PVC water pipe as the chlorination reagent:

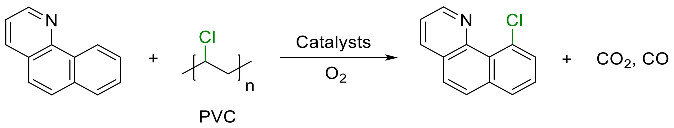

7,8-Benzoquinoline **1a** (0.25 mmol), PVC water pipe (1.5 equiv., 0.375 mmol), $Cu(NO_3)_2$•$3H_2O$ (20%, 0.05 mmol), PdO (5%, 0.0125 mmol), $NaNO_3$ (20%, 0.05 mmol), biphenyl (0.2 mmol, internal standard) and DMSO (2 mL) were added into glass insert vial of autoclave and charged with O_2 (3 bar). The reactor was heated to 140 ºC for 15 h. After cooling to room temperature, the gas products were collected with a Tedlar® gas sampling bag (Sigma-Aldrich) for analysis by gas chromatography (GC). Ethyl acetate (EA, 3×3 mL) and an aqueous saturated brine solution (5 mL) was added to the reaction mixture to extract the organic products. The combined organic phase was used for analysis. Further purification was achieved using silica gel chromatography (Hexane/ethyl acetate as eluent if not specified) when required.

Process mass intensity (PMI) = Total mass in process / Mass of products = [M(substrate) + M(PVC) + M($Cu(NO_3)_2$•$3H_2O$) + M(PdO) + M($NaNO_3$) + M(O_2)] / [M(product) + M(CO)]

Environmental factor (E-factor) = Mass of waste / Mass of products = [M(substrate) + M(PVC) + M($Cu(NO_3)_2$•$3H_2O$) + M(PdO) + M($NaNO_3$) + M(O_2) − M(product) − M(CO)] / [M(product) + M(CO)]

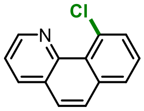

1b, 10-Chlorobenzoquinoline

^{1}H NMR (400 MHz, DMSO) δ 9.07 (dd, *J* = 4.3, 1.9 Hz, 1H), 8.46 (dd, *J* = 8.0, 1.9 Hz, 1H), 8.07 (dd, *J* = 7.9, 1.4 Hz, 1H), 8.01 (d, *J* = 8.8 Hz, 1H), 7.95 (d, *J* = 8.8 Hz, 1H), 7.87 (dd, *J* = 7.7, 1.3 Hz, 1H), 7.77 – 7.66 (m, 2H). ^{13}C NMR (101 MHz, DMSO) δ 148.31, 145.85, 136.59, 136.53, 131.83, 131.51, 128.64, 128.50, 127.76, 127.48, 126.97, 122.76.

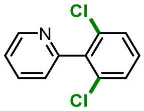

2b, 2-(2,6-dichlorophenyl)pyridine

^{1}H NMR (400 MHz, DMSO) δ 8.73 – 8.68 (m, 1H), 7.94 (t, *J* = 7.7 Hz, 1H), 7.59 (d, *J* = 8.1 Hz, 2H), 7.46 (dt, *J* = 17.0, 8.3 Hz, 3H). ^{13}C NMR (101 MHz, DMSO) δ 155.29, 149.96, 138.66, 137.32, 134.07, 131.23, 128.82, 125.31, 123.86.

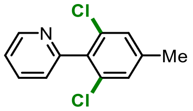

3b, 2-(2,6-dichloro-4-methylphenyl)pyridine

^{1}H NMR (400 MHz, DMSO) δ 8.70 (m, *J* = 4.9, 1.8, 1.0 Hz, 1H), 7.91 (td, *J* = 7.7, 1.8 Hz, 1H), 7.47 – 7.34 (m, 4H), 2.35 (d, *J* = 0.9 Hz, 3H). ^{13}C NMR (101 MHz, DMSO) δ 155.35, 149.91, 141.48, 137.15, 135.81, 133.65, 129.13, 125.45, 123.67, 20.65.

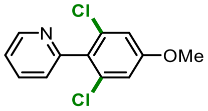

4b, 2-(2,6-dichloro-4-methoxyphenyl)pyridine

^{1}H NMR (400 MHz, DMSO) δ 8.68 (dt, *J* = 4.8, 1.4 Hz, 1H), 7.91 (td, *J* = 7.7, 1.9 Hz, 1H), 7.47 – 7.35 (m, 2H), 7.19 (s, 2H), 3.85 (s, 3H). ^{13}C NMR (101 MHz, DMSO) δ 160.08, 155.22, 149.89, 137.19, 134.56, 131.19, 125.84, 123.65, 114.63, 56.62.

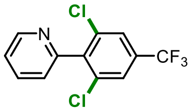

5b, 2-(2,6-dichloro-4-(trifluoromethyll)phenyl)pyridine

^{1}H NMR (400 MHz, DMSO) δ 8.74 (m, *J* = 4.8, 1.8, 1.1 Hz, 1H), 8.03 (d, *J* = 0.8 Hz, 2H), 7.98 (td, *J* = 7.7, 1.8 Hz, 1H), 7.49 (m, *J* = 7.7, 5.6, 1.1 Hz, 2H). ^{13}C NMR (101 MHz, DMSO) δ 154.24, 150.17, 142.50, 137.53, 135.34, 132.08, 131.74, 131.41, 131.08, 127.06, 125.82, 125.78, 125.75, 125.71, 125.03, 124.34, 124.27, 121.63, 118.91.

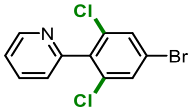

6b, 2-(2,6-dichloro-4-bromophenyl)pyridine

^{1}H NMR (400 MHz, DMSO) δ 8.71 (m, *J* = 4.9, 1.9, 1.0 Hz, 1H), 7.95 (td, *J* = 7.7, 1.8 Hz, 1H), 7.91 (s, 2H), 7.51 – 7.40 (m, 2H). ^{13}C NMR (101 MHz, DMSO) δ 154.48, 150.10, 138.03, 137.45, 135.09, 131.27, 125.32, 124.09, 122.46.

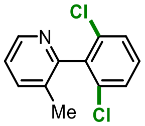

7b, 2-(2,6-dichloro-phenyl)-3-methyl-pyridine

^{1}H NMR (400 MHz, DMSO) δ 8.52 (m, *J* = 4.8, 1.6, 0.7 Hz, 1H), 7.80 (m, *J* = 7.7, 1.7, 0.9 Hz, 1H), 7.62 (dd, *J* = 8.0, 0.8 Hz, 2H), 7.50 (dd, *J* = 8.8, 7.3 Hz, 1H), 7.39 (dd, *J* = 7.8, 4.8 Hz, 1H), 2.03 (s, 3H). ^{13}C NMR (101 MHz, DMSO) δ 154.91, 147.55, 138.41, 137.99, 133.91, 132.09, 131.24, 128.81, 124.12, 18.05.

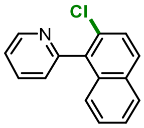

8b, 2-(2-chloronaphthalen-1-yl)pyridine

^{1}H NMR (400 MHz, $CDCl_3$) δ 8.76 (m, *J* = 4.9, 1.8, 1.0 Hz, 2H), 7.79 (m, *J* = 8.5, 6.9, 4.9 Hz, 6H), 7.48 (d, *J* = 8.8 Hz, 2H), 7.45 – 7.24 (m, 10H). ^{13}C NMR (101 MHz, $CDCl_3$) δ 156.66, 149.83, 136.41, 136.10, 133.24, 132.12, 130.70, 129.76, 128.04, 127.18, 126.01, 125.69, 122.67,

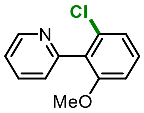

9b, 2-(2-chloro-6-methoxyphenyl)pyridine

^{1}H NMR (400 MHz, $CDCl_3$) δ 8.65 (m, *J* = 4.9, 1.8, 1.0 Hz, 1H), 7.67 (td, *J* = 7.7, 1.8 Hz, 1H), 7.27 – 7.16 (m, 3H), 7.01 (dd, *J* = 8.1, 1.0 Hz, 1H), 6.81 (dd, *J* = 8.4, 1.0 Hz, 1H), 3.64 (s, 3H). ^{13}C NMR (101 MHz, $CDCl_3$) δ 158.25, 154.97, 149.47, 136.05, 134.13, 129.79, 129.20, 125.67, 122.36, 121.82, 109.53, 77.31, 56.11.

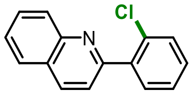

10b, 3-(2-chlorophenyl)isoquinoline

^{1}H NMR (400 MHz, $CDCl_3$) δ 8.19 – 8.07 (m, 2H), 7.81 (dd, *J* = 8.0, 1.5 Hz, 1H), 7.72 – 7.64 (m, 2H), 7.66 – 7.58 (m, 1H), 7.51 (m, *J* = 8.2, 6.9, 1.2 Hz, 1H), 7.44 (dd, *J* = 7.2, 2.0 Hz, 1H), 7.33 (pd, *J* = 7.4, 1.8 Hz, 2H). ^{13}C NMR (101 MHz, $CDCl_3$) δ 157.45, 148.11, 139.70, 135.67, 132.39, 131.71, 130.11, 129.89, 129.72, 129.69, 127.57, 127.19, 127.15, 126.79, 122.79, 77.23.

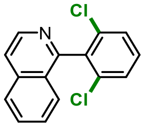

11b, 1-(2,6-dichlorophenyl)isoquinoline

^{1}H NMR (400 MHz, $CDCl_3$) δ 8.61 (d, *J* = 5.7 Hz, 1H), 7.86 (dt, *J* = 8.3, 1.1 Hz, 1H), 7.72 – 7.59 (m, 2H), 7.51 – 7.38 (m, 4H), 7.32 (dd, *J* = 8.9, 7.2 Hz, 1H). ^{13}C NMR (101 MHz, $CDCl_3$) δ 156.70, 142.49, 136.96, 136.36, 135.20, 130.45, 130.24, 128.18, 127.80, 127.16, 126.04, 121.01, 77.23.

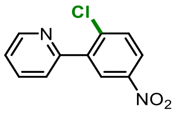

12b, 2-(2-chloro-5-nitrophenyl)pyridine

^{1}H NMR (500 MHz, $CDCl_3$) δ 8.79 (dd, *J* = 4.3, 2.6 Hz, 1H), 8.54 (d, *J* = 2.7 Hz, 1H), 8.22 (dt, *J* = 8.8, 1.8 Hz, 1H), 7.86 (td, *J* = 7.7, 1.8 Hz, 1H), 7.75 – 7.70 (m, 1H), 7.68 (d, *J* = 8.7 Hz, 1H), 7.40 (m, *J* = 7.6, 5.0, 1.3 Hz, 1H). ^{13}C NMR (126 MHz, $CDCl_3$) δ 154.60, 150.03, 146.74, 140.45, 139.09, 136.34, 131.22, 126.78, 124.73, 124.08, 123.40.

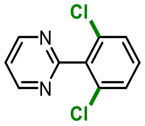

13b, 2-(2,6-dichlorophenyl)pyrimidine

^{1}H NMR (400 MHz, DMSO) δ 9.01 (d, *J* = 5.0 Hz, 2H), 7.66 – 7.59 (m, 3H), 7.54 (dd, *J* = 9.0, 7.1 Hz, 1H). ^{13}C NMR (101 MHz, DMSO) δ 163.64, 158.35, 137.82, 133.32, 131.65, 128.78, 121.38.

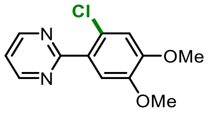

14b, 2-(2-chloro-4,5-dimethoxyphenyl)pyrimidine

^{1}H NMR (400 MHz, $CDCl_3$) δ 8.89 (d, *J* = 4.9 Hz, 2H), 7.39 (s, 1H), 7.28 (t, *J* = 4.9 Hz, 1H), 7.01 (s, 1H), 3.96 (d, *J* = 1.8 Hz, 6H). ^{13}C NMR (101 MHz, $CDCl_3$) δ 165.34, 157.23, 157.01, 150.39, 147.83, 129.50, 124.69, 118.95, 114.06, 113.52, 77.23, 56.28, 56.18.

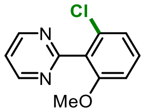

15b, 2-(2-chloro-6-methoxyphenyl)pyrimidine

^{1}H NMR (400 MHz, $CDCl_3$) δ 8.83 (d, *J* = 4.9 Hz, 2H), 7.30 – 7.17 (m, 2H), 7.02 (dd, *J* = 8.1, 0.9 Hz, 1H), 6.84 (dd, *J* = 8.4, 0.9 Hz, 1H), 3.68 (s, 3H). ^{13}C NMR (101 MHz, $CDCl_3$) δ 164.23, 158.11, 157.31, 133.54, 130.30, 128.29, 121.77, 119.55, 109.60, 77.23, 56.16.

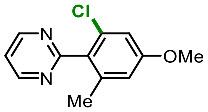

16b, 2-(2-chloro-4-methoxy-6-methylphenyl)pyrimidine

^{1}H NMR (400 MHz, $CDCl_3$) δ 8.80 (d, *J* = 4.9 Hz, 2H), 7.21 (t, *J* = 4.9 Hz, 1H), 6.79 (d, *J* = 2.5 Hz, 1H), 6.66 (dd, *J* = 2.4, 0.9 Hz, 1H), 3.74 (s, 3H), 2.03 (s, 3H). ^{13}C NMR (101 MHz, $CDCl_3$) δ 166.22, 159.75, 157.24, 139.05, 133.27, 131.14, 119.27, 114.77, 112.23, 55.52, 20.34.

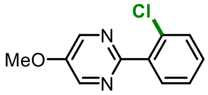

17b, 2-(2-chlorophenyl)-5-methoxypyrimidine

^{1}H NMR (500 MHz, $CDCl_3$) δ 8.56 (s, 3H), 7.72 (dd, *J* = 6.1, 3.4 Hz, 2H), 7.55 – 7.48 (m, 2H), 7.45 – 7.28 (m, 4H), 4.01 (s, 5H). ^{13}C NMR (126 MHz, $CDCl_3$) δ 158.22, 151.90, 143.53, 143.20, 137.44, 132.63, 131.55, 130.51, 130.03, 128.08, 126.79, 56.03.

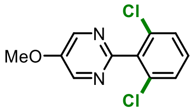

17b', 2-(2,6-dichlorophenyl)-5-methoxypyrimidine

^{1}H NMR (500 MHz, $CDCl_3$) δ 8.58 (s, 2H), 7.42 (d, *J* = 8.0 Hz, 2H), 7.30 (dd, *J* = 14.8, 6.5 Hz, 1H), 4.02 (s, 3H). ^{13}C NMR (126 MHz, $CDCl_3$) δ 156.51, 152.19, 143.52, 137.29, 134.49, 130.12, 128.08, 56.03.

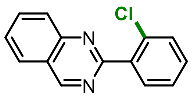

18b, 2-(2-chlorophenyl)quinazoline

^{1}H NMR (500 MHz, $CDCl_3$) δ 9.58 (d, *J* = 16.0 Hz, 1H), 8.16 (d, *J* = 8.2 Hz, 1H), 8.09 – 7.96 (m, 3H), 7.89 – 7.82 (m, 1H), 7.82 – 7.70 (m, 1H), 7.60 – 7.53 (m, 1H), 7.50 – 7.33 (m, 3H). ^{13}C NMR (126 MHz, $CDCl_3$) δ 162.02, 160.29, 150.38, 138.30, 134.64, 134.44, 132.92, 131.81, 130.58, 130.36, 130.25, 128.71, 128.68, 128.54, 128.19, 128.11, 127.31, 127.19, 126.93, 123.31.

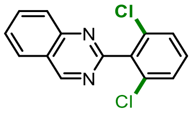

18b', 2-(2,6-dichlorophenyl)quinazoline

^{1}H NMR (500 MHz, $CDCl_3$) δ 9.59 (s, 1H), 8.18 (d, *J* = 8.5 Hz, 1H), 8.09 – 7.99 (m, 2H), 7.78 (m, *J* = 8.1, 6.9, 1.1 Hz, 1H), 7.47 (d, *J* = 8.1 Hz, 2H), 7.36 (dd, *J* = 8.7, 7.5 Hz, 1H). ^{13}C NMR (126 MHz, $CDCl_3$) δ 160.86, 160.38, 150.35, 137.90, 134.64, 134.26, 130.25, 128.71, 128.53, 128.19, 127.30, 123.61.

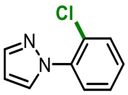

19b, 1-(2-chlorophenyl)-1H-pyrazole

^{1}H NMR (400 MHz, $CDCl_3$) δ 7.81 (d, *J* = 2.4 Hz, 3H), 7.68 (d, *J* = 1.9 Hz, 3H), 7.56 – 7.47 (m, 3H), 7.47 – 7.35 (m, 3H), 7.35 – 7.21 (m, 6H), 6.47 – 6.37 (m, 3H). ^{13}C NMR (101 MHz, $CDCl_3$) δ 140.93, 131.30, 130.66, 130.63, 129.00, 128.68, 128.37, 127.82, 127.67, 106.69, 106.66.

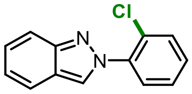

20b, 2-(2-chlorophenyl)-2H-indazole

^{1}H NMR (400 MHz, $CDCl_3$) δ 8.27 (d, *J* = 1.0 Hz, 1H), 7.76 – 7.58 (m, 3H), 7.57 – 7.46 (m, 1H), 7.44 – 7.24 (m, 3H), 7.07 (m, *J* = 8.5, 6.6, 0.9 Hz, 1H). ^{13}C NMR (101 MHz, $CDCl_3$) δ 149.43, 138.64, 130.70, 129.97, 129.00, 128.59, 127.71, 126.95, 125.23, 122.45, 122.02, 120.53, 117.95, 77.23.

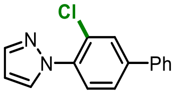

21b, 1-(3-chloro-[1,1'-biphenyl]-4-yl)-1H-pyrazole

^{1}H NMR (400 MHz, $CDCl_3$) δ 7.86 (dd, *J* = 2.4, 0.6 Hz, 1H), 7.69 (dd, *J* = 11.4, 1.9 Hz, 2H), 7.63 – 7.48 (m, 4H), 7.46 – 7.29 (m, 3H), 6.43 (dd, *J* = 2.5, 1.9 Hz, 1H). ^{13}C NMR (101 MHz, $CDCl_3$) δ 142.22, 141.01, 138.79, 137.13, 131.32, 129.11, 129.06, 128.40, 128.27, 127.92, 127.12, 126.30, 106.75, 77.23.

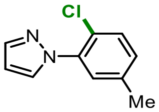

22b, 1-(2-chloro-5-methylphenyl)-1H-pyrazole

^{1}H NMR (400 MHz, $CDCl_3$) δ 7.90 (d, *J* = 2.5 Hz, 1H), 7.76 (d, *J* = 1.8 Hz, 1H), 7.46 – 7.38 (m, 2H), 7.16 (dd, *J* = 8.4, 2.2 Hz, 1H), 6.49 (t, *J* = 2.2 Hz, 1H), 2.40 (s, 3H). ^{13}C NMR (101 MHz, $CDCl_3$) δ 140.79, 138.01, 137.79, 131.29, 130.27, 129.72, 128.26, 124.97, 106.55, 77.23, 20.77.

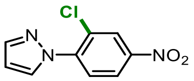

23b, 1-(2-chloro-4-nitrophenyl)-1*H*-pyrazole

^{1}H NMR (500 MHz, $CDCl_3$) δ 8.69 (d, *J* = 2.5 Hz, 3H), 8.55 (dd, *J* = 9.1, 2.5 Hz, 3H), 8.42 (d, *J* = 2.7 Hz, 3H), 8.15 (d, *J* = 9.1 Hz, 3H), 7.92 (d, *J* = 1.7 Hz, 3H), 6.67 (t, *J* = 2.1 Hz, 3H), 1.37 – 1.26 (m, 2H), 0.93 – 0.83 (m, 1H). ^{13}C NMR (126 MHz, $CDCl_3$) δ 145.64, 145.05, 143.91, 130.42, 129.60, 128.88, 124.21, 115.54, 110.33, 104.28.

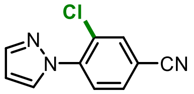

24b, 3-chloro-4-(1H-pyrazol-1-yl)benzonitrile

^{1}H NMR (400 MHz, $CDCl_3$) δ 8.01 (dd, *J* = 2.6, 0.6 Hz, 1H), 7.80 – 7.70 (m, 3H), 7.61 (dd, *J* = 8.4, 1.8 Hz, 1H), 6.47 (dd, *J* = 2.6, 1.8 Hz, 1H). ^{13}C NMR (101 MHz, $CDCl_3$) δ 142.15, 141.55, 134.60, 131.41, 131.24, 127.86, 127.55, 116.90, 112.18, 107.93, 77.23.

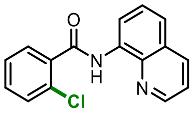

25b, 2-chloro-N-(quinolin-8-yl)benzamide

^{1}H NMR (400 MHz, $CDCl_3$) δ 10.61 (s, 1H), 8.85 – 8.78 (m, 2H), 8.52 (dd, *J* = 8.6, 1.7 Hz, 1H), 8.04 – 7.96 (m, 2H), 7.58 (d, *J* = 8.4 Hz, 1H), 7.56 – 7.43 (m, 4H). ^{13}C NMR (101 MHz, $CDCl_3$) δ 165.44, 148.79, 139.34, 134.91, 133.90, 133.50, 132.03, 128.87, 127.34, 127.30, 126.04, 124.51, 122.44, 116.49, 77.24.

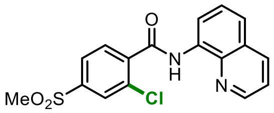

26b, 2-chloro-4-(methylsulfonyl)-N-(quinolin-8-yl)benzamide

^{1}H NMR (500 MHz, $CDCl_3$) δ 10.51 (s, 1H), 8.93 (p, *J* = 4.5 Hz, 1H), 8.81 (dd, *J* = 4.1, 1.9 Hz, 1H), 8.22 (dd, *J* = 8.2, 1.9 Hz, 1H), 8.11 (d, *J* = 1.8 Hz, 1H), 8.03 – 7.95 (m, 2H), 7.63 (d, *J* = 4.5 Hz, 2H), 7.50 (dd, *J* = 8.2, 4.1 Hz, 1H), 3.14 (d, *J* = 1.7 Hz, 3H). ^{13}C NMR (126 MHz, $CDCl_3$) δ 163.16, 148.60, 143.21, 140.64, 138.51, 136.50, 133.87, 132.60, 131.02, 129.54, 128.00, 127.35, 126.09, 122.81, 121.95, 117.17, 44.48.

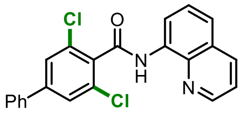

27b. 3,5-dichloro-N-(quinolin-8-yl)-[1,1'-biphenyl]-4-carboxamide

^{1}H NMR (500 MHz, $CDCl_3$) δ 10.13 (s, 1H), 9.01 (dd, *J* = 7.2, 1.8 Hz, 1H), 8.81 (dd, *J* = 4.3, 1.6 Hz, 1H), 8.22 (dd, *J* = 8.3, 1.7 Hz, 1H), 7.69 – 7.58 (m, 6H), 7.53 (d, *J* = 7.4 Hz, 1H), 7.53 – 7.44 (m, 3H). ^{13}C NMR (126 MHz, $CDCl_3$) δ 162.69, 148.45, 144.51, 138.49, 137.92, 136.43, 134.58, 134.01, 132.83, 129.21, 128.88, 128.03, 127.45, 127.15, 126.84, 122.51, 121.81, 117.22.

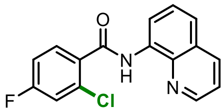

28b', 2-chloro-4-fluoro-N-(quinolin-8-yl)benzamide

^{1}H NMR (500 MHz, $CDCl_3$) δ 10.38 (s, 1H), 9.31 (dt, *J* = 8.9, 1.5 Hz, 1H), 9.07 – 9.01 (m, 1H), 8.98 – 8.89 (m, 1H), 8.64 (dd, *J* = 8.8, 1.6 Hz, 1H), 8.61 (s, 0H), 7.80 – 7.74 (m, 1H), 7.30 – 7.17 (m, 4H). ^{13}C NMR (126 MHz, $CDCl_3$) δ 163.43, 162.44, 149.29, 139.81, 139.59, 137.60, 133.64, 133.41, 127.51, 124.83, 121.75, 116.49, 116.29, 114.51, 77.25.

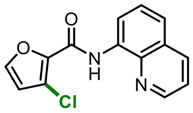

29b, 3-chloro-N-(quinolin-8-yl)furan-2-carboxamide

^{1}H NMR (500 MHz, $CDCl_3$) δ 10.75 (s, 1H), 8.95 (dd, *J* = 4.1, 1.6 Hz, 1H), 8.84 (d, *J* = 8.3 Hz, 1H), 8.61 (dd, *J* = 8.6, 1.8 Hz, 1H), 7.69 – 7.63 (m, 2H), 7.62 (dd, *J* = 8.5, 4.2 Hz, 1H), 7.33 (d, *J* = 3.5 Hz, 1H), 6.62 (dd, *J* = 3.5, 1.7 Hz, 1H). ^{13}C NMR (126 MHz, $CDCl_3$) δ 156.35, 148.91, 148.17, 144.66, 139.21, 133.50, 133.43, 127.28, 126.04, 124.65, 122.47, 116.57, 115.40, 112.55.

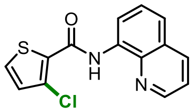

30b, 3-chloro-N-(quinolin-8-yl)thiophene-2-carboxamide

^{1}H NMR (400 MHz, $CDCl_3$) δ 11.29 (s, 1H), 8.85 (dd, *J* = 4.2, 1.6 Hz, 1H), 8.77 (d, *J* = 8.4 Hz, 1H), 8.53 (dd, *J* = 8.6, 1.6 Hz, 1H), 7.61 – 7.46 (m, 3H), 7.02 (d, *J* = 5.2 Hz, 1H). ^{13}C NMR (101 MHz, $CDCl_3$) δ 158.69, 149.02, 139.47, 133.96, 133.82, 133.37, 130.20, 129.86, 127.27, 126.04, 125.02, 124.24, 122.46, 117.25, 77.23.

Substrate preparation

Synthesis of (3-chlorobutyl)benzene, **C13**:[1]

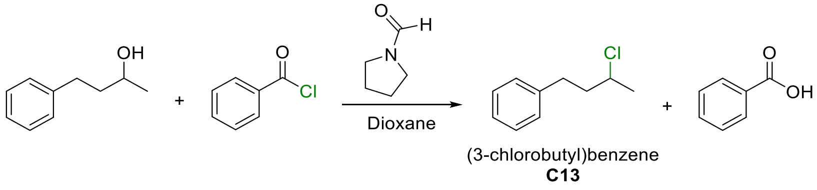

4-Phenyl-2-butanol (2 mmol), benzoyl chloride (1.2 equiv., 2 mmol), formyl pyrrolidine (20 mol%, 0.4 mmol) and dioxane (2 mL) were added to a cylindrical pressure vessel and heated at 80 ºC for 24 h. The reaction mixture was concentrated under vacuum and purified with silica gel chromatography to afford pure **C13** (yield: 80%).

Synthesis of 2,4-dichloropentane **5c**:[2]

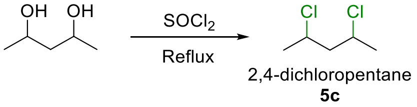

Thionyl chloride ($SOCl_2$, 50 g) was slowly added to a solution of pyridine (1 g) and 2,4-pentanediol (8 g) at -10 ºC. After addition, the reaction mixture was heated at reflux for 3 h. After cooling to room temperature, ice water (200 mL) was added to the reaction mixture and the product extracted with diethyl ether (3×200 mL). The organic phase was washed with aqueous $NaHCO_3$ and water and dried with Na_2SO_4. Pure product was obtained by fractional distillation (b.p. 140 ºC, yield: 63%).

Synthesis of (chloro(cyclopropyl)methyl)benzene **9c**:[3]

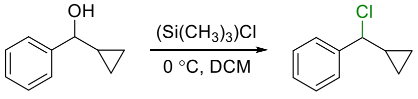

9c

Trimethylchlorosilane (30.0 mmol, 5.0 equiv.) was added dropwise into a solution of 1-phenyl-1-cyclopropylmethanol (6.0 mmol) in dry dichloromethane (DCM, 6 mL) at 0 ºC under N_2. The reaction was stirred at 0 ºC for 1 h, then quenched with water (20 mL) and the product extracted with DCM (3×20 mL). The organic phase was washed with brine, dried and concentrated in vacuo to give a colorless oil. The product was purified using silica gel chromatography.

Synthesis of pyridine and pyrimidine directing arenes:[4]

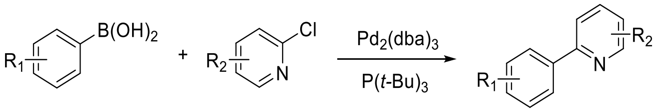

Aryl chloride (2 mmol), boronic acid (2.4 mmol), tris(dibenzylideneacetone)dipalladium(0) ($Pd_2(dba)_3$, 0.03 mmol. 1.5 mol%), P(*t*-Bu)$_3$ (0.08 mmol. 4 mol%), Cs_2CO_3 (5 mmol) and dioxane (5 mL) were added to a Schlenk tube charged with N_2 and heated at 80 ºC for 5 h. After cooling to room temperature, the reaction mixture was concentrated under vacuum and the product purified using silica gel chromatography.

Synthesis of pyrazol directing arenes:[5]

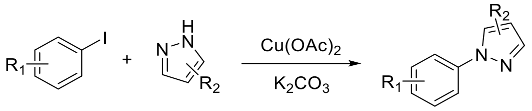

Aryl iodide (2.4 mmol), pyrazol (2.0 mmol), Cs_2CO_3 (4 mmol) and DMF (5 mL) were added to a Schlenk tube charged with N_2 and heated at 110 ºC for 24 h. After cooling to ambient temperature, a saturated aqueous NH_4Cl solution (20 mL) was added and the product extracted with EA (3×20 mL). The organic phase was washed with brine and water, dried with Na_2SO_4 and purified using silica gel chromatography.

Synthesis of 8-aminoquinoline directing arenes:[6]

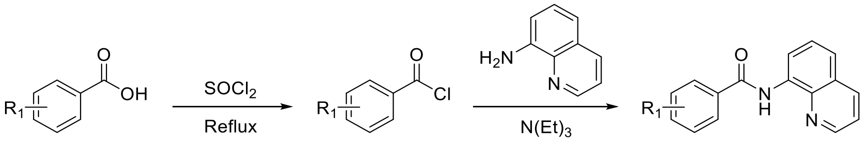

The carboxylic acid (12 mmol) and $SOCl_2$ (11 mL) were added heated under reflux for 3 h at 85 ºC. After cooling to ambient temperature, the reaction mixture was concentrated under vacuum to afford the crude acyl chloride. Dry DCM (20 mL) was added under a N_2 atmosphere and 8-aminoquinoline (10 mmol) and NEt_3 (11 mmol) were dissolved in DCM (20 mL), and slowly added dropwise to the acyl chloride solution.

The reaction was stirred at room temperature for 6 h. After reaction, the mixture was concentrated under vacuum and purified using silica gel chromatography.

Synthesis of benzo[h]quinolinyl Pd(II) chloride dimer:[7]

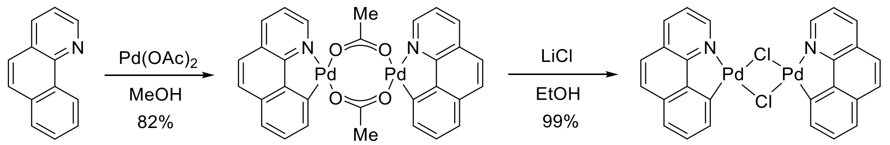

$Pd(OAc)_2$ (6 mmol, 1 equiv.) and benzo[h]quinoline **1a** (6 mmol, 1 equiv.) was added into a MeOH (80 mL) solution, stirred at RT for 8 h. The yellow precipitate was isolated by filtration and washed with MeOH (3×20 mL) and Et_2O (3×20 mL), giving benzo[h]quinolinyl Pd(II) acetate dimer in 82% yield. LiCl (20 mmol, 20 equiv.) was added into a benzo[h]quinolinyl Pd(II) acetate dimer (1 mmol, 1 equiv.) and EtOH (10 mL) suspension at 0 ºC. Then the ice-water bath was removed and the suspension was stirred at RT for another 1 h. The pale yellow precipitate was isolated by filtration and washed with water (3×20 mL), MeOH (3×20 mL), and Et_2O (3×20 mL), giving the benzo[h]quinolinyl Pd(II) chloride dimer in 99% yield.

Analytical Methods

Qualitative and quantitative analysis of gas phase products was performed by gas chromatography (GC) using an Agilent 7890B instrument equipped with a hydrogen flame-ionization detector (FID) and a thermal conductivity detector (TCD). The GC yield was determined based on standard gas mixtures and integrated peak areas. Qualitative and quantitative analysis of crude liquid products was performed using an Agilent 7000C GCMS equipped with a hydrogen flame-ionization detector (FID), electron ionization mass detector (EI-MS) and HP-5 nonpolar column. The GC yield was determined based on internal standard curves and integrated peak areas.

Determination of the molecular weight and polydispersity of the polymers was performed using Gel Permeation Chromatography (GPC, Agilent 390-MDS), equipped with refractive index detector (RI), and Dual-Angle Light Scattering detector. THF was used as eluent. For the measurement after reaction, to the DMSO solution (2 mL) brine (10 mL) was added and the organic components extracted with THF (3×5 mL). The THF extract was used for GPC.

^1H and ^{13}C NMR spectra recorded on a Bruker Avance III HD 400 instrument equipped with a 5 mm BBFO probe. DMSO-d_6 or $CDCl_3$ were used as solvent. DMSO-d_6 was used as the reaction solvent for the reaction that was monitored by ^1H NMR spectroscopy. After centrifuging to remove the PdO catalyst, the crude reaction mixture (0.2 mL) was diluted with DMSO-d_6 or THF-d_8 (0.4 mL) and directly used for ^1H NMR experiment.

Supplementary Figures

Supplementary Table 1. Summary of recent approaches reported for the destruction of PVC.

Year	Catalyst	Solvent	Product	Temp. (ºC)	Yield (%)	Fate of Cl	Ref.
Homogeneous catalysts							
**2000**	NaOH	NaOH	Oxalic acid	250	40	Neutralization by base	8
**2008**	Ammonia	Ammonia	Polyene compounds	230	80	Neutralization by base	9
**2010**	[Bmim]Cl	[Bmim]Cl	Polyene compounds	220	90	Post-neutralization	10
**2010**	TBAB	Na_2S/ethylene glycol	Polyene compounds	170	58	Post-neutralization	11
**2011**	$Ca(OH)_2$	Ethylene glycol	Polyene compounds	190	86	Neutralization by base	12
**2014**	$[P_{4444}][Cl]$	$[P_{4444}][Cl]$	*Trans*-polyacetylene	180	98	Post-neutralization	13
**2020**	Ionic liquids	Ionic liquids	Polyene compounds	160	85	HCl emissions	14
**2023**	$[Ph_3C][B(C_6F_5)_4]$	Benzene	Polyene compounds	110	77	Et_3SiCl	15
**2023**	Bu_4PCl and H(CO) $Rh(PPh_3)_3$	2-Butanone	Polyene compounds	180	81	HCl emissions	16
Heterogeneous catalysts							
**2007**	La-MgO	-	Polyene compounds	400	30	HCl, HOCl, emissions	17
**2016**	Al-modified graphitic-C_3N_4 (10 wt% Al)	Polyethylene glycol	Dechlorinated PVC	170	75	HCl emissions	18
**2018**	CuAl-layered double hydroxide	-	Solid	600	81	HCl, HOCl, emissions	19
**2020**	ZnFeAl-layered double hydroxide	-	Solid	300-800	98	HCl, HOCl emissions	20
**2021**	Benzimidazole modified layered double hydroxide	-	Solid	300-600	95	HCl, HOCl emissions	21
**2023**	Mg/Al mixed oxide	-	Dechlorinated PVC	250	100	HCl emissions	22

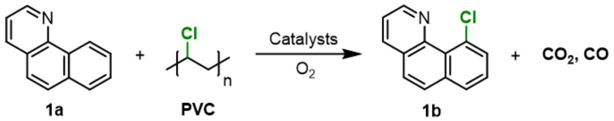

Entry	[Cu]	$NaNO_3$ (%)	Yield of **1b** (%)
1	CuO	100	4
2	$CuSO_4$	100	0
3	$Cu(OAc)_2 \cdot H_2O$	100	0
4	$Cu(NO_3)_2 \cdot 3H_2O$	100	15
5	$Cu(NO_3)_2 \cdot 3H_2O$	50	16
6	$Cu(NO_3)_2 \cdot 3H_2O$	20	19
7	$Cu(NO_3)_2 \cdot 3H_2O$	0	3

Supplementary Figure 1. Cu catalyzed aerobic chlorination using PVC as a chlorination reagent (without a Pd catalyst). Reaction conditions: 7,8-benzoquinoline **1a** (0.25 mmol), PVC (1.5 equiv., 0.375 mmol), Cu catalyst (20 mol%, 0.05 mmol), $NaNO_3$ (0-100 mol%), biphenyl (0.2 mmol, internal standard), DMSO (2 mL), O_2 (3 bar), 140 ºC, 15 h

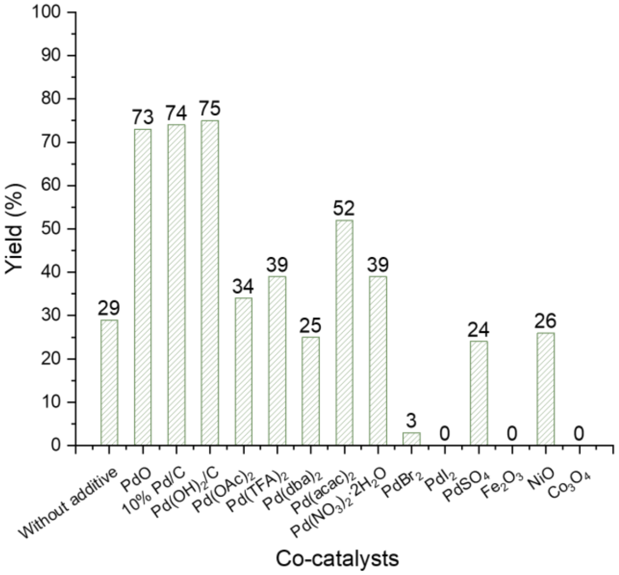

Supplementary Figure 2. Screening of Pd catalysts. Reaction conditions: 7,8-benzoquinoline **1a** (0.25 mmol), PVC (1.5 equiv., 0.375 mmol), $Cu(NO_3)_2 \cdot 3H_2O$ (20 mol%, 0.05 mmol), **Pd catalyst** (10 mol%, 0.025 mmol), $NaNO_3$ (100 mol%, 0.25 mmol), biphenyl (0.2 mmol), DMSO (2 mL), O_2 (3 bar), 140 ºC, 15 h.

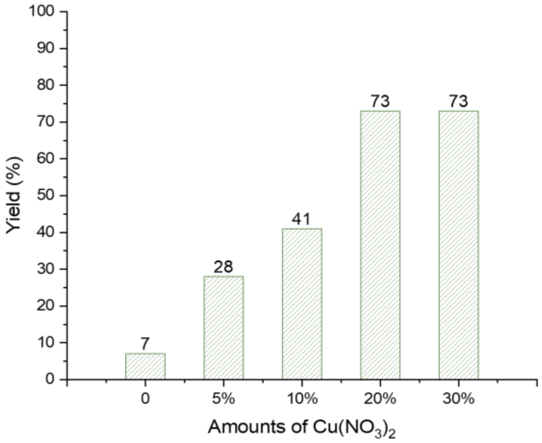

Supplementary Figure 3. Optimization of the $Cu(NO_3)_2$ concentration. Reaction conditions: 7,8-benzoquinoline **1a** (0.25 mmol), PVC (1.5 equiv., 0.375 mmol), **$Cu(NO_3)_2$•3H_2O (0-30 mol%)**, PdO (10 mol%, 0.025 mmol), $NaNO_3$ (100 mol%, 0.25 mmol), biphenyl (0.2 mmol), DMSO (2 mL), O_2 (3 bar), 140 ºC, 15 h.

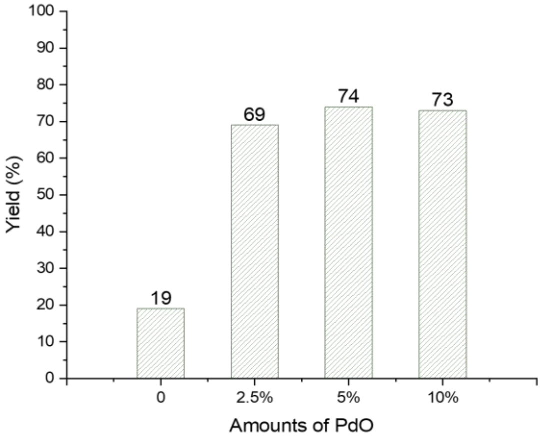

Supplementary Figure 4. Optimization of the PdO concentration. Reaction conditions: 7,8-benzoquinoline **1a** (0.25 mmol), PVC (1.5 equiv., 0.375 mmol), $Cu(NO_3)_2$•3H_2O (20 mol%, 0.05 mmol), **PdO (0-10 mol%)**, $NaNO_3$ (100 mol%, 0.25 mmol), biphenyl (0.2 mmol), DMSO (2 mL), O_2 (3 bar), 140 ºC, 15 h.

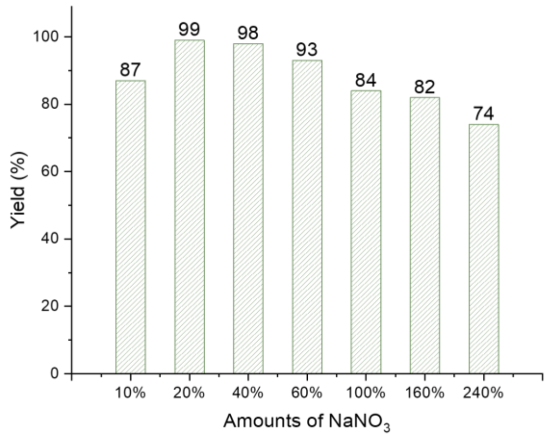

Supplementary Figure 5. Optimization of the $NaNO_3$ concentration. Reaction conditions: 7,8-benzoquinoline **1a** (0.25 mmol), PVC (1.5 equiv., 0.375 mmol), $Cu(NO_3)_2{\bullet}3H_2O$ (20 mol%, 0.05 mmol), PdO (5 mol%, 0.0125 mmol), **$NaNO_3$ (10-240 mol%)**, biphenyl (0.2 mmol), DMSO (2 mL), O_2 (3 bar), 140 ºC, 15 h.

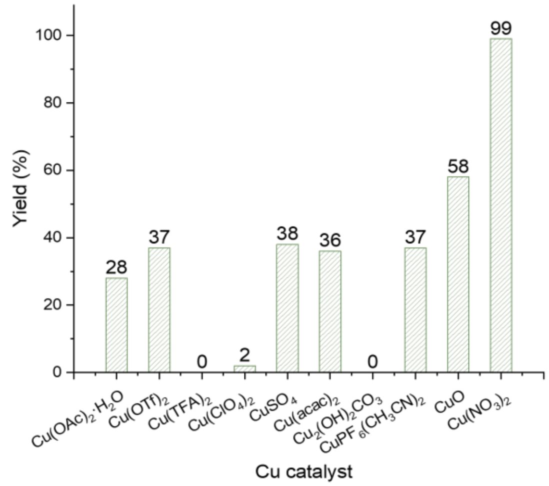

Supplementary Figure 6. Screening of Cu catalysts. Reaction conditions: 7,8-benzoquinoline **1a** (0.25 mmol), PVC (1.5 equiv., 0.375 mmol), **Cu catalyst (20 mol%, 0.05 mmol)**, PdO (5 mol%, 0.0125 mmol), $NaNO_3$ (20 mol%, 0.05 mmol), biphenyl (0.2 mmol), DMSO (2 mL), O_2 (3 bar), 140 ºC, 15 h.

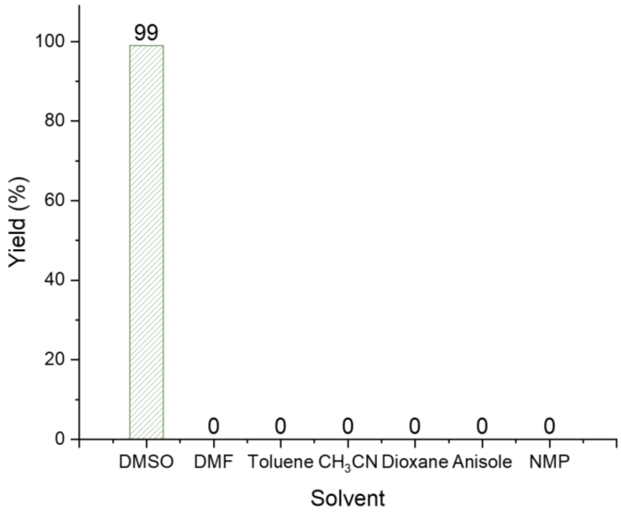

Supplementary Figure 7. Screening of the solvent. Reaction conditions: 7,8-benzoquinoline **1a** (0.25 mmol), PVC (1.5 equiv., 0.375 mmol), $Cu(NO_3)_2$•$3H_2O$ (20 mol%, 0.05 mmol), PdO (5 mol%, 0.0125 mmol), $NaNO_3$ (20 mol%, 0.05 mmol), biphenyl (0.2 mmol), **solvent (2 mL)**, O_2 (3 bar), 140 ºC, 15 h.

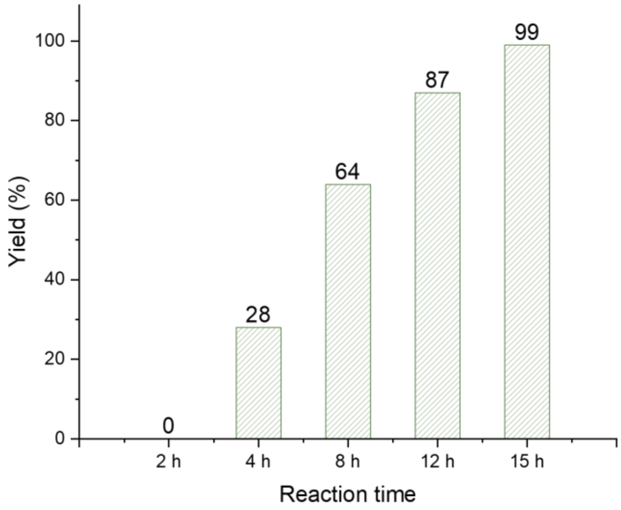

Supplementary Figure 8. Optimization of reaction time. Reaction conditions: 7,8-benzoquinoline **1a** (0.25 mmol), PVC (1.5 equiv., 0.375 mmol), $Cu(NO_3)_2$•$3H_2O$ (20 mol%, 0.05 mmol), PdO (5 mol%, 0.0125 mmol), $NaNO_3$ (20 mol%, 0. 5 mmol), biphenyl (0.2 mmol), DMSO (2 mL), O_2 (3 bar), 140 ºC, **2-15 h**.

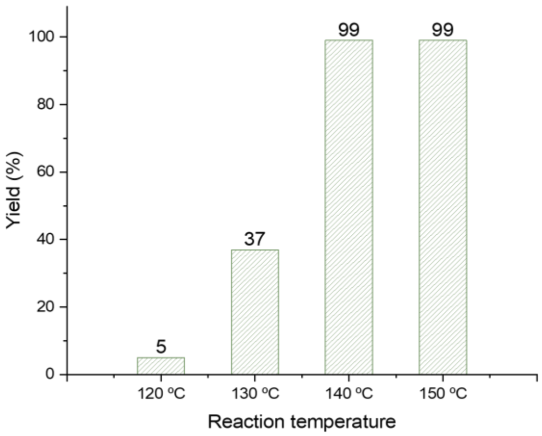

Supplementary Figure 9. Optimization of reaction temperature. Reaction conditions: 7,8-benzoquinoline **1a** (0.25 mmol), PVC (1.5 equiv., 0.375 mmol), $Cu(NO_3)_2•3H_2O$ (20 mol%, 0.05 mmol), PdO (5 mol%, 0.0125 mmol), $NaNO_3$ (20 mol%, 0.05 mmol), biphenyl (0.2 mmol), DMSO (2 mL), O_2 (3 bar), **120-150 ºC**, 15 h.

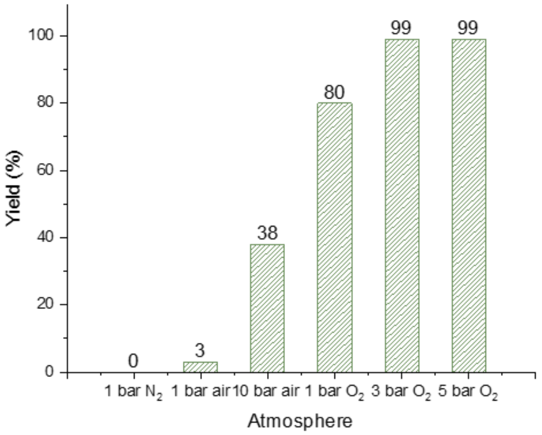

Supplementary Figure 10. Optimization of gas and pressure. Reaction conditions: 7,8-benzoquinoline **1a** (0.25 mmol), PVC (1.5 equiv., 0.375 mmol), $Cu(NO_3)_2•3H_2O$ (20 mol%, 0.05 mmol), PdO (5 mol%, 0.0125 mmol), $NaNO_3$ (20 mol%, 0.05 mmol), biphenyl (0.2 mmol), DMSO (2 mL), **gas**, 140 ºC, 15 h.

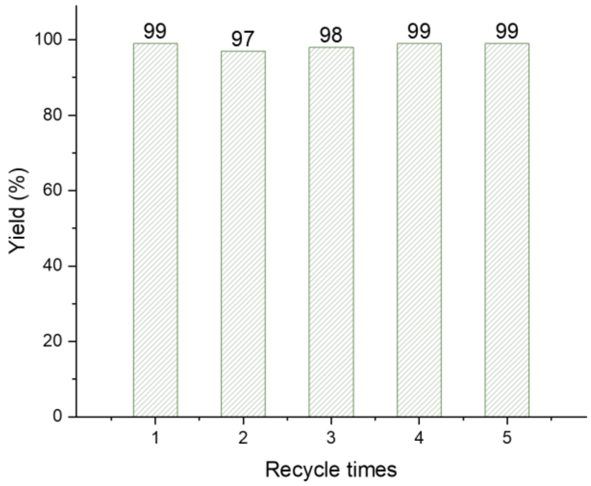

Supplementary Figure 11. Recycling of the PdO catalyst. Reactions were performed using the standard reaction conditions. PdO was recovered by filtration, and was washed with water, acetone, ethyl acetate and diethyl ether, dried under vacuum at 30 ºC for 18 h.

Supplementary Table 2. Optimization of CO production.

Entry	Variation from standard conditions		Yield of 1b (%)	Yield of CO (%)	Yield of CO_2 (%)
1	Standard conditions		99	15	81
2	With additives	50 mol% bis(benzene)chromium	72	48	50
3		50 mol% $Cr(CO)_6$	68	33	65
4		50 mol% Cr_2O_3	65	20	80
5		50 mol% $Cr_2O_3 \cdot CuO$	66	25	72
6		50 mol% $PbCl_2$	98	10	90
7		50 mol% $CaCO_3$	99	10	89
8		50 mol% Na_2SO_4	45	30	66
9		50 mol% Na_2HPO_4	92	16	83
10		50 mol% $MgCl_2$	55	32	67
11		20 equivalent H_2O	54	24	76
12	Other Pd catalysts	Lindlar catalyst instead of PdO	32	37	60
13		5% Pd/$CaCO_3$ instead of PdO	99	11	89
14		10% Pd/C instead of PdO	99	15	85
15		10% Pd/Al_2O_3 instead of PdO	99	9	90
16		5% Pd/$BaSO_4$ instead of PdO	95	5	90
17	Other gases	1 bar O_2	80	16	81
18		1 bar air	3	15	85
19		5 bar air	30	16	83
20	No PdO catalyst		12	83	17

Reaction conditions are the same as those given in Fig. 2a, entry 7.

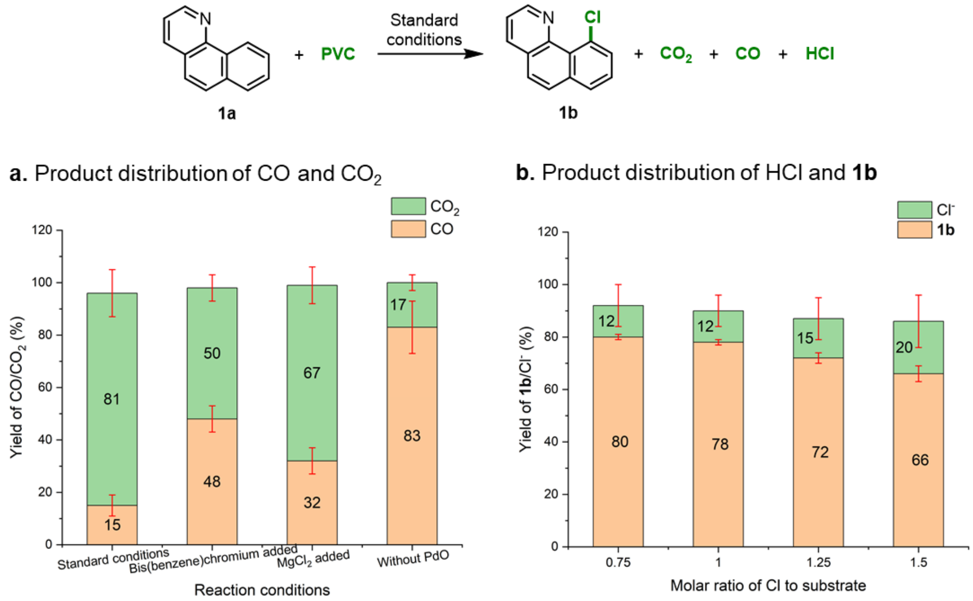

Supplementary Figure 12. Quantification of products. Each reaction was repeated three times to obtain the standard errors (n = 3). The bar graph with labels represents the mean value of reaction yield. Reaction yield were expressed as mean values ± standard error. (**a**) Product distribution of CO and CO_2. Reaction conditions are the same as those given in Supplementary Table 2, entries 1, 2, 10 and 20. (**b**) Product distribution of **1b** and HCl. The chloride concentration in the aqueous phase was determined by ion chromatography. Reaction conditions are the same as those given in Supplementary Table 2, entry 1, but with different amount of PVC (molar ratio of Cl to substrate range from 0.75 to 1.5). Note, HCl is volatile and some is lost during sample preparation (e.g. transfer, dilution).

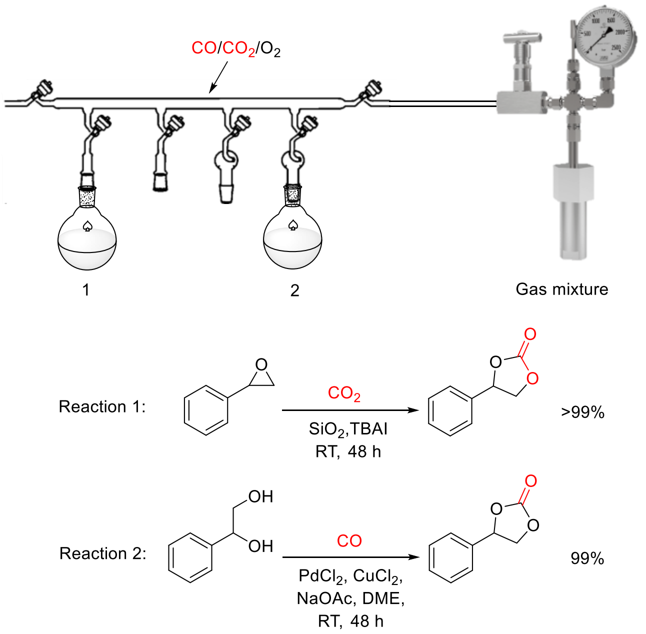

Supplementary Figure 13. Reaction of the CO/CO_2 containing gas produced. Reaction conditions: (**1**) Styrene oxide (2 mmol), SiO_2 (100 mg), tetrabutylammonium iodide (TBAI, 0.06 mmol), RT, 48 h. (**2**) 1-

Phenylethane-2,3-diol (0.2 mmol), $PdCl_2$ (0.02 mmol), $CuCl_2$ (0.4 mmol), NaOAc (0.04 mmol), dichloromethane (DME, 5 mL), RT, 48 h.[23] Yields were determined by GC.

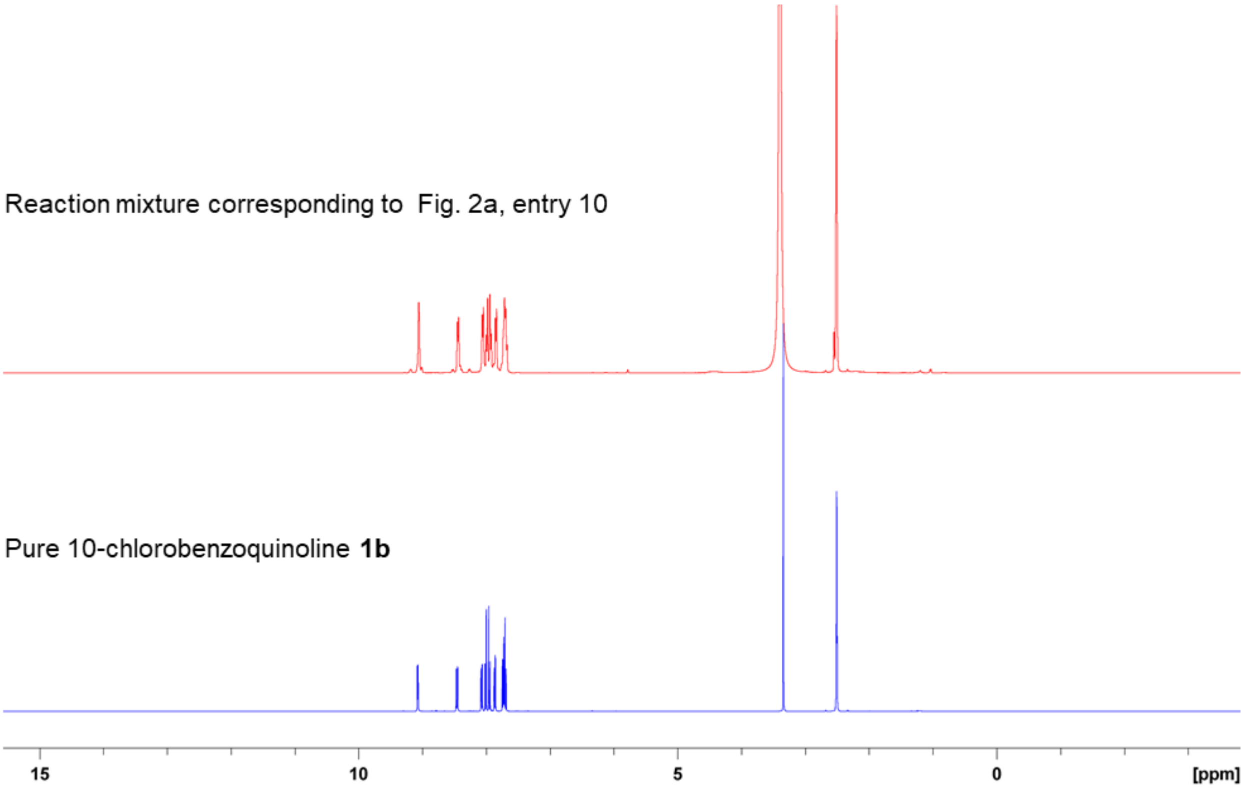

Supplementary Figure 14. Comparison of the ^{1}H NMR spectra of crude product **1b** and pure **1b**. Crude product **1b** is the unpurified reaction mixture corresponding to Fig. 2a, entry 7. Pure **1b** was obtained by purification using silica gel chromatography.

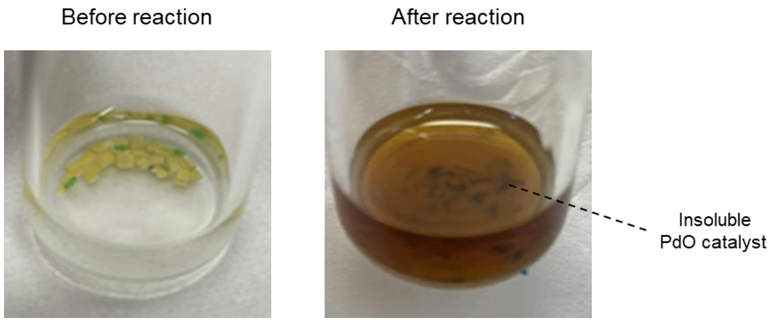

Supplementary Figure 15. Photographs showing the reaction using electric wire before and after reaction.

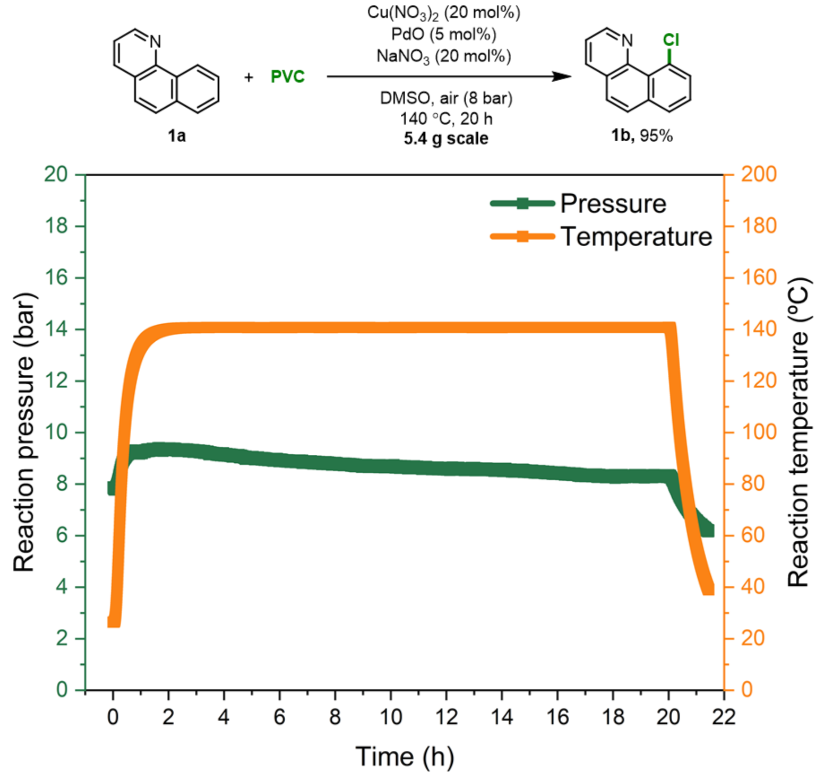

Supplementary Figure 16. Monitoring of the pressure and temperature for the reaction of **1a** on a 5.4 g scale.

Supplementary Table 3. Additive screening to demonstrate functional group compatibility.

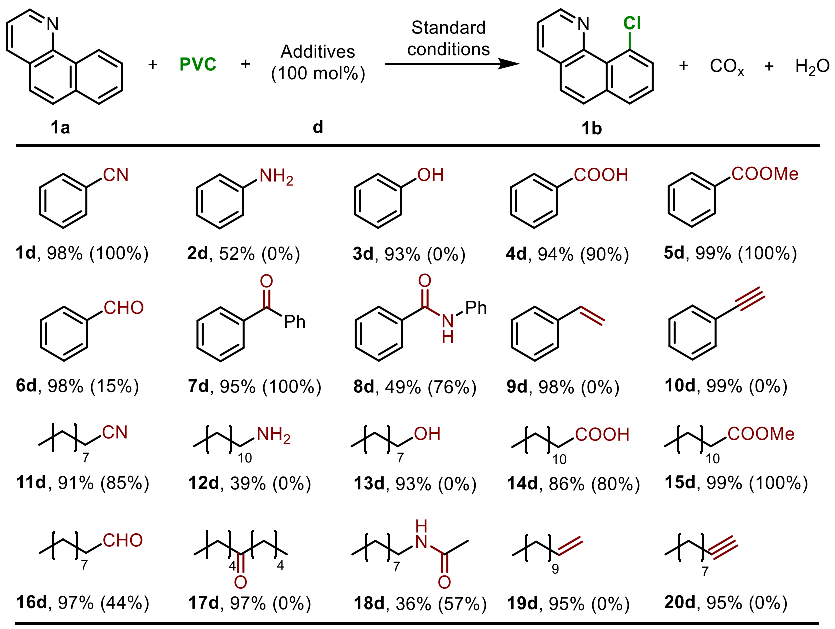

Reaction conditions are the same as those reported in Supplementary Table 2, entry 1. The remaining additive is given in parenthesis.

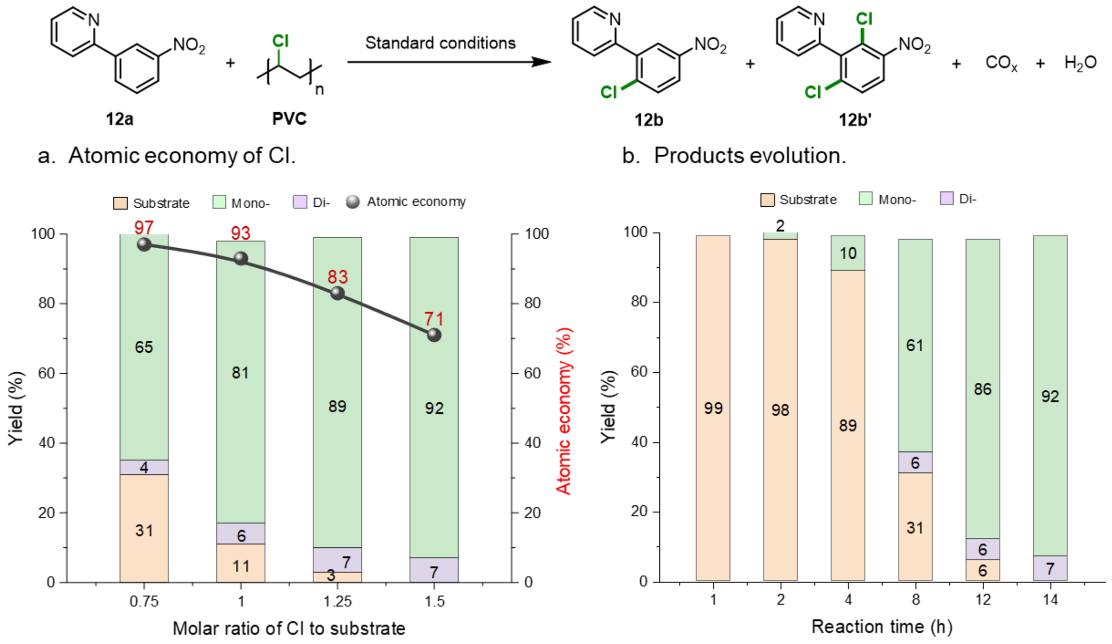

Supplementary Figure 17. Effect of PVC loading and Cl atom economy on yield and selectivity of *ortho*-di and monochloride products in the reaction between **12a** and PVC under the standard reaction conditions. (**a**) Cl atom economy and selectivity to the monochloride product **12b** with different PVC loadings (0.75 to 1.5 equiv. based on **12a**). Reactions were performed using the standard conditions: **12a** (0.25 mmol), PVC water pipe (0.75-1.5 equiv.), $Cu(NO_3)_2$•$3H_2O$ (20 mol%, 0.05 mmol), PdO (5 mol%, 0.0125 mmol), $NaNO_3$ (20 mol%, 0.05 mmol), DMSO (2 mL), O_2 (3 bar), 140 ºC, 15 h. (**b**) Evolution of the reaction with time. The bar graph with labels represents the mean value of reaction yield.

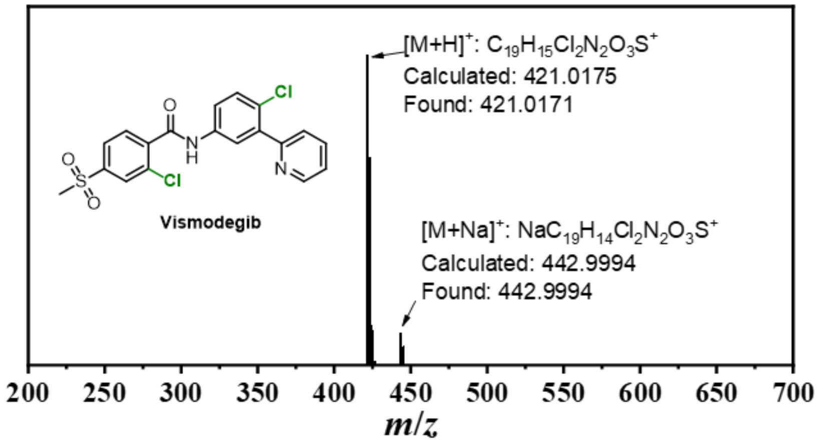

Supplementary Figure 18. Electrospray ionisation mass spectrum (ESI-MS) of vismodegib in THF (0.001 mmol/mL).

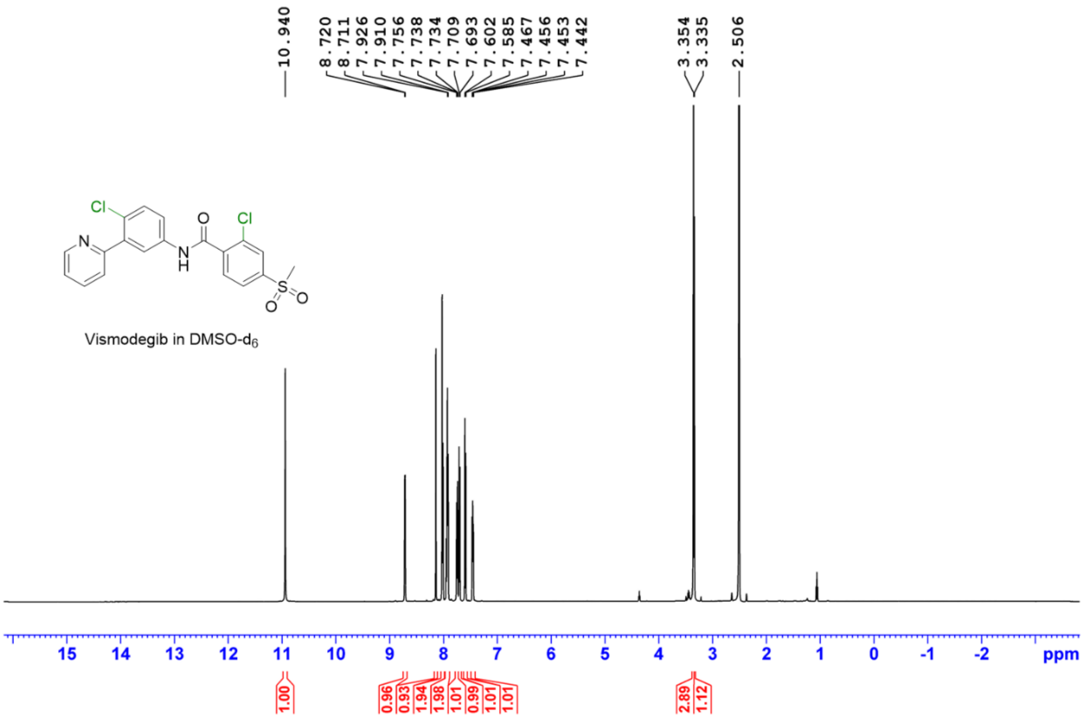

Supplementary Figure 19. ^{1}H NMR spectrum of vismodegib in DMSO-d_6.

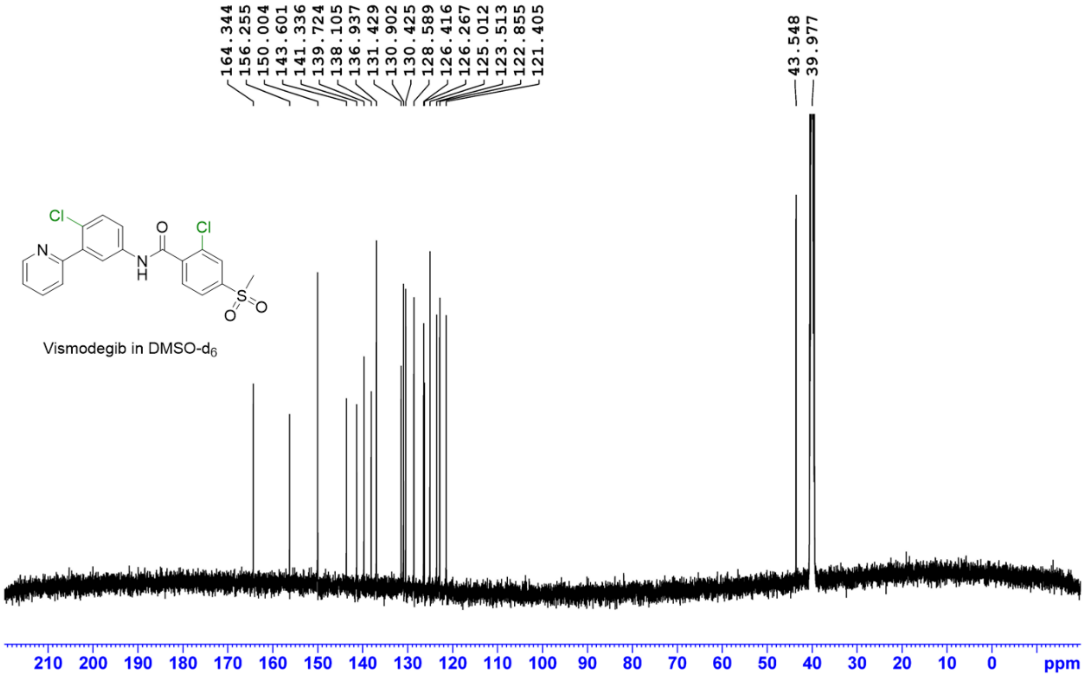

Supplementary Figure 20. ^{13}C NMR spectrum of vismodegib in DMSO-d_6.

Supplementary Table 4. Elemental analysis of vismodegib.

Elemental composition	Calculated (wt%)	Found-1 (wt%)	Found-2 (wt%)	Found-3 (wt%)
C	54.17	54.10	54.10	54.11
H	3.35	3.48	3.44	3.45
N	6.65	6.48	6.68	6.60
S	7.61	7.43	7.22	7.60

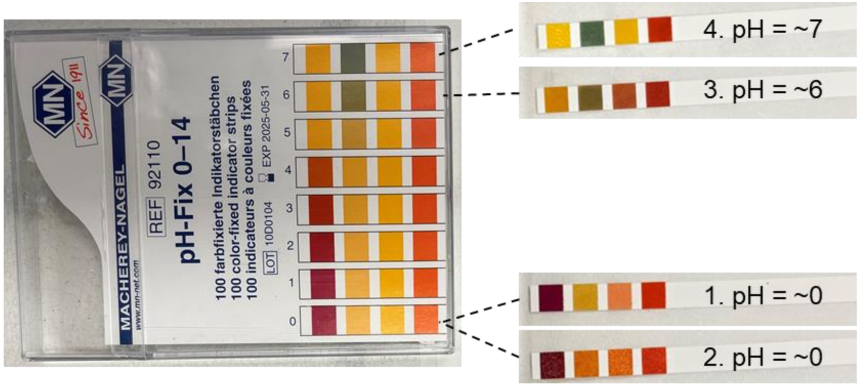

Reaction 1: PVC (2 mmol), THF (2 mL), O_2 (3 bar)

Reaction 2: Standard reaction conditions (Fig. 2a, entry 10)

Reaction 3: PVC (2 mmol), DMSO (2 mL), O_2 (3 bar)

Reaction 4: 1-phenylethyl chloride (**4c**) (2 mmol), DMSO (2 mL), O_2 (3 bar)

Supplementary Figure 21. pH tests of the reaction mixture. Reaction mixture was diluted with H_2O (1:1).

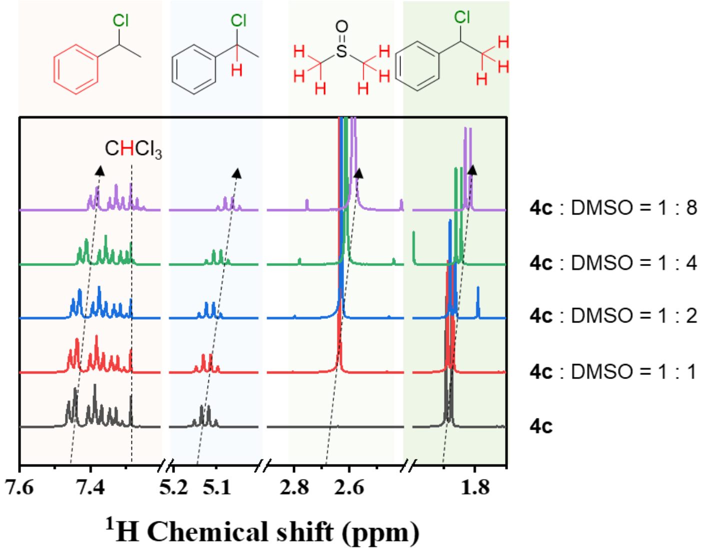

Supplementary Figure 22. ^{1}H NMR spectra of 1-phenylethyl chloride (**4c,** concentration: 1 mmol/mL) and DMSO (concentration: 1-8 mmol/mL) in $CDCl_3$. The peak at 7.26 ppm corresponding to the proton in $CHCl_3$

(present in the $CDCl_3$ solvent) does not change with the DMSO concentration and may be considered as an internal standard. As the concentration of DMSO increases the characteristic peaks of **4c** shift to lower frequencies, indicative of non-covalent interactions between the DMSO and **4c**, activating the C−Cl bond.[24]

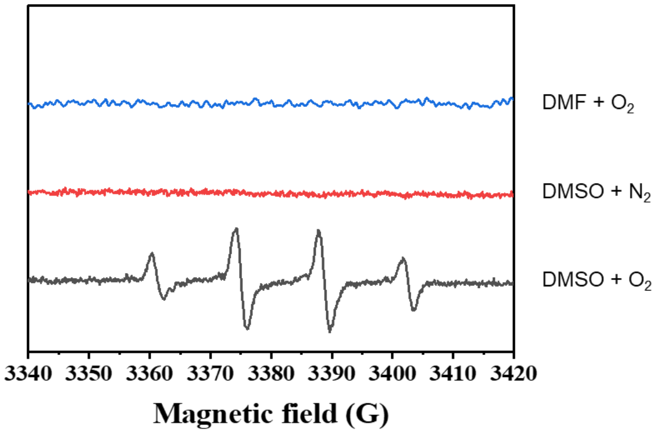

Supplementary Figure 23. Electron paramagnetic resonance (EPR) spectrum of the DMSO/O_2 promoted dehydrochlorination of **4c**. Reaction conditions: 1-phenylethyl chloride **4c** (0.5 mmol), DMSO (5 mL), O_2 or N_2, 140 °C, 0.5 h. The crude reaction mixture (200 μL) was mixed with 5,5-dimethyl-1-pyrroline N-oxide (DMPO, 3 M in DMSO, 200 μL) and an EPR spectrum was recorded at room temperature. An EPR signal (g_0= 2.007) corresponding to DMPO-$^{\bullet}$OH was identified from the reaction of O_2, DMSO and **4c**, confirming the formation of hydroxyl radicals during the reaction.

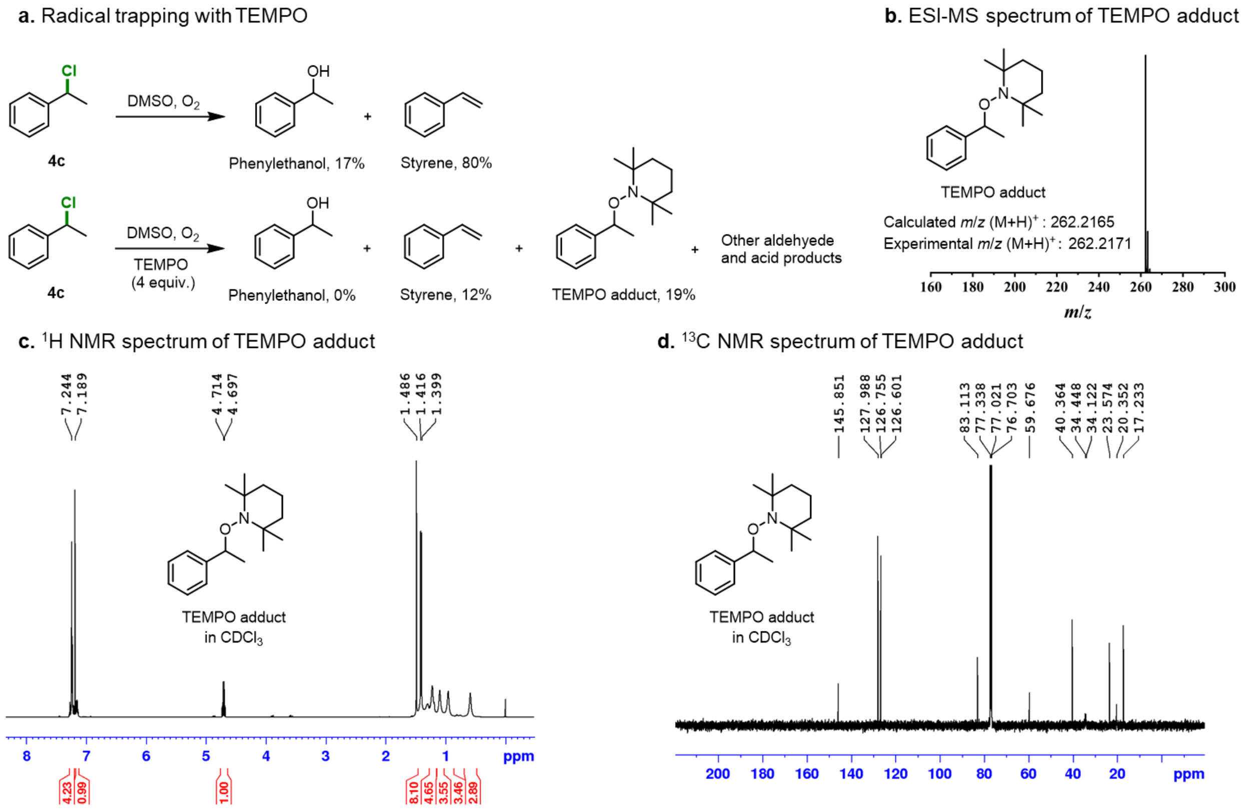

Supplementary Figure 24. Radical trapping experiments. Radical trapping with 4 equiv. 2,2,6,6-tetramethylpiperidin-1-yl)oxyl (TEMPO). Reaction conditions are the same those reported in Extended Data Fig. 2, entry 4. The TEMPO radical adduct was isolated by silica gel chromatography (hexane was used as the mobile phase). (**b**) ESI-MS of the TEMPO radical adduct. (**c**) ^{1}H and (**d**) ^{13}C NMR spectra of the TEMPO radical adduct.

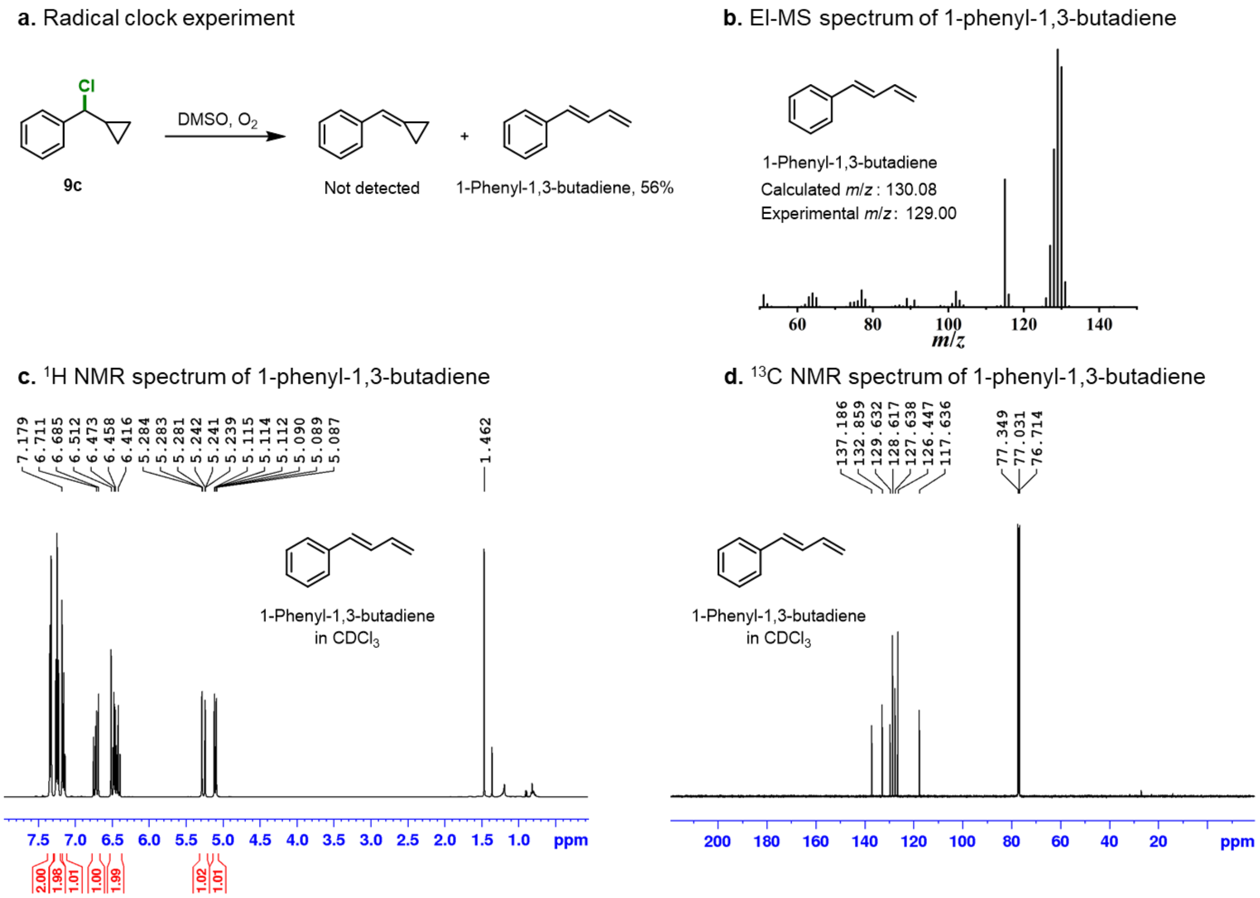

Supplementary Figure 25. Radical clock experiment. (**a**) Radical clock experiment employing (chloro(cyclopropyl)methyl)benzene **9c** as substrate. Reaction conditions are the same as those reported in Extended Data Fig. 2, entry 4. 1-Phenyl-1,3-butadiene was isolated as the main product by silica gel chromatography (hexane was used as the mobile phase). (**b**) EI-MS of 1-phenyl-1,3-butadiene. (**c**) ^{1}H (**d**) ^{13}C NMR spectra of 1-phenyl-1,3-butadiene.

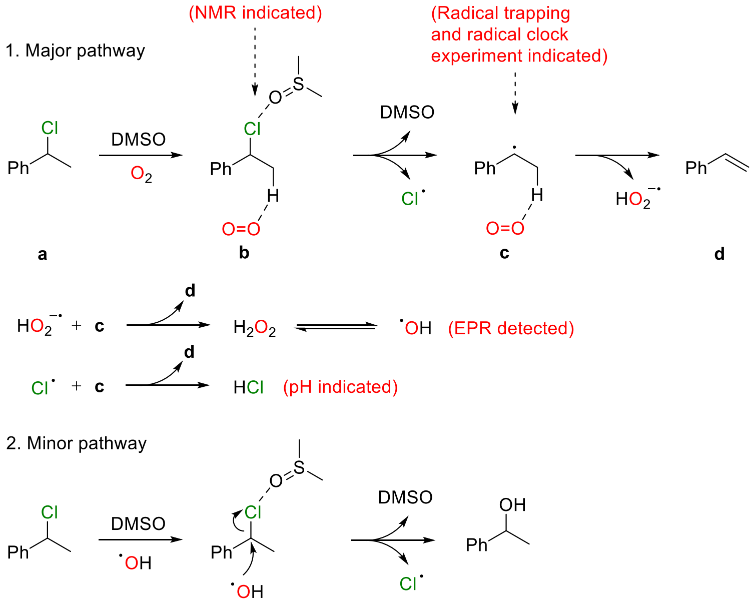

Supplementary Figure 26. Proposed pathways for the DMSO/O_2 catalyzed dechlorination reaction (**a** to **d**). 1,2-Elimination is the major pathway for the thermal dehydrochlorination (>300 ºC) of PVC to produce HCl and polyenes.[10, 13, 17, 19, 25-26] In the reaction reported here, DMSO[27] and O_2[25-26, 28] act as electrophilic Cl^- and nucleophilic H^+ adsorbents, respectively. DMSO is expected to activate the chloride atom in **a** (confirmed by NMR spectroscopy, see Supplementary Figure 22) and O_2 may simultaneously adsorb the β-H, to generate styrene and a $Cl^•$ radical (Major pathway),[25-26] evidenced by the dehydrochlorination product, styrene, not being observed in the absence of O_2 or DMSO (Extended Data Fig. 2, entries 5-7). In addition, O_2 converts into a superoxide radical and the generation of highly active $Cl^•$ radicals and superoxide radicals propagate the continuous dehydrochlorination of **a**, giving HCl (indicated by a decrease in pH, see Supplementary Figure 21) and (hydroxyl radicals, indicated by EPR spectroscopy, see Supplementary Figure 23). A dechloro-hydroxylation product, 1-phenylethanol, was also detected in low yield irrespective of whether the reaction was performed in the absence or presence of O_2 (Extended Data Fig. 2, entries 3-5). A tentative mechanism involving nucleophilic attack of hydroxyl radicals is shown (Minor pathway).[29-31]

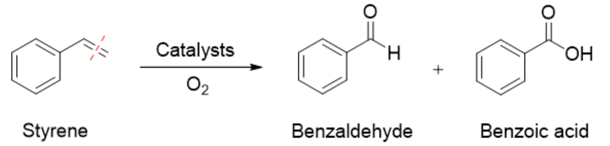

Entry	Catalyst	Benzaldehyde (%)	Benzoic acid (%)
1		0	0
2	20 mol% $NaNO_3$	5	93
3	20 mol% CuO	8	4
4	20 mol% $Cu(OAc)_2$	3	0
5	5 mol% PdO	14	0

Supplementary Figure 27. Testing the activity of Cu, Pd and $NaNO_3$ in C=C bond activation. Reaction conditions: styrene (0.25 mmol), catalyst, biphenyl (0.2 mmol, internal standard), DMSO (2 mL), O_2 (3 bar), 140 ºC, 4 h. $NaNO_3$ is the most efficient catalyst for C=C bond activation.

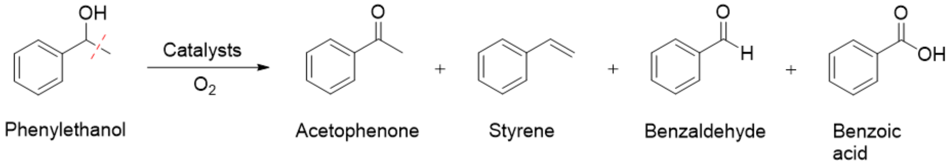

Entry	Catalyst	Acetophenone (%)	Styrene (%)	Benzaldehyde (%)	Benzoic acid (%)
1		2	1	0	0
2	20 mol% $NaNO_3$	1	0	5	90
3	20 mol% CuO	10	0	0	0
4	20 mol% $Cu(OAc)_2$	7	0	0	0
5	5 mol% PdO	90	2	2	3

Supplementary Figure 28. Testing the activity of Cu, Pd and $NaNO_3$ in C(OH)−C bond activation. Reaction conditions: 1-phenylethanol (0.25 mmol), catalyst, biphenyl (0.2 mmol, internal standard), DMSO (2 mL), O_2 (3 bar), 140 ºC, 4 h. PdO promotes the oxidation of 1-phenylethanol to acetophenone.[32-35] However, $NaNO_3$ was found to be the most efficient catalyst for C(OH)−C bond activation, as reported previously.[36] The mechanism of $NaNO_3$ catalyzed C=C/C(OH)−C bond activation is described below.

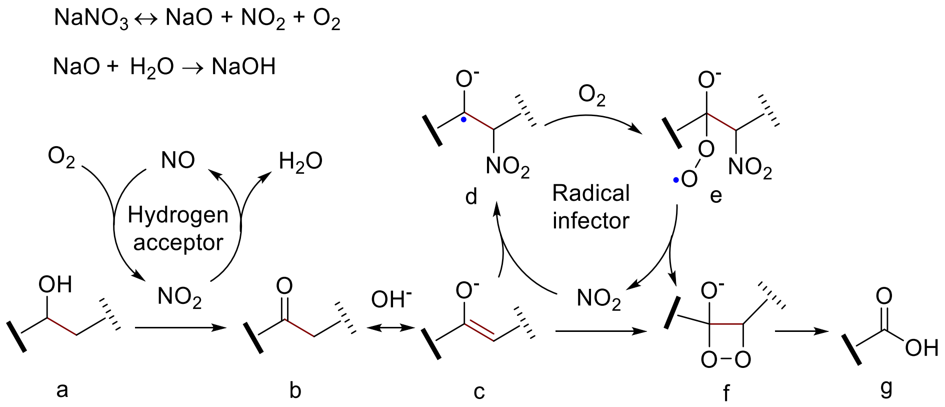

Supplementary Figure 29. Tentative reaction pathway for $NaNO_3$ catalyzed C=C/C(OH)−C bond activation of the alkene and alkanol intermediates. $NaNO_3$ catalyzed C=C/C(OH)−C bond activation was reported in previously.[36] Decomposition of $NaNO_3$ provides NO_2 gas and ^{-}OH that catalyze the cleavage of C=C/C(OH)−C bonds. Specifically, for the activation of C(OH)−C bond, NO_2 acts as hydrogen acceptor promoting the conversion of alcohol **a** to carbonyl **b**. ^{-}OH promotes the tautomerization of **b** to enol **c**. The addition of a NO_2 radical to enol **c** generates corresponding carbon centered radical **d**. Radical **d** may capture O_2 resulting in the formation of peroxyl radical intermediate **e**. Following the release of NO_2 radical from **e**, dioxetane intermediate **f** is generated, which undergoes thermal cleavage yielding the carboxylic acid product **g**. The reaction pathway of $NaNO_3$ catalyzed C=C bond activation is similar to the transformation of enol **c** to the carboxylic acid product **g**. Using chlorinated waste, alkene (dominant) and alkanol (minor) intermediates are formed, which can transfer into the carboxylic acid product (i.e. formic acid and other alkyl carboxylic acids) after the $NaNO_3$ catalyzed cleavage of C=C/C(OH)−C bonds. Formic acid can decompose into CO_x gas and H_2O, leading to the total mineralization of the hydrocarbon component.

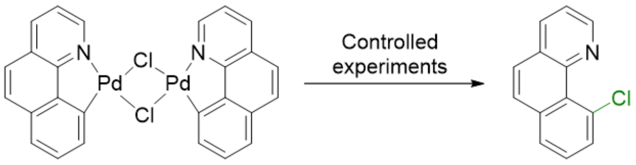

1b

Entry	Reaction condition	Yield of **1b** (%)
1	O_2	0
2	$NaNO_3 + O_2$	33
3	$CuCl_2 + O_2$	0
4	$CuCl_2 + NaNO_3 + O_2$	99

Supplementary Figure 30. Chlorinated product **1b** generated from model benzo[h]quinolinyl Pd(II) chloride intermediate. Reaction conditions: benzo[h]quinolinyl Pd(II) chloride (0.1 mmol), $CuCl_2$ (20 mol%, 0.02 mmol), $NaNO_3$ (20 mol%, 0.02 mmol), biphenyl (0.1 mmol), DMSO (2 mL), O_2 (3 bar), 140 ºC, 1 h. Only with the combination of the $CuCl_2$ and $NaNO_3$ catalysts, benzo[h]quinolinyl Pd(II) chloride affords the chlorinated product **1b** in near-quantitative yield.

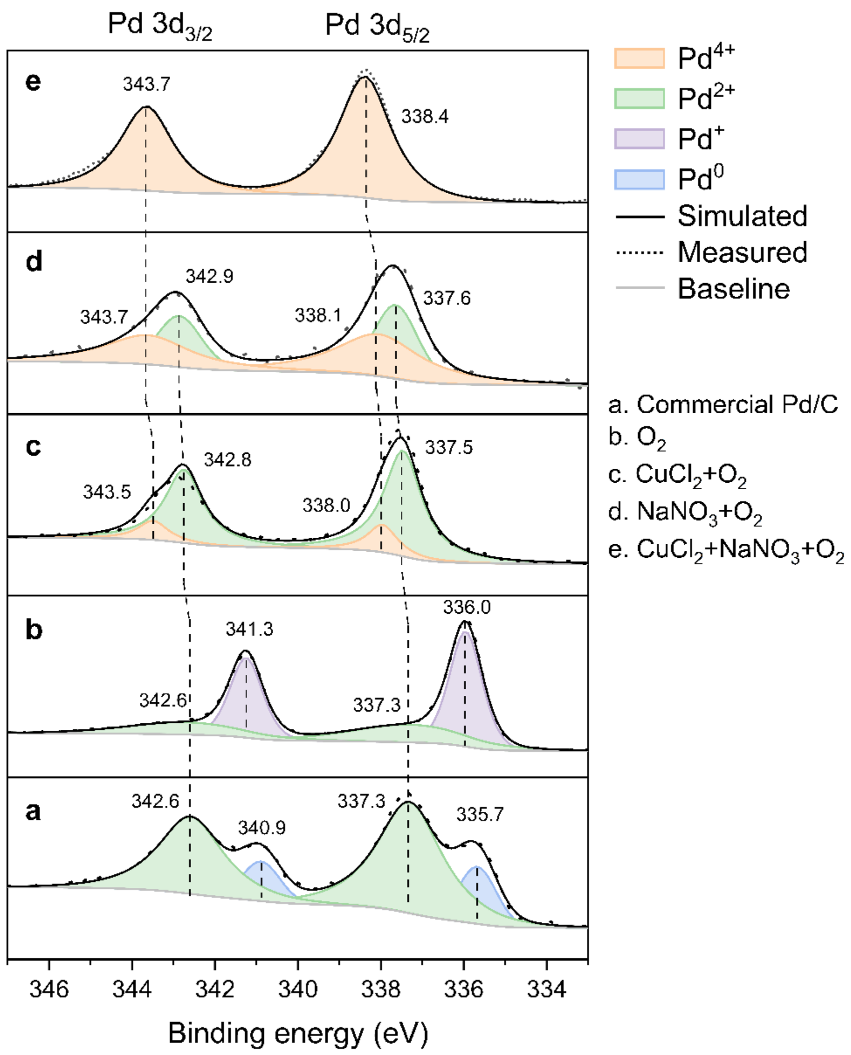

Supplementary Figure 31. X-ray photoelectron spectroscopy (XPS) of the Pd/C catalyst under different reaction conditions in the absence of a substrate. In order to monitor the evolution of the surface species, 10% Pd/C catalyst was used instead of PdO (Pd/C from Sigma-Aldrich has the same catalytic activity as PdO, see Supplementary Table 2, entry 14). After reaction, the Pd/C catalyst was purified by washing with water (3×10 mL), acetone (3×10 mL), ethyl acetate (3×10 mL) and diethyl ether (3×10 mL) and drying under vacuum (30 ºC, 18 h) prior to XPS. (**a**) XPS of commercial 10% Pd/C under anaerobic conditions, peaks with binding energy values E_b (Pd3$d_{5/2}$ and Pd3$d_{5/2}$) of 335.7 and 340.9 eV correspond to Pd^0 species and peaks with E_b (Pd3$d_{5/2}$ and Pd3$d_{5/2}$) of 337.3 and 342.6 eV correspond to Pd^{2+} species, indicating that the Pd/C catalyst is partially oxidized with PdO surface species. (**b**) XPS of the Pd/C catalyst after treatment under aerobic reactions (10% Pd/C (0.0125 mmol), DMSO (2 mL), O_2 (3 bar), 140 ºC, 15 h), peaks corresponding to Pd^0 and Pd^{2+} species nearly disappear and new peaks with E_b (Pd3$d_{5/2}$ and Pd3$d_{5/2}$) at 336.0 and 341.3 eV appear, which may be attributed to Pd^+ species. Thus, the surface species on the Pd/C catalyst undergo synproportionation in O_2 rather than further oxidation. (**c**) XPS of the Pd/C catalyst after treatment under Cu catalyzed aerobic reaction conditions ($Cu(NO_3)_2$•$3H_2O$ (0.05 mmol), 10% Pd/C (0.0125 mmol), DMSO (2 mL), O_2 (3 bar), 140 ºC, 15 h). Peaks with E_b (Pd3$d_{5/2}$ and Pd3$d_{5/2}$) at 337.5 and 342.8 eV correspond to Pd^{2+} species. The new component with highest E_b (Pd3$d_{5/2}$ and Pd3$d_{5/2}$) at 338.0 and 343.5 eV may be assigned to Pd^{4+} species. (**d**) XPS of the Pd/C catalyst following treatment under $NaNO_3$ catalyzed aerobic reaction conditions ($NaNO_3$ (0.05 mmol), 10% Pd/C (0.0125 mmol), DMSO (2 mL), O_2 (3 bar), 140 ºC, 15 h). Peaks with E_b (Pd3$d_{5/2}$ and Pd3$d_{5/2}$) at 337.6 and 342.9 eV correspond to Pd^{2+} species.

The new component with highest E_b (Pd3d$_{5/2}$ and Pd3d$_{5/2}$) at 338.1 and 343.7 eV corresponds to Pd^{4+} species. These results suggest that $CuCl_2$ or $NaNO_3$ act as redox mediator,[37] with O_2 acting as the final oxidant to promote the full oxidation of Pd^0 to Pd^{2+} species and partial oxidation of Pd^{2+} to Pd^{4+} species. (**e**) XPS of the Pd/C catalyst following treatment under aerobic reaction conditions in the presence of both $CuCl_2$ and $NaNO_3$ ($Cu(NO_3)_2$•$3H_2O$ (0.05 mmol), 10% Pd/C (0.0125 mmol), $NaNO_3$ (0.05 mmol), DMSO (2 mL), O_2 (3 bar), 140 ºC, 15 h). Peaks indicative of Pd^0 and Pd^{2+} species are not observed. The only component with highest E_b (Pd3d$_{5/2}$ and Pd3d$_{5/2}$) at 338.4 and 343.7 eV may be assigned Pd^{4+} species, indicating that using both $CuCl_2$ and $NaNO_3$ as redox mediators, oxidation of Pd^0 and Pd^{2+} species to Pd^{4+} is favored. Pd(IV) species formed under the standard reaction conditions are consequently expected to be the key intermediates that afford C−Cl bonds via reductive elimination.[38-39] These results agree with the observation that the chlorination reaction cannot proceed without either Cu or $NaNO_3$ catalyst (Extended Data Fig. 3).

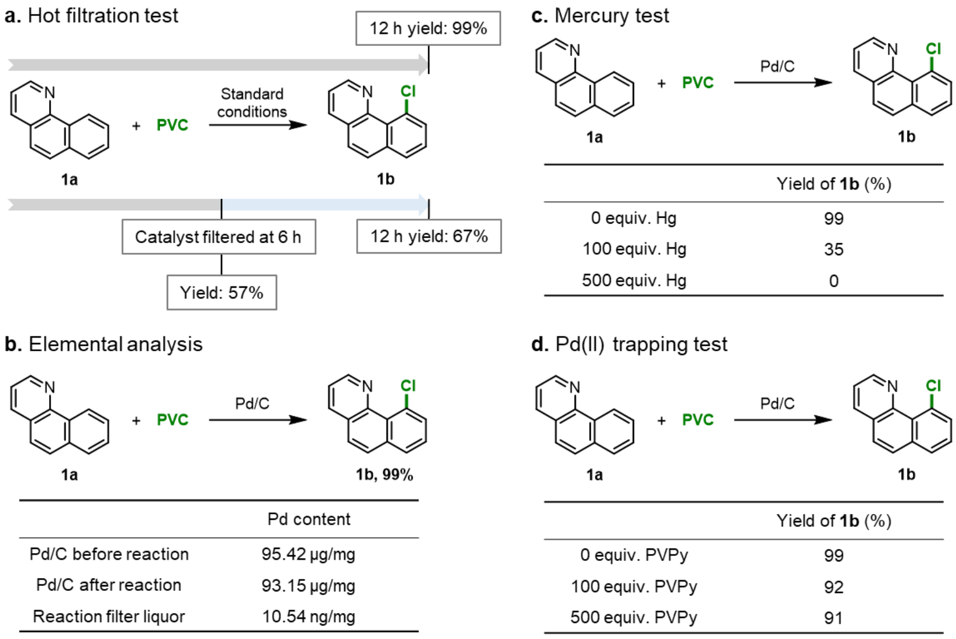

Supplementary Figure 32. Experiments to probe the nature of the active PdO catalyst. (**a**) Hot filtration test under the standard reaction conditions.[40] The hot reaction mixture was filtered to remove all heterogenous PdO catalyst after 6 h reaction, and the reaction was continued for a further 6 h, no further reaction was observed, suggesting all active heterogenous species were removed by filtration. This observation is consistent with the homogeneous Pd catalysts studied being less active than the heterogenous Pd catalysts studied (see Supplementary Figure 2 and Supplementary Table 2). (**b**) Elemental analysis of the heterogenous Pd/C catalyst and liquid reaction mixture. In order to determine if the heterogenous Pd catalyst leaches, Pd/C catalyst was used (Pd/C transforms into PdO/C under the reaction conditions and shows the same catalytic activity as PdO, see Supplementary Table 2). Inductively Coupled Plasma (ICP) mass spectrometry was used to determine the Pd content of the reaction mixture before and after reaction, as well as the filtered solution after reaction, suggesting very little leaching of Pd takes place. (**c**) Mercury poisoning test. Pd/C was used instead of PdO. Mercury acts as a selective poison for nanoparticle catalysts to form a catalytically inactive Hg amalgam. Addition of Hg was found to inhibit the reaction, suggesting that the catalyst is not homogeneous.[41] (**d**) Pd(II) trapping test using insoluble poly(4-vinylpyridine) (PVPy).[42] The pyridine ligands in PVPy strongly coordinates to homogeneous Pd species, trapping them within the insoluble polymer. Since PVPy does not suppress the reaction it suggests that homogeneous Pd species are not the active catalyst.[42]

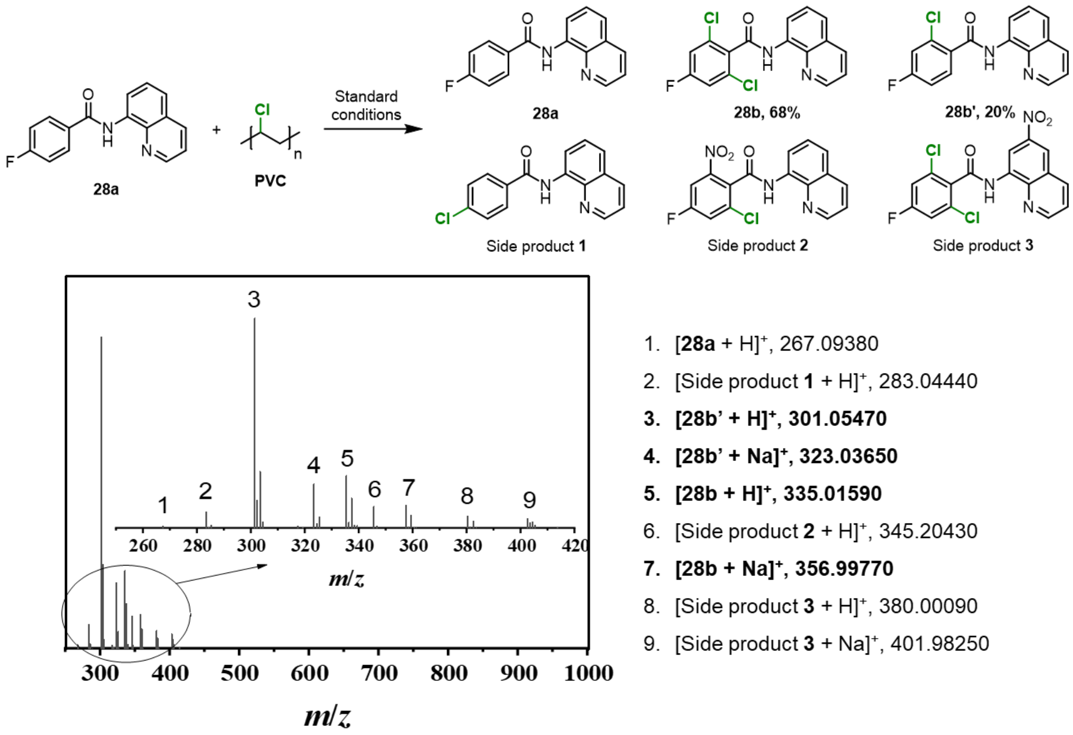

Supplementary Figure 33. Electrospray ionization mass spectrometry (ESI-MS) of the reaction mixture after filtration. In order to obtain strong mass signals **28a** was used as the substrate. Heterogeneous PdO was filtered and the DMSO reaction mixture solution was diluted with THF (DMSO : THF, 1 : 20, v/v). Pd species were not detected. Peak assignments are listed and correspond to the substrate and products.

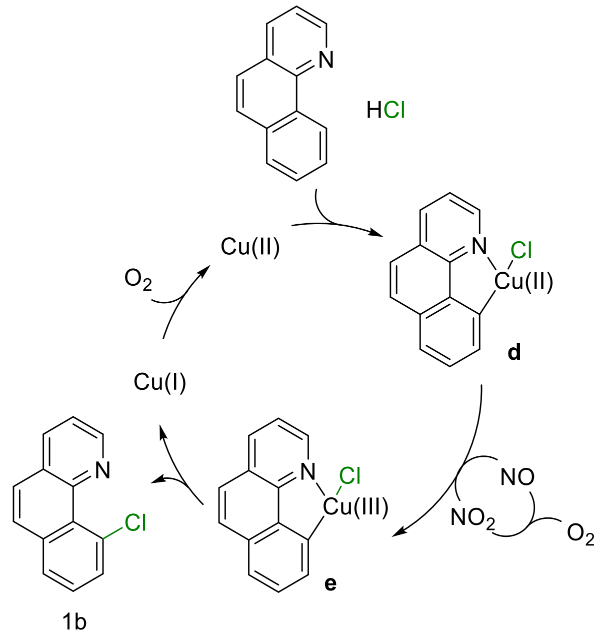

Supplementary Figure 34. Proposed reaction pathway for Cu catalyzed C−H chlorination with $NaNO_3$ promoter. Without Pd catalyst, Cu also can catalyze C−H chlorination with $NaNO_3$ as promoter, but less efficiently (Extended Data Fig. 3, entry 5). The Cu catalyst also will activate C−H bond of substrate **1a** to generate the Cu(II) complex **d**.[43-45] After oxidation with NO_2 (derived from the decomposition of $NaNO_3$), the Cu(III) complex **c** is formed. Although less favorable, Cu(III) complex **c** can also undergo reductive elimination to afford product **1b**.[45]

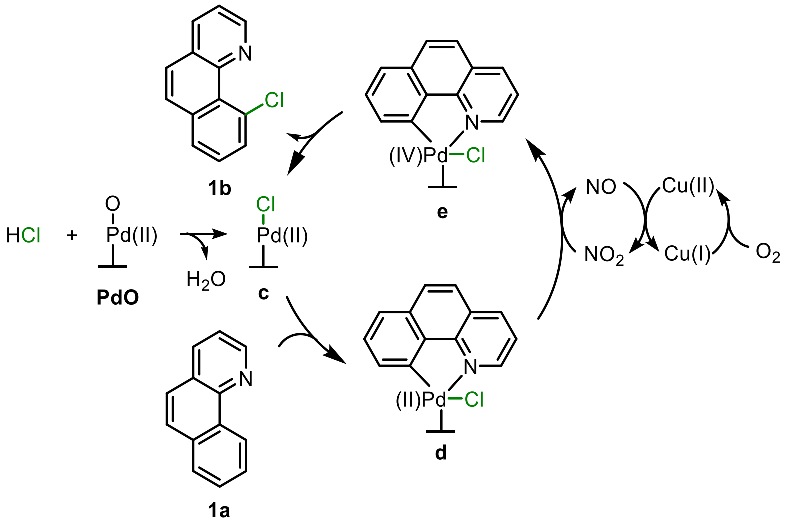

Supplementary Figure 35. Proposed reaction pathway for PdO catalyzed C−H chlorination with Cu and $NaNO_3$ promotor. Using HCl as chlorination reagent, PdO, $Cu(NO_3)_2$, $NaNO_3$ and O_2 are indispensable for the chlorination of 7,8-benzoquinoline **1a** to 10-chlorobenzoquinoline **1b**. A bimetallic pathway might be in operation as reported elsewhere for catalytic C–C or C–X bond formation.[46-47] Ion exchange of HCl with PdO generates the Pd(II) intermediate **c**.[48] The Pd catalyst **c** will activate C−H bond of substrate **1a** to generate the Pd(II) complex **d**.[43-45] After oxidation with Cu and NO_2 (derived from the decomposition of $NaNO_3$), the Pd(IV) complex **e** is formed.[45] Reductive elimination of Pd(IV) complex **e** gives product **1b**.[45]

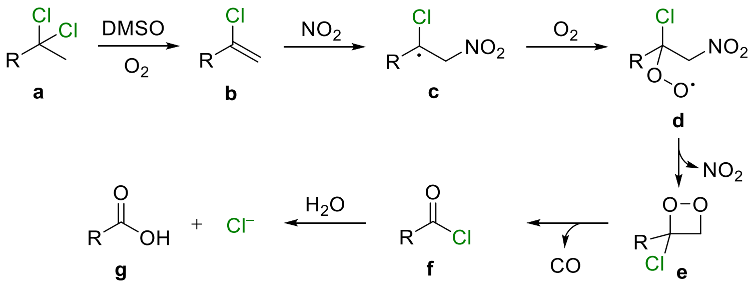

Supplementary Figure 36. Proposed pathway for the dechlorination and depolymerization of PVDC-type model compounds, i.e. containing two Cl atoms at the α-position. The Cl atoms undergo DMSO/O_2 catalyzed dechlorination to afford alkene intermediate **b** (see Supplementary Figure 26 for further details), followed by $NaNO_3$ catalyzed C=C bond cleavage and depolymerization to generate CO and acyl chloride **f** (Supplementary Figure 29). Hydrolysis of acyl chloride **f** affords Cl^- and carboxylic acid **g**.

Life cycle assessment (LCA)

Methods

The Life Cycle Assessment (LCA) approach implemented is a gate-to-gate analysis exclusively focused on the manufacturing phase of aryl chlorides. The production process for each scenario is synthetically represented in Supplementary Figs. 37-40. Analysis starts from the acquisition of raw materials, encompassing the synthetic process of aryl chlorides and it ends with waste disposal. LCA using PVC as chlorination reagent (scenario 1) is compared with a typical chlorination method using N-chlorosuccinimide (NCS) as chlorination reagent (scenario 2),[49] affording 2-(2-chloro-5-nitrophenyl)pyridine **12b**, an intermediate in the synthesis of the anticancer drug vismodegib. LCA of this work (scenario 3) is also compared with electrochemical method using PVC as chlorination reagent,[50] for the generation of 1-(2-chlorophenyl)-pyrazole **19b** (scenario 4)**.** LCA was performed using OpenLCA 1.11, with the Ecoinvent v3.8 database.[51] The scope of the LCA is to compare the 100-year global warming potential (GWP 100a) for the production of aryl chlorides of using PVC waste as chlorination reagent with other chlorination methods. Impact assessment is based on IPCC 2013 GWP 100a.[50, 52] Reaction details and LCA results are summarized in Supplementary Tables S5-7.

Assumptions

1. All scenarios are scaled to afford 1 kg of product.
2. In all scenarios, reactions are performed in sealed batch reactors and the energy consumption of each scenario was assumed to be constant.
3. In scenario 1 the equivalent PVC is considered to be incinerated, i.e. if the PVC is not used as a chlorination reagent.
4. In scenario 3 unreacted PVC is incinerated and the impact of DEHP is not included.
5. In scenarios 2 and 4 the production of valuable CO is not included.
6. Repeated reagents, solvents, energy consumption during product isolation, equipment required and waste effluent treatment and disposal, are deemed constant and are therefore not included.

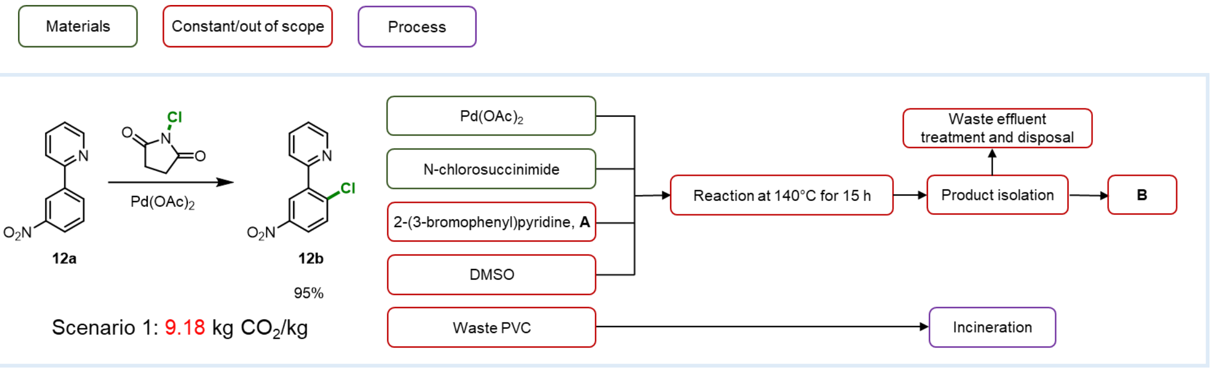

Supplementary Figure 37. LCA diagram for scenario 1.

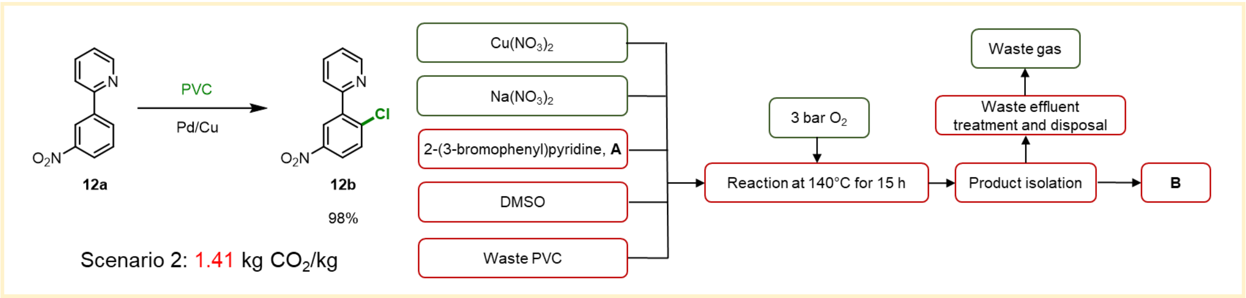

Supplementary Figure 38. LCA diagram for scenario 2.

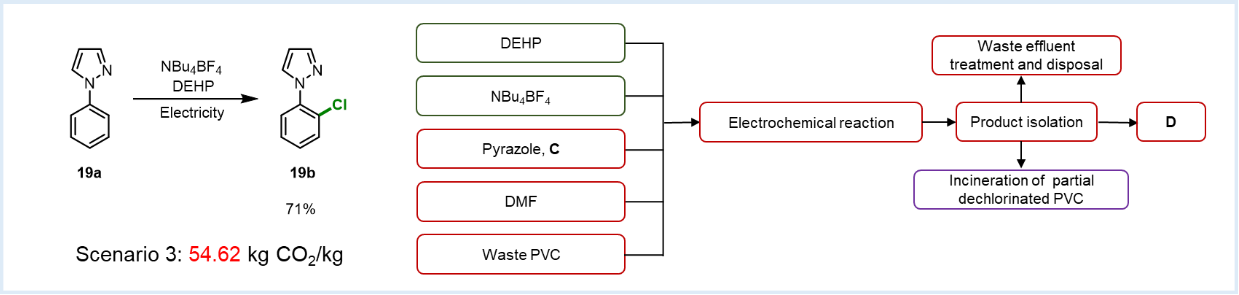

Supplementary Figure 39. LCA diagram for scenario 3.

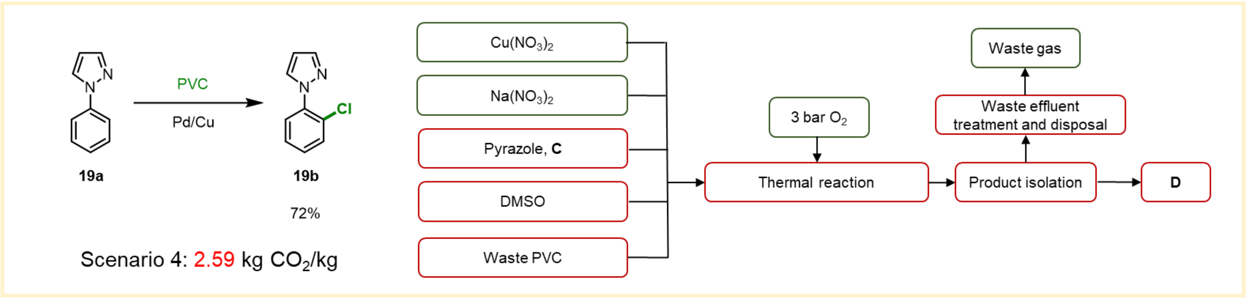

Supplementary Figure 40. LCA diagram for scenario 4.

Supplementary Table 5. Reaction details of scenarios 1-4.

Scenario	Substrate	Product	Materials	Yield (%)	Source
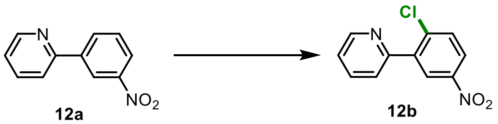 12a → 12b					
1	**12a** (0.90 kg)	**12b** (1.00 kg)	Pd(OAc)$_2$ (5 mol%, 0.05 kg), N-chlorosuccinimide (1.5 equiv., 0.90 kg)	95	Ref. 7
2	**12a** (0.87 kg)	**12b** (1.00 kg)	PVC water pipe (1.5 equiv., 0.27 kg), Cu(NO$_3$)$_2$•3H$_2$O (20 mol%, 0.21 kg), PdO (5 mol%, 0.03 kg), NaNO$_3$ (20 mol%, 0.07 kg)	98	This work
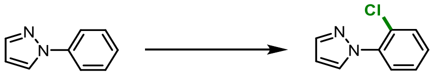 19a → 19b					
3	**19a** (1.14 kg)	**19b** (1.00 kg)	PVC (~8 equiv., 3.95 kg), NBu$_4$BF$_4$ (~7.3 equiv., 5.15 kg), DEHP (1 equiv., 3.04 kg)	71	Ref. 8
4	**19a** (1.12 kg)	**19b** (1.00 kg)	PVC water pipe (1.5 equiv., 0.73 kg), Cu(NO$_3$)$_2$•3H$_2$O (20 mol%, 0.37 kg), PdO (5 mol%, 0.05 kg), NaNO$_3$ (20 mol%, 0.13 kg)	72	This work

Supplementary Table 6. LCA results for scenarios 1-2.

**Item**	**Impact (kg CO_2 per eq/kg)**	**Scenario 1**		**Scenario 2**	
		Amounts (kg)	*Impact (kg CO_2 per eq/kg)*	*Amounts (kg)*	*Impact (kg CO_2 per eq/kg)*
Materials production					
N-chlorosuccinimide	4.59	0.90	4.13	0	0
Palladium(II) acetate	92.97	0.05	4.65	0	0
Copper(II) nitrate	4.50	0	0	0.21	0.95
Sodium nitrate	0.52	0	0	0.07	0.04
Oxygen	1.07	0	0	0.02	0.02
Distribution and use life/end of life					
PVC incineration	1.47	0.27	0.40	0	0
CO_2 from reaction	1	0	0	0.40	0.40

**LCA–GWP (kg CO_2 per eq/kg)**	**9.18**	**1.41**

Supplementary Table 7. LCA results for scenarios 3-4.

Item	Impact (kg CO_2 per eq/kg)	Scenario 3		Scenario 4	
		Amounts (kg)	*Impact (kg CO_2 per eq/kg)*	*Amounts (kg)*	*Impact (kg CO_2 per eq/kg)*
Materials production					
DEHP	802.39	0	0	0	0
NBu_4BF_4	9.48	5.15	48.82	0	0
Copper(II) nitrate	4.50	0	0	0.37	1.67
Sodium nitrate	0.52	0	0	0.14	0.07
Oxygen	1.07	0	0	0.02	0.02
Distribution and use life/end of life					
PVC incineration	1.47	3.95	5.80	0	0
CO_2 from reaction	1	0	0	0.83	0.83
**LCA–GWP (kg CO_2 per eq/kg)**		**54.62**		**2.59**	

Supplementary Information

NMR spectra

Substrate

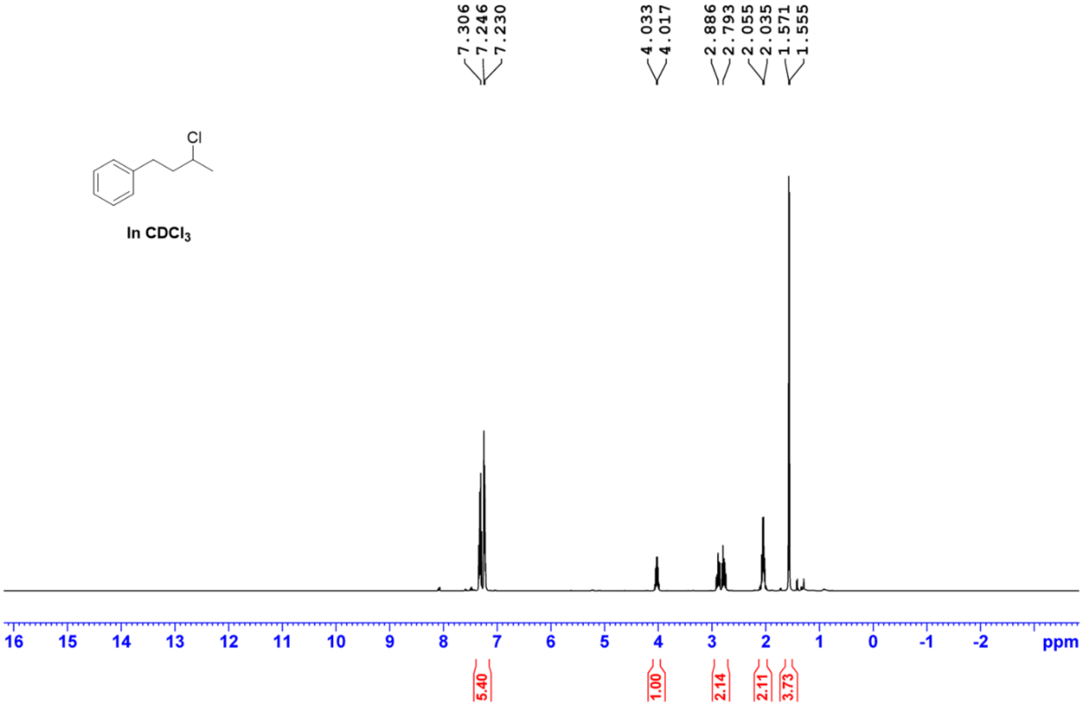

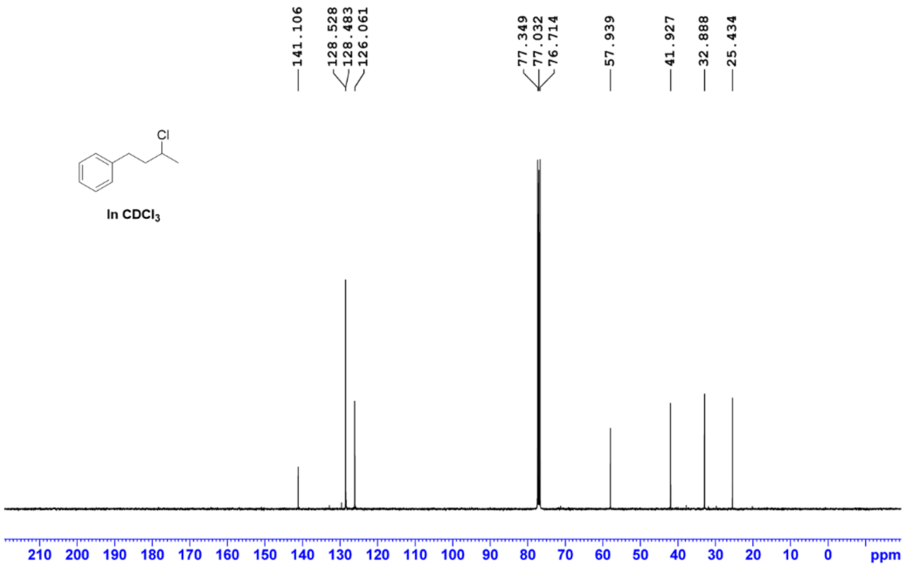

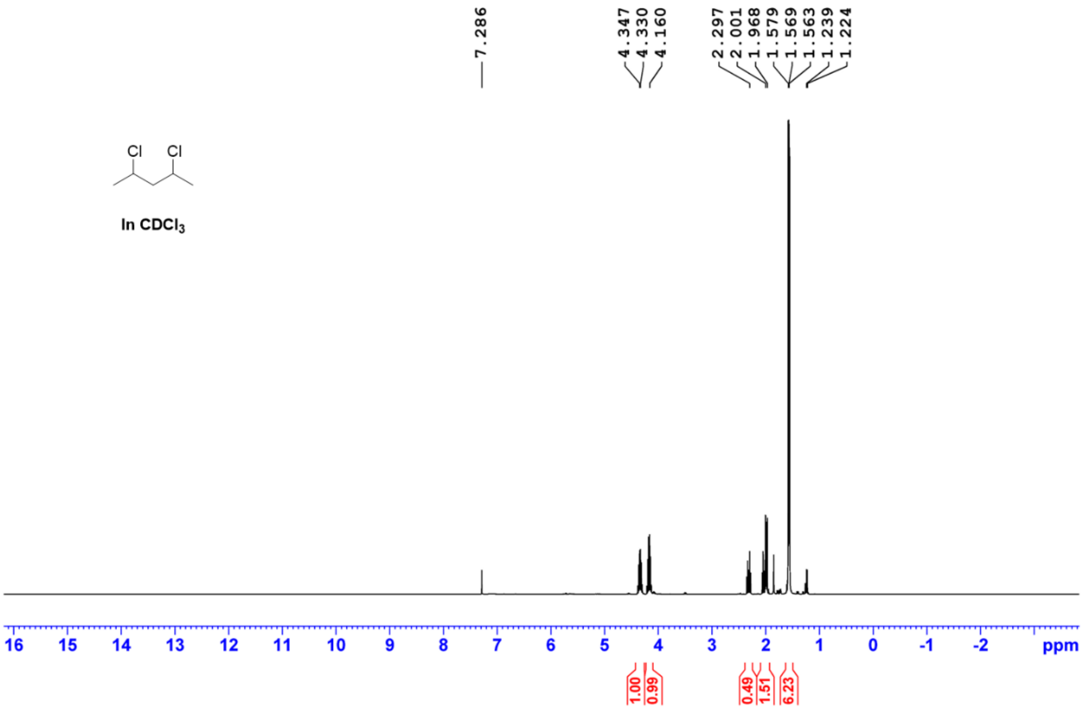
7.286
4.347
4.330
4.160
2.297
2.001
1.968
1.579
1.569
1.563
1.239
1.224
Cl
Cl
In CDCl3
16
15
14
13
12
11
10
9
8
7
6
5
4
3
2
1
0
-1
-2
ppm
1.00
0.99
0.49
1.51
6.23

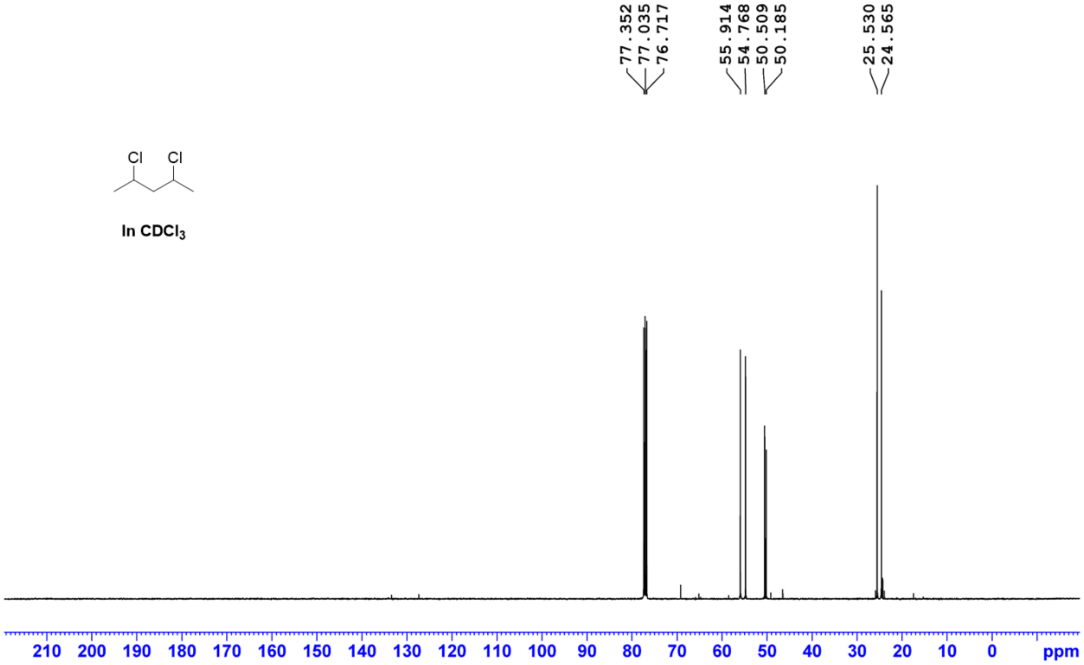
77.352
77.035
76.717
55.914
54.768
50.509
50.185
25.530
24.565
Cl
Cl
In CDCl3
210
200
190
180
170
160
150
140
130
120
110
100
90
80
70
60
50
40
30
20
10
0
ppm

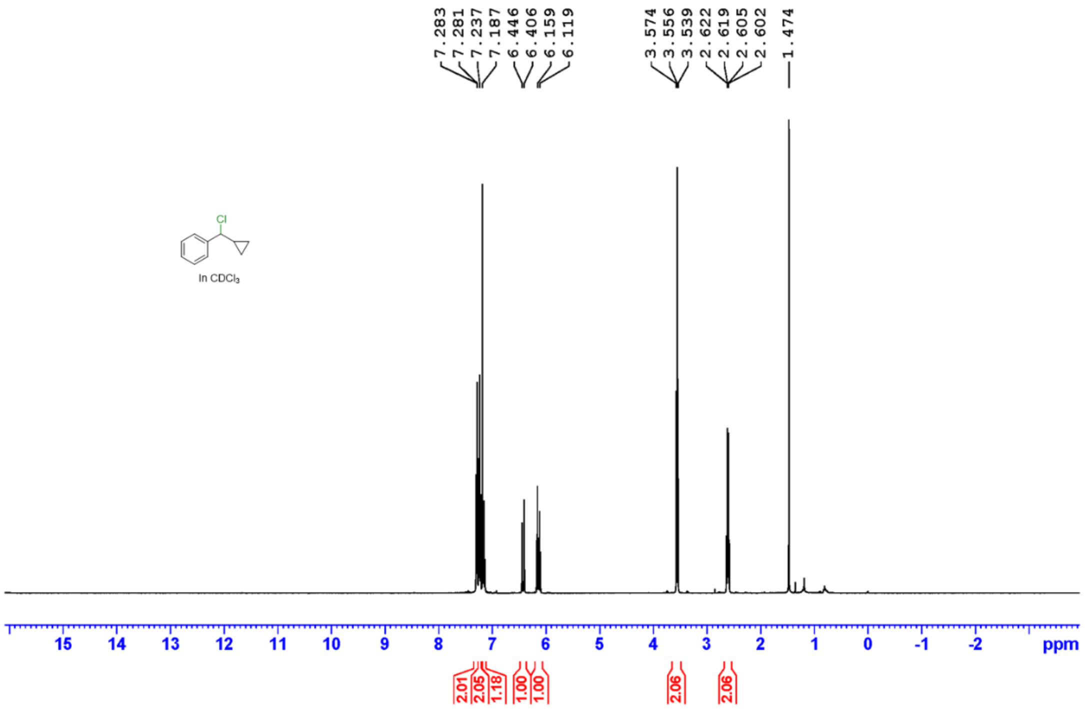
7.283
7.281
7.237
7.187
6.446
6.406
6.159
6.119
3.574
3.556
3.539
2.622
2.619
2.605
2.602
1.474
Cl
In CDCl3
ppm
2.01
2.05
1.18
1.00
1.00
2.06
2.06

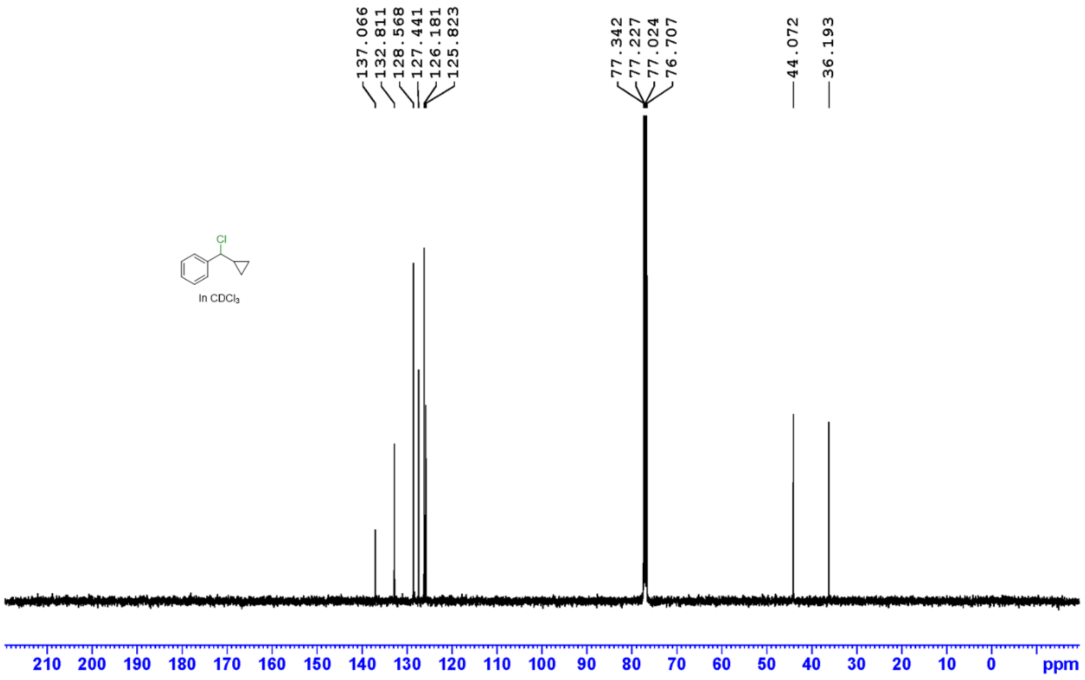
137.066
132.811
128.568
127.441
126.181
125.823
77.342
77.227
77.024
76.707
44.072
36.193
Cl
In CDCl3
ppm

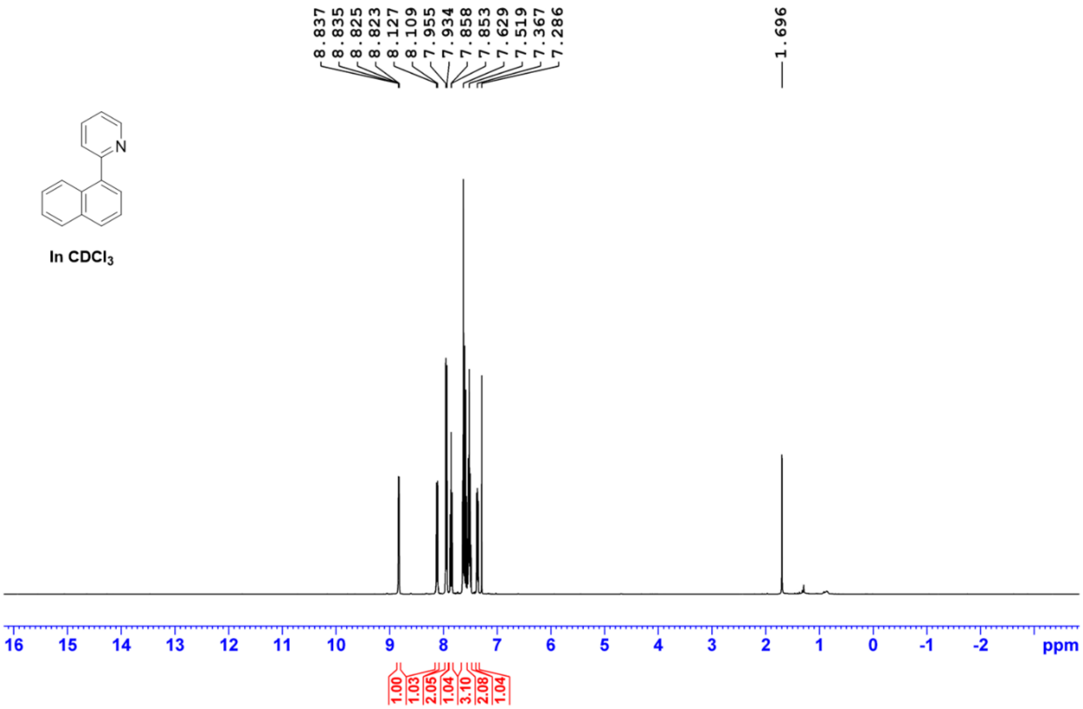
8.837
8.835
8.825
8.823
8.127
8.109
7.955
7.934
7.858
7.853
7.629
7.519
7.367
7.286
1.696
N
In $CDCl_3$
1.00
1.03
2.05
1.04
3.10
2.08
1.04
ppm

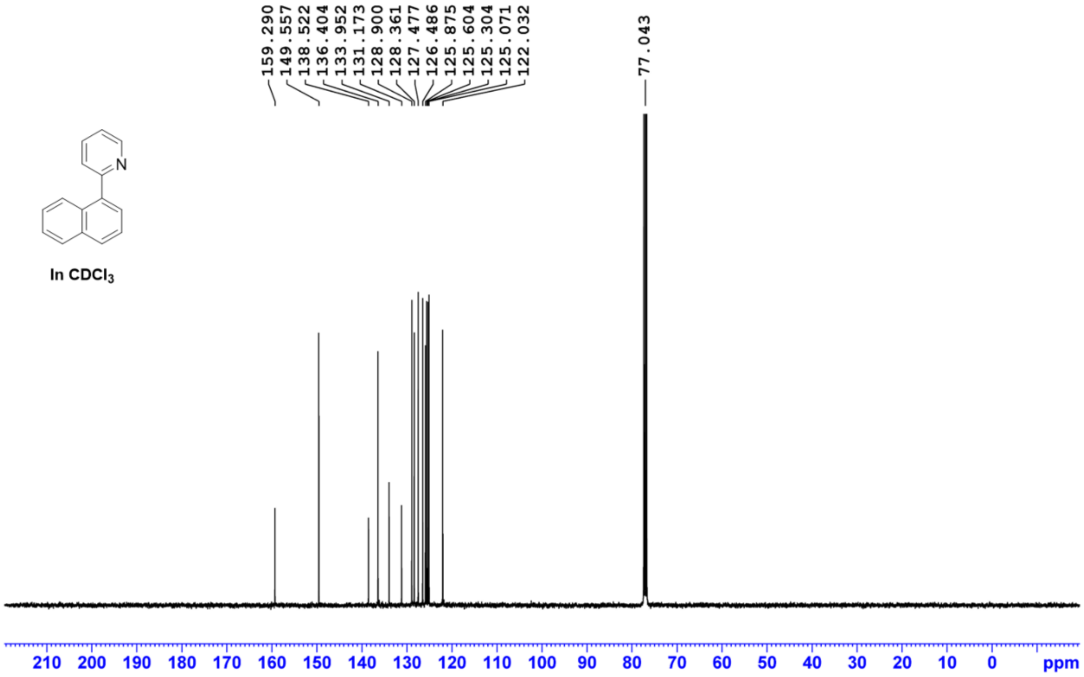
159.290
149.557
138.522
136.404
133.952
131.173
128.900
128.361
127.477
126.486
125.875
125.604
125.304
125.071
122.032
77.043
N
In $CDCl_3$
ppm

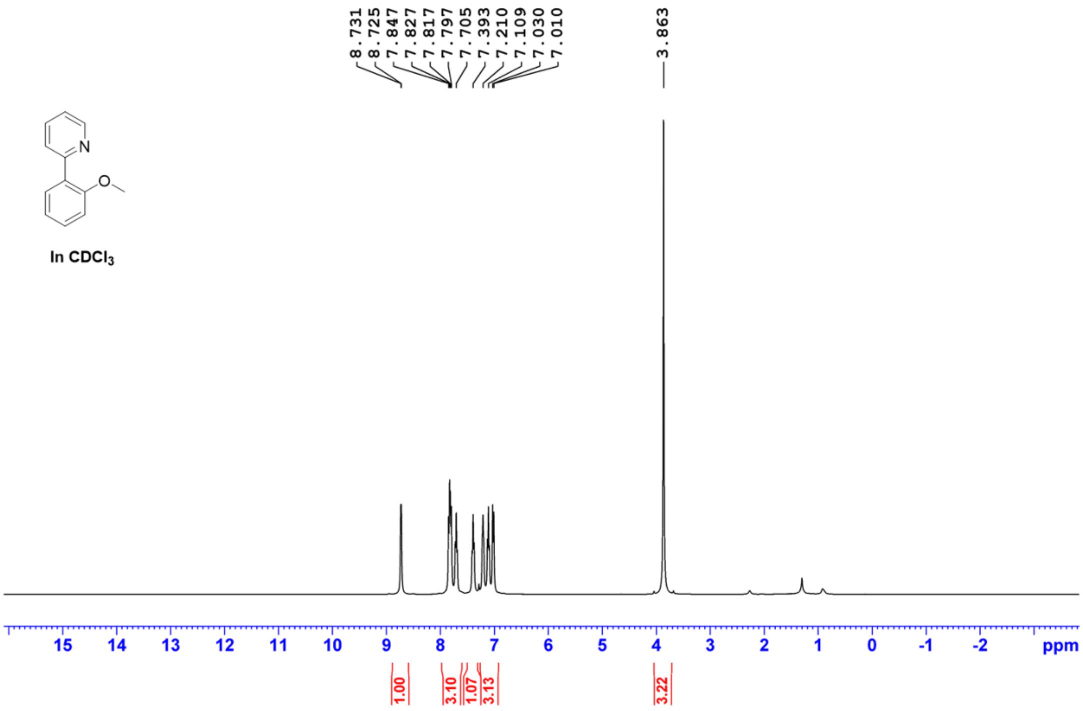
8.731
8.725
7.847
7.827
7.817
7.797
7.705
7.393
7.210
7.109
7.030
7.010
3.863
N
O
In CDCl3
15
14
13
12
11
10
9
8
7
6
5
4
3
2
1
0
-1
-2
ppm
1.00
3.10
1.07
3.13
3.22

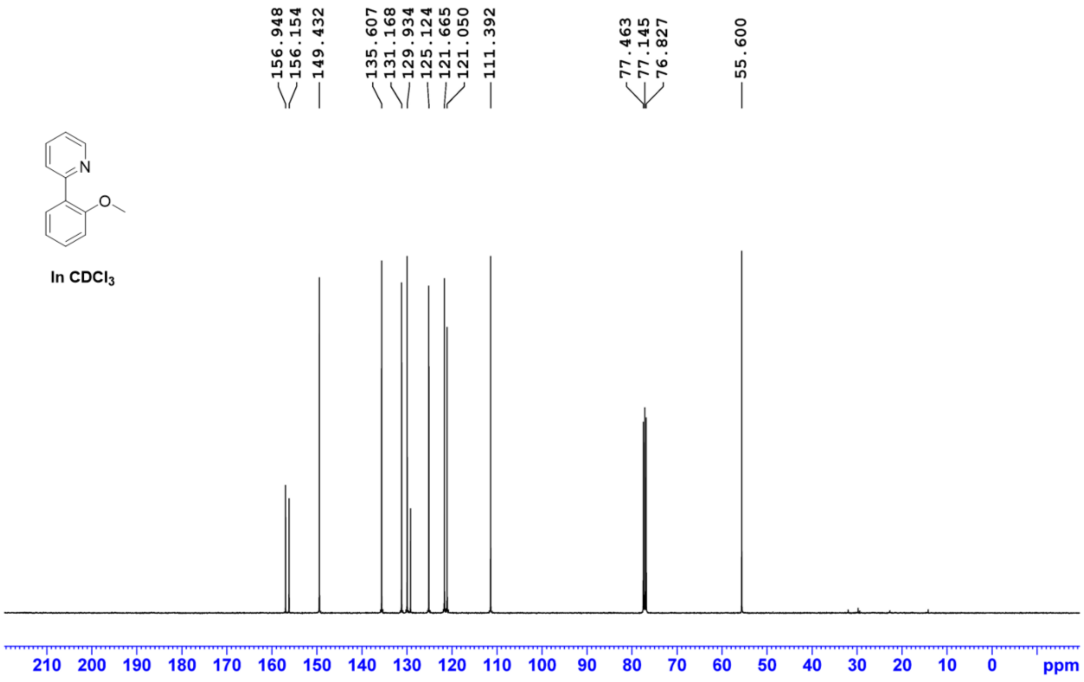
156.948
156.154
149.432
135.607
131.168
129.934
125.124
121.665
121.050
111.392
77.463
77.145
76.827
55.600
N
O
In CDCl3
210
200
190
180
170
160
150
140
130
120
110
100
90
80
70
60
50
40
30
20
10
0
ppm

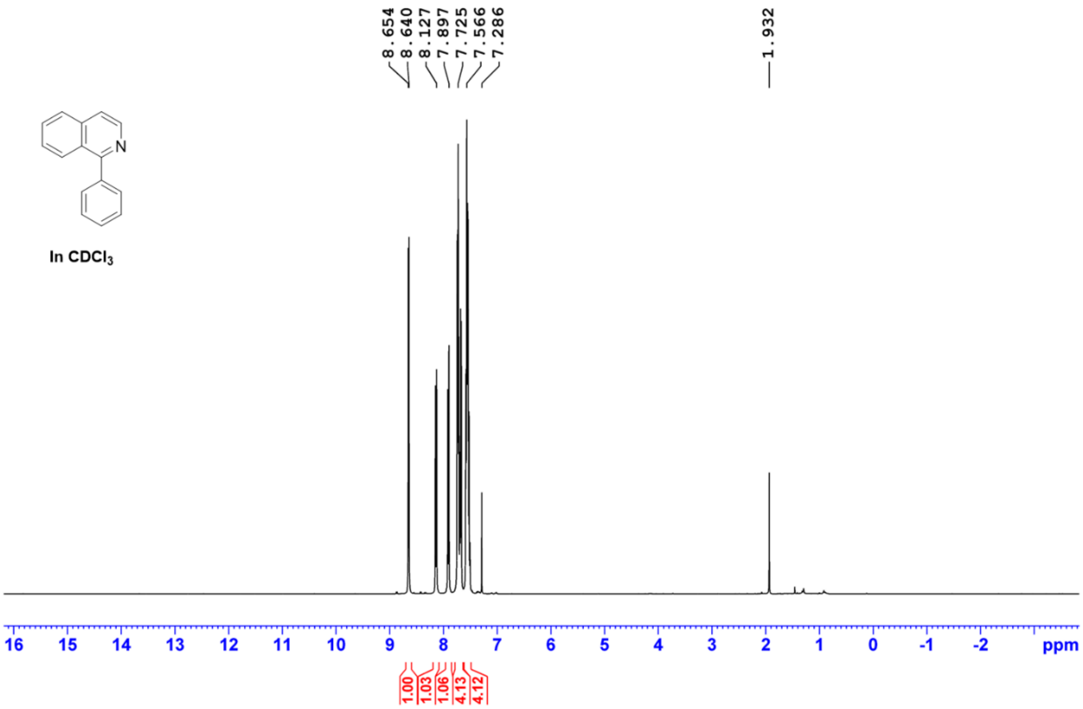
8.654
8.640
8.127
7.897
7.725
7.566
7.286
1.932
N
In $CDCl_3$
16 15 14 13 12 11 10 9 8 7 6 5 4 3 2 1 0 -1 -2 ppm
1.00
1.03
1.06
4.13
4.12

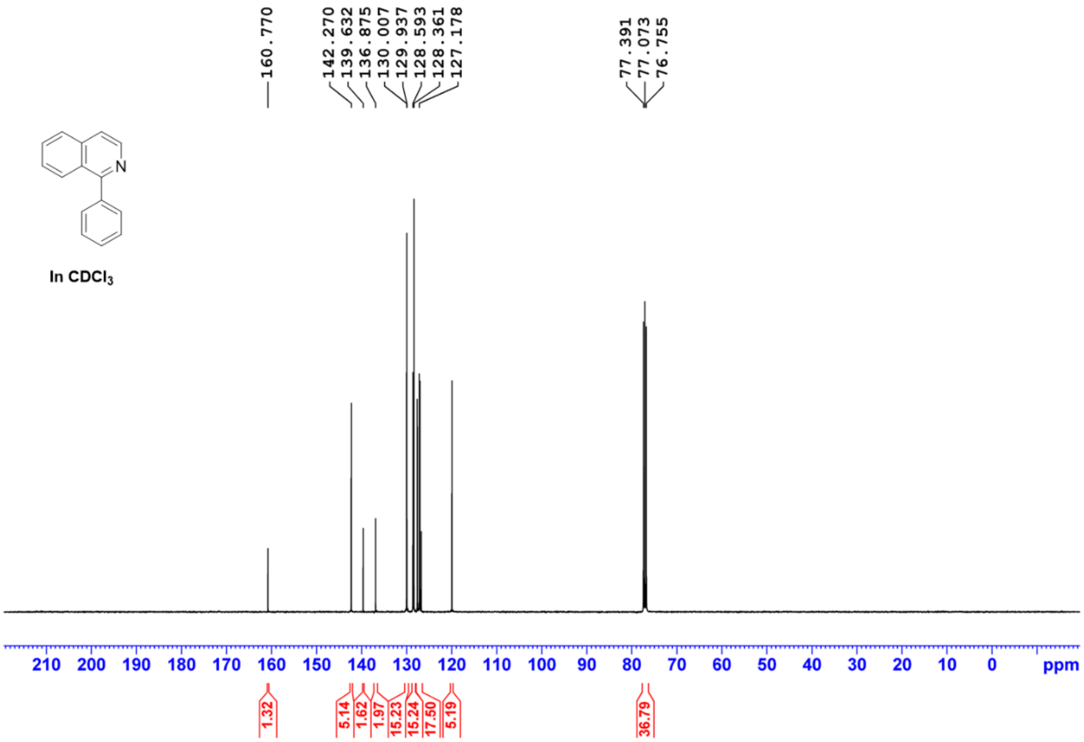
160.770
142.270
139.632
136.875
130.007
129.937
128.593
128.361
127.178
77.391
77.073
76.755
N
In $CDCl_3$
210 200 190 180 170 160 150 140 130 120 110 100 90 80 70 60 50 40 30 20 10 0 ppm
1.32
5.14
1.62
1.97
15.23
15.24
17.50
5.19
36.79

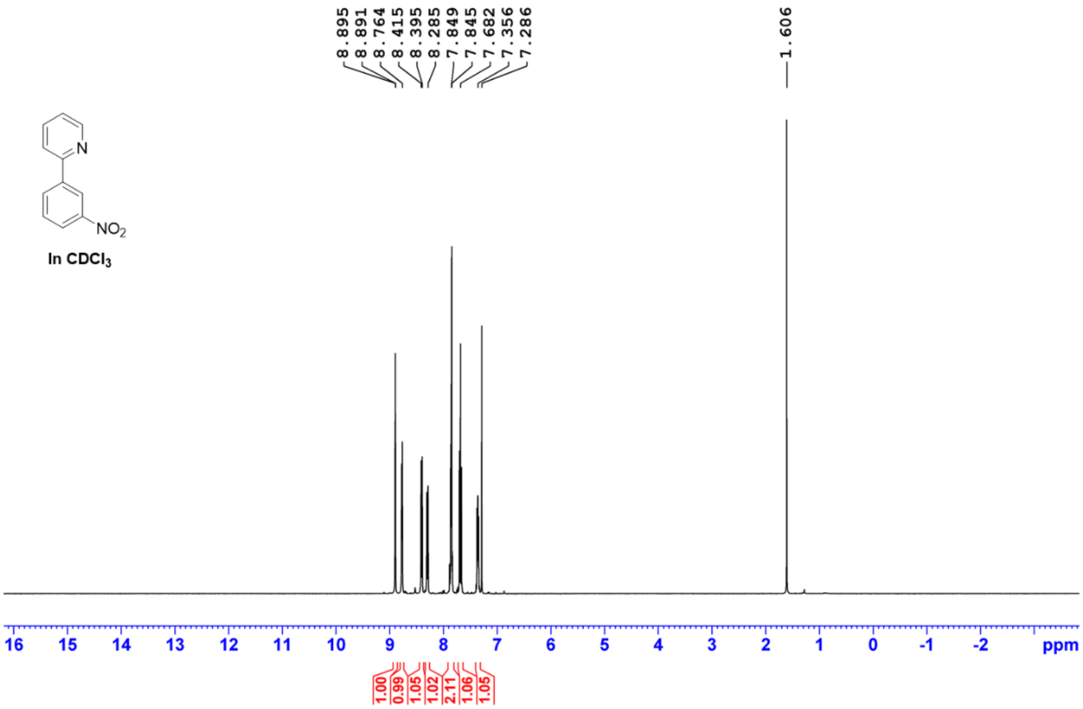
8.895
8.891
8.764
8.415
8.395
8.285
7.849
7.845
7.682
7.356
7.286
1.606
N
NO2
In CDCl3
16 15 14 13 12 11 10 9 8 7 6 5 4 3 2 1 0 -1 -2 ppm
1.00
0.99
1.05
1.02
2.11
1.06
1.05

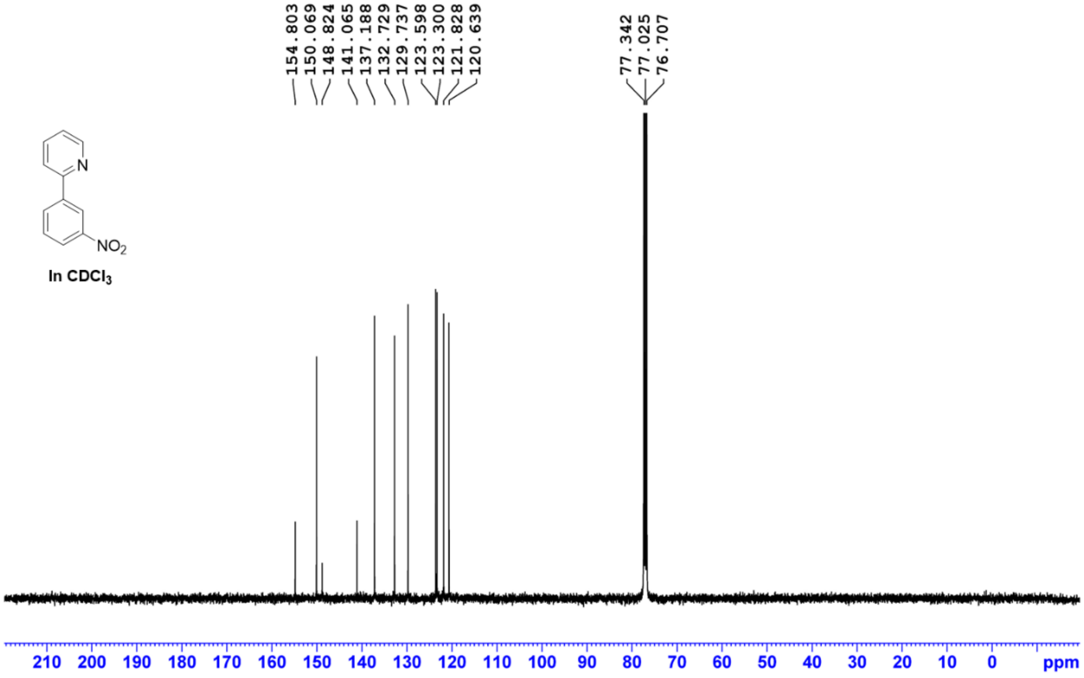
154.803
150.069
148.824
141.065
137.188
132.729
129.737
123.598
123.300
121.828
120.639
77.342
77.025
76.707
N
NO2
In CDCl3
210 200 190 180 170 160 150 140 130 120 110 100 90 80 70 60 50 40 30 20 10 0 ppm

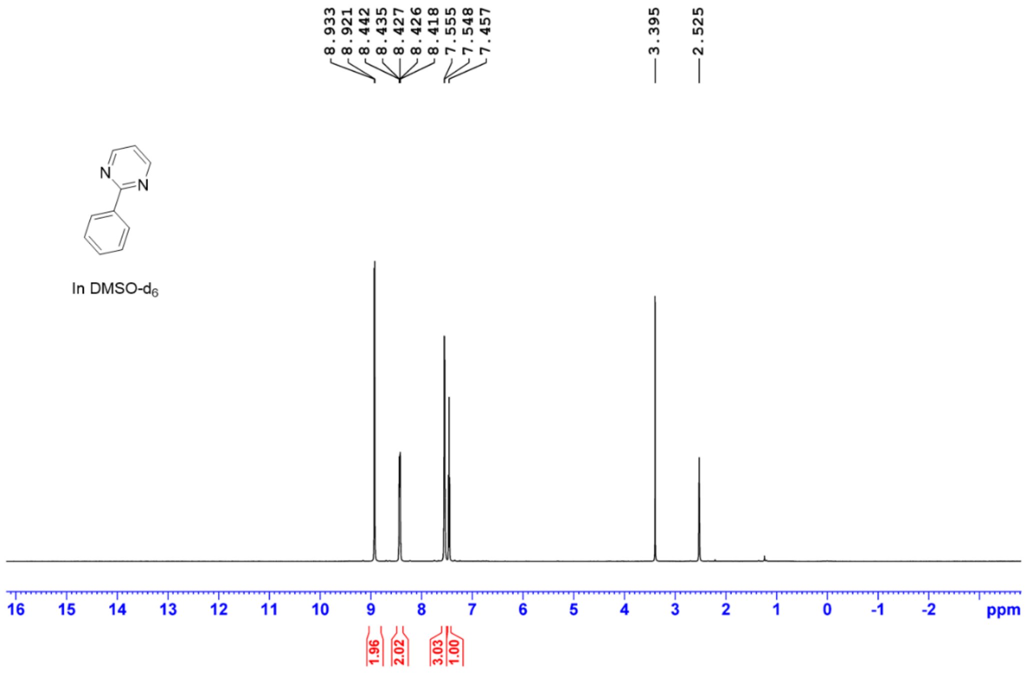
8.933
8.921
8.442
8.435
8.427
8.426
8.418
7.555
7.548
7.457
3.395
2.525
N
N
In DMSO-d6
16 15 14 13 12 11 10 9 8 7 6 5 4 3 2 1 0 -1 -2 ppm
1.96
2.02
3.03
1.00

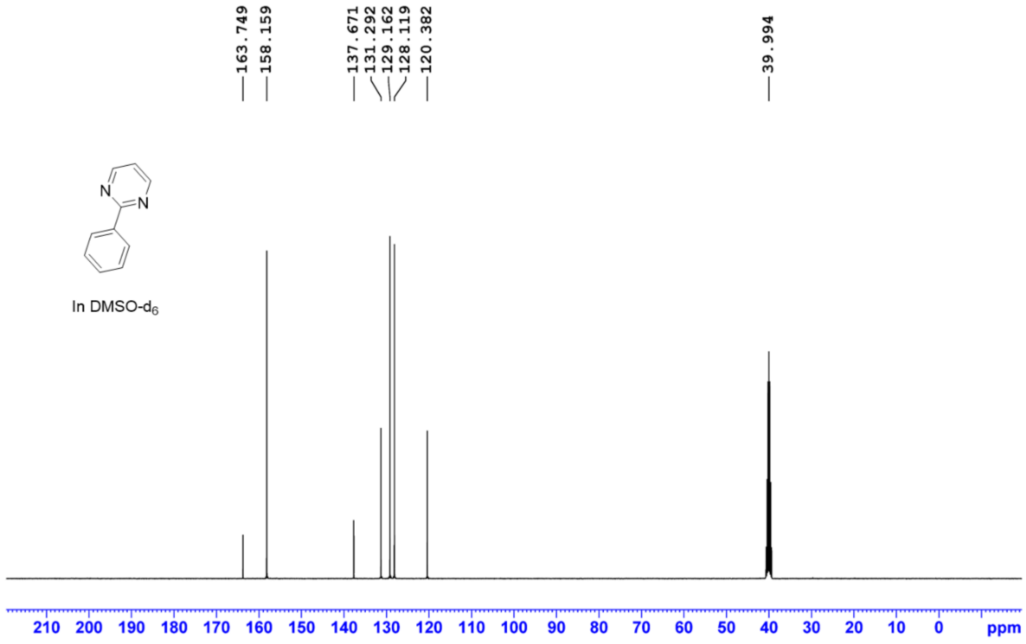
163.749
158.159
137.671
131.292
129.162
128.119
120.382
39.994
N
N
In DMSO-d6
210 200 190 180 170 160 150 140 130 120 110 100 90 80 70 60 50 40 30 20 10 0 ppm

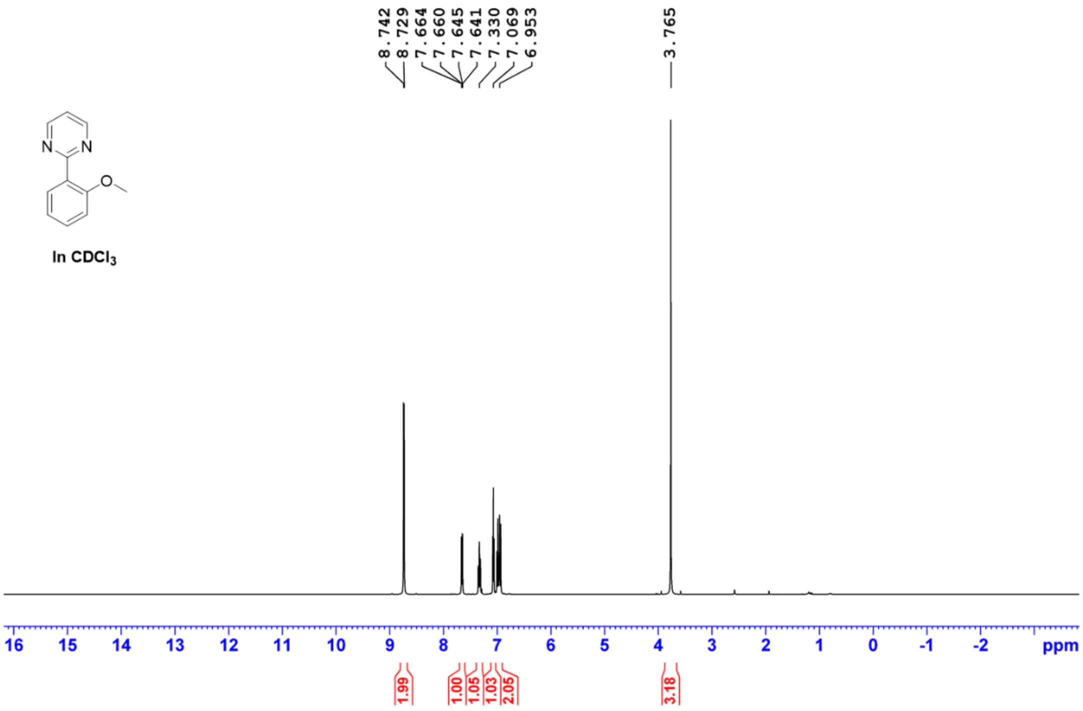
8.742
8.729
7.664
7.660
7.645
7.641
7.330
7.069
6.953
3.765
N
N
O
In CDCl3
16 15 14 13 12 11 10 9 8 7 6 5 4 3 2 1 0 -1 -2 ppm
1.99
1.00
1.05
1.03
2.05
3.18

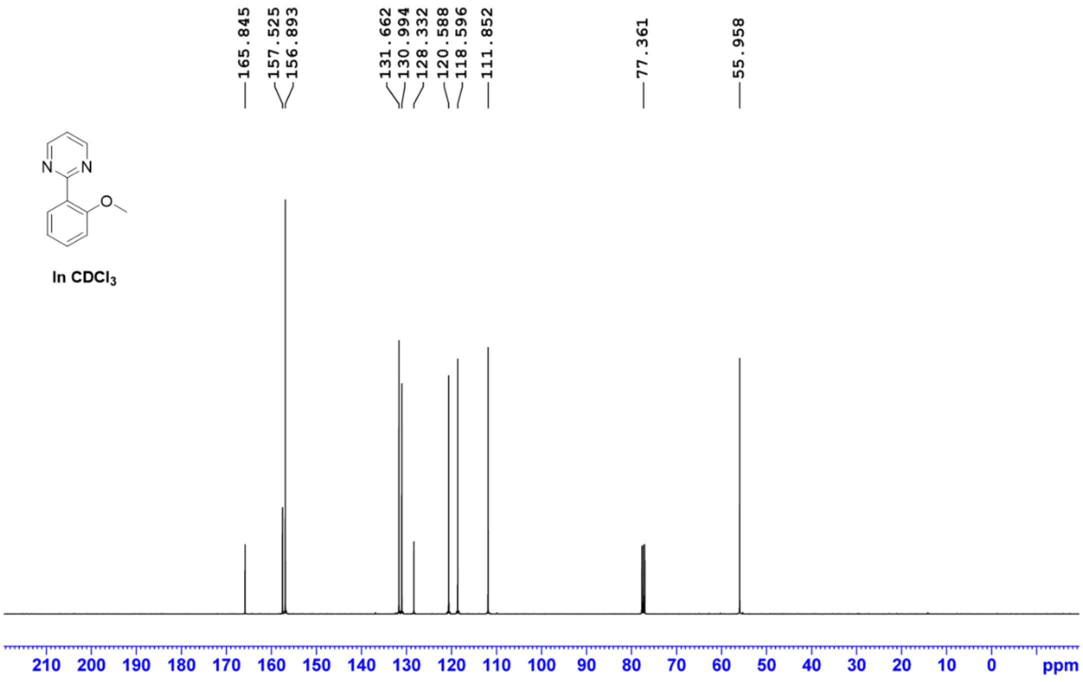
165.845
157.525
156.893
131.662
130.994
128.332
120.588
118.596
111.852
77.361
55.958
N
N
O
In CDCl3
210 200 190 180 170 160 150 140 130 120 110 100 90 80 70 60 50 40 30 20 10 0 ppm

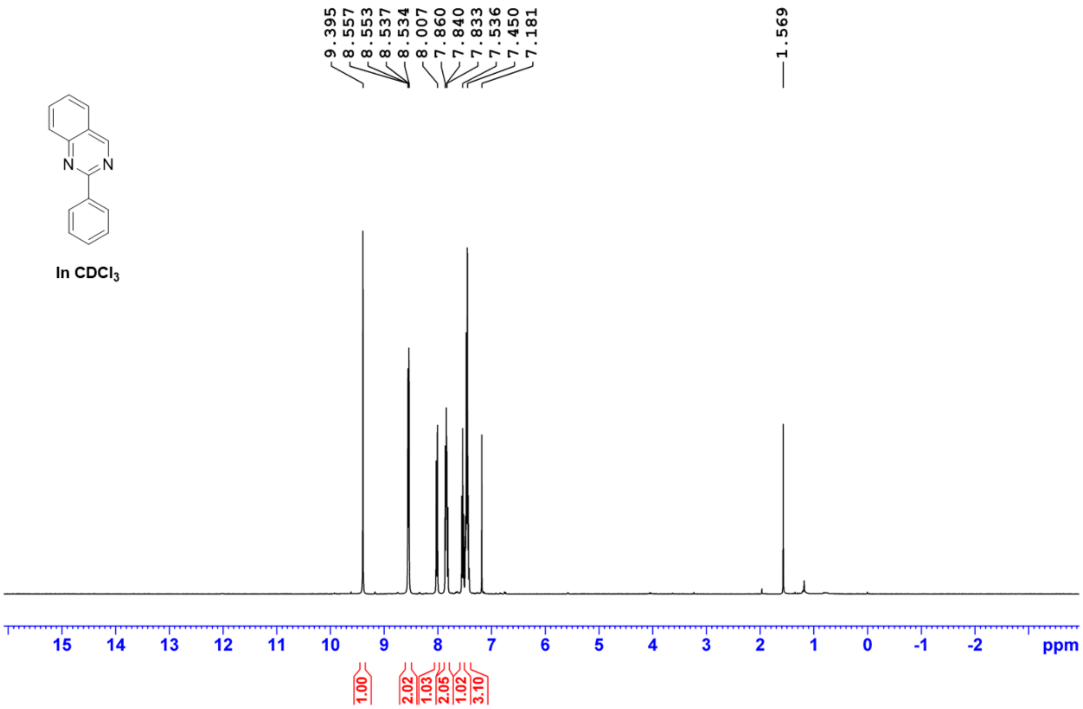
9.395
8.557
8.553
8.537
8.534
8.007
7.860
7.840
7.833
7.536
7.450
7.181
1.569
In CDCl3
15 14 13 12 11 10 9 8 7 6 5 4 3 2 1 0 -1 -2 ppm
1.00
2.02
1.03
2.05
1.02
3.10

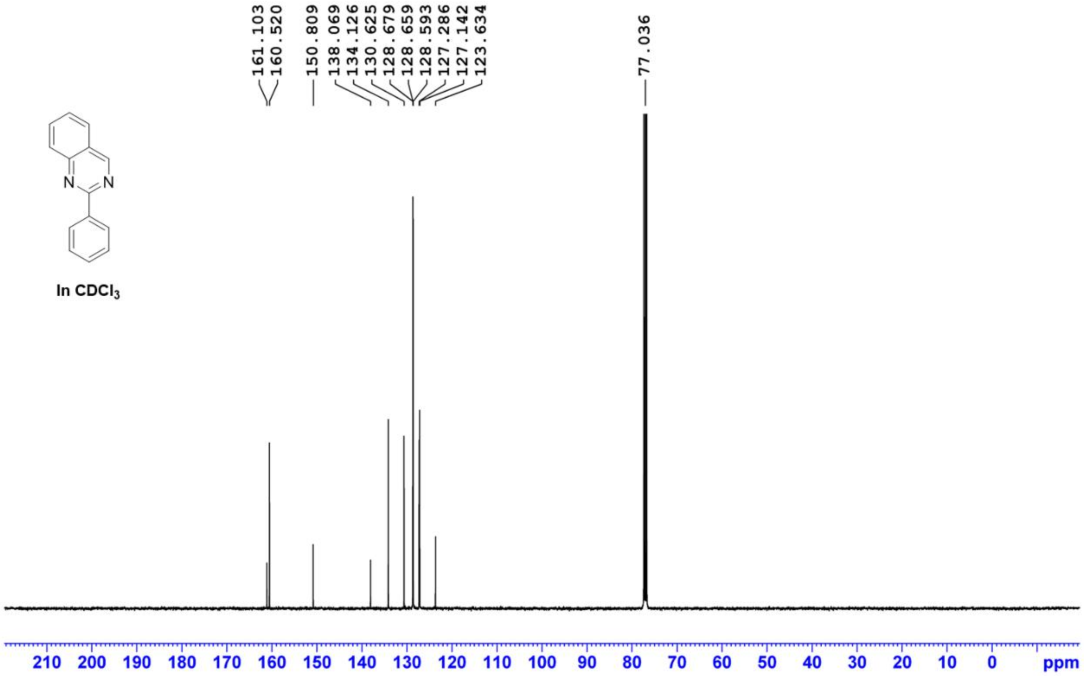
161.103
160.520
150.809
138.069
134.126
130.625
128.679
128.659
128.593
127.286
127.142
123.634
77.036
In CDCl3
210 200 190 180 170 160 150 140 130 120 110 100 90 80 70 60 50 40 30 20 10 0 ppm

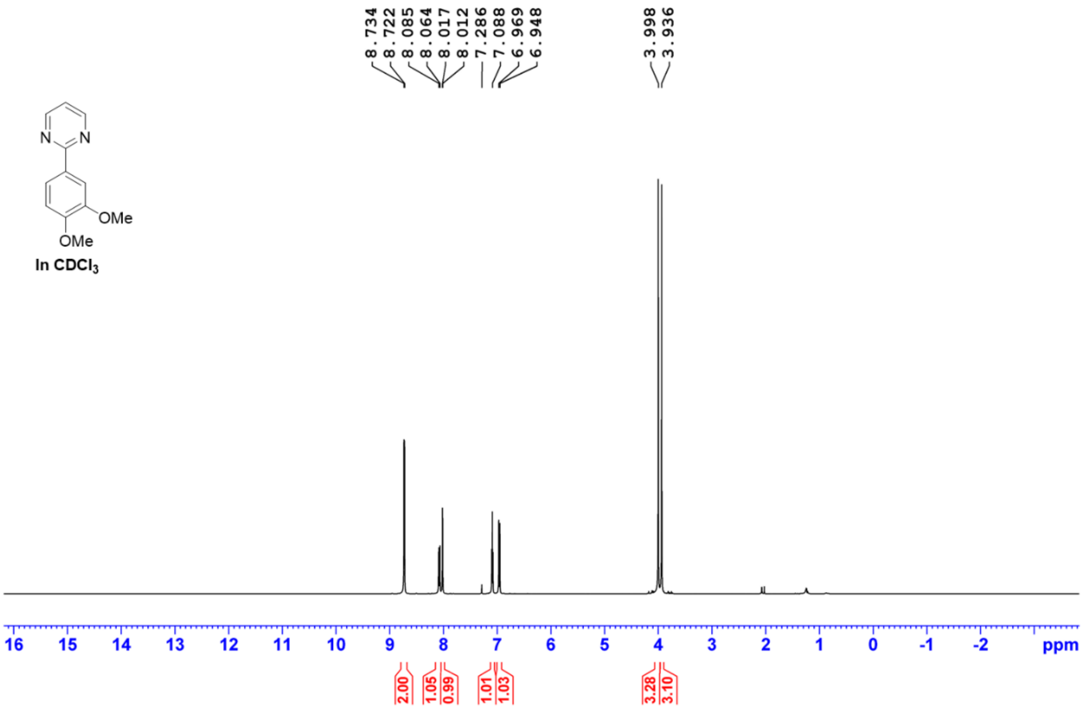
8.734
8.722
8.085
8.064
8.017
8.012
7.286
7.088
6.969
6.948
3.998
3.936
N
N
OMe
OMe
In CDCl3
16 15 14 13 12 11 10 9 8 7 6 5 4 3 2 1 0 -1 -2 ppm
2.00
1.05
0.99
1.01
1.03
3.28
3.10

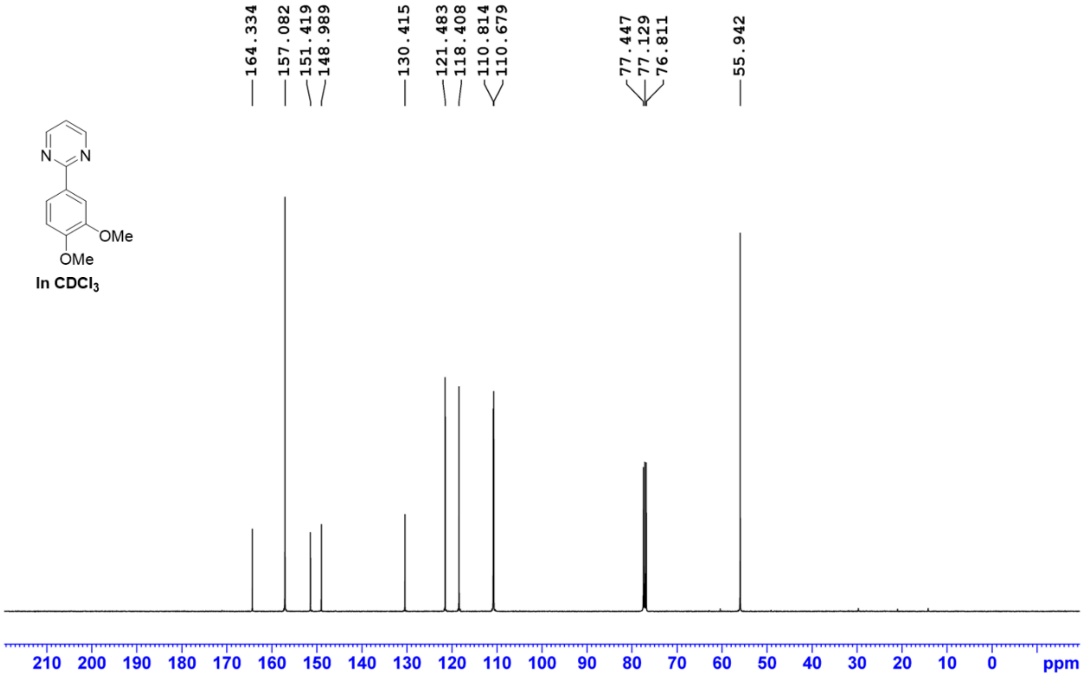
164.334
157.082
151.419
148.989
130.415
121.483
118.408
110.814
110.679
77.447
77.129
76.811
55.942
N
N
OMe
OMe
In CDCl3
210 200 190 180 170 160 150 140 130 120 110 100 90 80 70 60 50 40 30 20 10 0 ppm

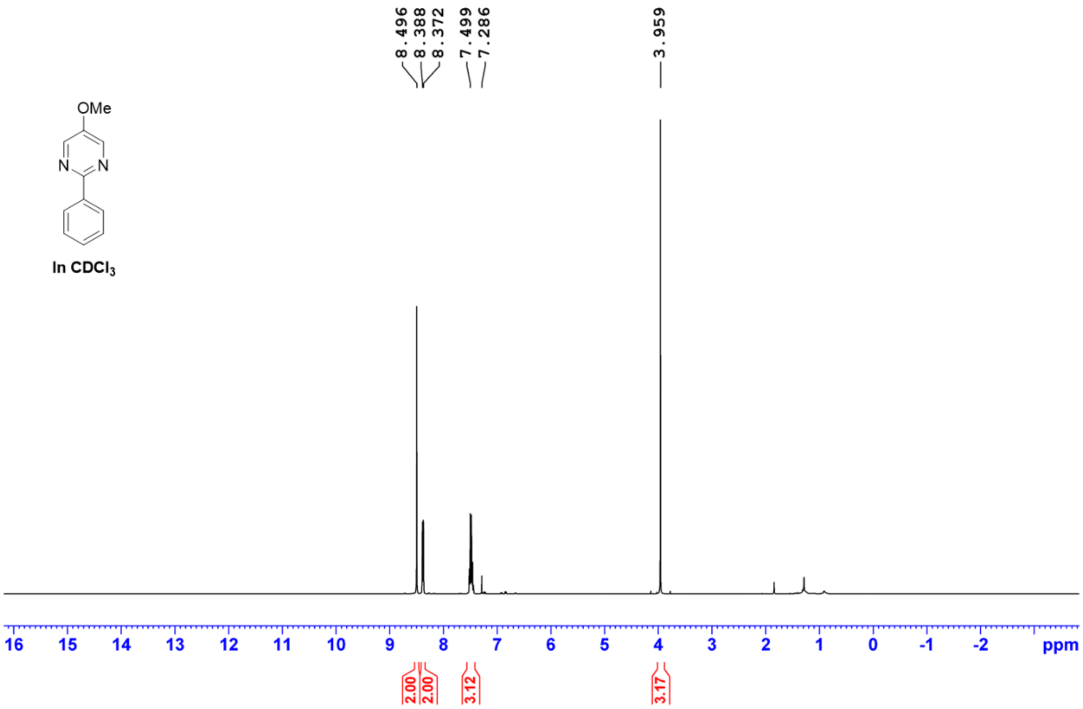
8.496
8.388
8.372
7.499
7.286
3.959
OMe
N
N
In CDCl3
16
15
14
13
12
11
10
9
8
7
6
5
4
3
2
1
0
-1
-2
ppm
2.00
2.00
3.12
3.17

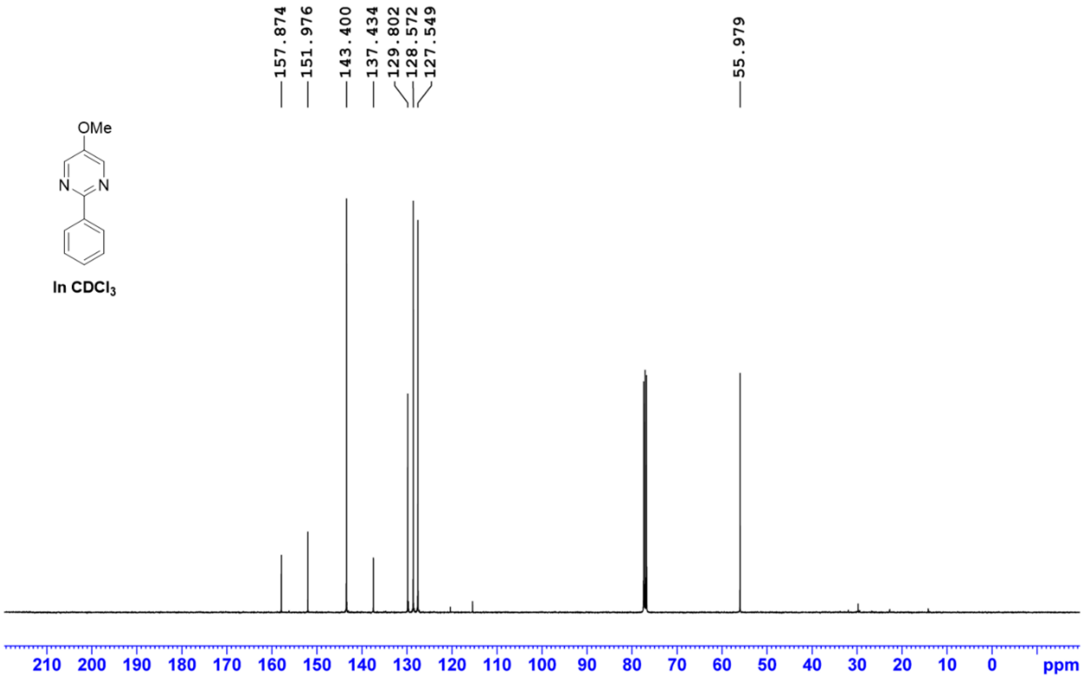
157.874
151.976
143.400
137.434
129.802
128.572
127.549
55.979
OMe
N
N
In CDCl3
210
200
190
180
170
160
150
140
130
120
110
100
90
80
70
60
50
40
30
20
10
0
ppm

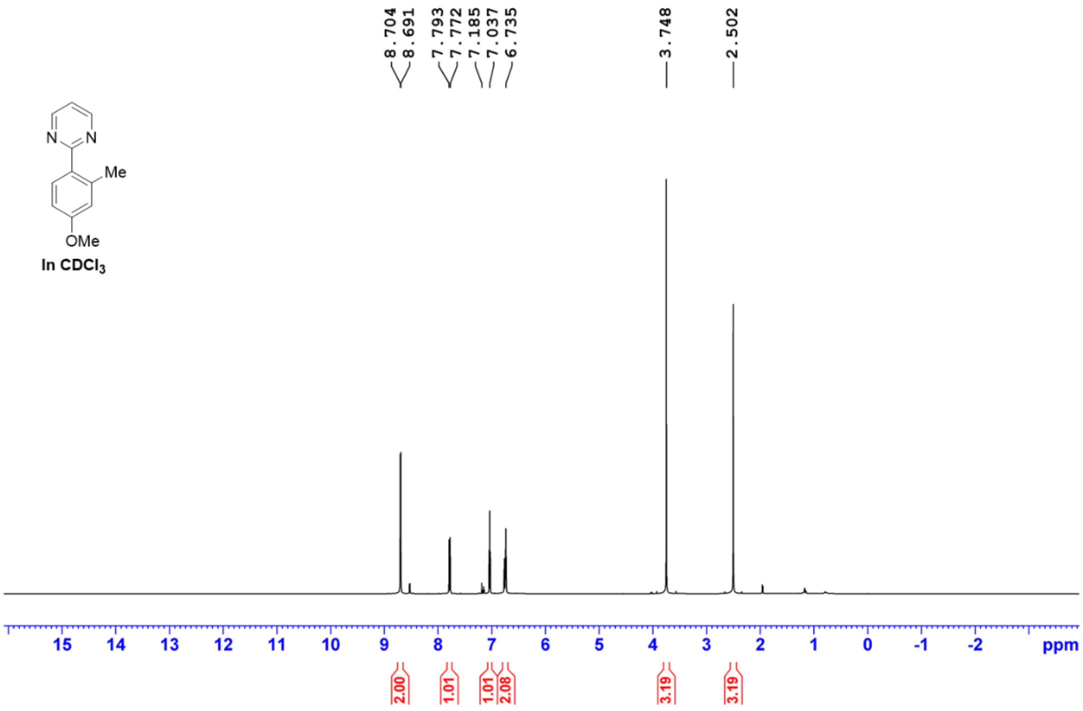

8.704
8.691
7.793
7.772
7.185
7.037
6.735
3.748
2.502
Me
OMe
In CDCl3
15 14 13 12 11 10 9 8 7 6 5 4 3 2 1 0 -1 -2 ppm
2.00
1.01
1.01
2.08
3.19
3.19

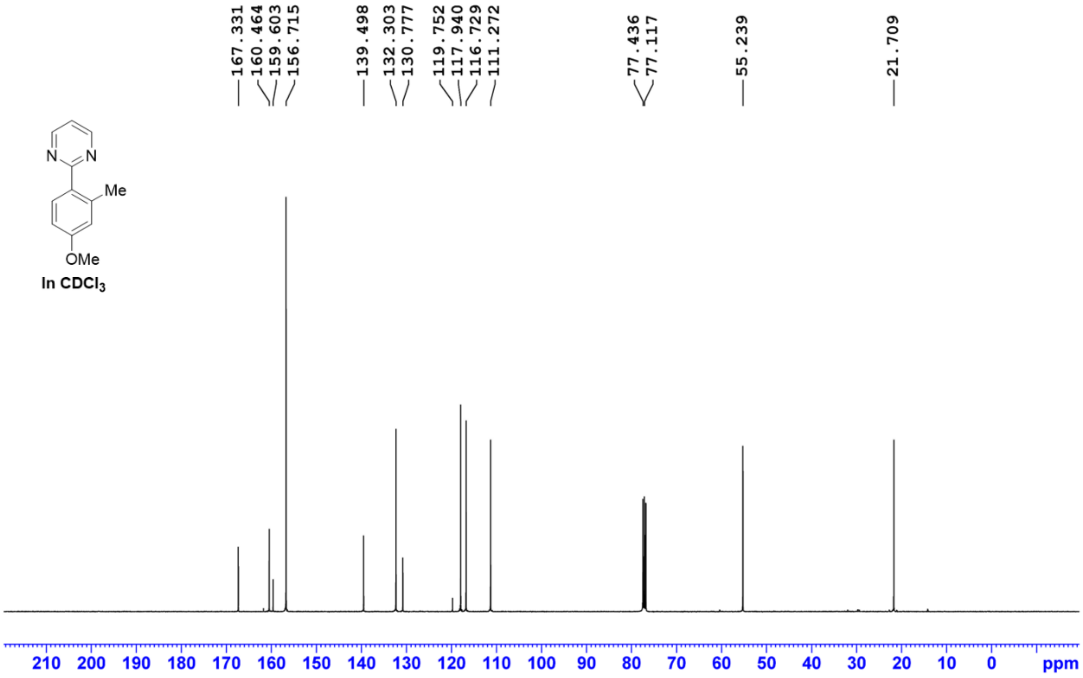

167.331
160.464
159.603
156.715
139.498
132.303
130.777
119.752
117.940
116.729
111.272
77.436
77.117
55.239
21.709
Me
OMe
In CDCl3
210 200 190 180 170 160 150 140 130 120 110 100 90 80 70 60 50 40 30 20 10 0 ppm

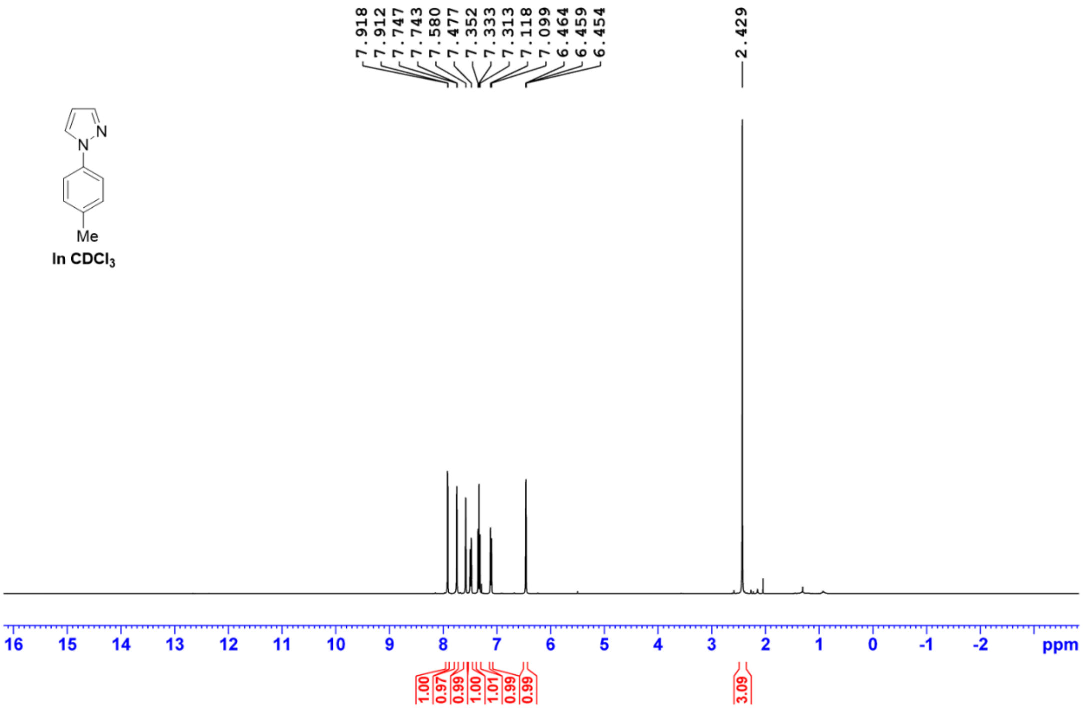
7.918
7.912
7.747
7.743
7.580
7.477
7.352
7.333
7.313
7.118
7.099
6.464
6.459
6.454
2.429
N
N
Me
In $CDCl_3$
1.00
0.97
0.99
1.00
1.01
0.99
0.99
3.09
ppm

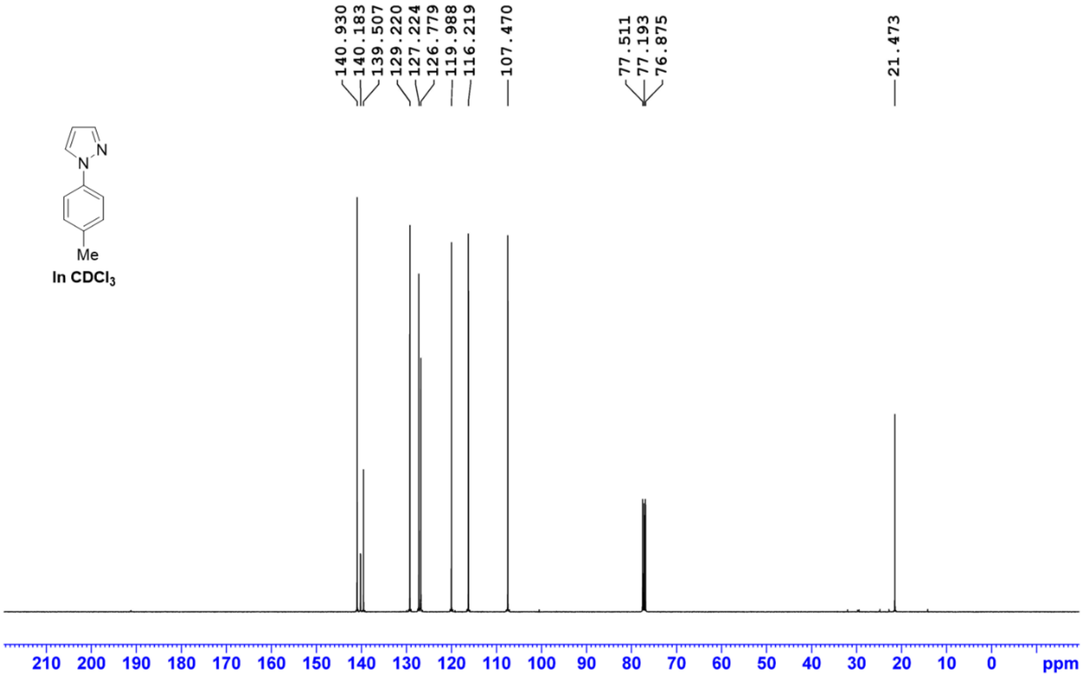
140.930
140.183
139.507
129.220
127.224
126.779
119.988
116.219
107.470
77.511
77.193
76.875
21.473
N
N
Me
In $CDCl_3$
ppm

Supplementary Information

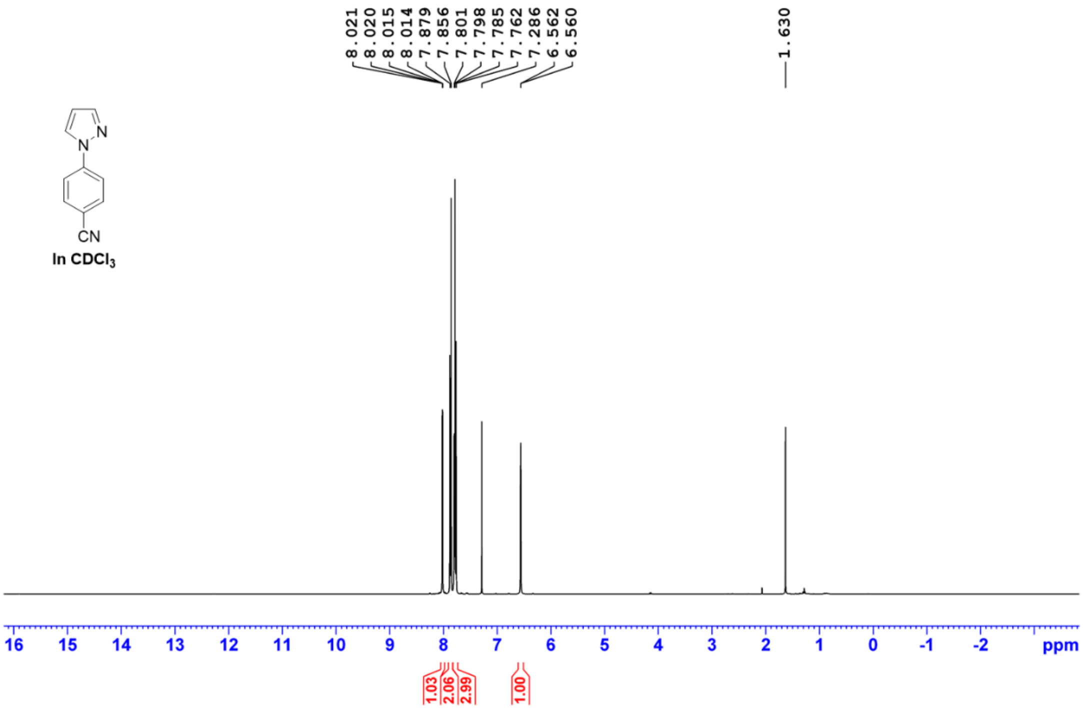

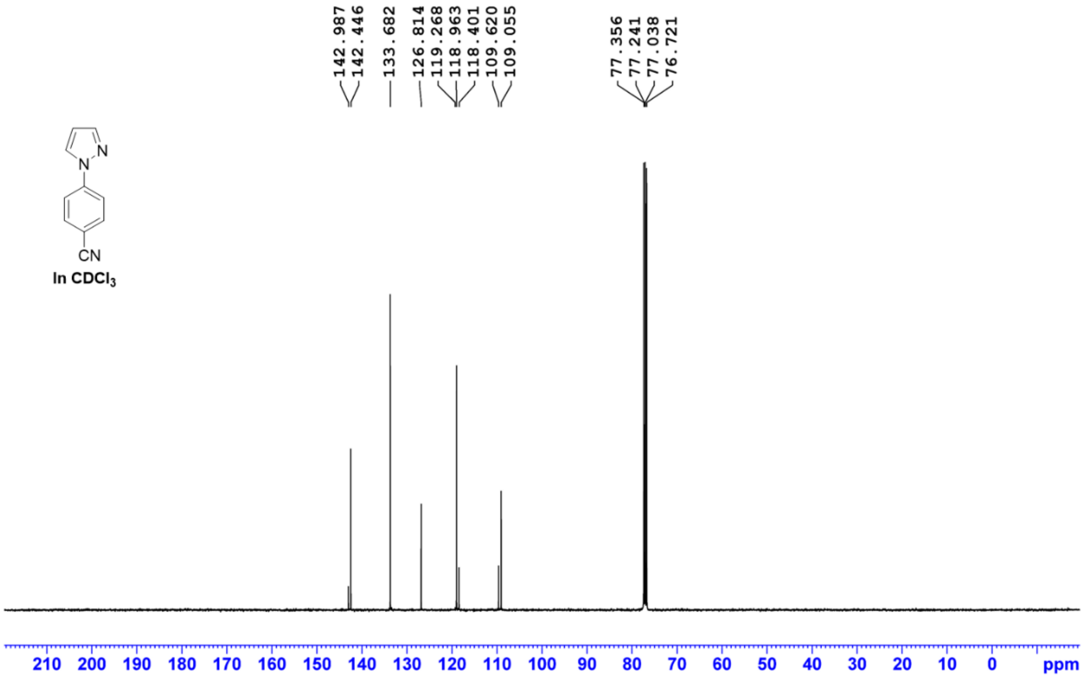

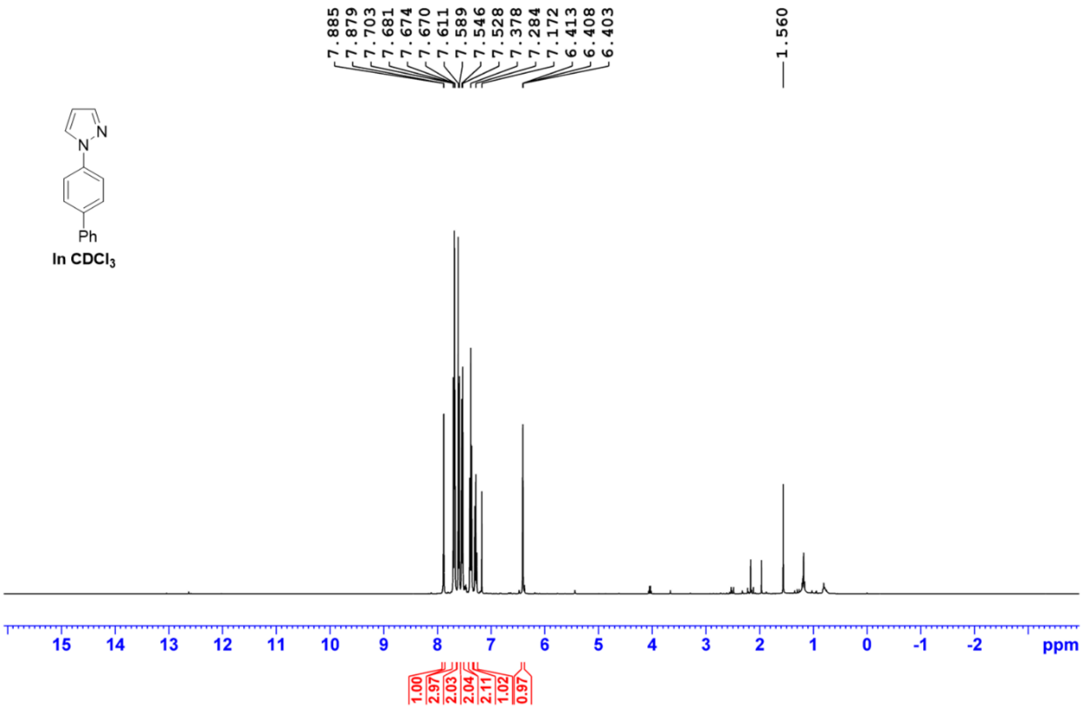
7.885
7.879
7.703
7.681
7.674
7.670
7.611
7.589
7.546
7.528
7.378
7.284
7.172
6.413
6.408
6.403
1.560
N
N
Ph
In $CDCl_3$
1.00
2.97
2.03
2.04
2.11
1.02
0.97
15 14 13 12 11 10 9 8 7 6 5 4 3 2 1 0 -1 -2 ppm

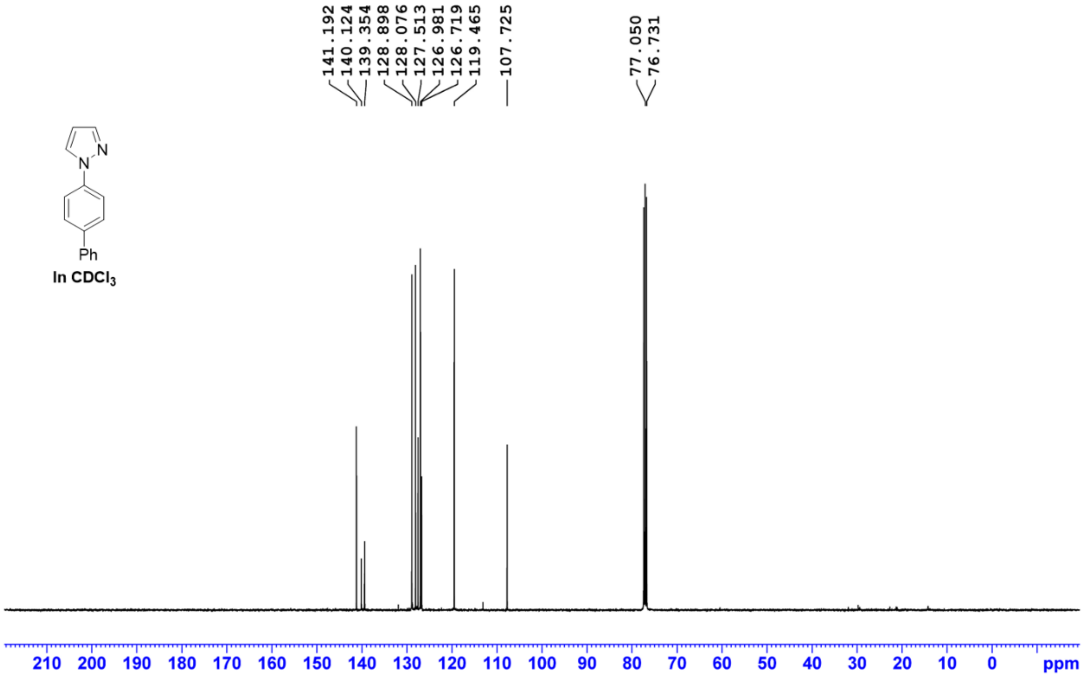
141.192
140.124
139.354
128.898
128.076
127.513
126.981
126.719
119.465
107.725
77.050
76.731
N
N
Ph
In $CDCl_3$
210 200 190 180 170 160 150 140 130 120 110 100 90 80 70 60 50 40 30 20 10 0 ppm

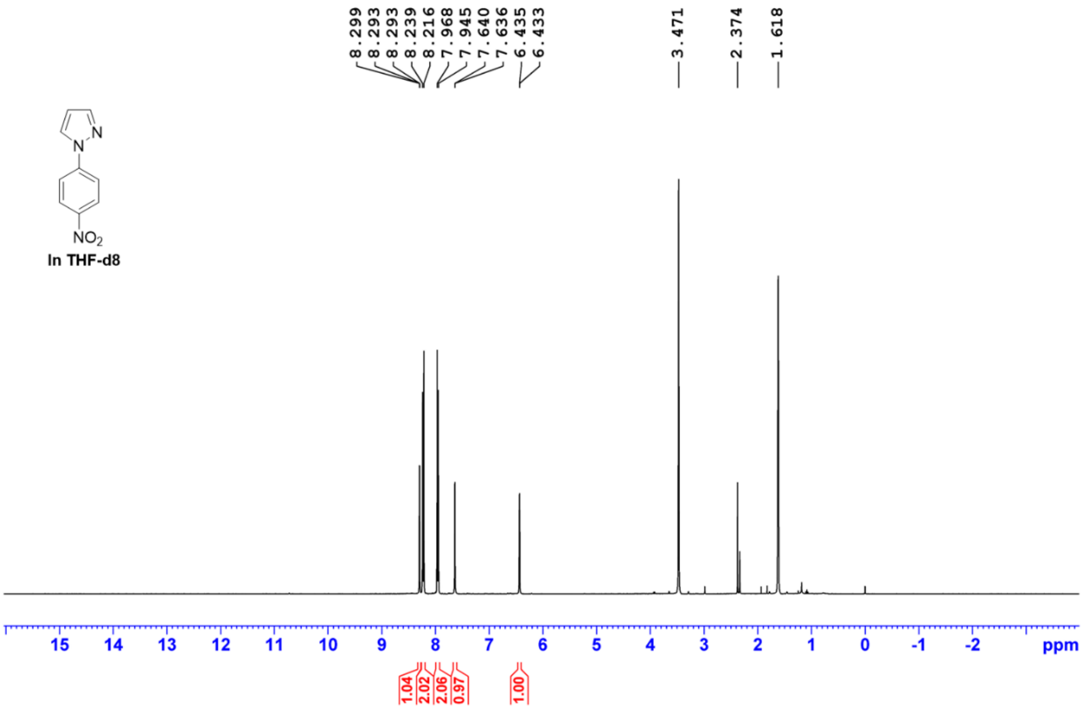
8.299
8.293
8.293
8.239
8.216
7.968
7.945
7.640
7.636
6.435
6.433
3.471
2.374
1.618
N
N
NO2
In THF-d8
15
14
13
12
11
10
9
8
7
6
5
4
3
2
1
0
-1
-2
ppm
1.04
2.02
2.06
0.97
1.00

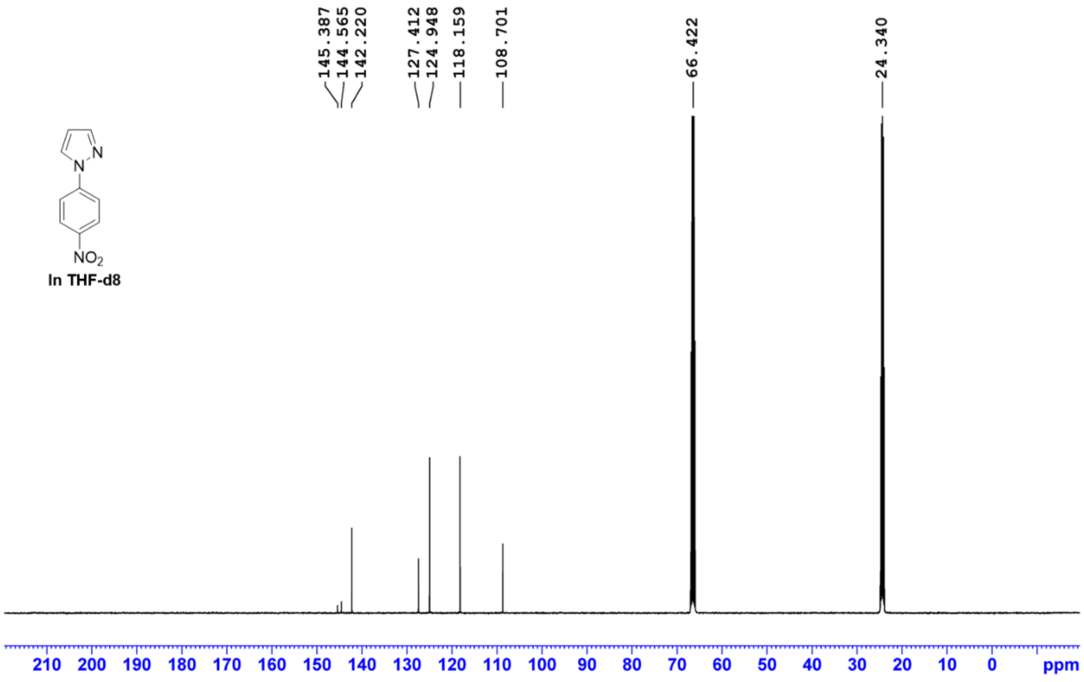
145.387
144.565
142.220
127.412
124.948
118.159
108.701
66.422
24.340
N
N
NO2
In THF-d8
210
200
190
180
170
160
150
140
130
120
110
100
90
80
70
60
50
40
30
20
10
0
ppm

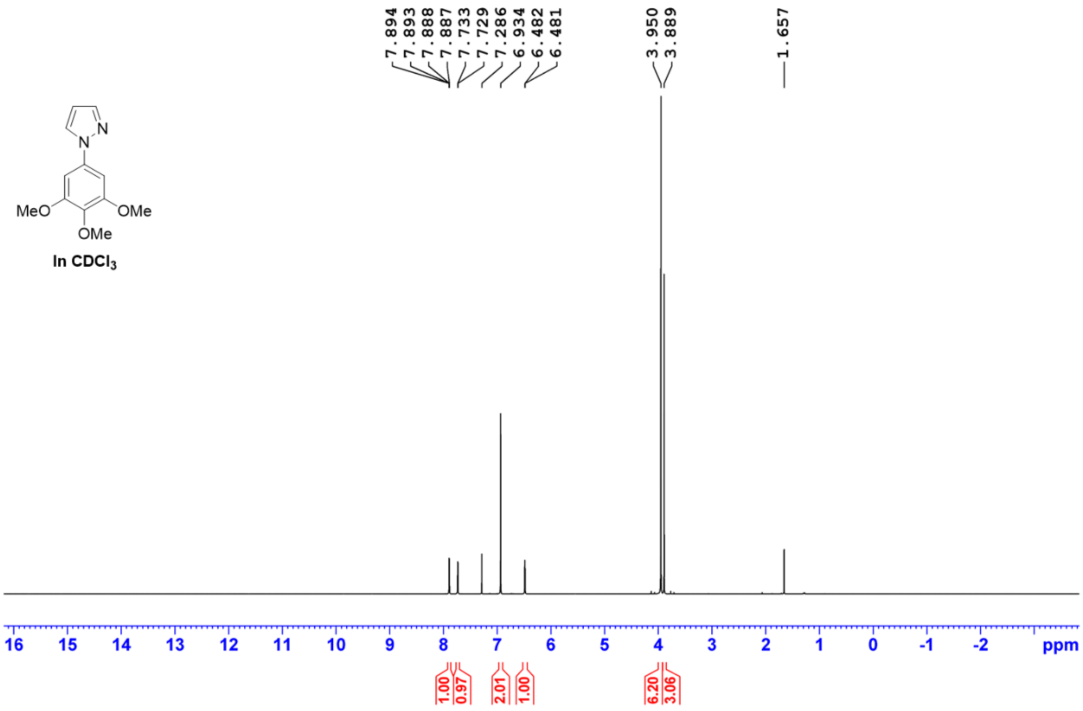
7.894
7.893
7.888
7.887
7.733
7.729
7.286
6.934
6.482
6.481
3.950
3.889
1.657
N
N
MeO
OMe
OMe
In CDCl3
ppm
1.00
0.97
2.01
1.00
6.20
3.06

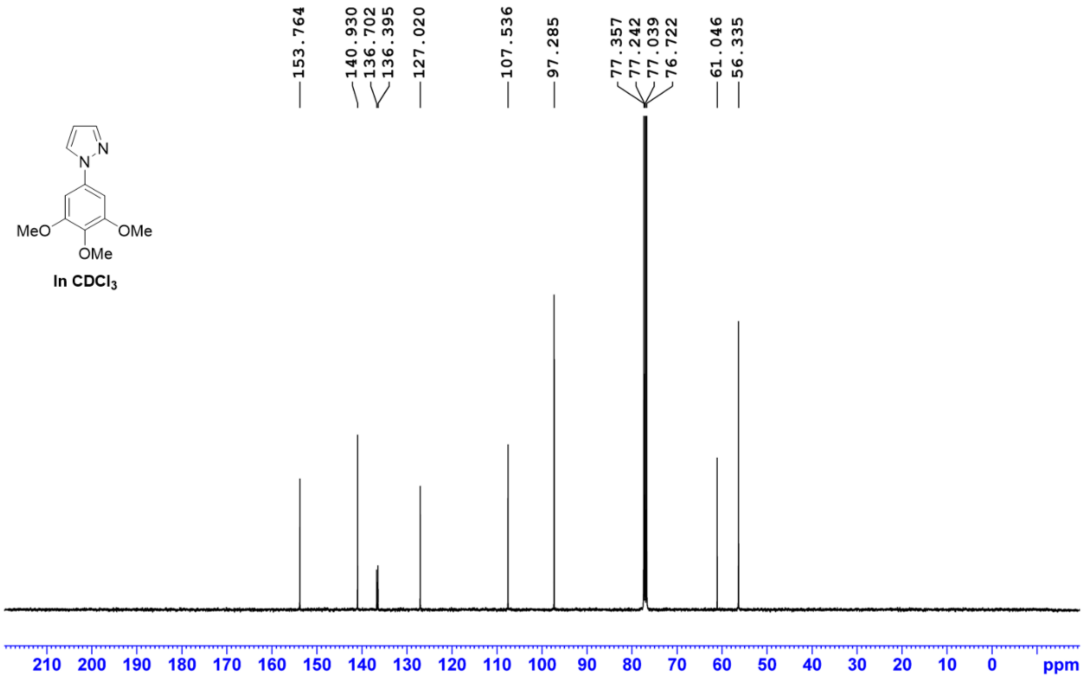
153.764
140.930
136.702
136.395
127.020
107.536
97.285
77.357
77.242
77.039
76.722
61.046
56.335
N
N
MeO
OMe
OMe
In CDCl3
ppm

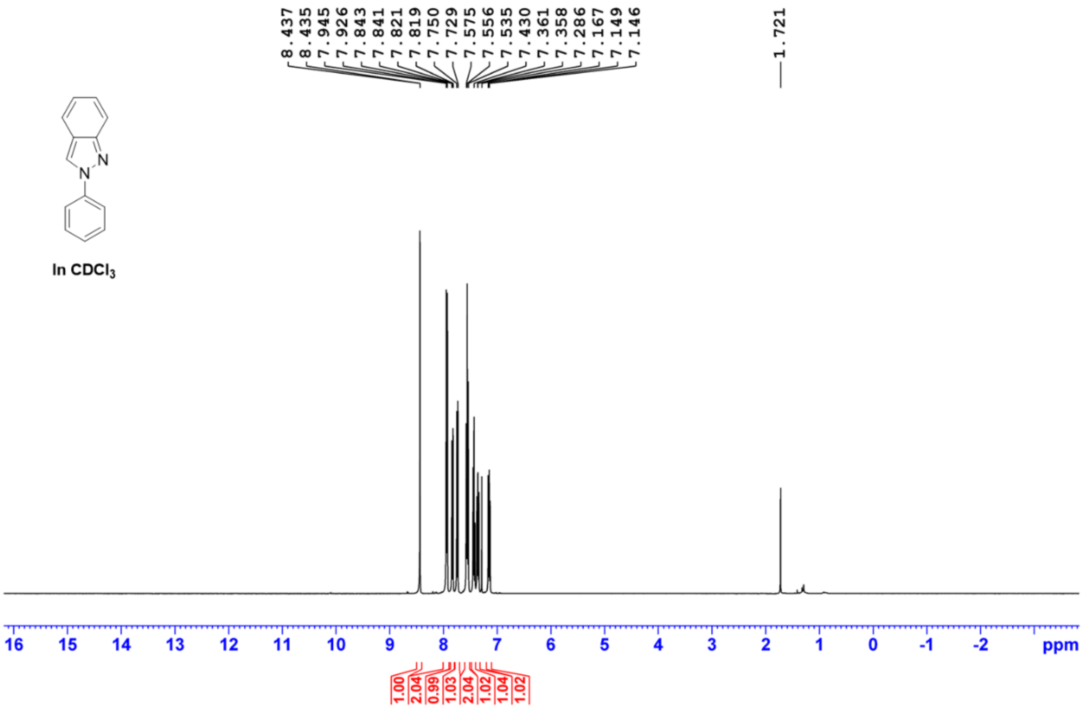
8.437
8.435
7.945
7.926
7.843
7.841
7.821
7.819
7.750
7.729
7.575
7.556
7.535
7.430
7.361
7.358
7.286
7.167
7.149
7.146
1.721
N
N
In CDCl3
1.00
2.04
0.99
1.03
2.04
1.02
1.04
1.02
ppm

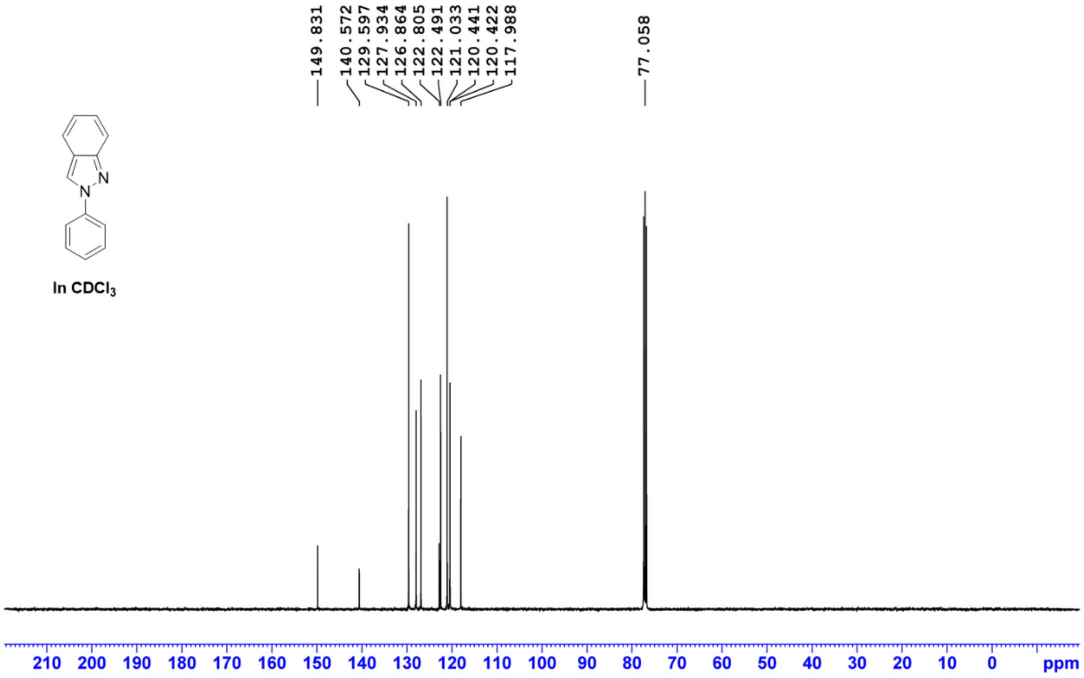
149.831
140.572
129.597
127.934
126.864
122.805
122.491
121.033
120.441
120.422
117.988
77.058
N
N
In CDCl3
ppm

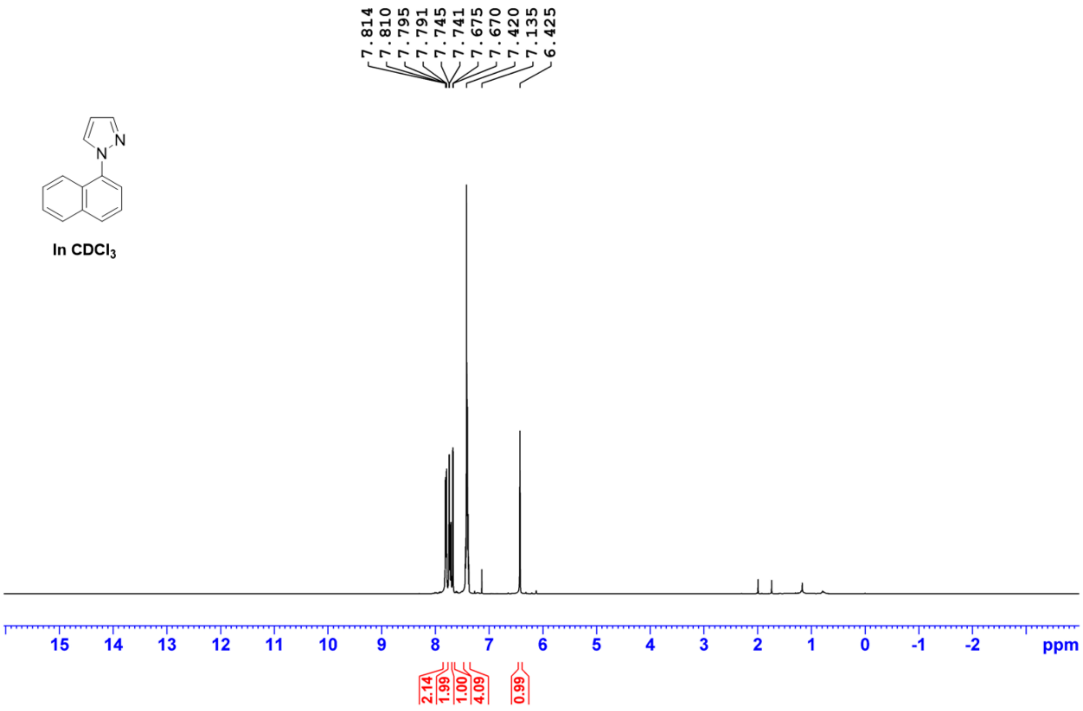

7.814
7.810
7.795
7.791
7.745
7.741
7.675
7.670
7.420
7.135
6.425
N
N
In $CDCl_3$
15
14
13
12
11
10
9
8
7
6
5
4
3
2
1
0
-1
-2
ppm
2.14
1.99
1.00
4.09
0.99

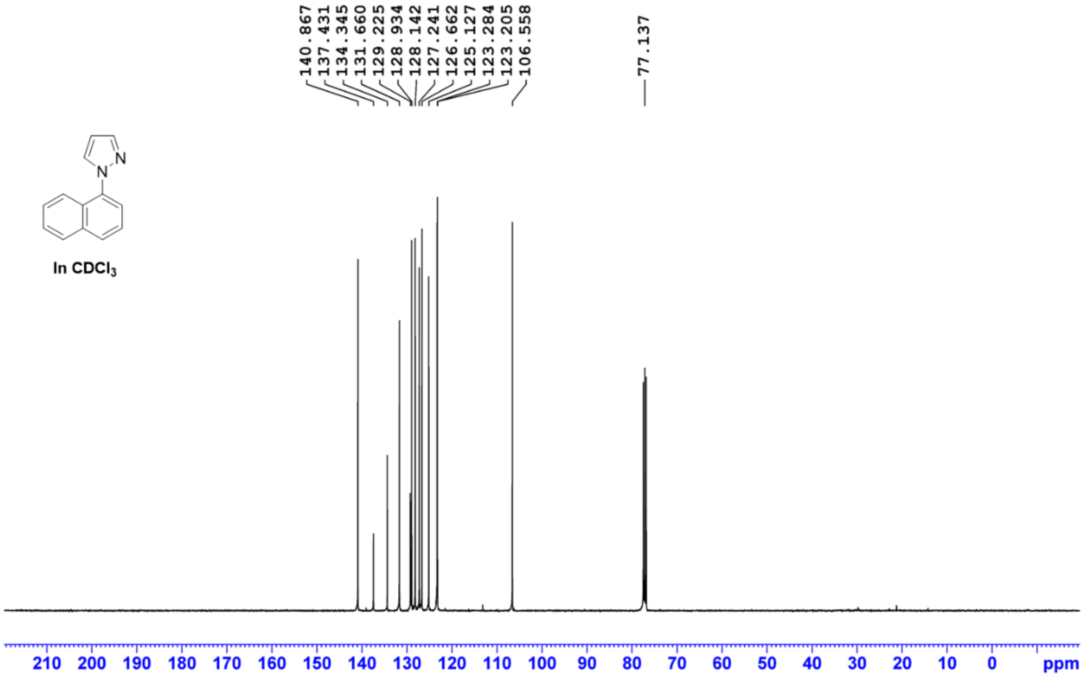

140.867
137.431
134.345
131.660
129.225
128.934
128.142
127.241
126.662
125.127
123.284
123.205
106.558
77.137
N
N
In $CDCl_3$
210
200
190
180
170
160
150
140
130
120
110
100
90
80
70
60
50
40
30
20
10
0
ppm

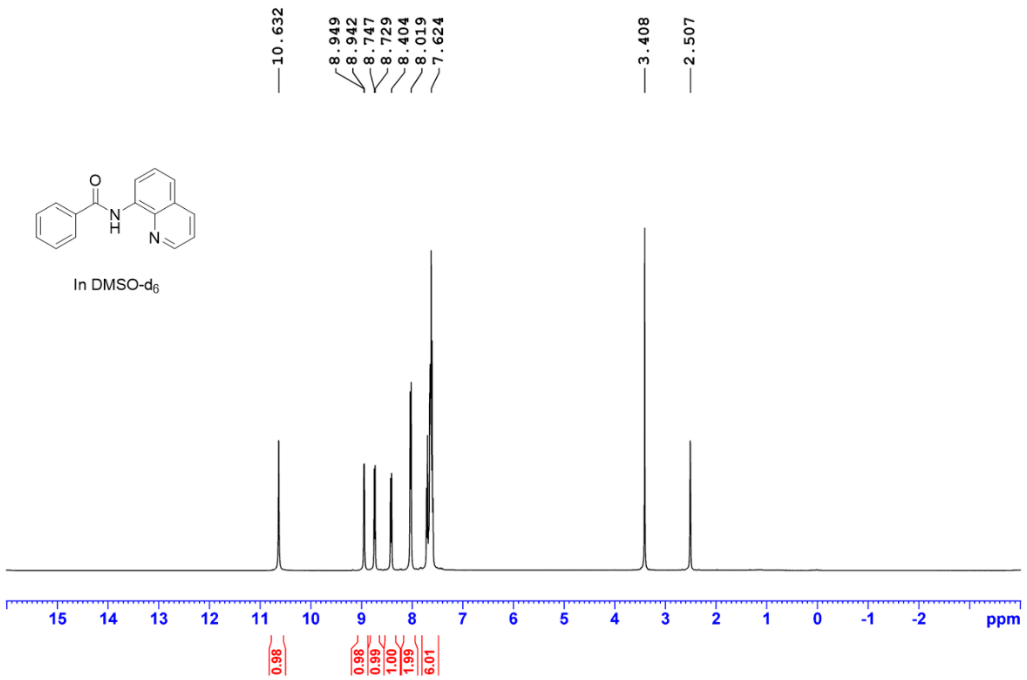
10.632
8.949
8.942
8.747
8.729
8.404
8.019
7.624
3.408
2.507
In DMSO-d6
15 14 13 12 11 10 9 8 7 6 5 4 3 2 1 0 -1 -2 ppm
0.98
0.98
0.99
1.00
1.99
6.01

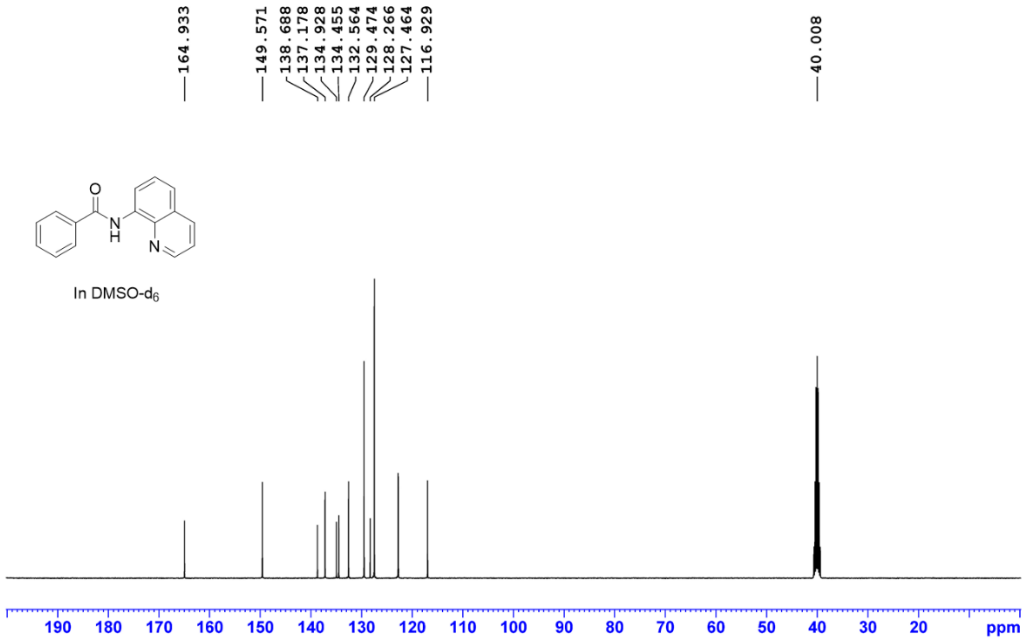
164.933
149.571
138.688
137.178
134.928
134.455
132.564
129.474
128.266
127.464
116.929
40.008
In DMSO-d6
190 180 170 160 150 140 130 120 110 100 90 80 70 60 50 40 30 20 ppm

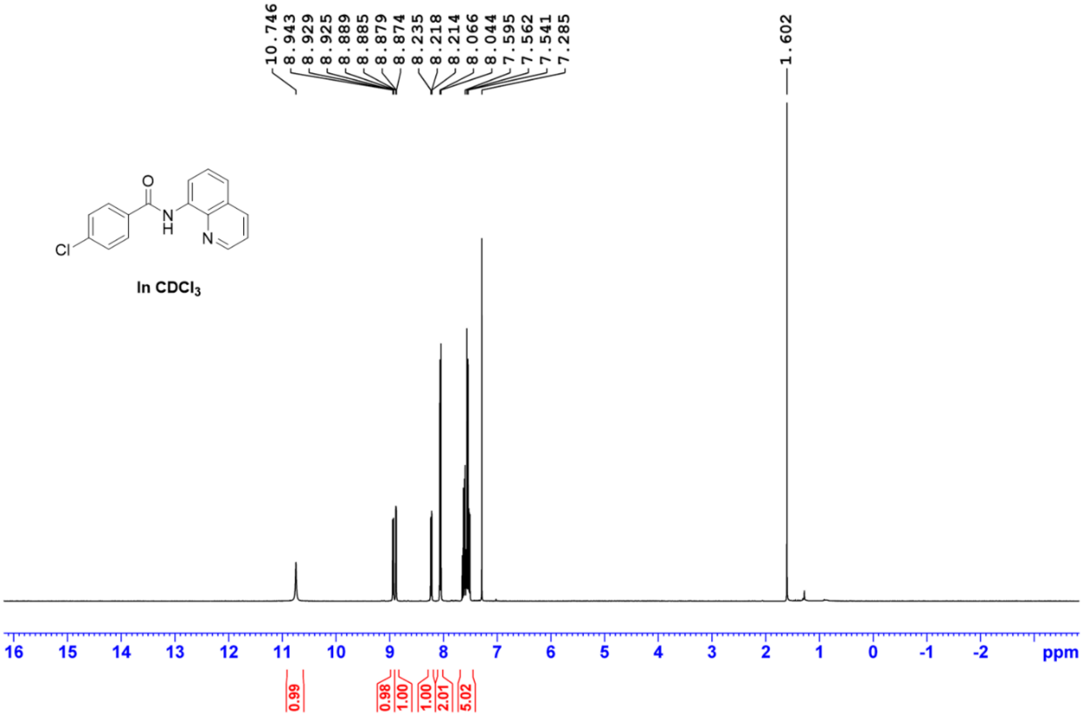
10.746
8.943
8.929
8.925
8.889
8.885
8.879
8.874
8.235
8.218
8.214
8.066
8.044
7.595
7.562
7.541
7.285
1.602
O
N
H
Cl
N
In CDCl3
16 15 14 13 12 11 10 9 8 7 6 5 4 3 2 1 0 -1 -2 ppm
0.99
0.98
1.00
1.00
2.01
5.02

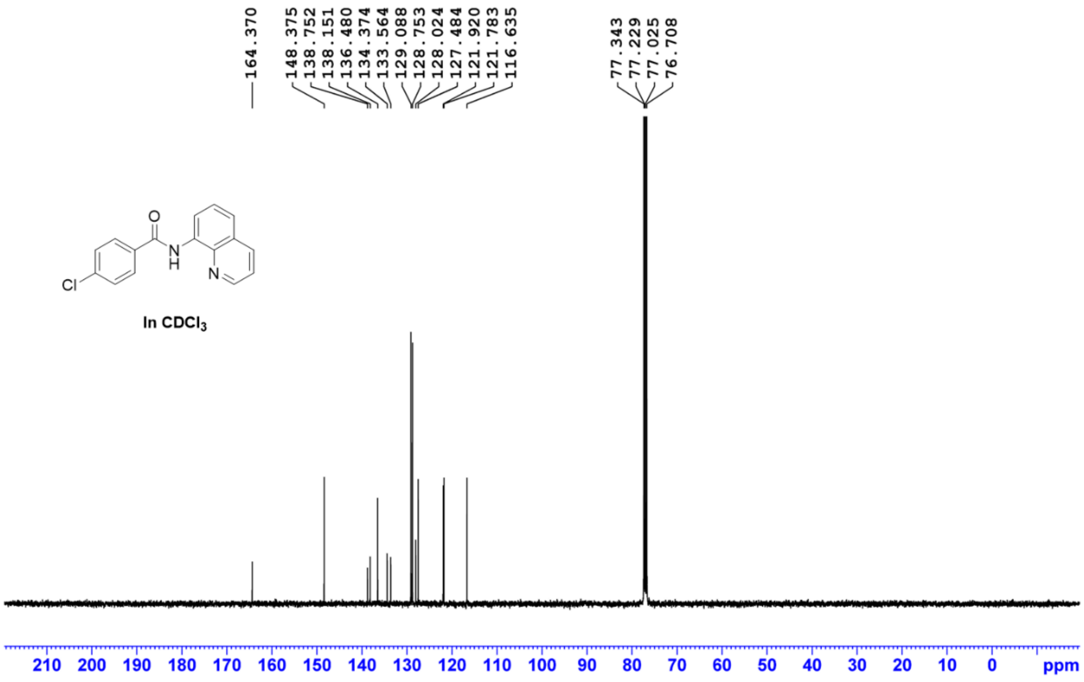
164.370
148.375
138.752
138.151
136.480
134.374
133.564
129.088
128.753
128.024
127.484
121.920
121.783
116.635
77.343
77.229
77.025
76.708
O
N
H
Cl
N
In CDCl3
210 200 190 180 170 160 150 140 130 120 110 100 90 80 70 60 50 40 30 20 10 0 ppm

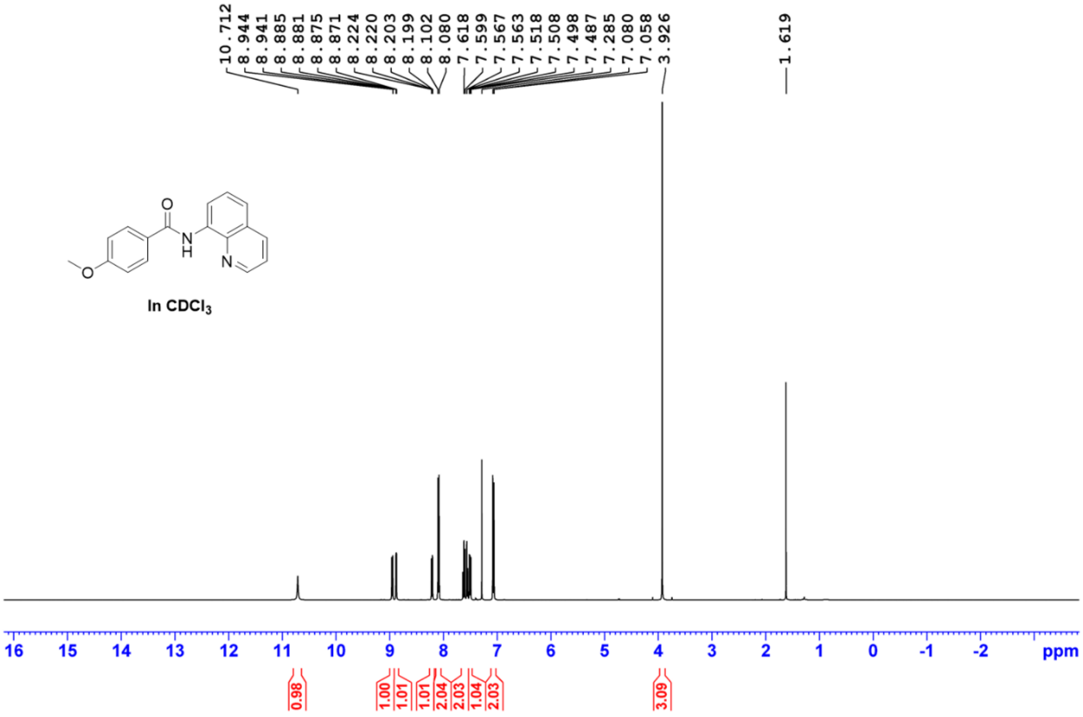

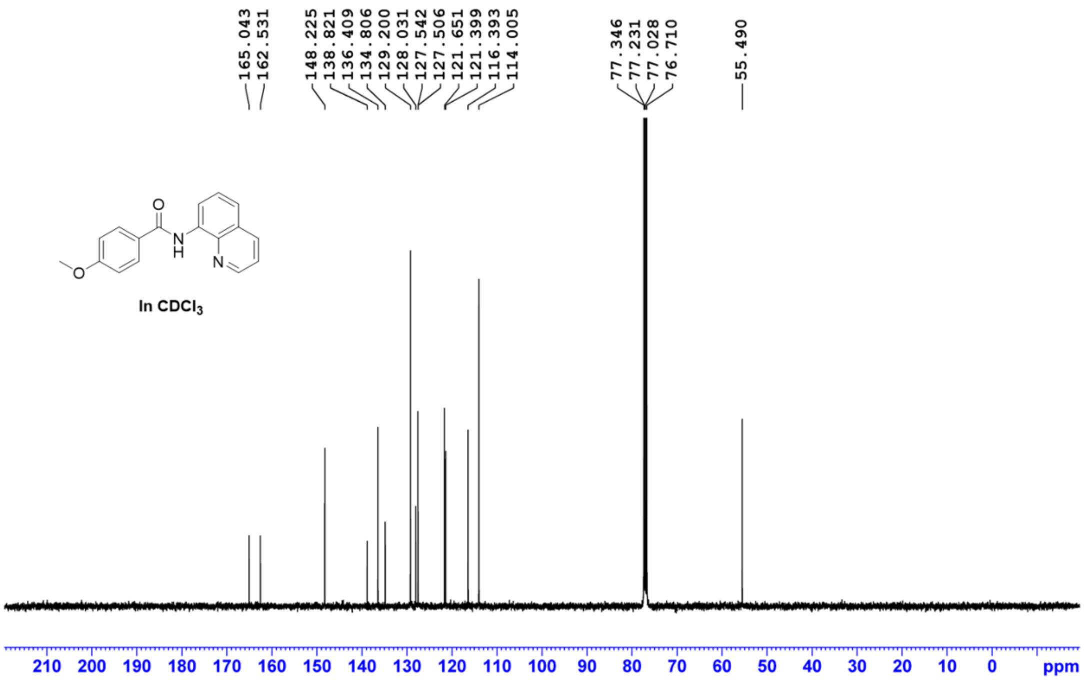

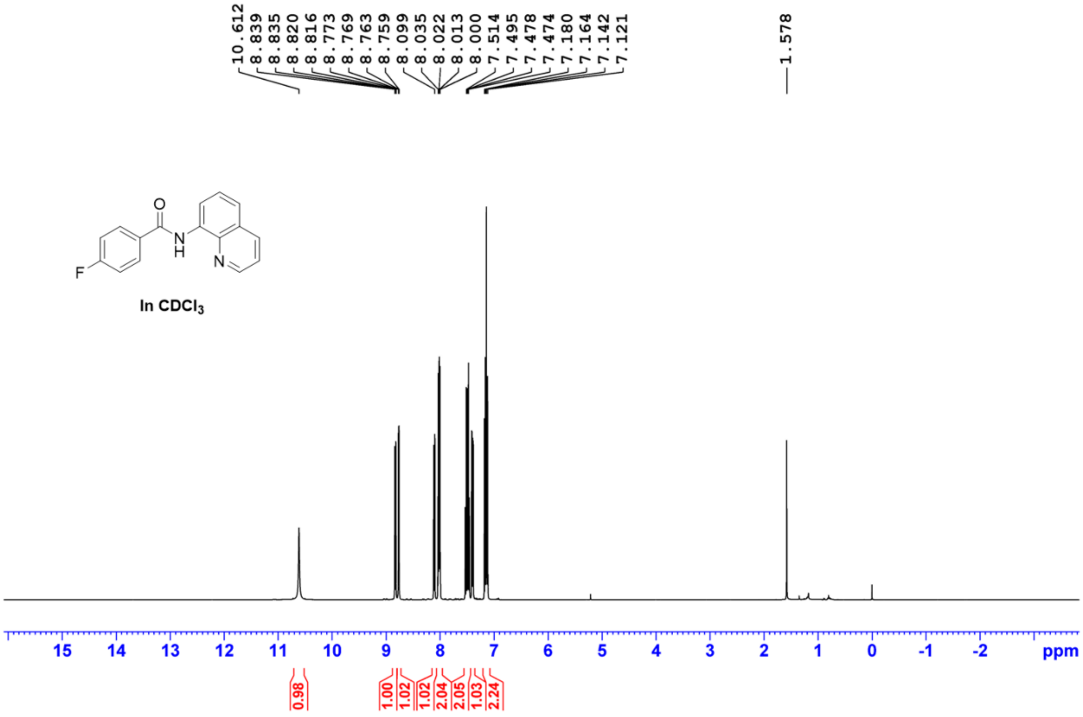
10.612
8.839
8.835
8.820
8.816
8.773
8.769
8.763
8.759
8.099
8.035
8.022
8.013
8.000
7.514
7.495
7.478
7.474
7.180
7.164
7.142
7.121
1.578
O
N
H
F
N
In CDCl3
15 14 13 12 11 10 9 8 7 6 5 4 3 2 1 0 -1 -2 ppm
0.98
1.00
1.02
1.02
2.04
2.05
1.03
2.24

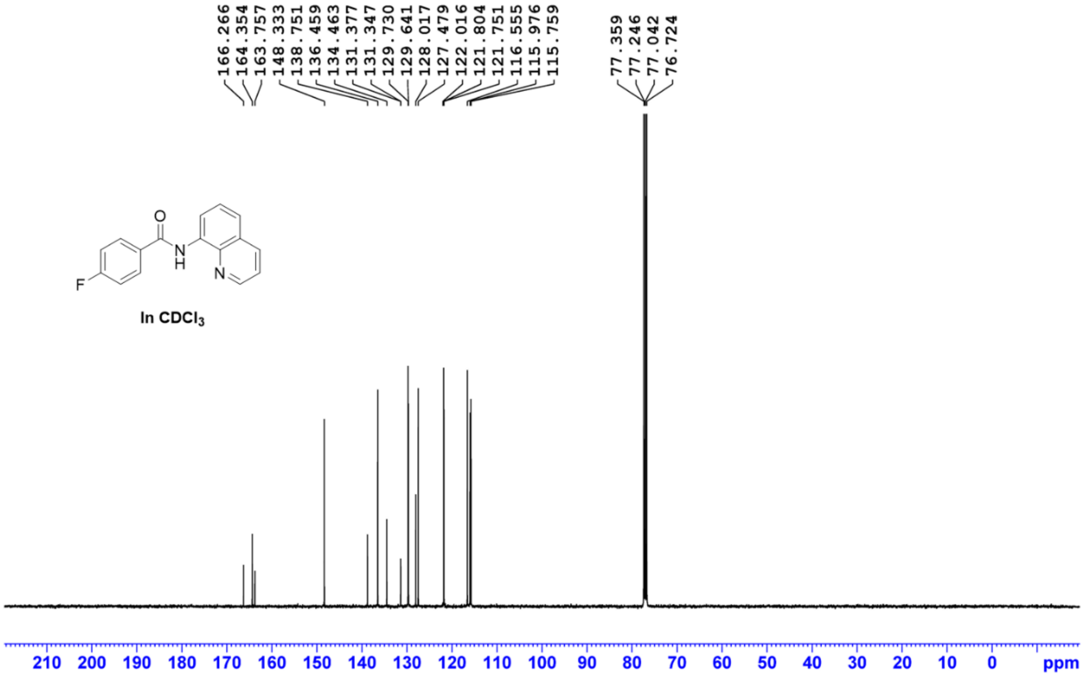
166.266
164.354
163.757
148.333
138.751
136.459
134.463
131.377
131.347
129.730
129.641
128.017
127.479
122.016
121.804
121.751
116.555
115.976
115.759
77.359
77.246
77.042
76.724
O
N
H
F
N
In CDCl3
210 200 190 180 170 160 150 140 130 120 110 100 90 80 70 60 50 40 30 20 10 0 ppm

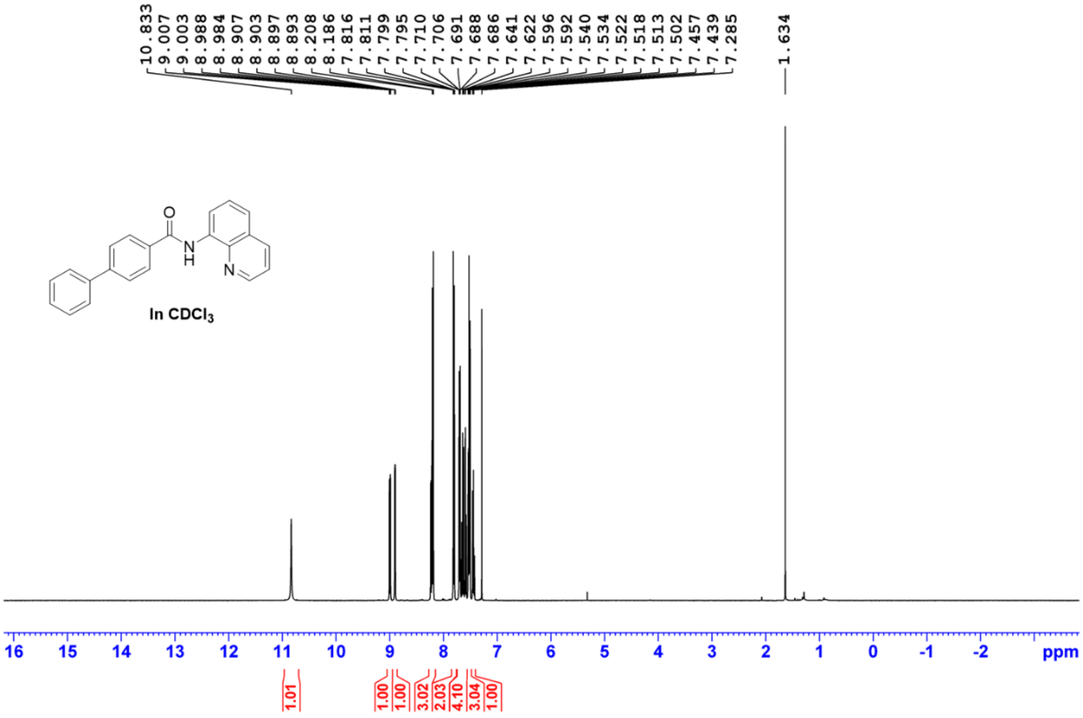
10.833
9.007
9.003
8.988
8.984
8.907
8.903
8.897
8.893
8.208
8.186
7.816
7.811
7.799
7.795
7.710
7.706
7.691
7.688
7.686
7.641
7.622
7.596
7.592
7.540
7.534
7.522
7.518
7.513
7.502
7.457
7.439
7.285
1.634
In CDCl3
1.01
1.00
1.00
3.02
2.03
4.10
3.04
1.00
ppm

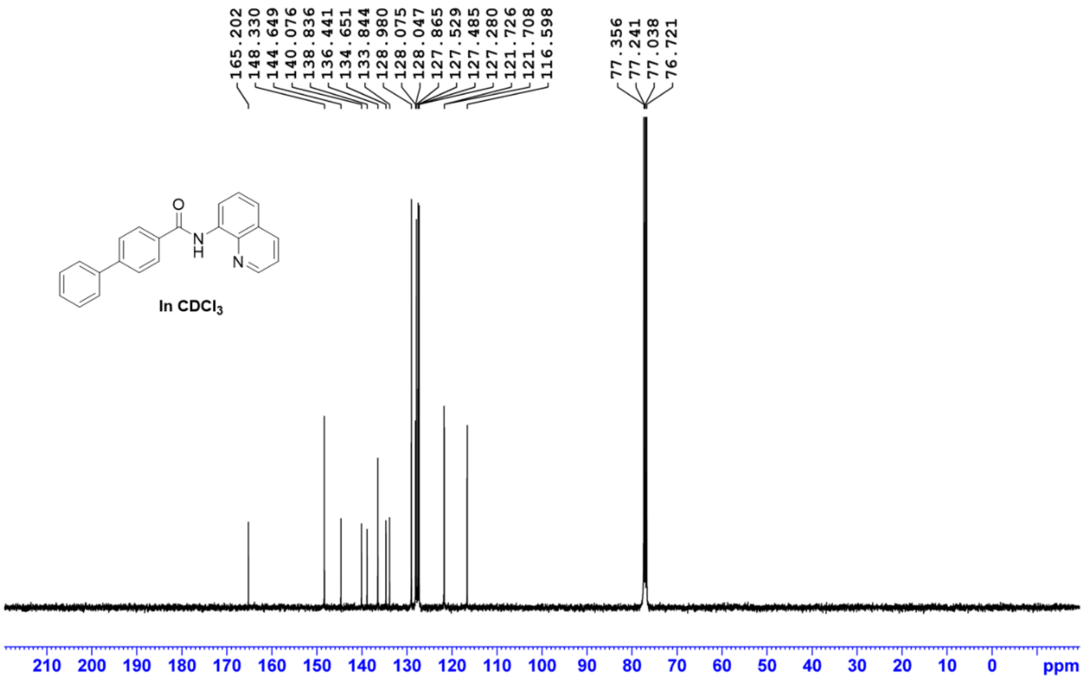
165.202
148.330
144.649
140.076
138.836
136.441
134.651
133.844
128.980
128.075
128.047
127.865
127.529
127.485
127.280
121.726
121.708
116.598
77.356
77.241
77.038
76.721
In CDCl3
ppm

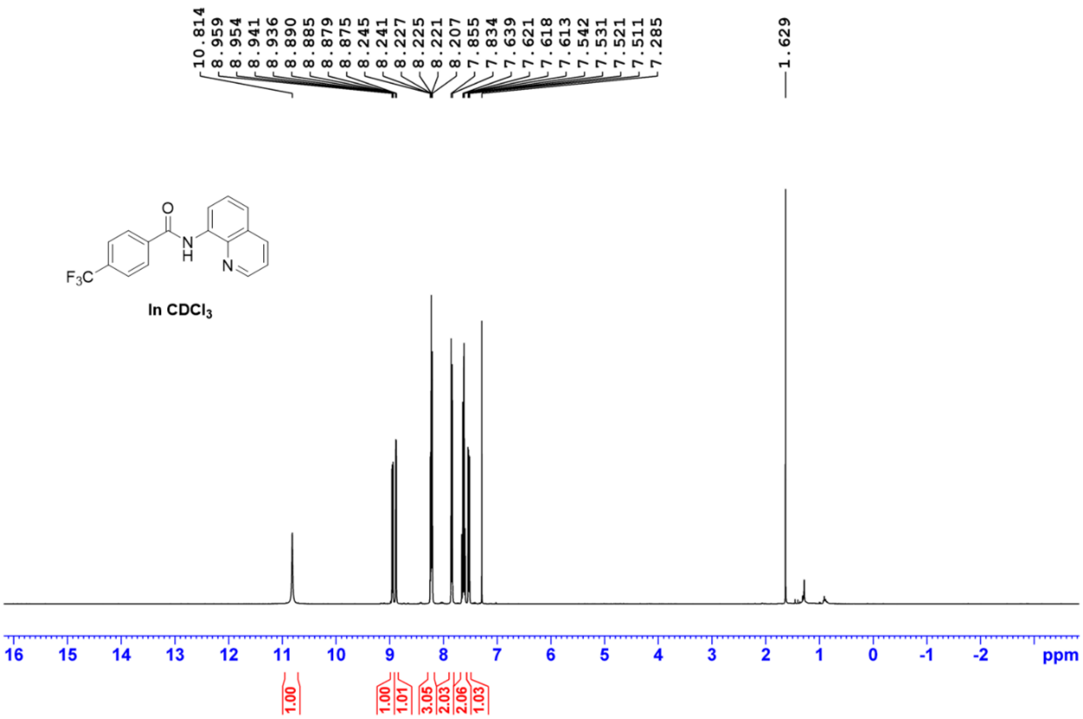
10.814
8.959
8.954
8.941
8.936
8.890
8.885
8.879
8.875
8.245
8.241
8.227
8.225
8.221
8.207
7.855
7.834
7.639
7.621
7.618
7.613
7.542
7.531
7.521
7.511
7.285
1.629
In CDCl3
16 15 14 13 12 11 10 9 8 7 6 5 4 3 2 1 0 -1 -2 ppm
1.00
1.00
1.01
3.05
2.03
2.06
1.03

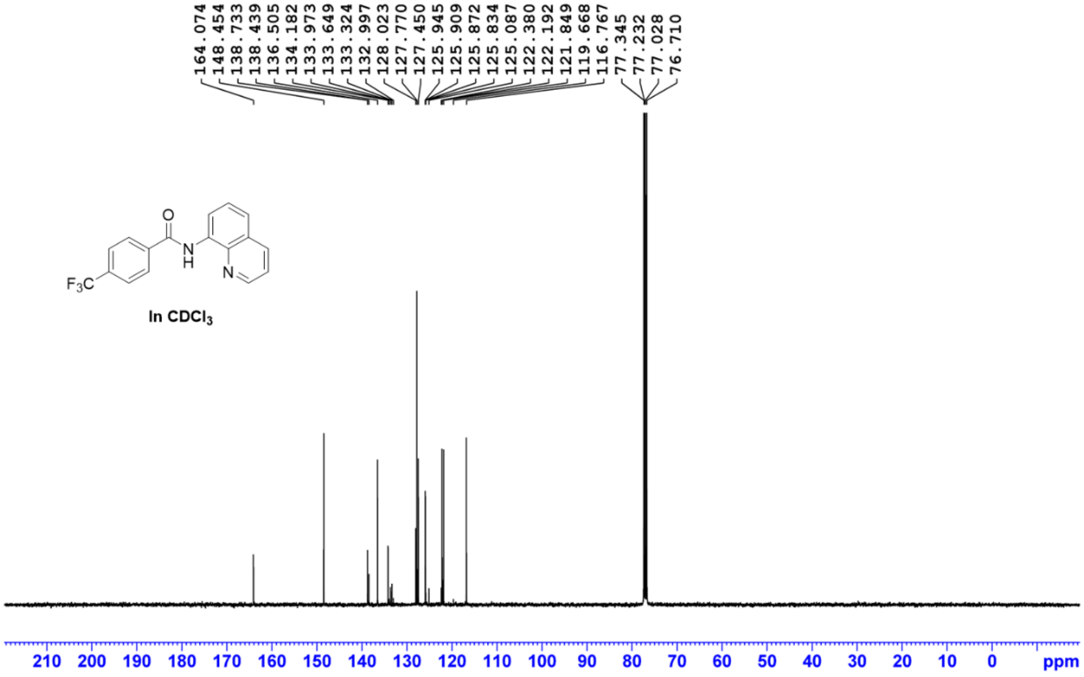
164.074
148.454
138.733
138.439
136.505
134.182
133.973
133.649
133.324
132.997
128.023
127.770
127.450
125.945
125.909
125.872
125.834
125.087
122.380
122.192
121.849
119.668
116.767
77.345
77.232
77.028
76.710
In CDCl3
210 200 190 180 170 160 150 140 130 120 110 100 90 80 70 60 50 40 30 20 10 0 ppm

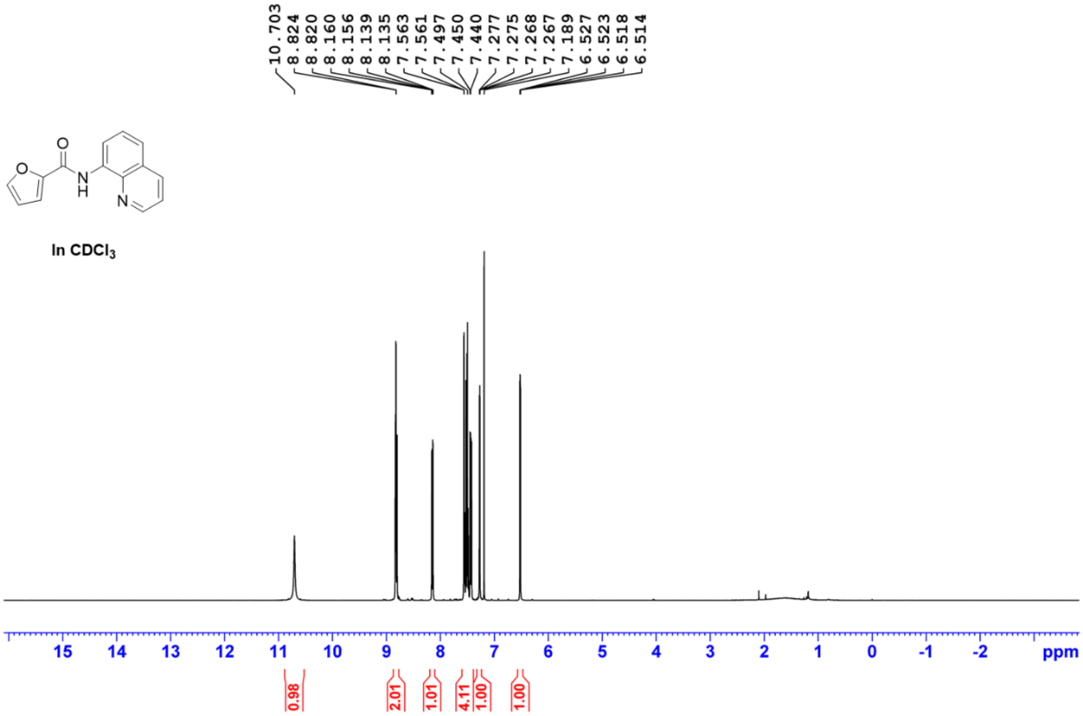
10.703
8.824
8.820
8.160
8.156
8.139
8.135
7.563
7.561
7.497
7.450
7.440
7.277
7.275
7.268
7.267
7.189
6.527
6.523
6.518
6.514
O
N
H
N
In $CDCl_3$
15 14 13 12 11 10 9 8 7 6 5 4 3 2 1 0 -1 -2 ppm
0.98
2.01
1.01
4.11
1.00
1.00

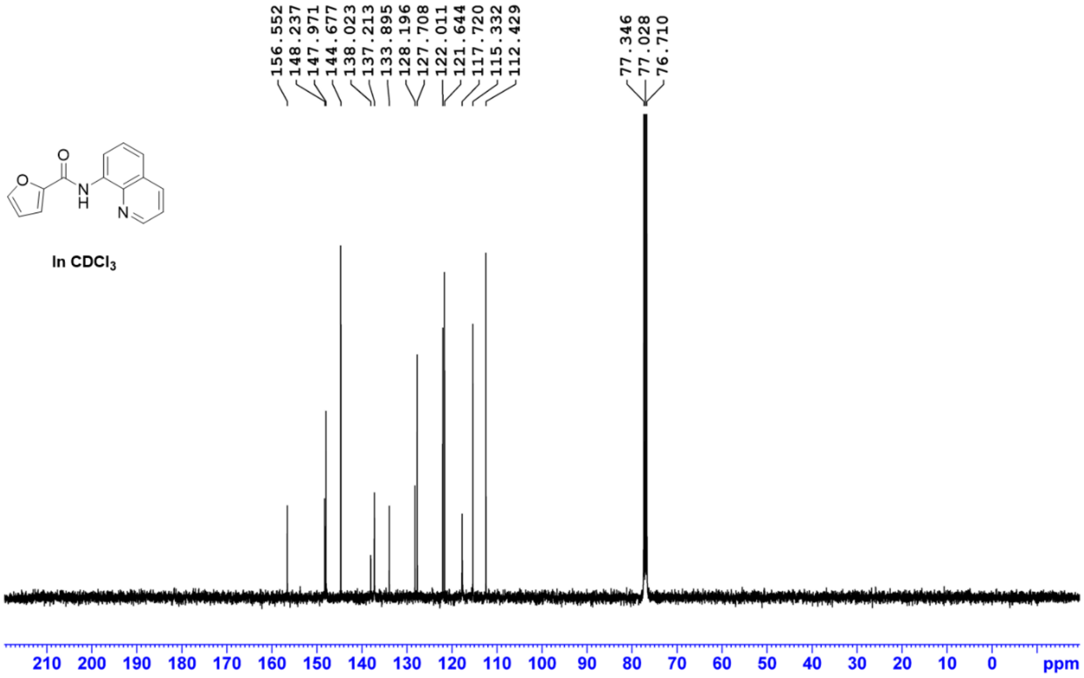
156.552
148.237
147.971
144.677
138.023
137.213
133.895
128.196
127.708
122.011
121.644
117.720
115.332
112.429
77.346
77.028
76.710
O
N
H
N
In $CDCl_3$
210 200 190 180 170 160 150 140 130 120 110 100 90 80 70 60 50 40 30 20 10 0 ppm

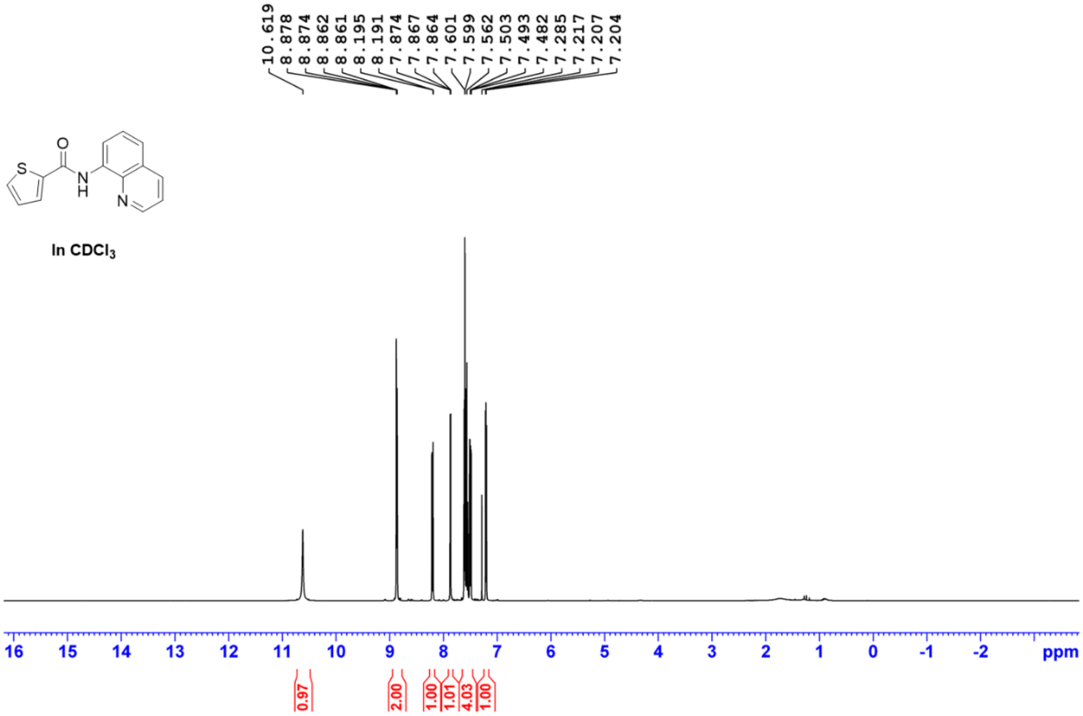
10.619
8.878
8.874
8.862
8.861
8.195
8.191
7.874
7.867
7.864
7.601
7.599
7.562
7.503
7.493
7.482
7.285
7.217
7.207
7.204
S
O
N
H
N
In $CDCl_3$
16 15 14 13 12 11 10 9 8 7 6 5 4 3 2 1 0 -1 -2 ppm
0.97
2.00
1.00
1.01
4.03
1.00

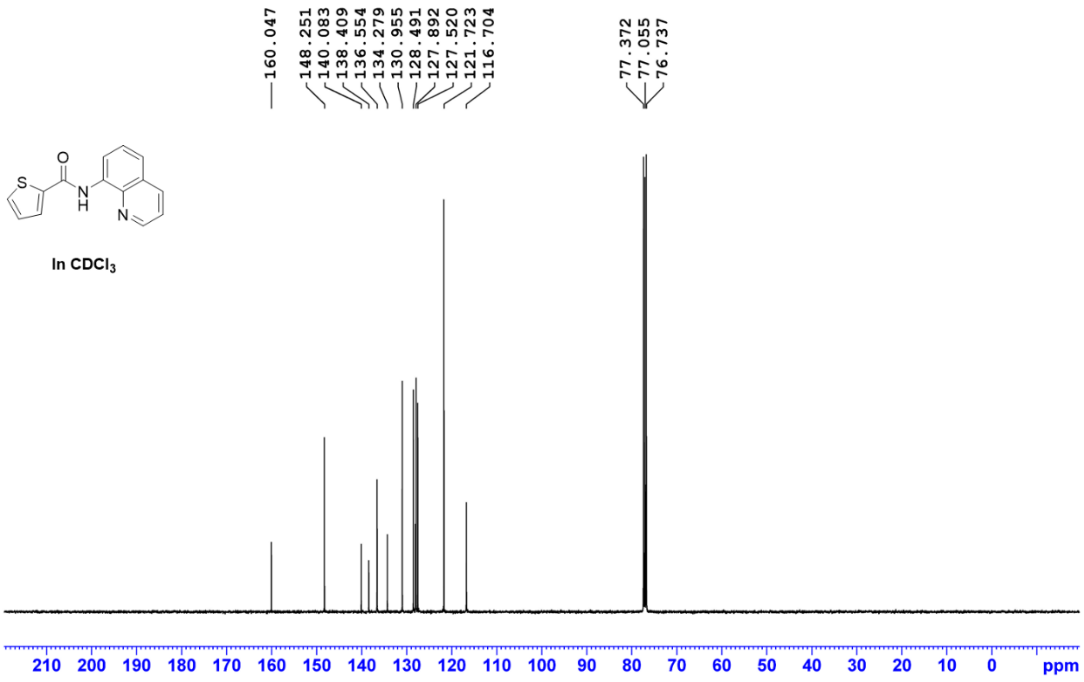
160.047
148.251
140.083
138.409
136.554
134.279
130.955
128.491
127.892
127.520
121.723
116.704
77.372
77.055
76.737
S
O
N
H
N
In $CDCl_3$
210 200 190 180 170 160 150 140 130 120 110 100 90 80 70 60 50 40 30 20 10 0 ppm

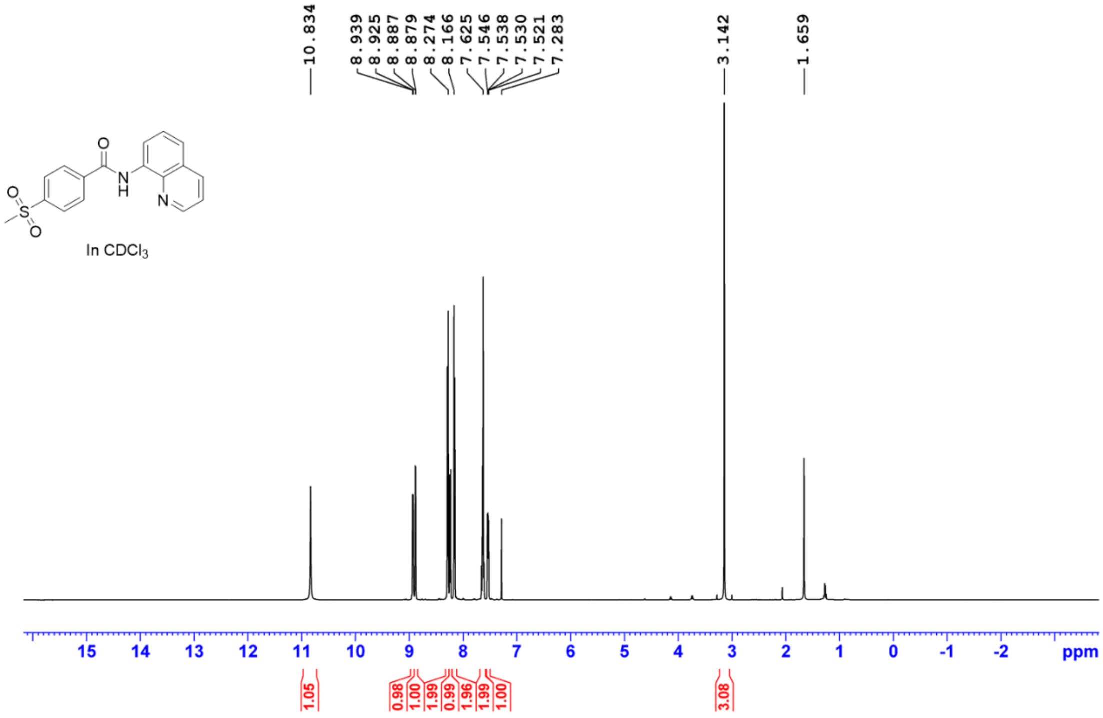
10.834
8.939
8.925
8.887
8.879
8.274
8.166
7.625
7.546
7.538
7.530
7.521
7.283
3.142
1.659
In CDCl3
ppm
1.05
0.98
1.00
1.99
0.99
1.96
1.99
1.00
3.08

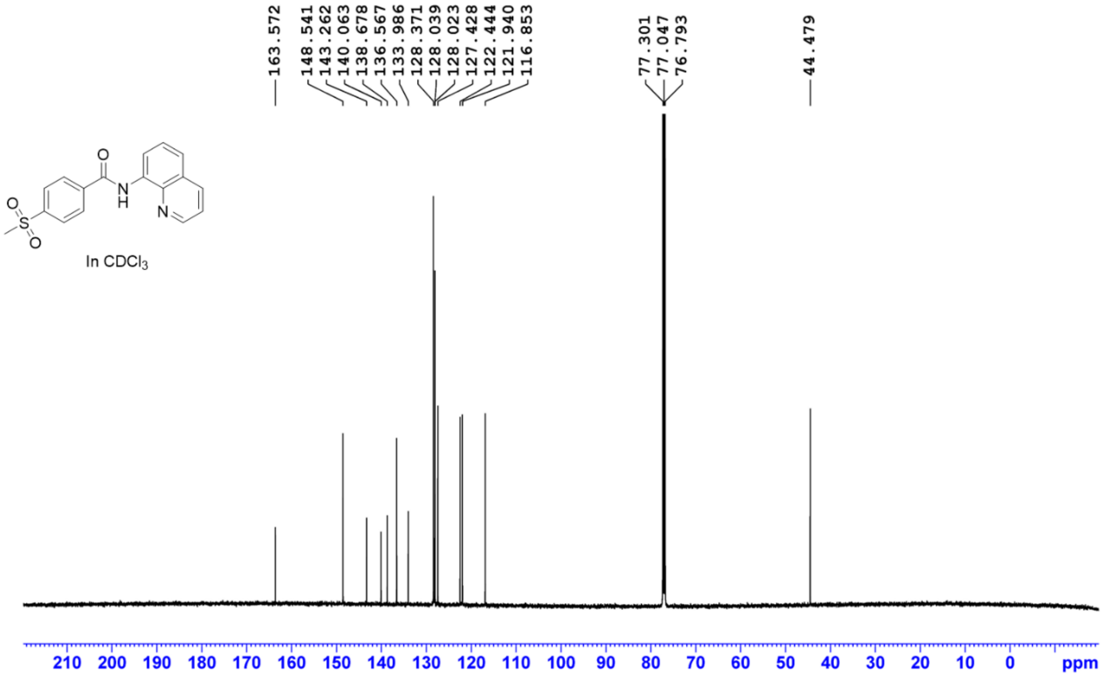
163.572
148.541
143.262
140.063
138.678
136.567
133.986
128.371
128.039
128.023
127.428
122.444
121.940
116.853
77.301
77.047
76.793
44.479
In CDCl3
ppm

Supplementary Information

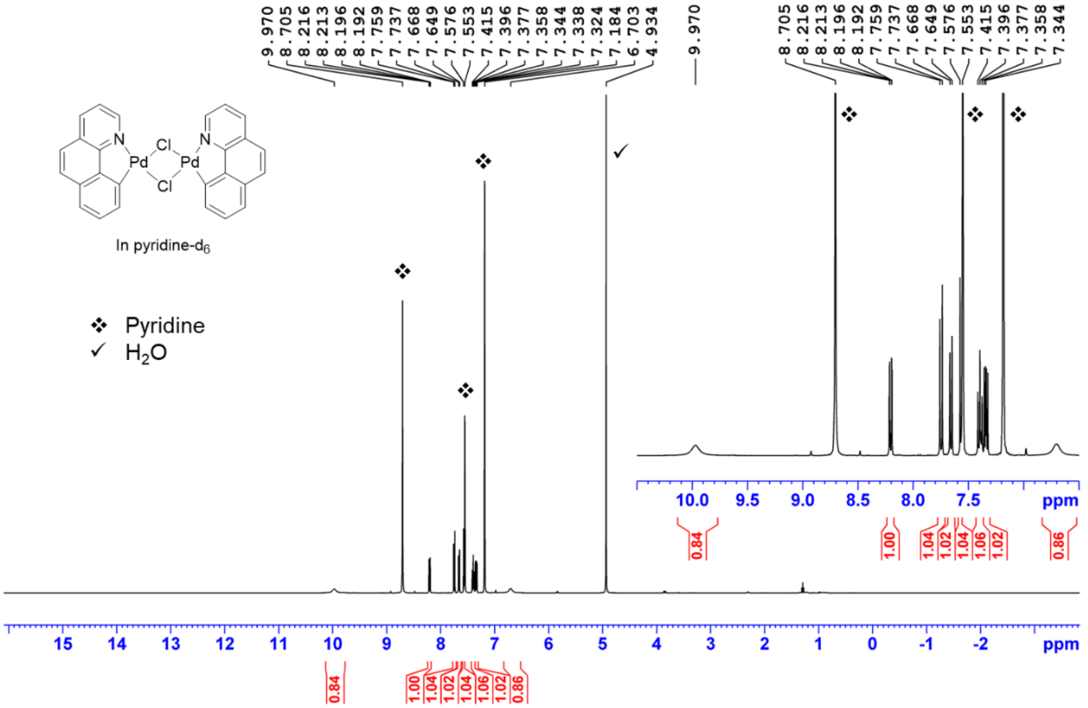

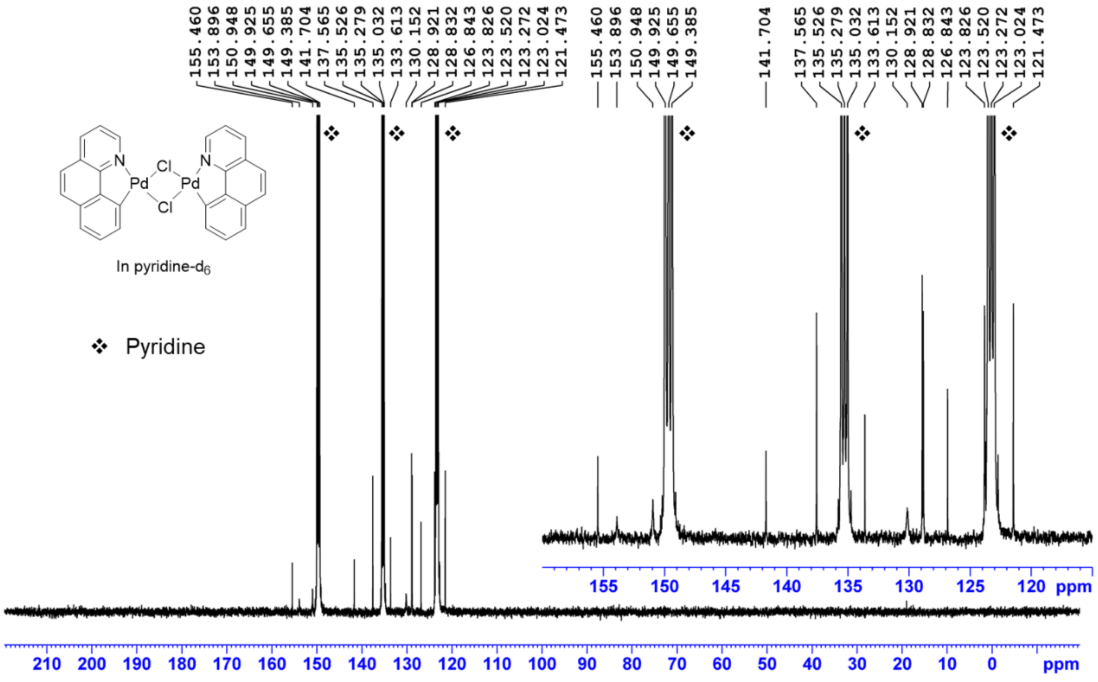

Supplementary Information

Product

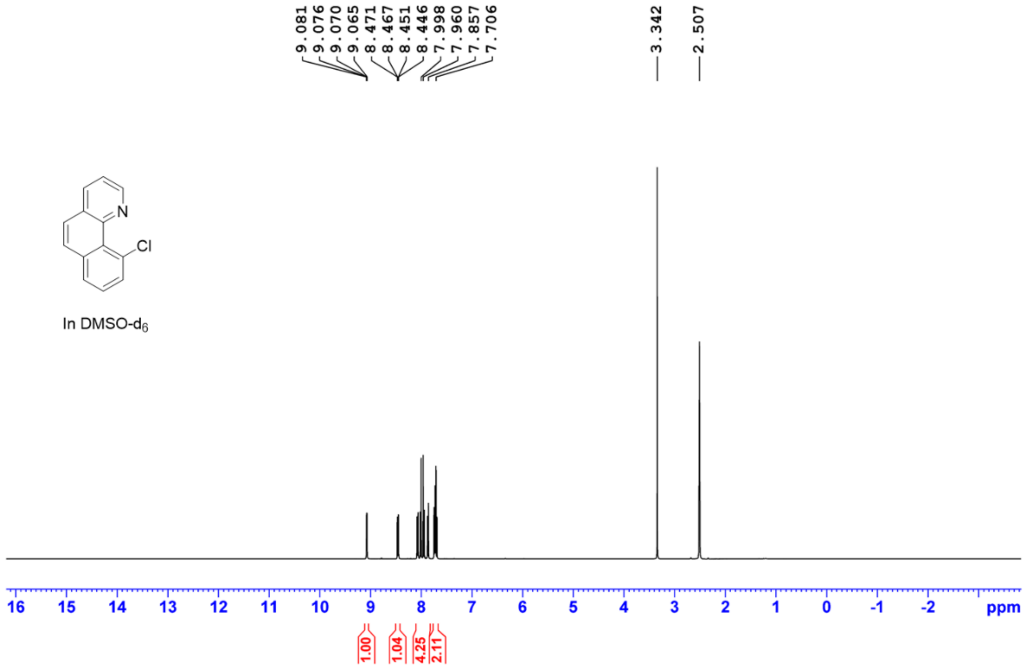

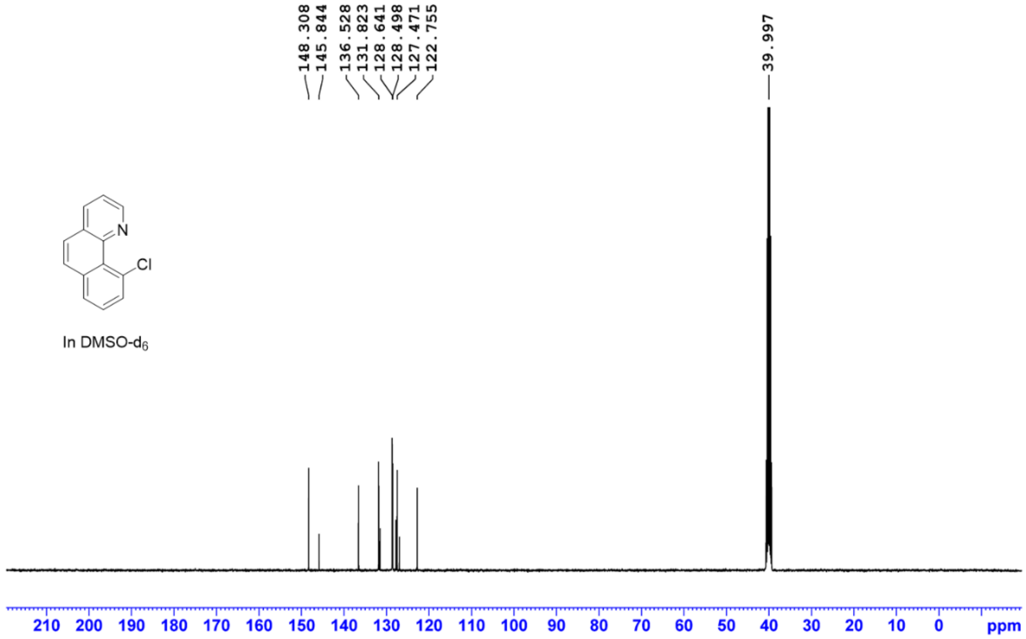

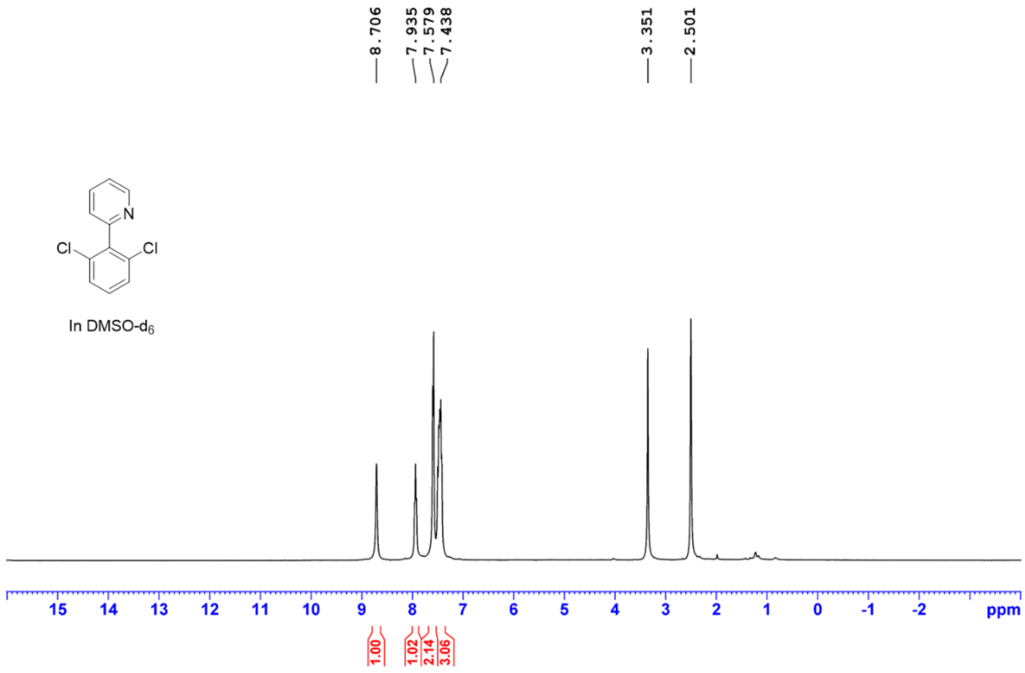

8.706
7.935
7.579
7.438
3.351
2.501
N
Cl
Cl
In DMSO-d6
15
14
13
12
11
10
9
8
7
6
5
4
3
2
1
0
-1
-2
ppm
1.00
1.02
2.14
3.06

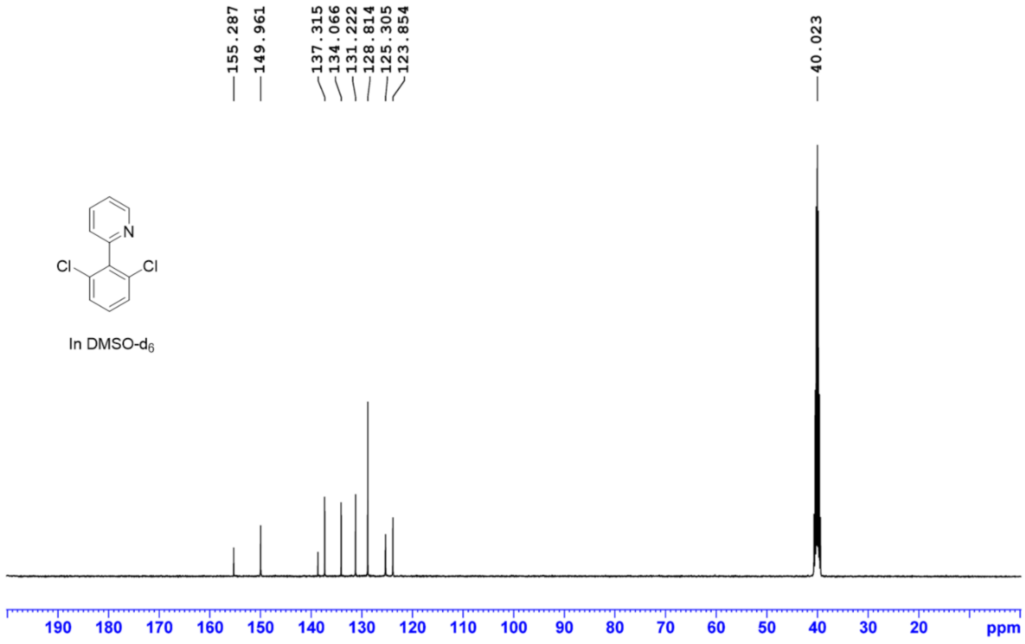

155.287
149.961
137.315
134.066
131.222
128.814
125.305
123.854
40.023
N
Cl
Cl
In DMSO-d6
190
180
170
160
150
140
130
120
110
100
90
80
70
60
50
40
30
20
ppm

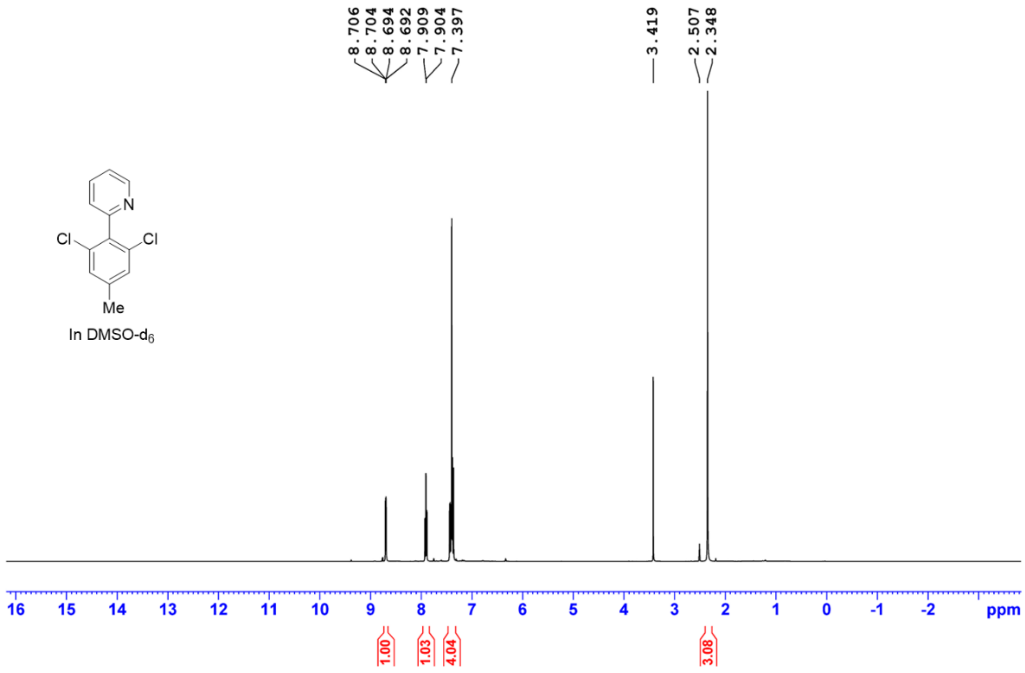
8.706
8.704
8.694
8.692
7.909
7.904
7.397
3.419
2.507
2.348
N
Cl
Cl
Me
In DMSO-d6
16 15 14 13 12 11 10 9 8 7 6 5 4 3 2 1 0 -1 -2 ppm
1.00
1.03
4.04
3.08

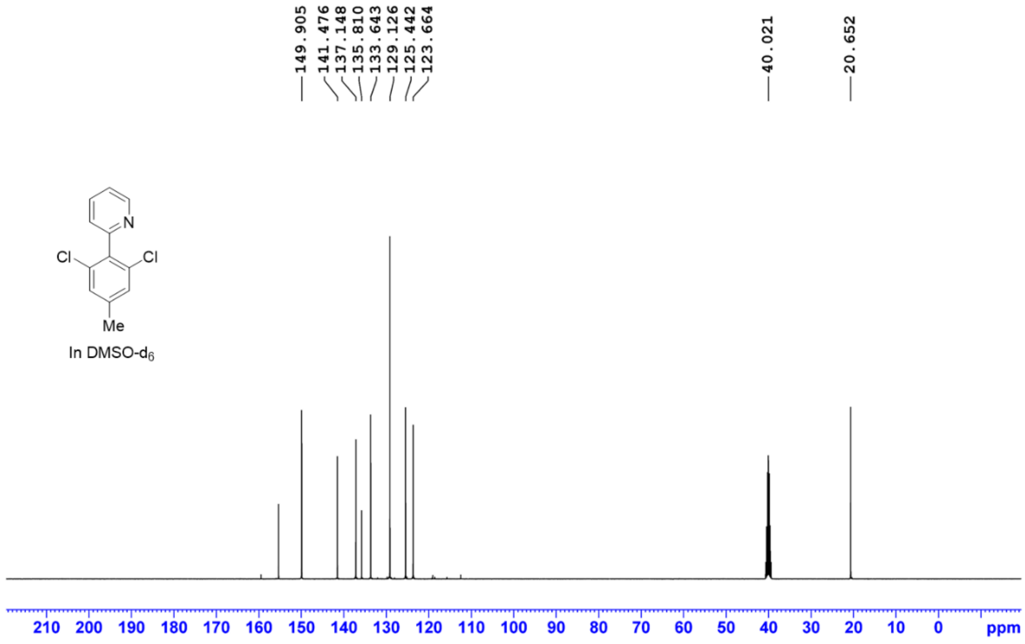
149.905
141.476
137.148
135.810
133.643
129.126
125.442
123.664
40.021
20.652
N
Cl
Cl
Me
In DMSO-d6
210 200 190 180 170 160 150 140 130 120 110 100 90 80 70 60 50 40 30 20 10 0 ppm

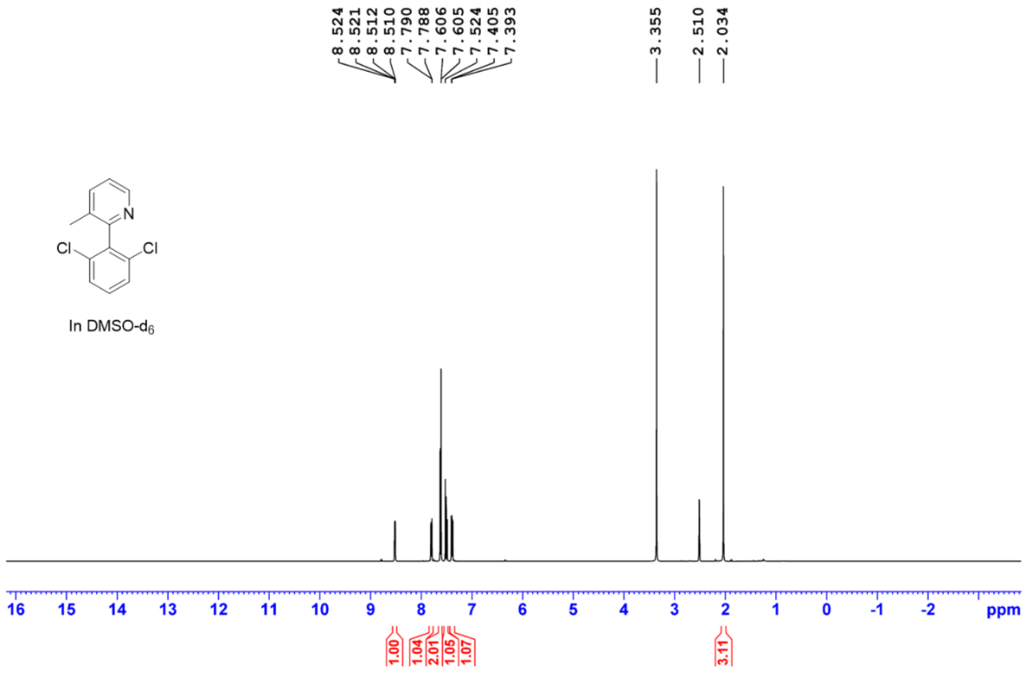
8.524
8.521
8.512
8.510
7.790
7.788
7.606
7.605
7.524
7.405
7.393
3.355
2.510
2.034
N
Cl
Cl
In DMSO-d6
16 15 14 13 12 11 10 9 8 7 6 5 4 3 2 1 0 -1 -2 ppm
1.00
1.04
2.01
1.05
1.07
3.11

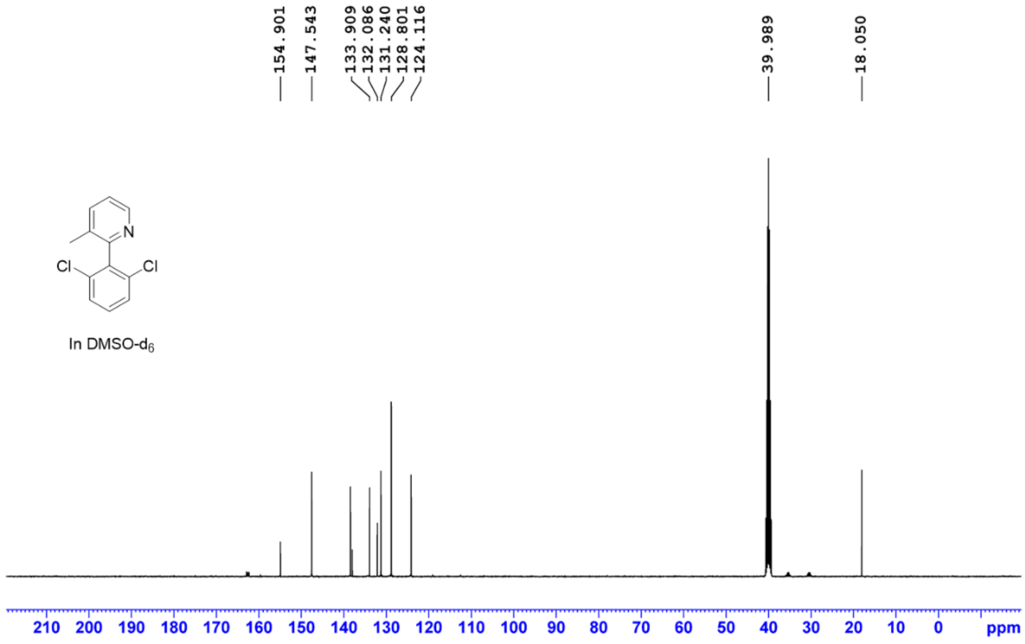
154.901
147.543
133.909
132.086
131.240
128.801
124.116
39.989
18.050
N
Cl
Cl
In DMSO-d6
210 200 190 180 170 160 150 140 130 120 110 100 90 80 70 60 50 40 30 20 10 0 ppm

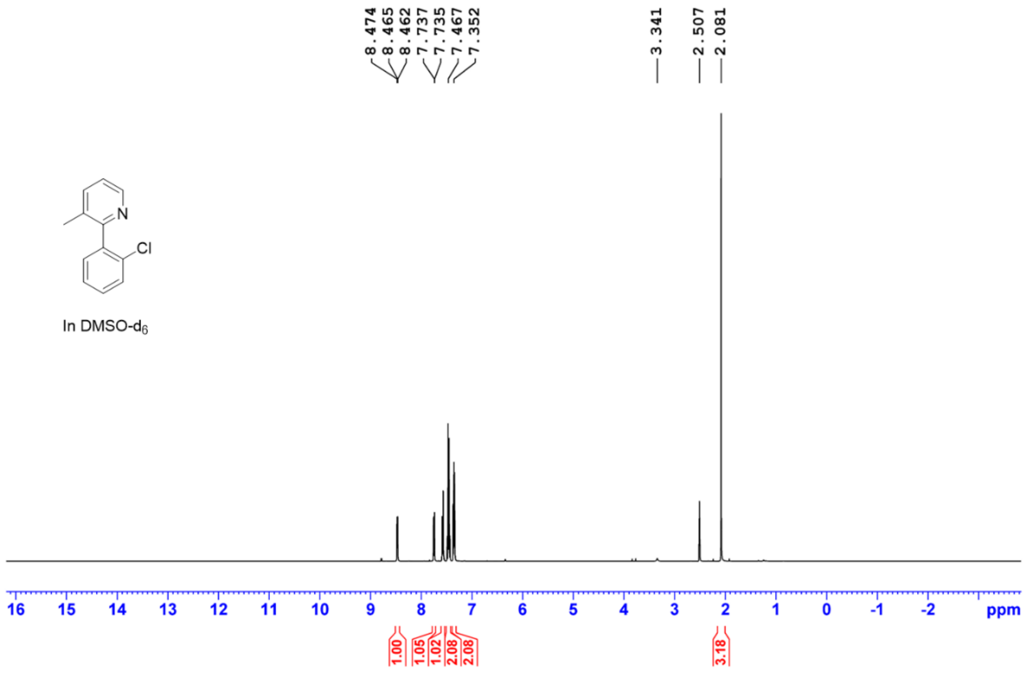
8.474
8.465
8.462
7.737
7.735
7.467
7.352
3.341
2.507
2.081
N
Cl
In DMSO-d6
16 15 14 13 12 11 10 9 8 7 6 5 4 3 2 1 0 -1 -2 ppm
1.00
1.05
1.02
2.08
2.08
3.18

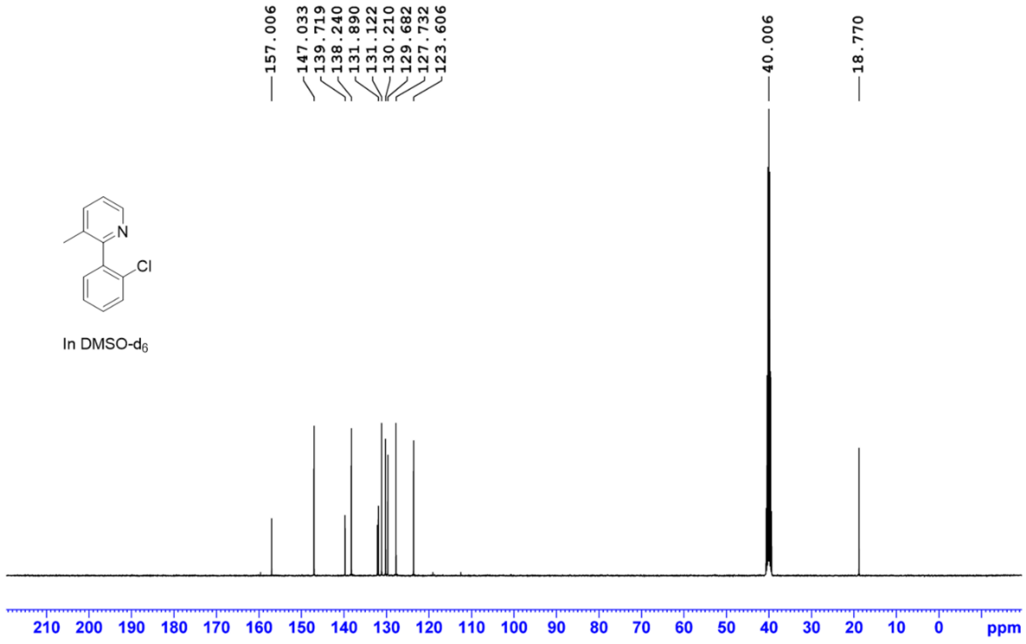
157.006
147.033
139.719
138.240
131.890
131.122
130.210
129.682
127.732
123.606
40.006
18.770
N
Cl
In DMSO-d6
210 200 190 180 170 160 150 140 130 120 110 100 90 80 70 60 50 40 30 20 10 0 ppm

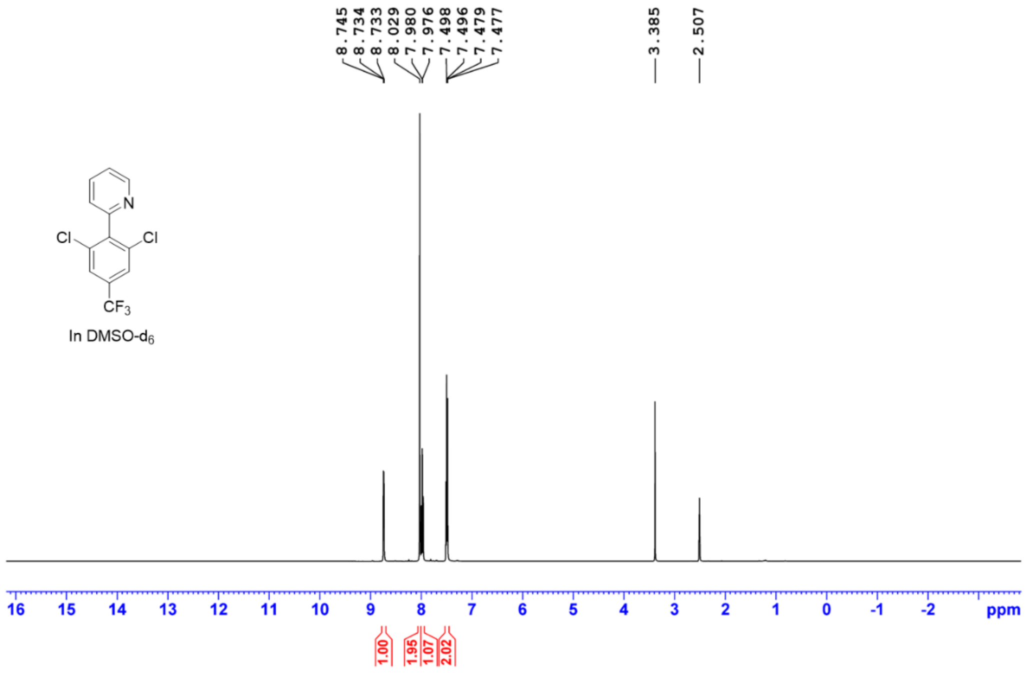
8.745
8.734
8.733
8.029
7.980
7.976
7.498
7.496
7.479
7.477
3.385
2.507
Cl
Cl
N
CF3
In DMSO-d6
16 15 14 13 12 11 10 9 8 7 6 5 4 3 2 1 0 -1 -2 ppm
1.00
1.95
1.07
2.02

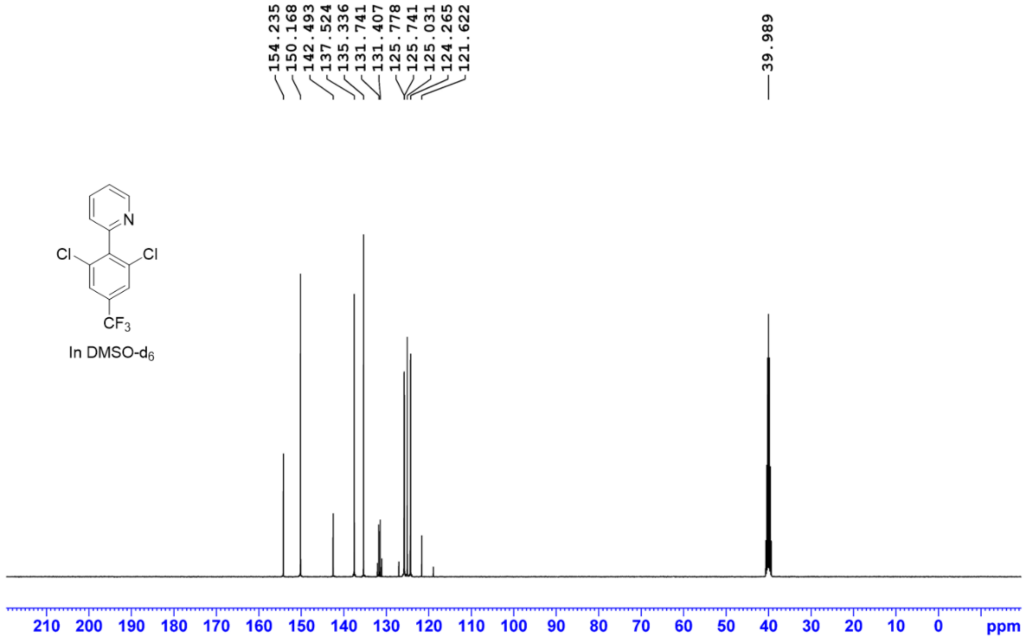
154.235
150.168
142.493
137.524
135.336
131.741
131.407
125.778
125.741
125.031
124.265
121.622
39.989
Cl
Cl
N
CF3
In DMSO-d6
210 200 190 180 170 160 150 140 130 120 110 100 90 80 70 60 50 40 30 20 10 0 ppm

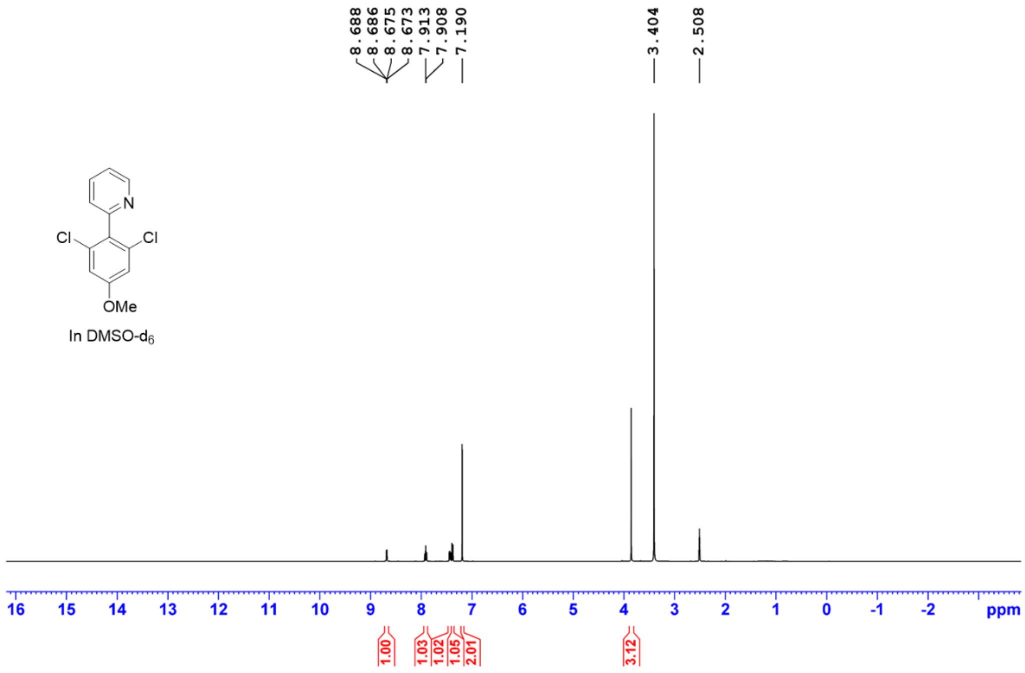
8.688
8.686
8.675
8.673
7.913
7.908
7.190
3.404
2.508
N
Cl
Cl
OMe
In DMSO-d6
16 15 14 13 12 11 10 9 8 7 6 5 4 3 2 1 0 -1 -2 ppm
1.00
1.03
1.02
1.05
2.01
3.12

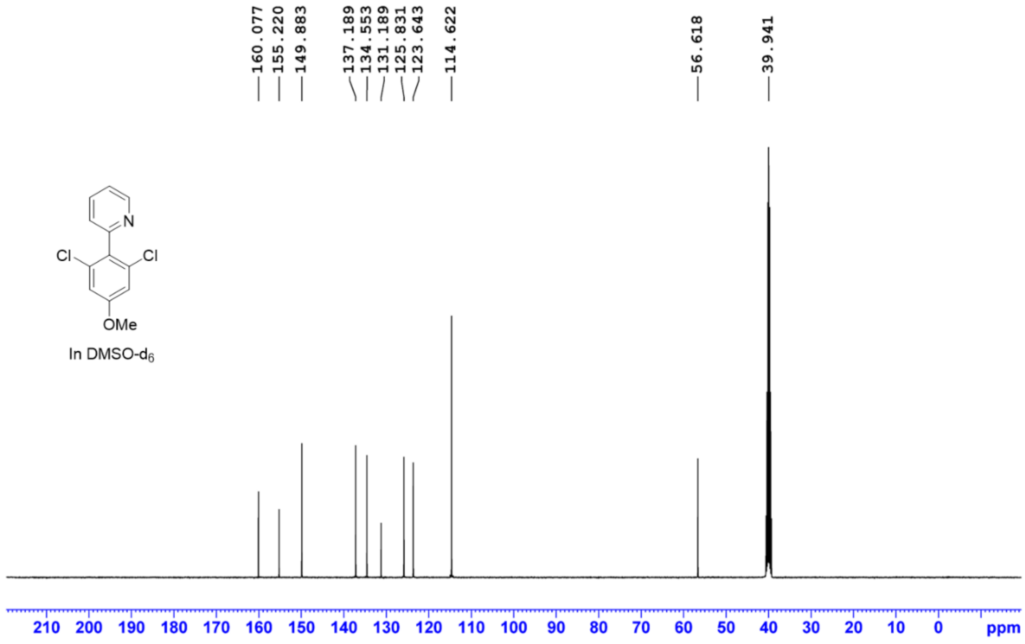
160.077
155.220
149.883
137.189
134.553
131.189
125.831
123.643
114.622
56.618
39.941
N
Cl
Cl
OMe
In DMSO-d6
210 200 190 180 170 160 150 140 130 120 110 100 90 80 70 60 50 40 30 20 10 0 ppm

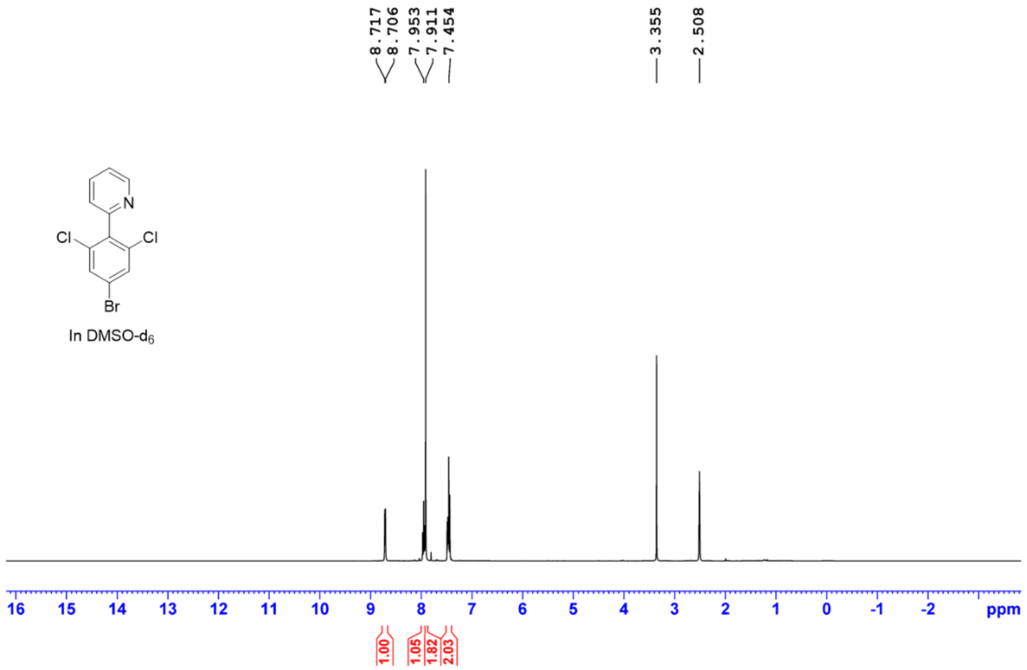
8.717
8.706
7.953
7.911
7.454
3.355
2.508
Cl
Cl
N
Br
In DMSO-d6
16 15 14 13 12 11 10 9 8 7 6 5 4 3 2 1 0 -1 -2 ppm
1.00
1.05
1.82
2.03

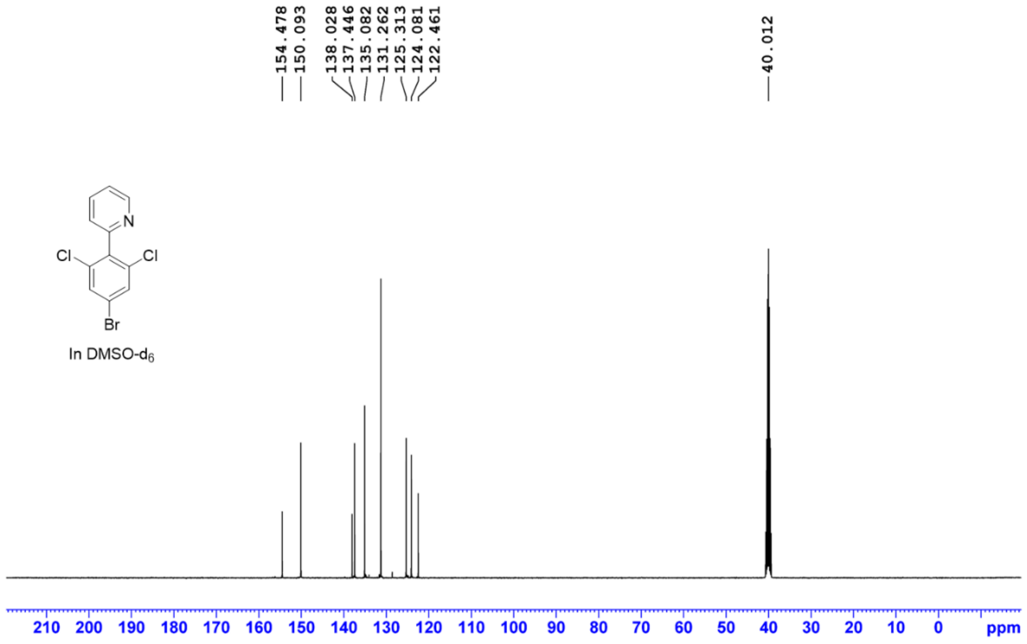
154.478
150.093
138.028
137.446
135.082
131.262
125.313
124.081
122.461
40.012
Cl
Cl
N
Br
In DMSO-d6
210 200 190 180 170 160 150 140 130 120 110 100 90 80 70 60 50 40 30 20 10 0 ppm

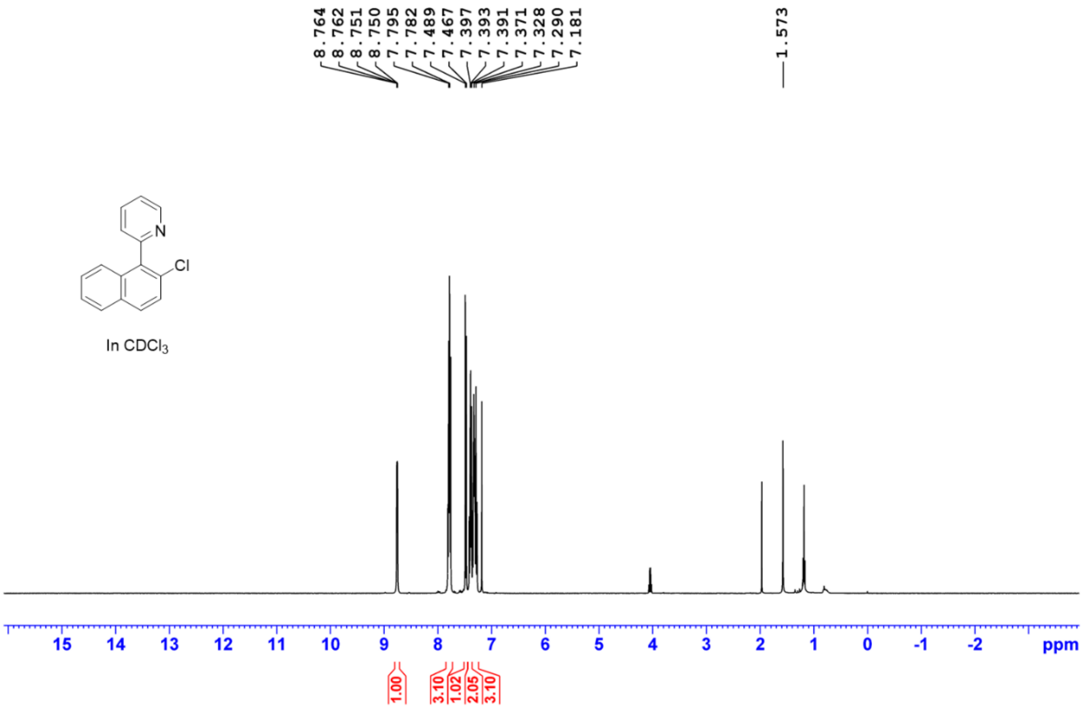
8.764
8.762
8.751
8.750
7.795
7.782
7.489
7.467
7.397
7.393
7.391
7.371
7.328
7.290
7.181
1.573
N
Cl
In $CDCl_3$
15 14 13 12 11 10 9 8 7 6 5 4 3 2 1 0 -1 -2 ppm
1.00
3.10
1.02
2.05
3.10

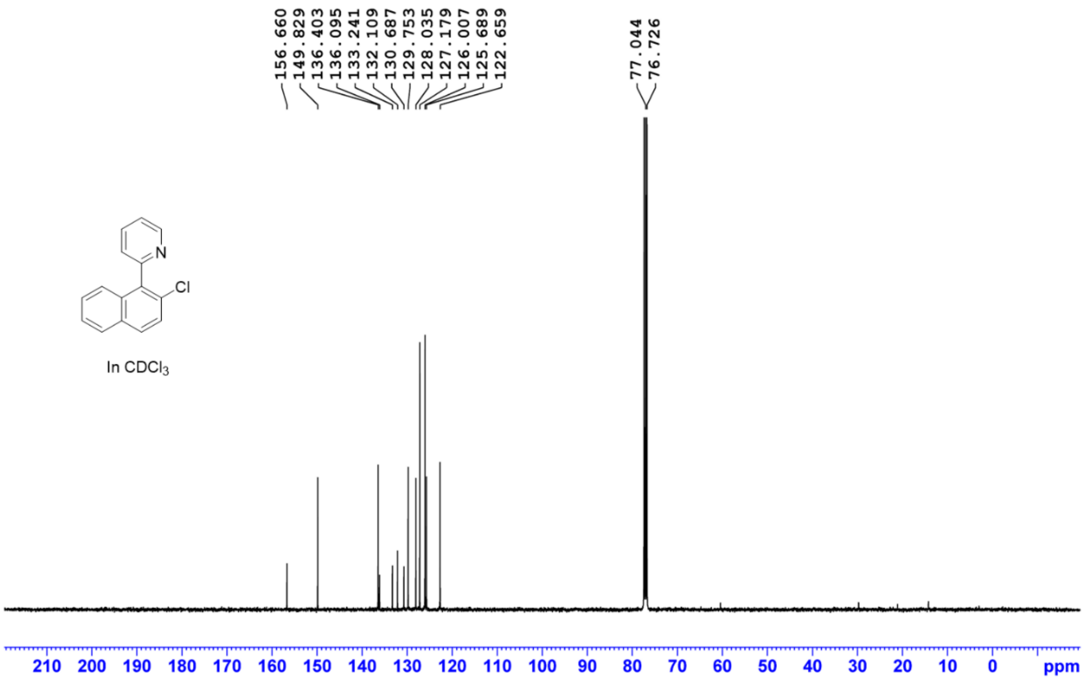
156.660
149.829
136.403
136.095
133.241
132.109
130.687
129.753
128.035
127.179
126.007
125.689
122.659
77.044
76.726
N
Cl
In $CDCl_3$
210 200 190 180 170 160 150 140 130 120 110 100 90 80 70 60 50 40 30 20 10 0 ppm

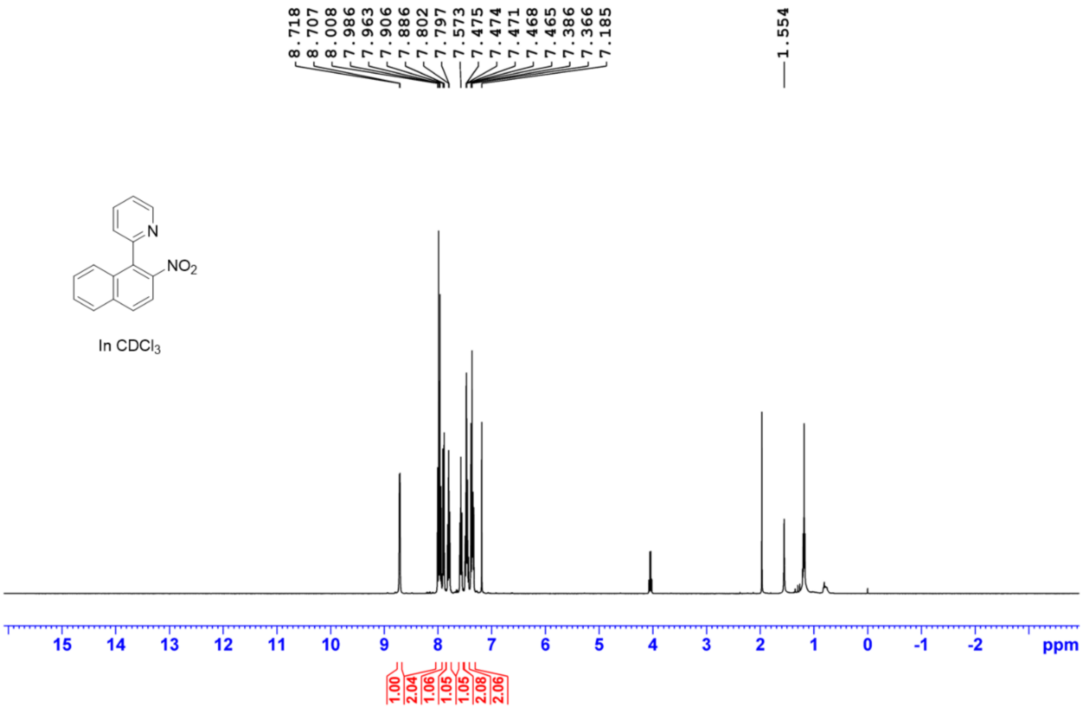
8.718
8.707
8.008
7.986
7.963
7.906
7.886
7.802
7.797
7.573
7.475
7.474
7.471
7.468
7.465
7.386
7.366
7.185
1.554
NO2
N
In CDCl3
15 14 13 12 11 10 9 8 7 6 5 4 3 2 1 0 -1 -2 ppm
1.00
2.04
1.06
1.05
1.05
2.08
2.06

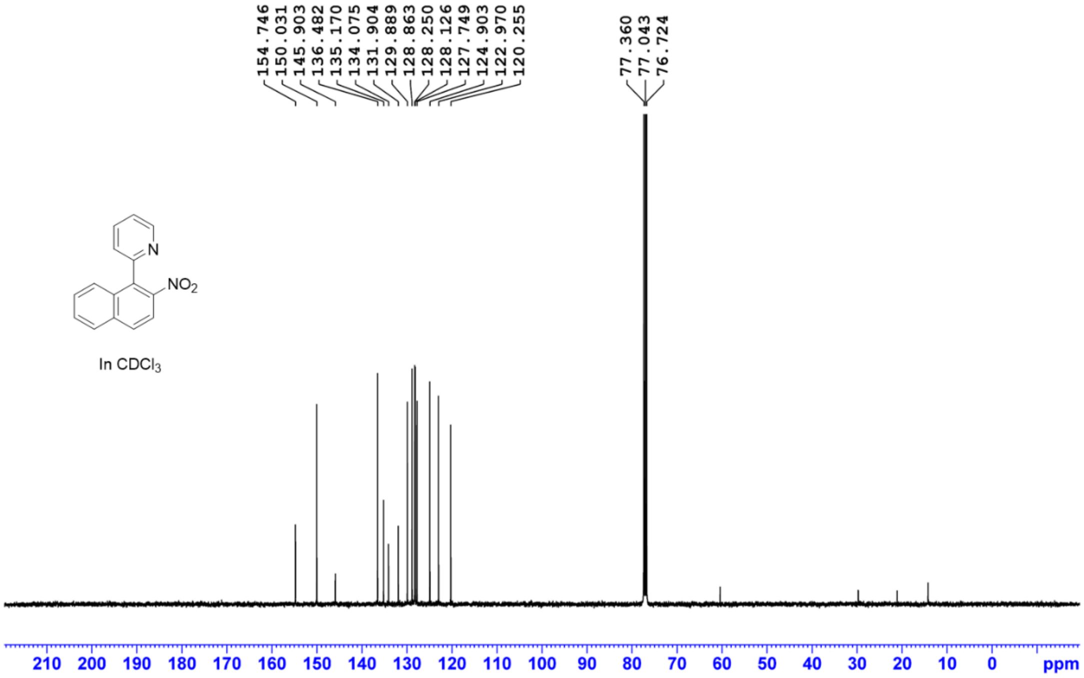
154.746
150.031
145.903
136.482
135.170
134.075
131.904
129.889
128.863
128.250
128.126
127.749
124.903
122.970
120.255
77.360
77.043
76.724
NO2
N
In CDCl3
210 200 190 180 170 160 150 140 130 120 110 100 90 80 70 60 50 40 30 20 10 0 ppm

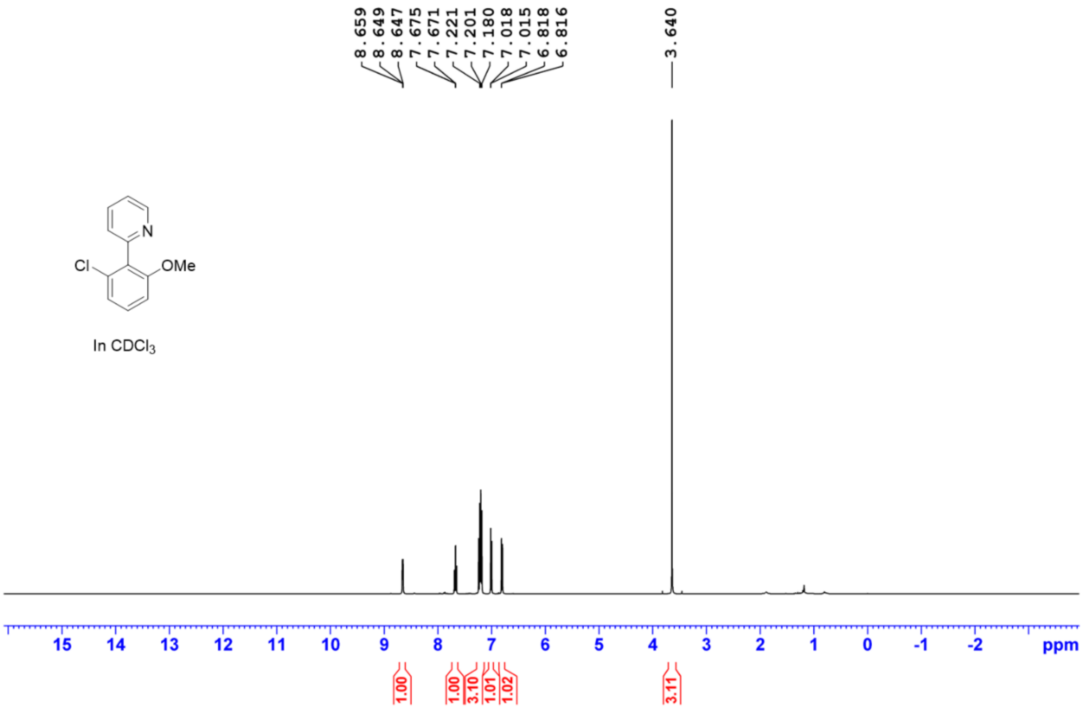
8.659
8.649
8.647
7.675
7.671
7.221
7.201
7.180
7.018
7.015
6.818
6.816
3.640
N
Cl
OMe
In CDCl3
ppm
1.00
1.00
3.10
1.01
1.02
3.11

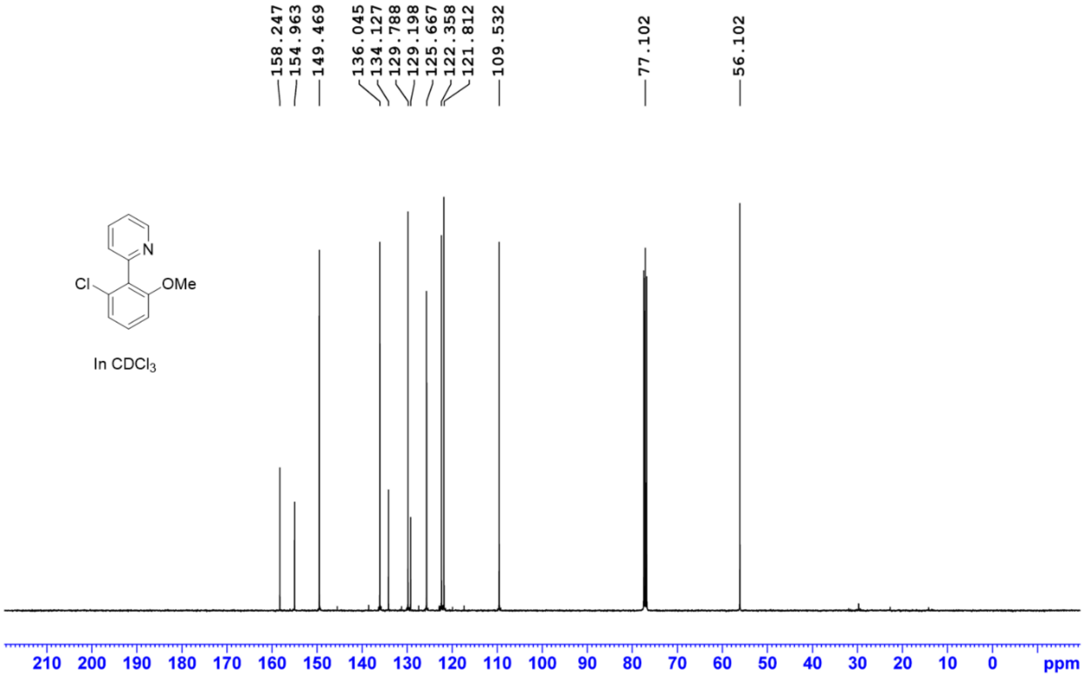
158.247
154.963
149.469
136.045
134.127
129.788
129.198
125.667
122.358
121.812
109.532
77.102
56.102
N
Cl
OMe
In CDCl3
ppm

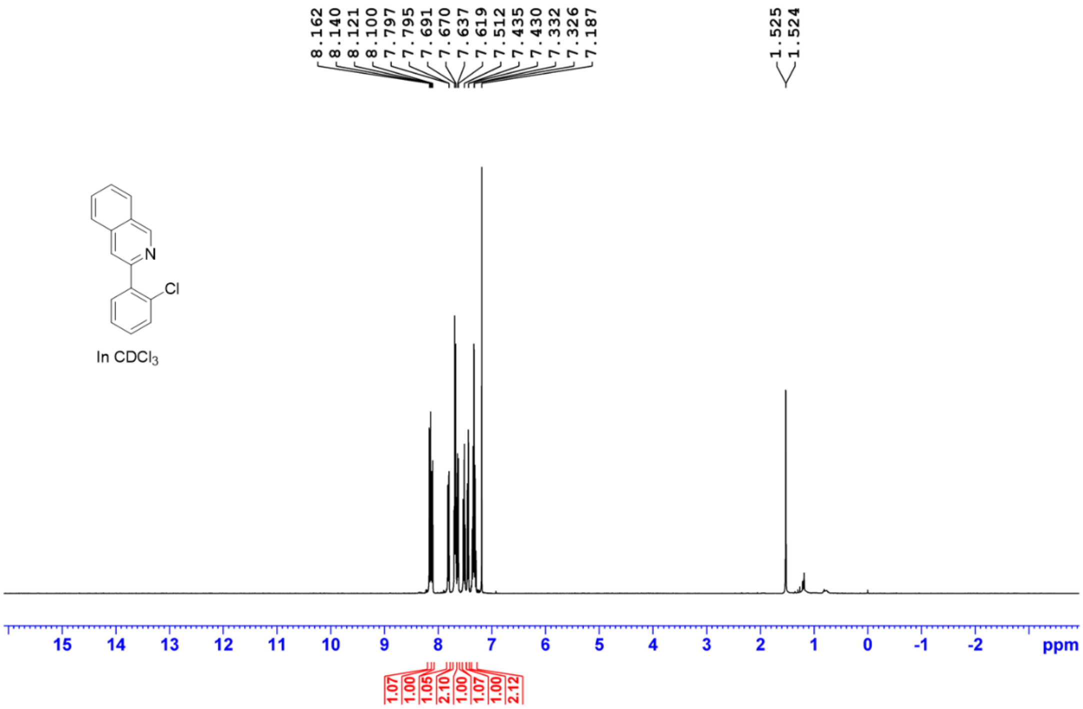
8.162
8.140
8.121
8.100
7.797
7.795
7.691
7.670
7.637
7.619
7.512
7.435
7.430
7.332
7.326
7.187
1.525
1.524
N
Cl
In CDCl3
15 14 13 12 11 10 9 8 7 6 5 4 3 2 1 0 -1 -2 ppm
1.07
1.00
1.05
2.10
1.00
1.07
1.00
2.12

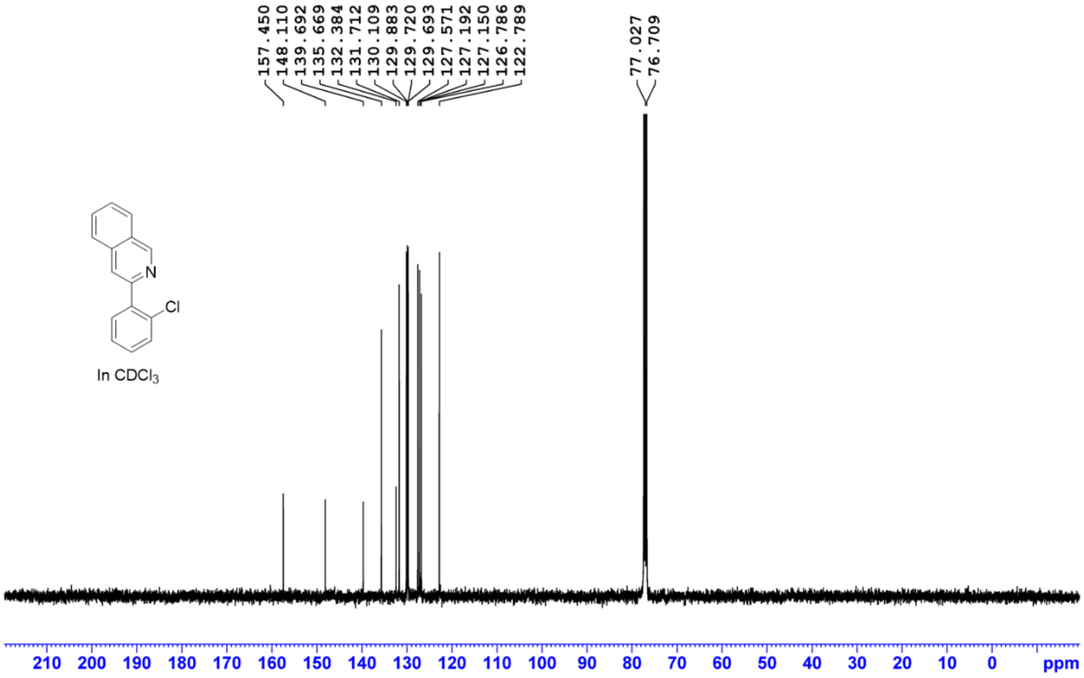
157.450
148.110
139.692
135.669
132.384
131.712
130.109
129.883
129.720
129.693
127.571
127.192
127.150
126.786
122.789
77.027
76.709
N
Cl
In CDCl3
210 200 190 180 170 160 150 140 130 120 110 100 90 80 70 60 50 40 30 20 10 0 ppm

Supplementary Information

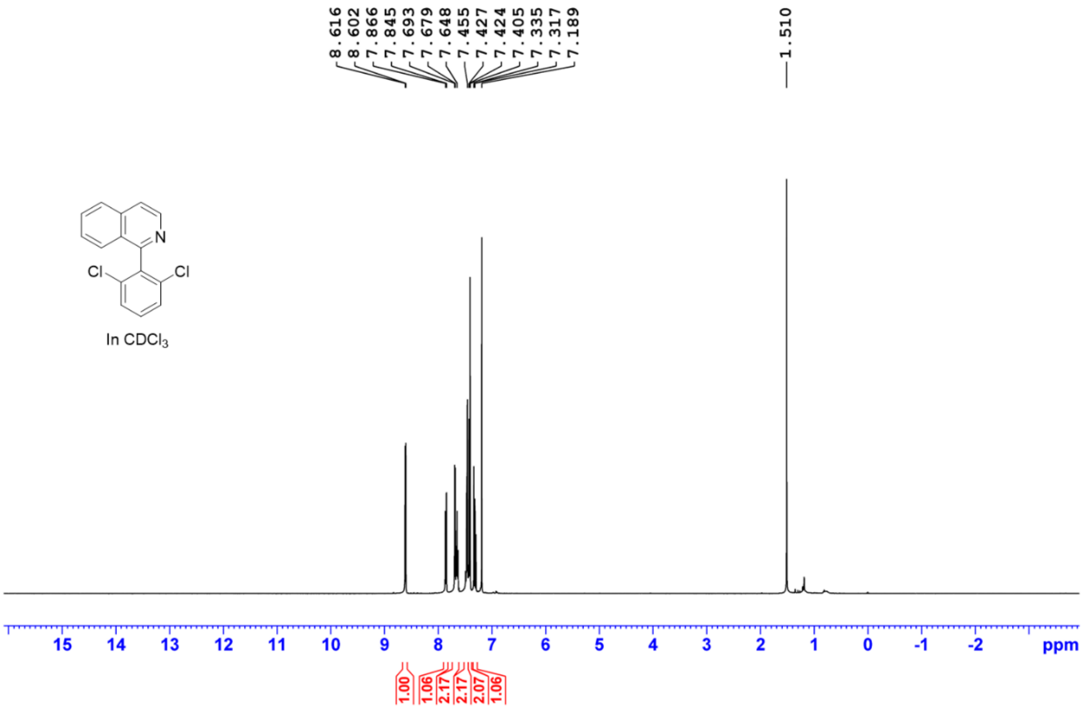

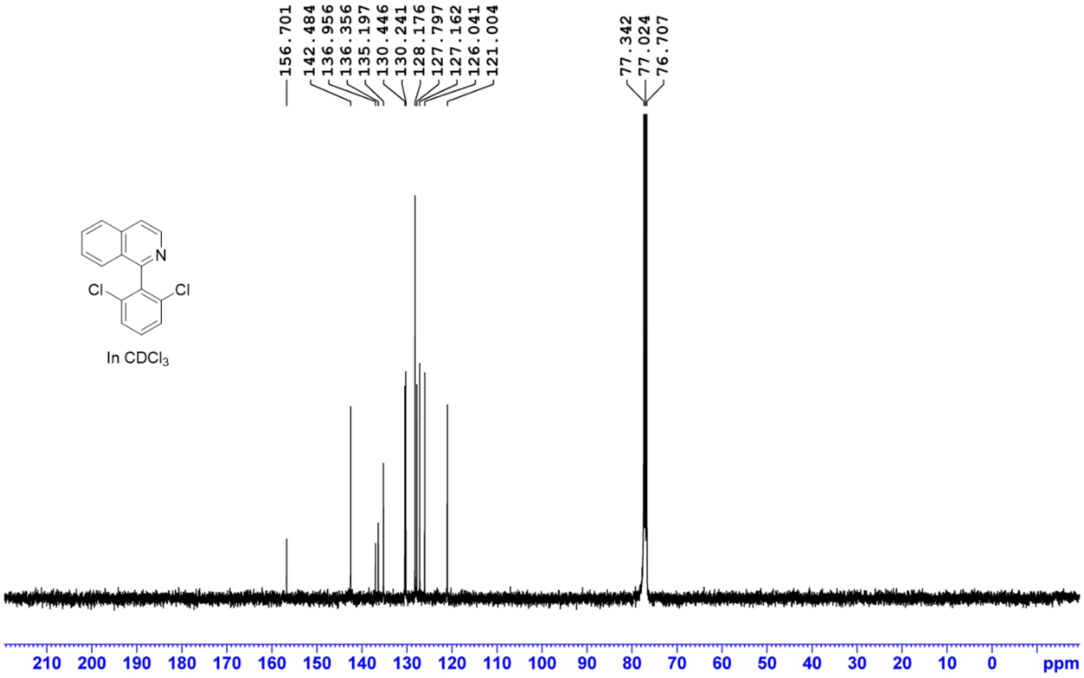

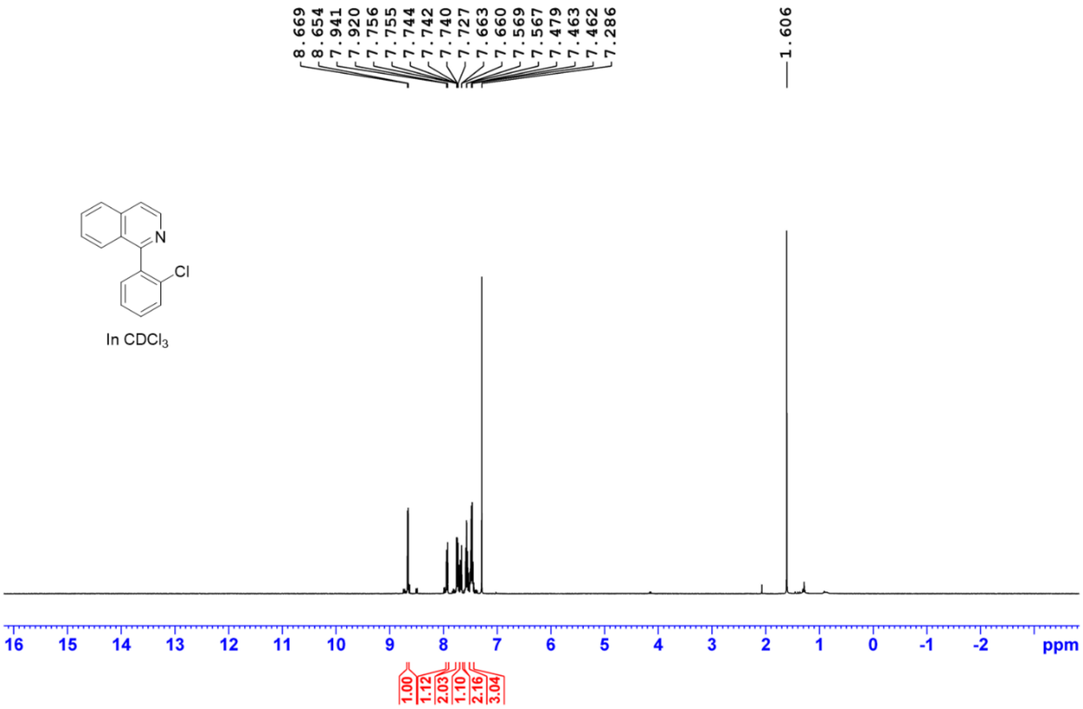

8.669
8.654
7.941
7.920
7.756
7.755
7.744
7.742
7.740
7.727
7.663
7.660
7.569
7.567
7.479
7.463
7.462
7.286
1.606
Cl
N
In CDCl3
16 15 14 13 12 11 10 9 8 7 6 5 4 3 2 1 0 -1 -2 ppm
1.00
1.12
2.03
1.10
2.16
3.04

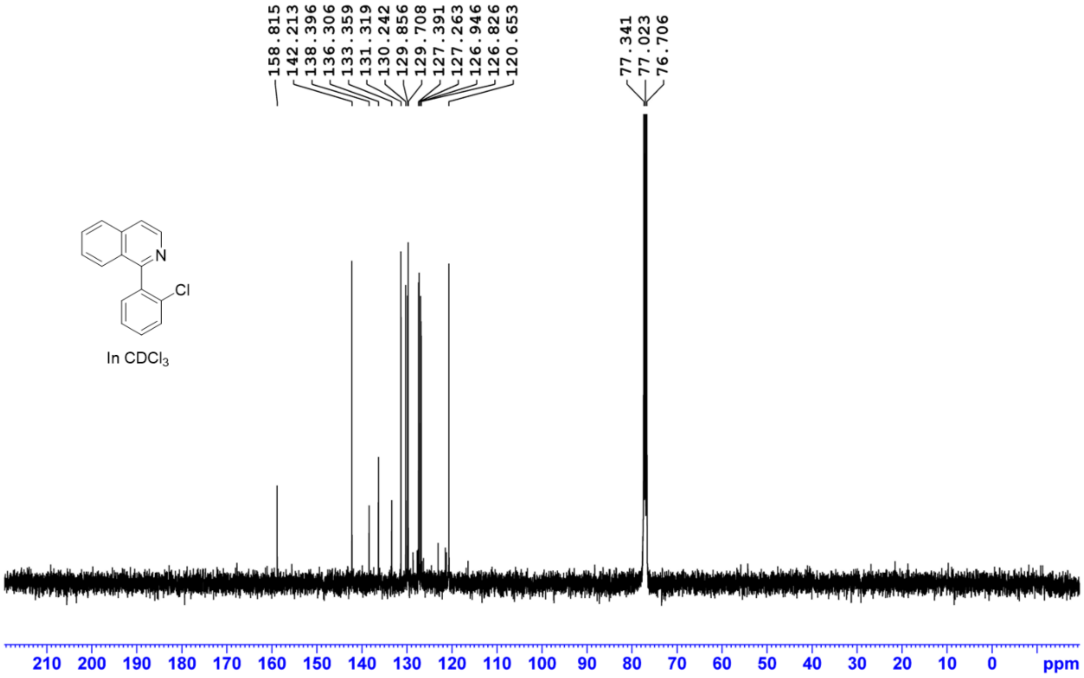

158.815
142.213
138.396
136.306
133.359
131.319
130.242
129.856
129.708
127.391
127.263
126.946
126.826
120.653
77.341
77.023
76.706
Cl
N
In CDCl3
210 200 190 180 170 160 150 140 130 120 110 100 90 80 70 60 50 40 30 20 10 0 ppm

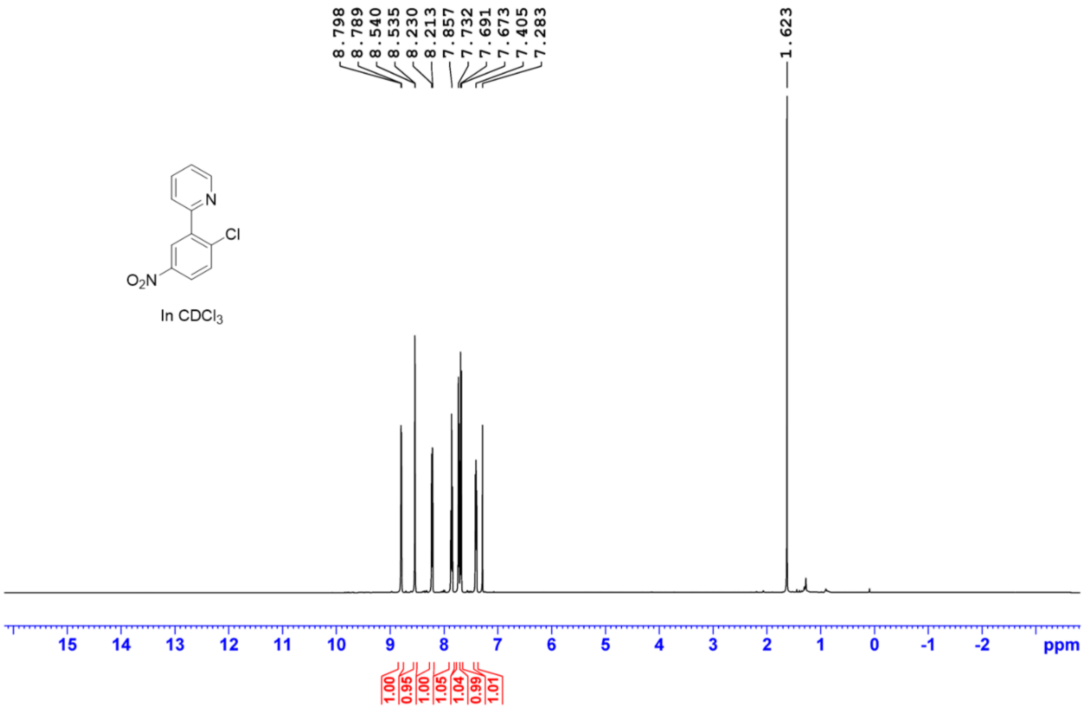
8.798
8.789
8.540
8.535
8.230
8.213
7.857
7.732
7.691
7.673
7.405
7.283
1.623
N
Cl
O2N
In CDCl3
15
14
13
12
11
10
9
8
7
6
5
4
3
2
1
0
-1
-2
ppm
1.00
0.95
1.00
1.05
1.04
0.99
1.01

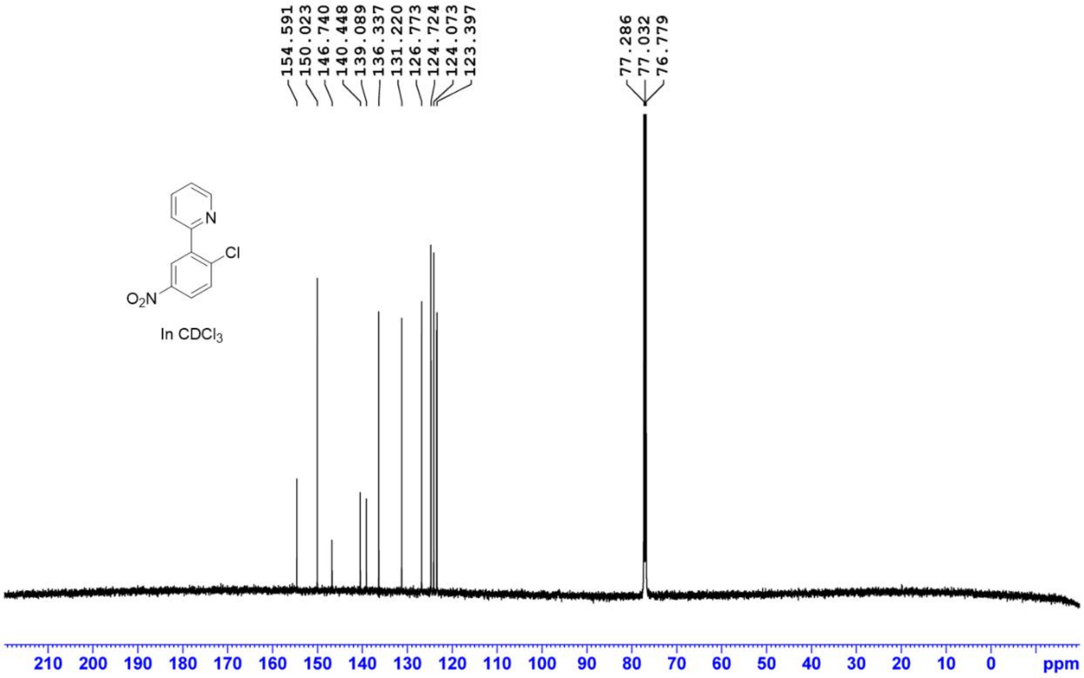
154.591
150.023
146.740
140.448
139.089
136.337
131.220
126.773
124.724
124.073
123.397
77.286
77.032
76.779
N
Cl
O2N
In CDCl3
210
200
190
180
170
160
150
140
130
120
110
100
90
80
70
60
50
40
30
20
10
0
ppm

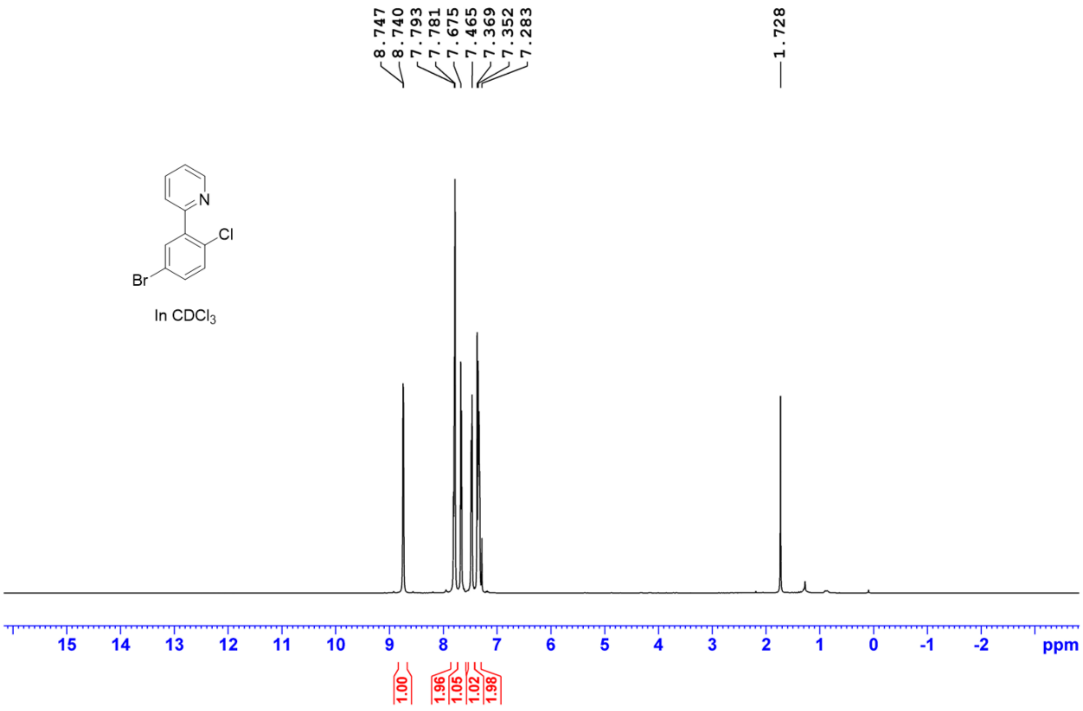
8.747
8.740
7.793
7.781
7.675
7.465
7.369
7.352
7.283
1.728
In CDCl3
1.00
1.96
1.05
1.02
1.98
ppm

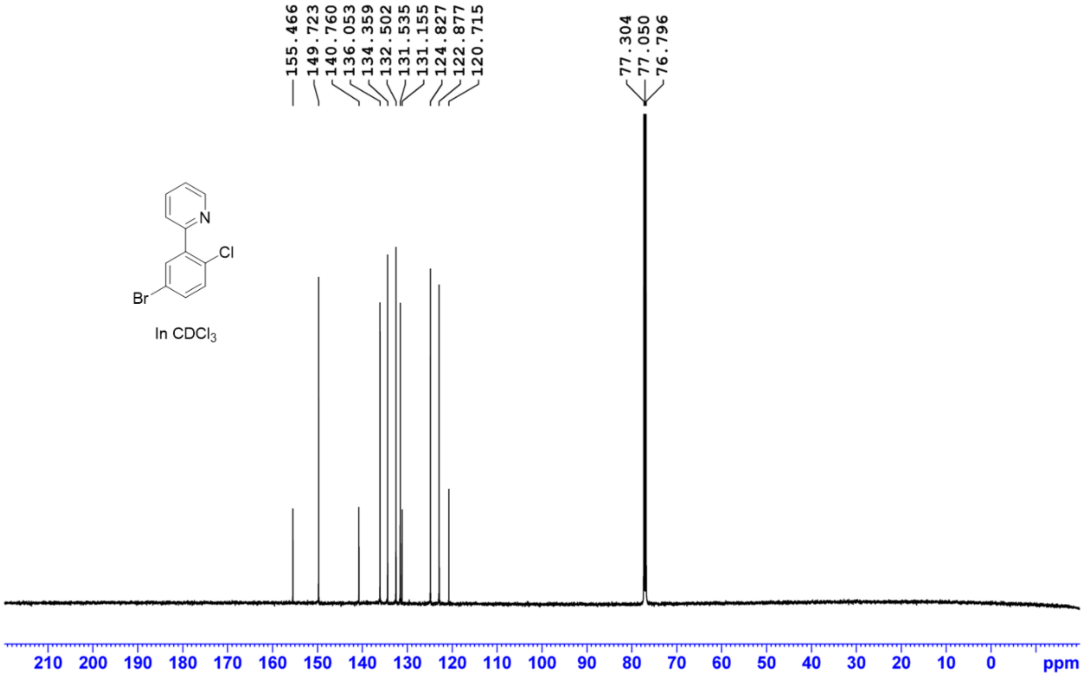
155.466
149.723
140.760
136.053
134.359
132.502
131.535
131.155
124.827
122.877
120.715
77.304
77.050
76.796
In CDCl3
ppm

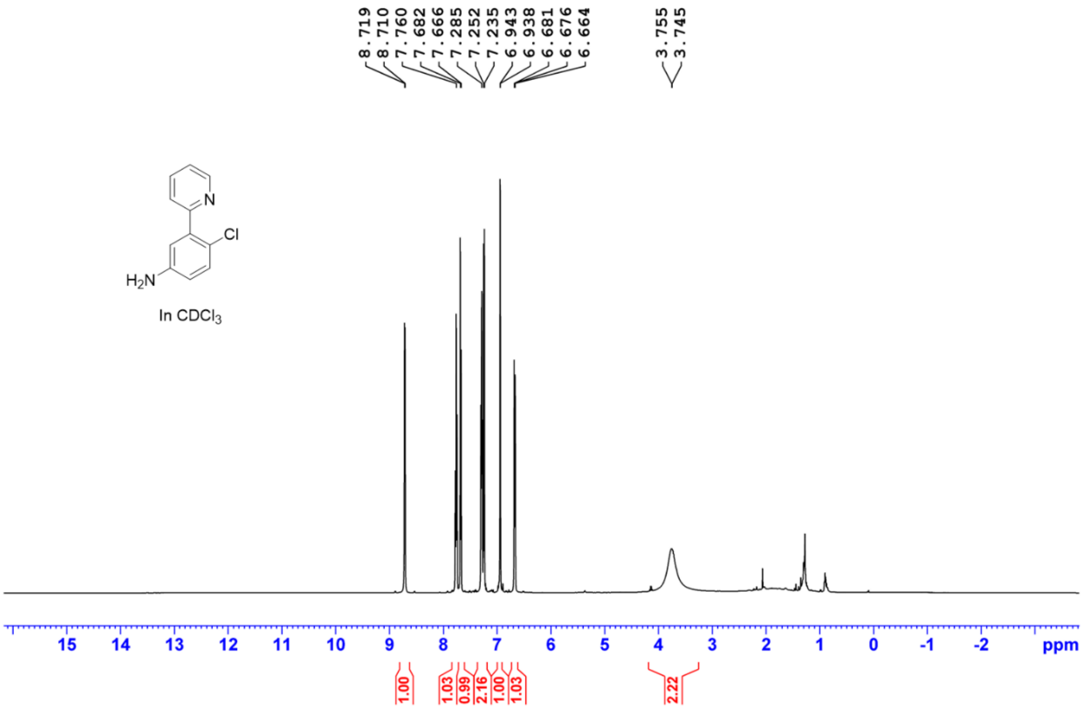
8.719
8.710
7.760
7.682
7.666
7.285
7.252
7.235
6.943
6.938
6.681
6.676
6.664
3.755
3.745
Cl
N
H_2N
In $CDCl_3$
15 14 13 12 11 10 9 8 7 6 5 4 3 2 1 0 -1 -2 ppm
1.00
1.03
0.99
2.16
1.00
1.03
2.22

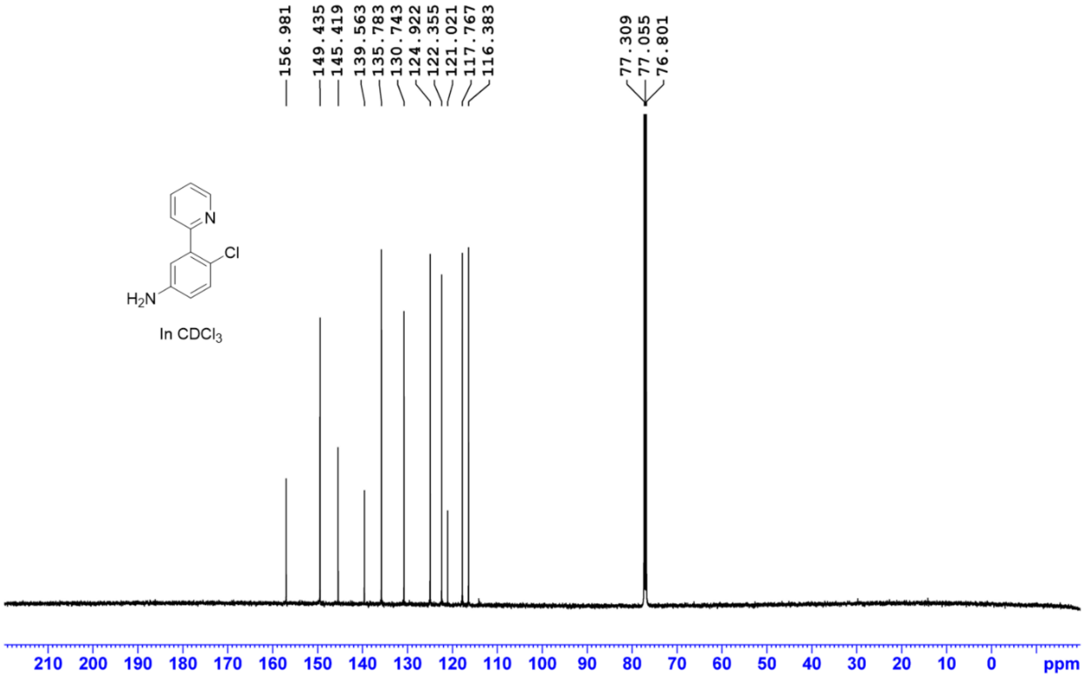
156.981
149.435
145.419
139.563
135.783
130.743
124.922
122.355
121.021
117.767
116.383
77.309
77.055
76.801
Cl
N
H_2N
In $CDCl_3$
210 200 190 180 170 160 150 140 130 120 110 100 90 80 70 60 50 40 30 20 10 0 ppm

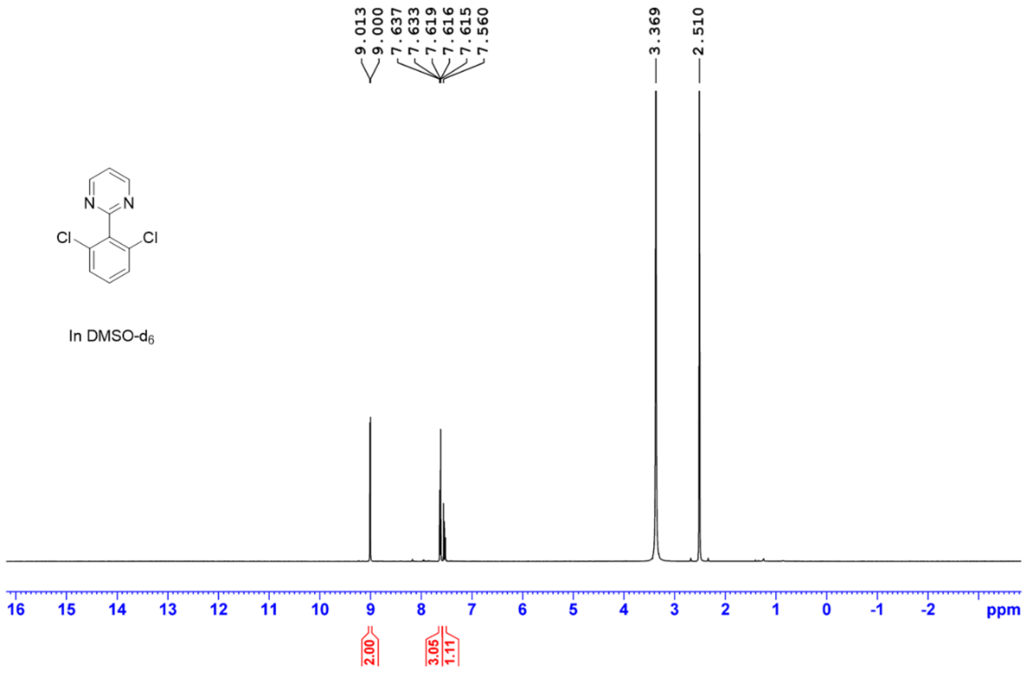
9.013
9.000
7.637
7.633
7.619
7.616
7.615
7.560
3.369
2.510
In DMSO-d6
2.00
3.05
1.11
ppm

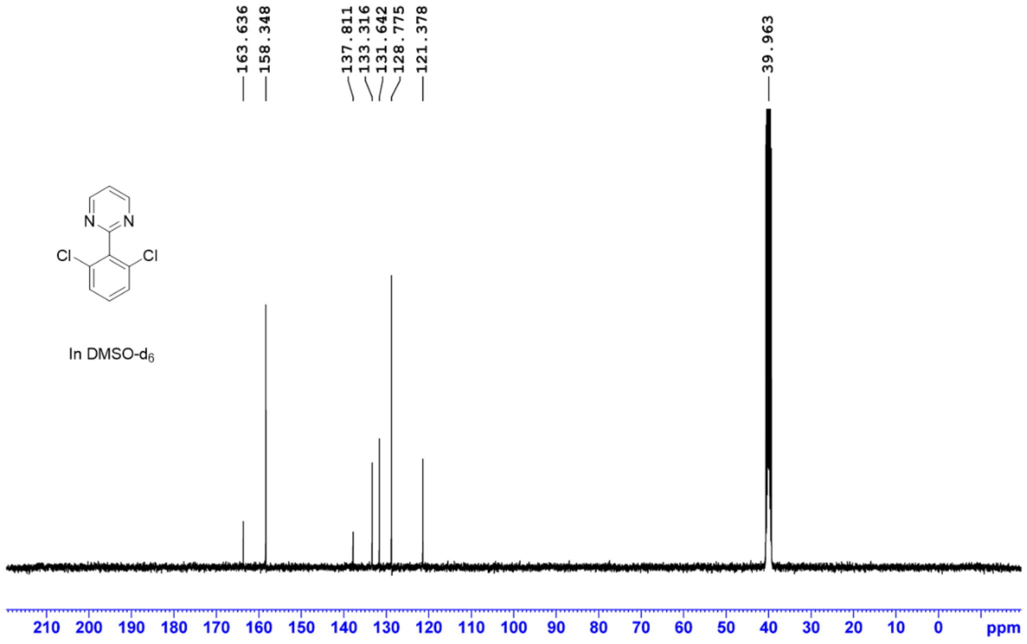
163.636
158.348
137.811
133.316
131.642
128.775
121.378
39.963
In DMSO-d6
ppm

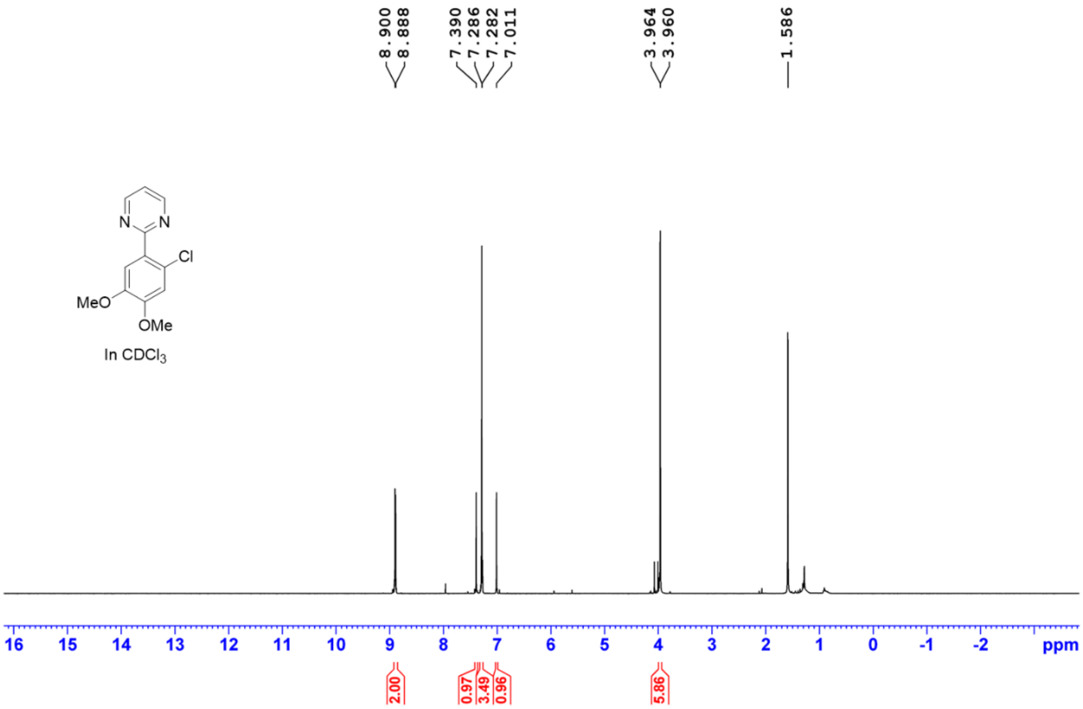

8.900
8.888
7.390
7.286
7.282
7.011
3.964
3.960
1.586
N
N
Cl
MeO
OMe
In CDCl3
16 15 14 13 12 11 10 9 8 7 6 5 4 3 2 1 0 -1 -2 ppm
2.00
0.97
3.49
0.96
5.86

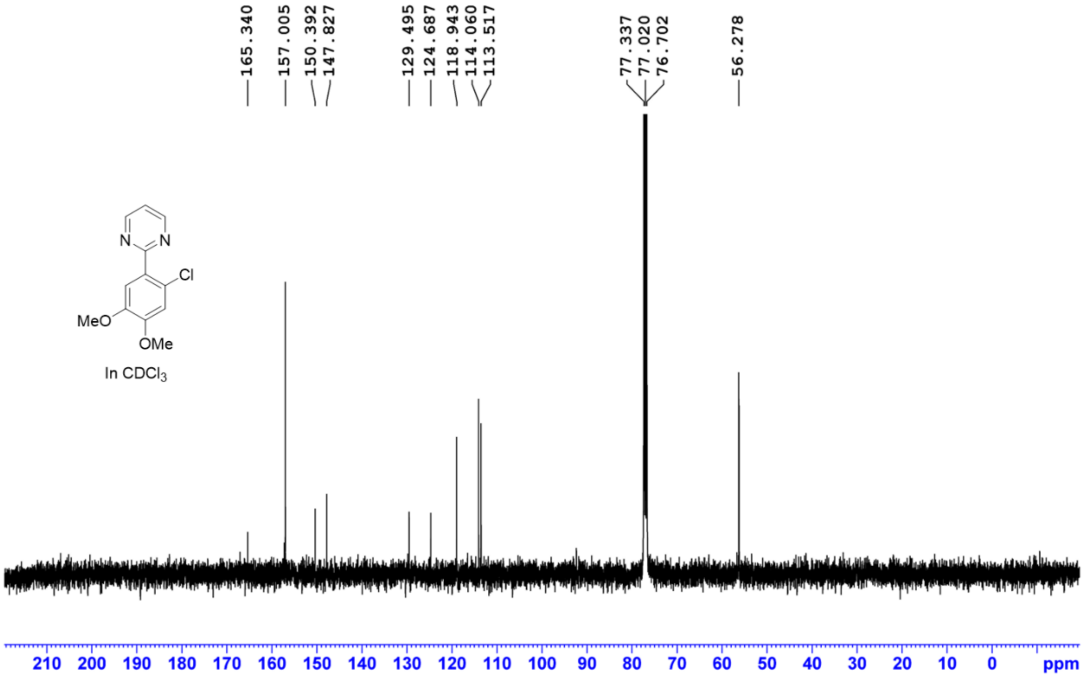

165.340
157.005
150.392
147.827
129.495
124.687
118.943
114.060
113.517
77.337
77.020
76.702
56.278
N
N
Cl
MeO
OMe
In CDCl3
210 200 190 180 170 160 150 140 130 120 110 100 90 80 70 60 50 40 30 20 10 0 ppm

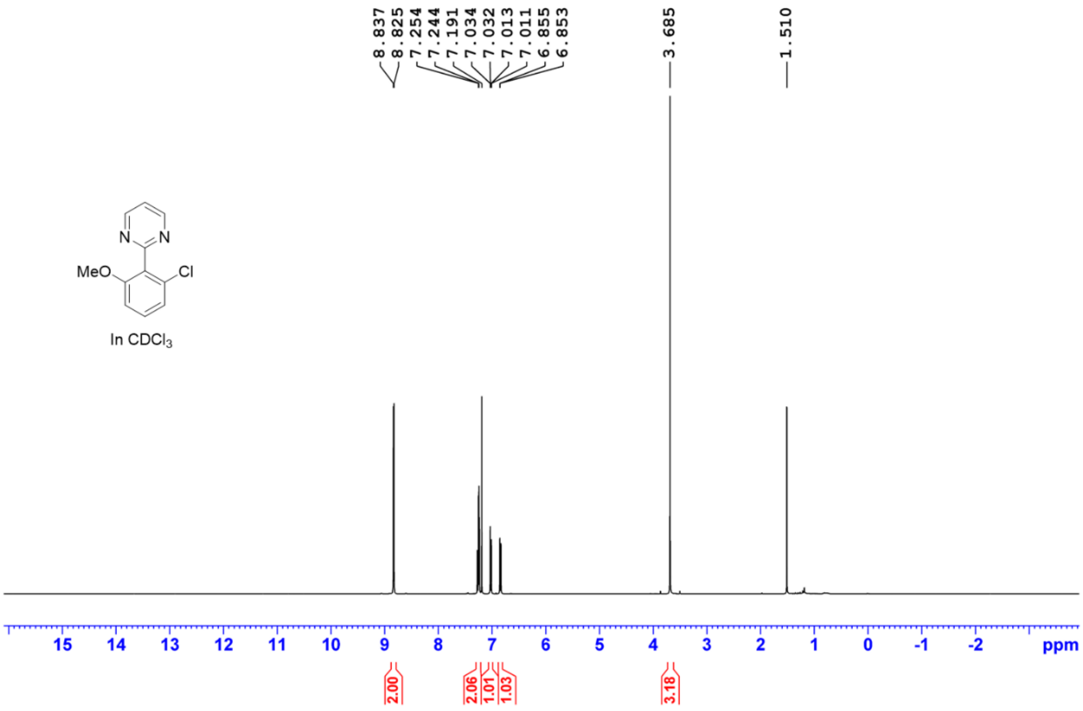
8.837
8.825
7.254
7.244
7.191
7.034
7.032
7.013
7.011
6.855
6.853
3.685
1.510
N
N
MeO
Cl
In $CDCl_3$
2.00
2.06
1.01
1.03
3.18
ppm

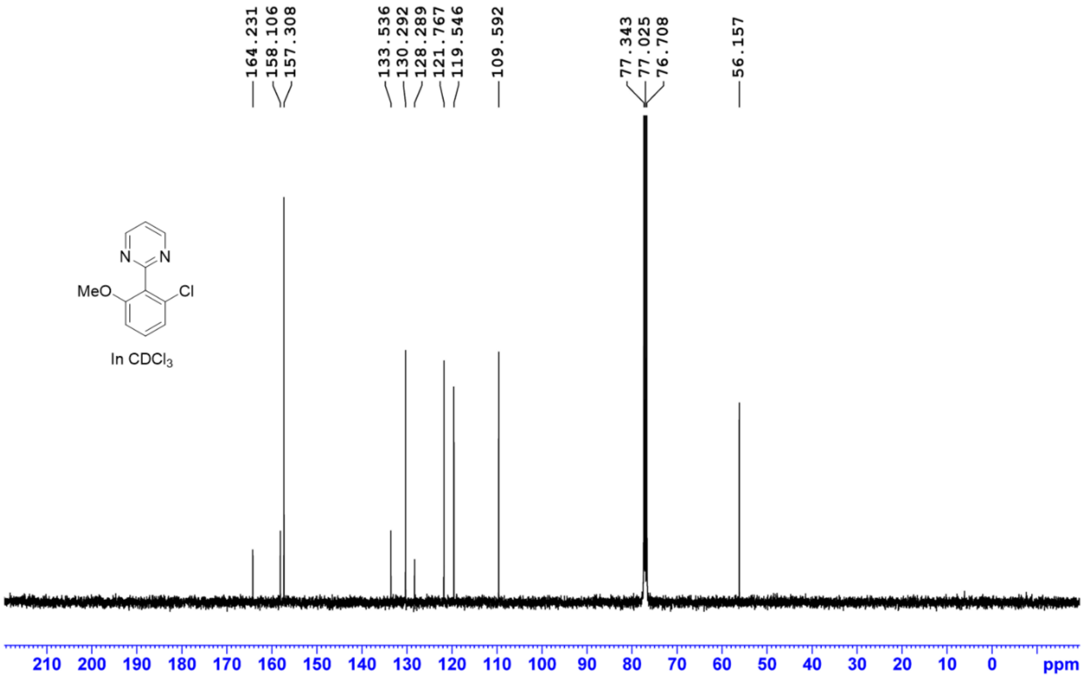
164.231
158.106
157.308
133.536
130.292
128.289
121.767
119.546
109.592
77.343
77.025
76.708
56.157
N
N
MeO
Cl
In $CDCl_3$
ppm

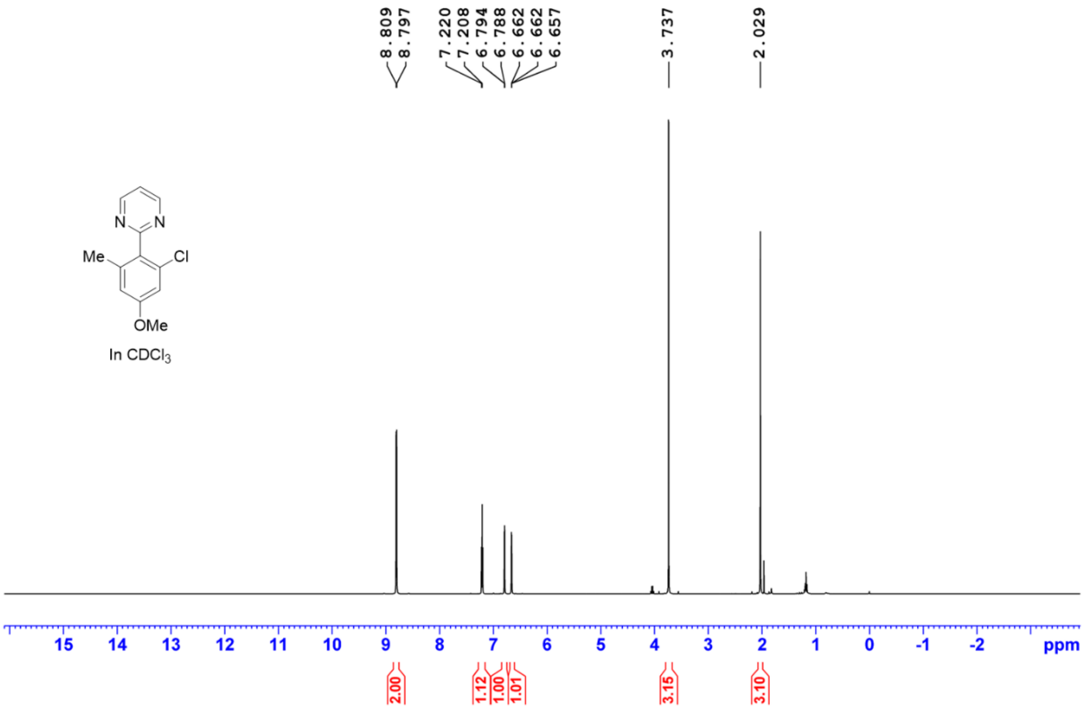

8.809
8.797
7.220
7.208
6.794
6.788
6.662
6.662
6.657
3.737
2.029
N
N
Me
Cl
OMe
In CDCl3
15
14
13
12
11
10
9
8
7
6
5
4
3
2
1
0
-1
-2
ppm
2.00
1.12
1.00
1.01
3.15
3.10

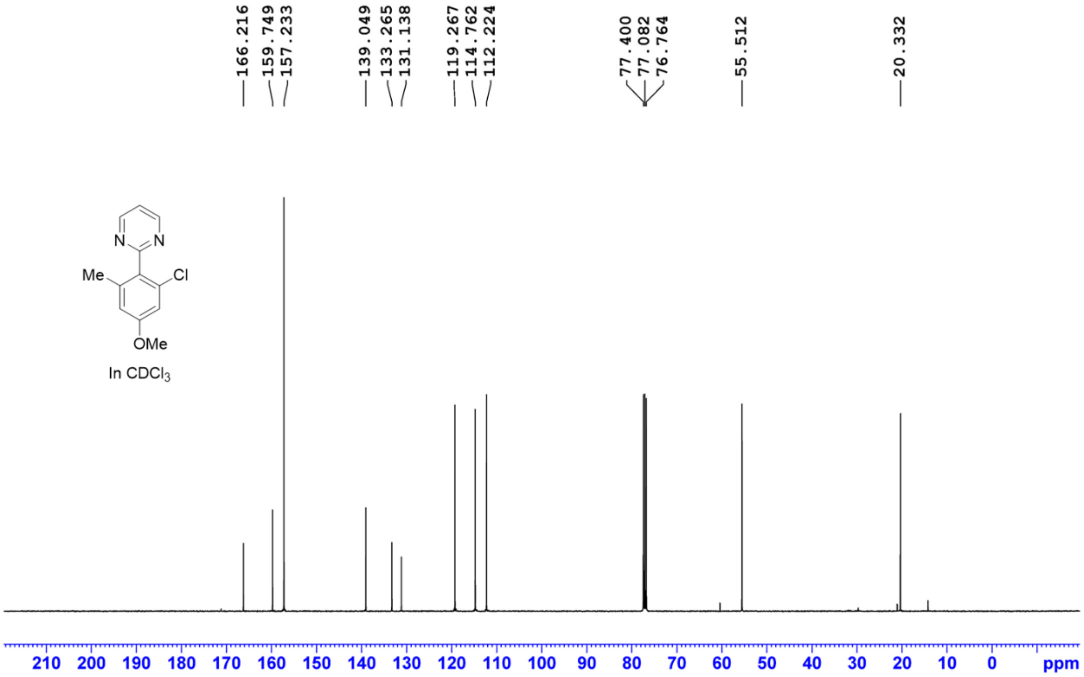

166.216
159.749
157.233
139.049
133.265
131.138
119.267
114.762
112.224
77.400
77.082
76.764
55.512
20.332
N
N
Me
Cl
OMe
In CDCl3
210
200
190
180
170
160
150
140
130
120
110
100
90
80
70
60
50
40
30
20
10
0
ppm

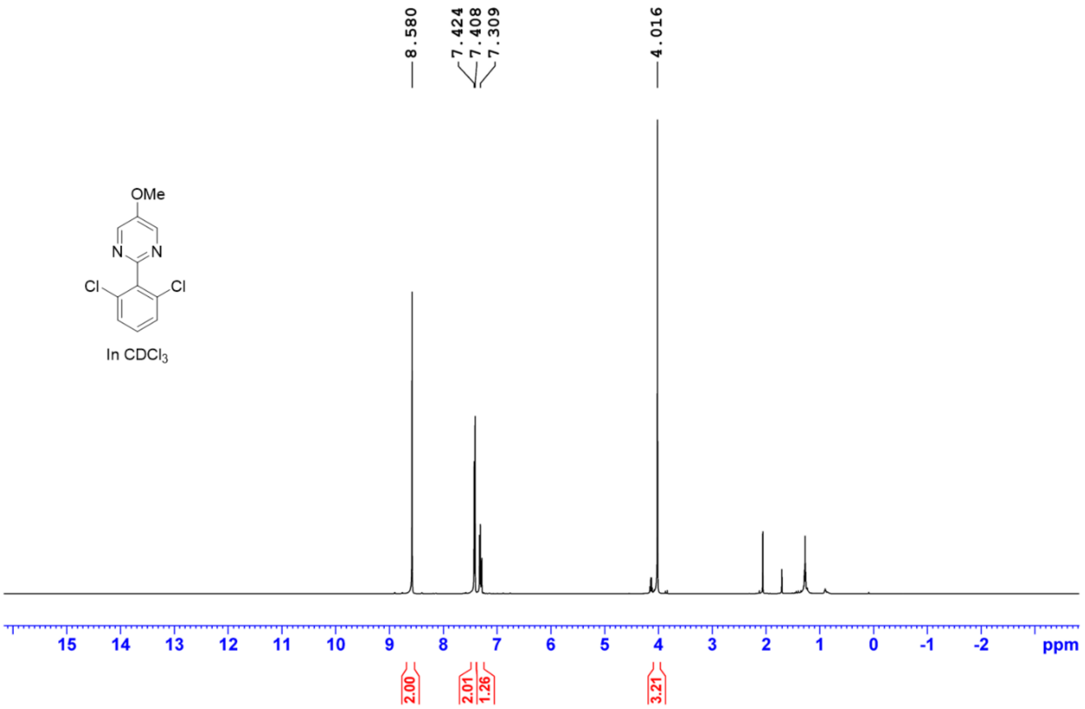
8.580
7.424
7.408
7.309
4.016
OMe
N
N
Cl
Cl
In CDCl3
15 14 13 12 11 10 9 8 7 6 5 4 3 2 1 0 -1 -2 ppm
2.00
2.01
1.26
3.21

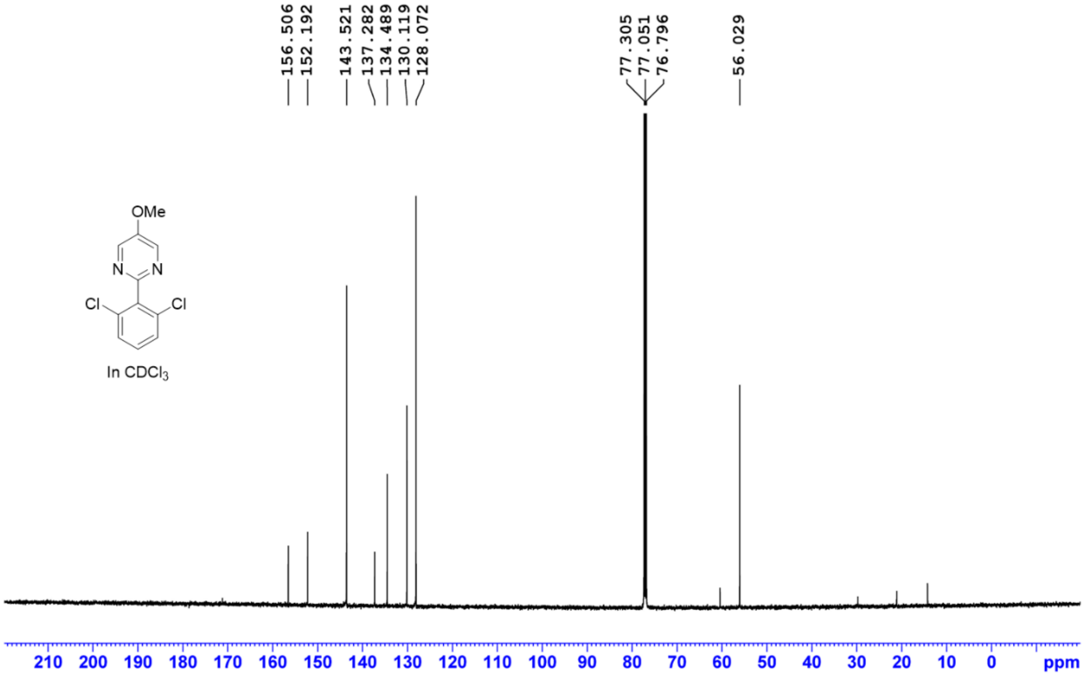
156.506
152.192
143.521
137.282
134.489
130.119
128.072
77.305
77.051
76.796
56.029
OMe
N
N
Cl
Cl
In CDCl3
210 200 190 180 170 160 150 140 130 120 110 100 90 80 70 60 50 40 30 20 10 0 ppm

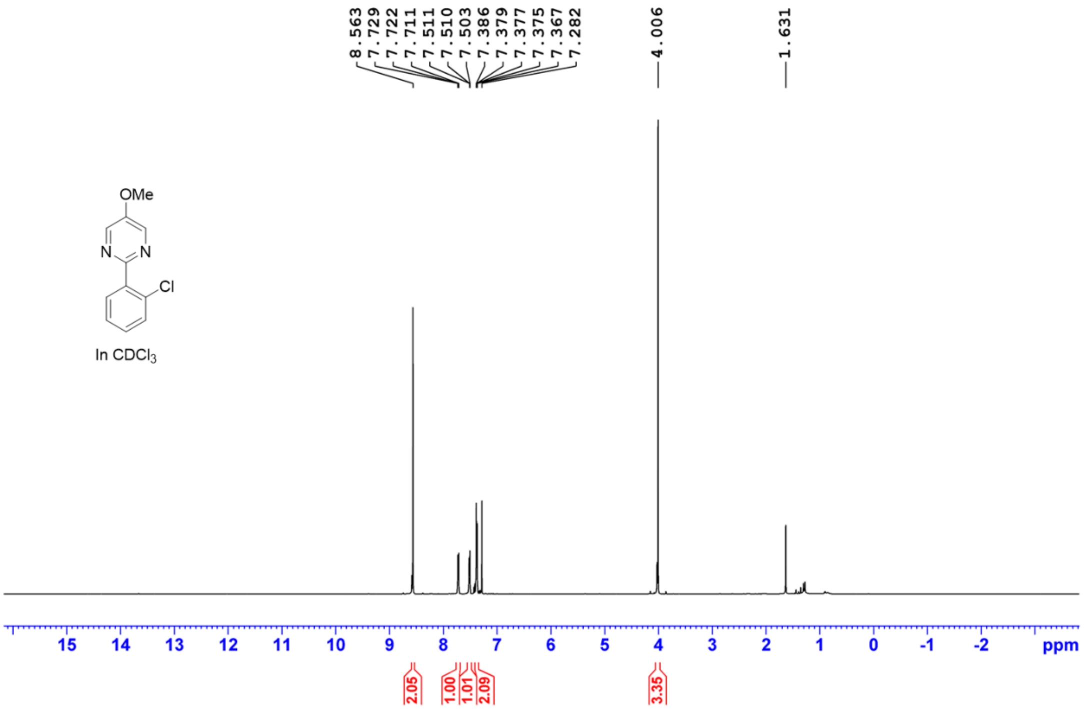
8.563
7.729
7.722
7.711
7.511
7.510
7.503
7.386
7.379
7.377
7.375
7.367
7.282
4.006
1.631
OMe
N
N
Cl
In CDCl3
15 14 13 12 11 10 9 8 7 6 5 4 3 2 1 0 -1 -2 ppm
2.05
1.00
1.01
2.09
3.35

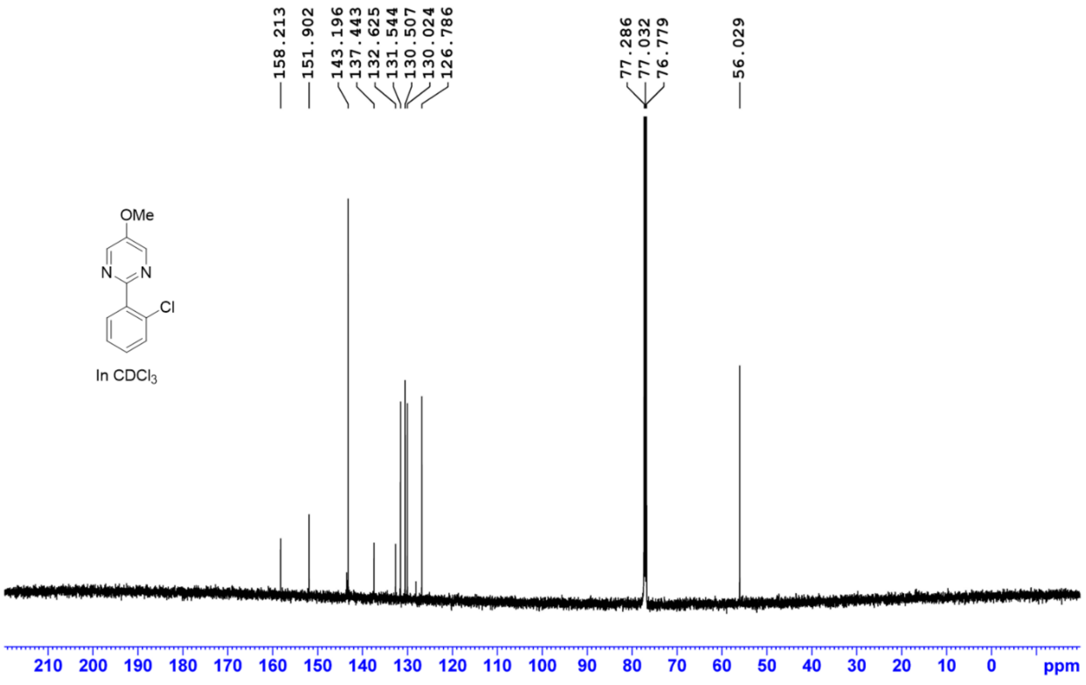
158.213
151.902
143.196
137.443
132.625
131.544
130.507
130.024
126.786
77.286
77.032
76.779
56.029
OMe
N
N
Cl
In CDCl3
210 200 190 180 170 160 150 140 130 120 110 100 90 80 70 60 50 40 30 20 10 0 ppm

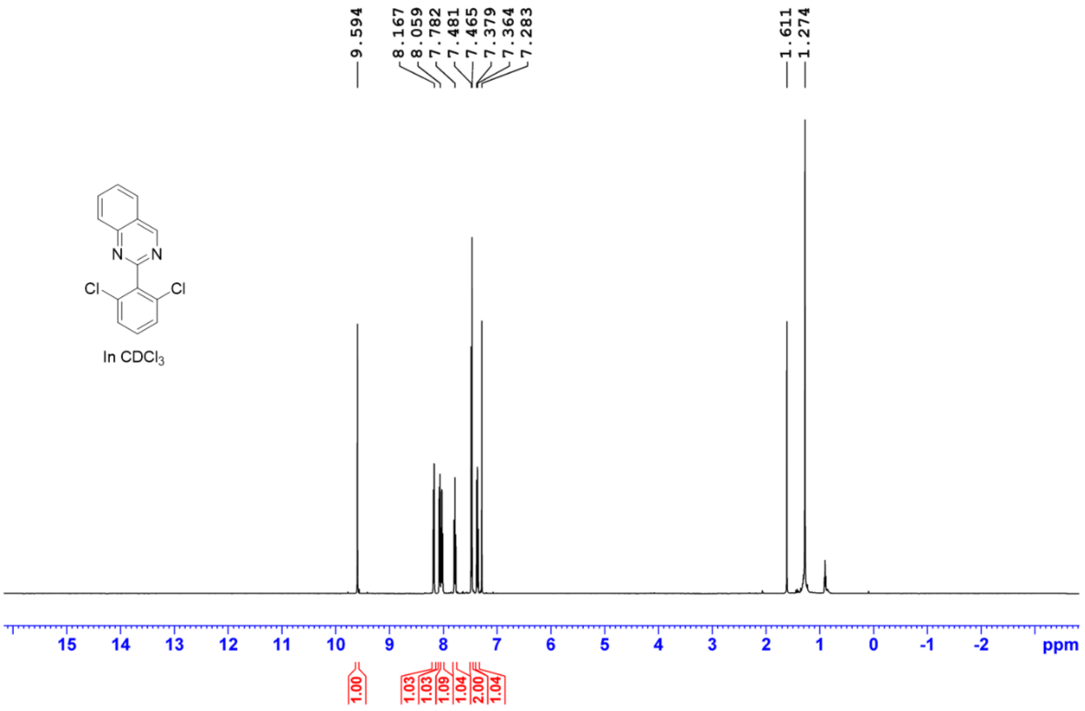
9.594
8.167
8.059
7.782
7.481
7.465
7.379
7.364
7.283
1.611
1.274
In CDCl3
15 14 13 12 11 10 9 8 7 6 5 4 3 2 1 0 -1 -2 ppm
1.00
1.03
1.03
1.09
1.04
2.00
1.04

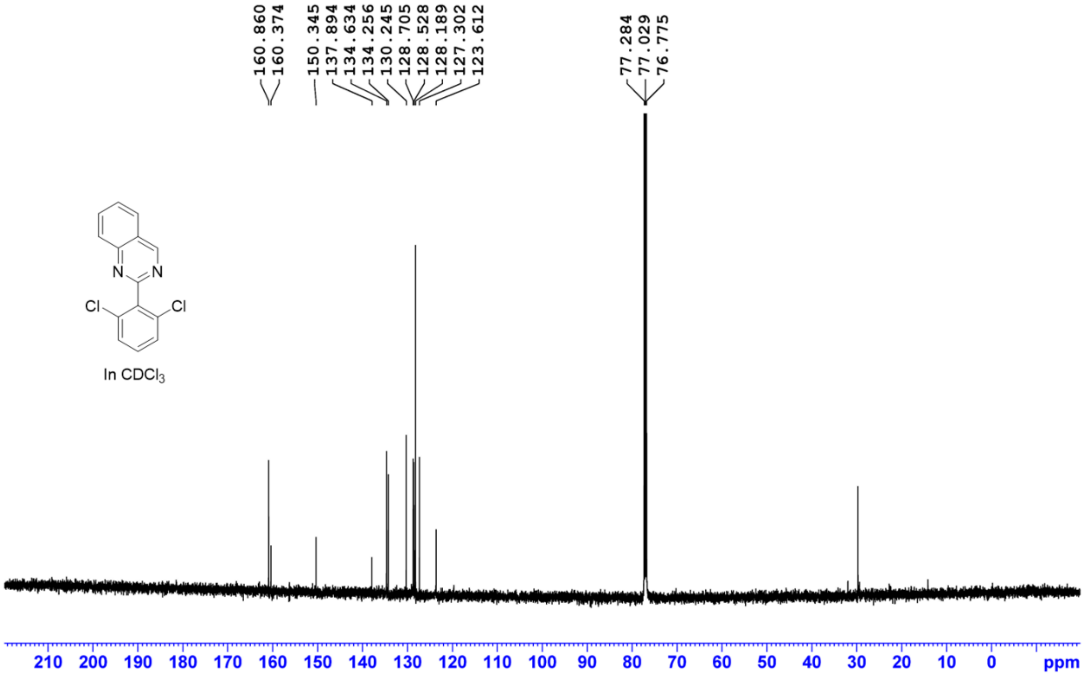
160.860
160.374
150.345
137.894
134.634
134.256
130.245
128.705
128.528
128.189
127.302
123.612
77.284
77.029
76.775
In CDCl3
210 200 190 180 170 160 150 140 130 120 110 100 90 80 70 60 50 40 30 20 10 0 ppm

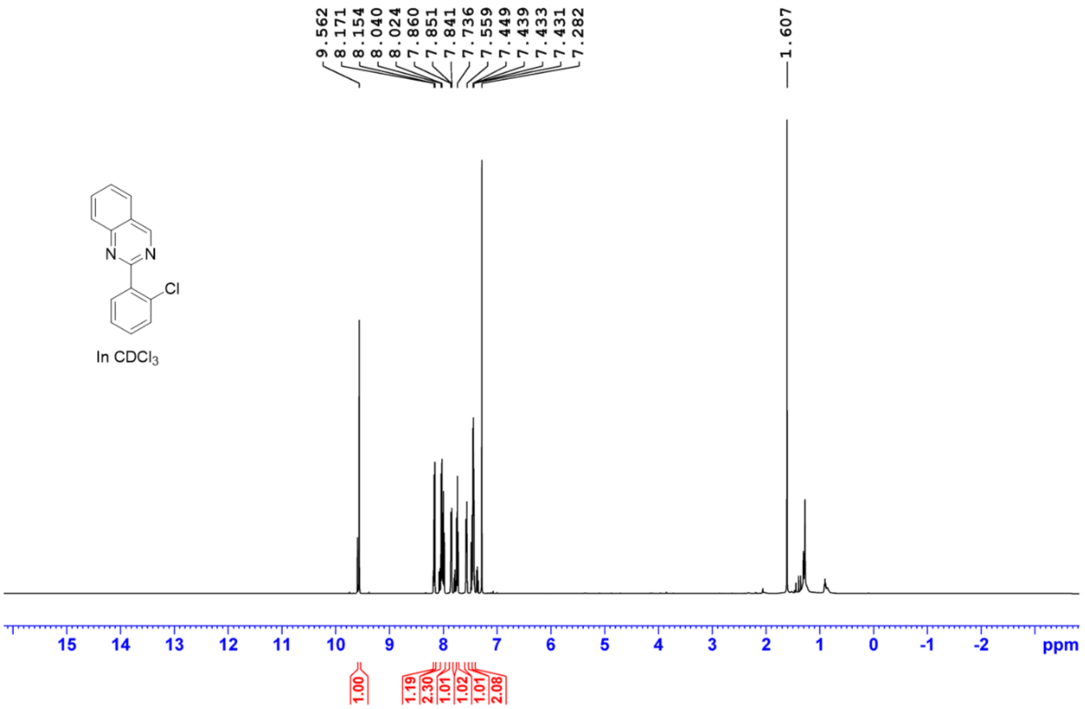
9.562
8.171
8.154
8.040
8.024
7.860
7.851
7.841
7.736
7.559
7.449
7.439
7.433
7.431
7.282
1.607
N
N
Cl
In CDCl3
1.00
1.19
2.30
1.01
1.02
1.01
2.08
ppm

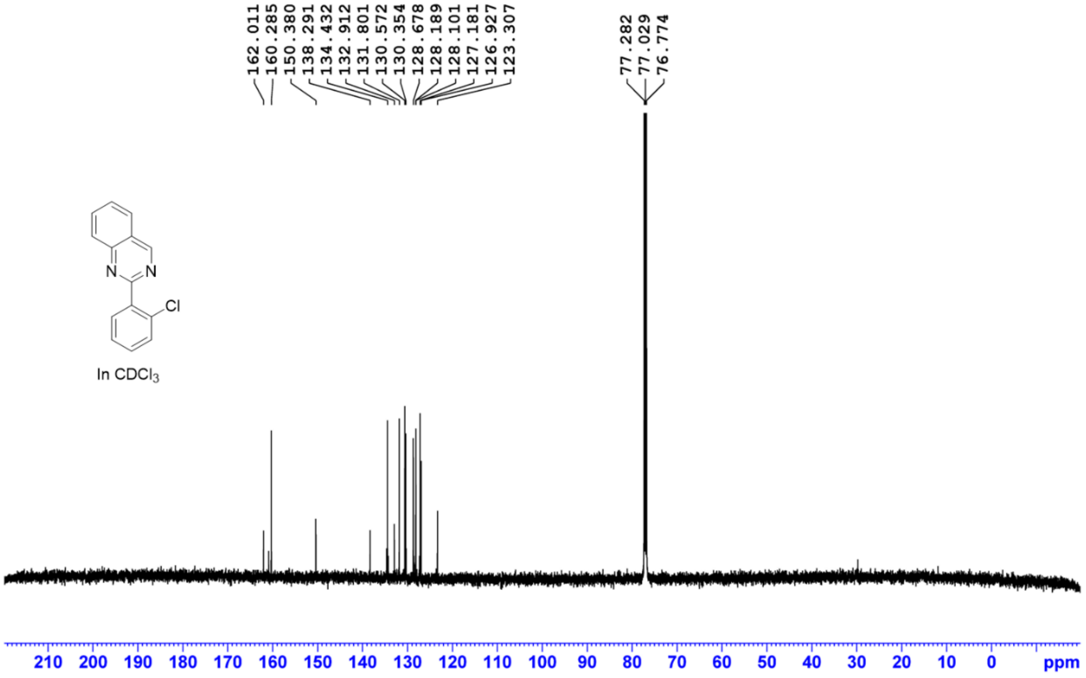
162.011
160.285
150.380
138.291
134.432
132.912
131.801
130.572
130.354
128.678
128.189
128.101
127.181
126.927
123.307
77.282
77.029
76.774
N
N
Cl
In CDCl3
ppm

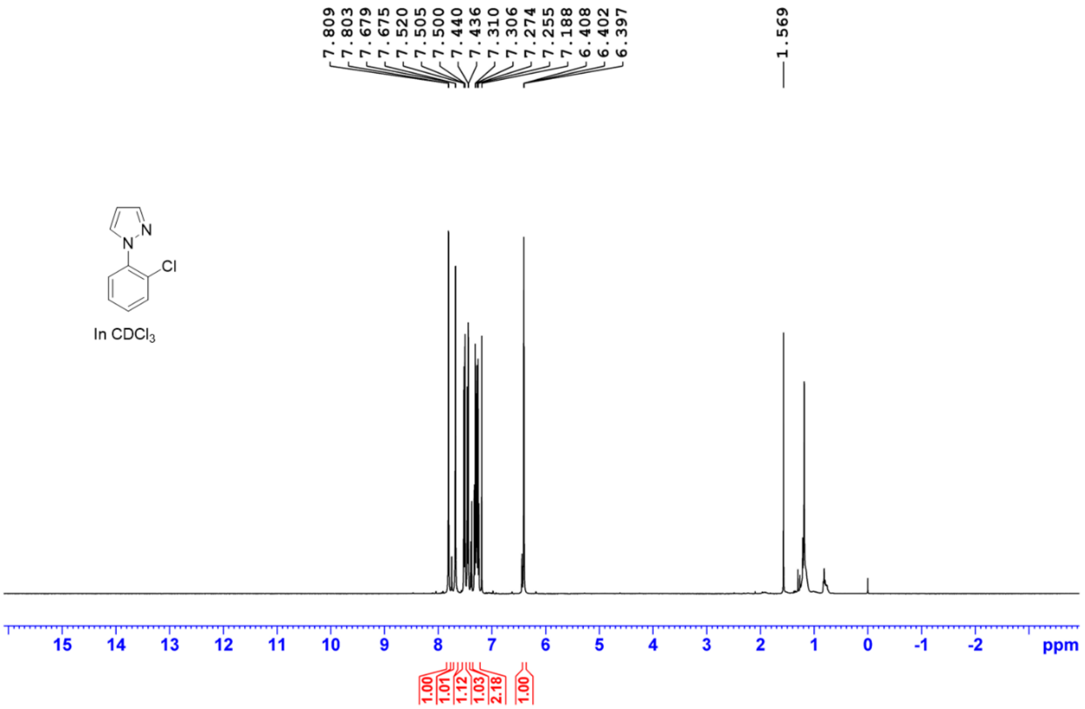
7.809
7.803
7.679
7.675
7.520
7.505
7.500
7.440
7.436
7.310
7.306
7.274
7.255
7.188
6.408
6.402
6.397
1.569
Cl
N
N
In CDCl3
15 14 13 12 11 10 9 8 7 6 5 4 3 2 1 0 -1 -2 ppm
1.00
1.01
1.12
1.03
2.18
1.00

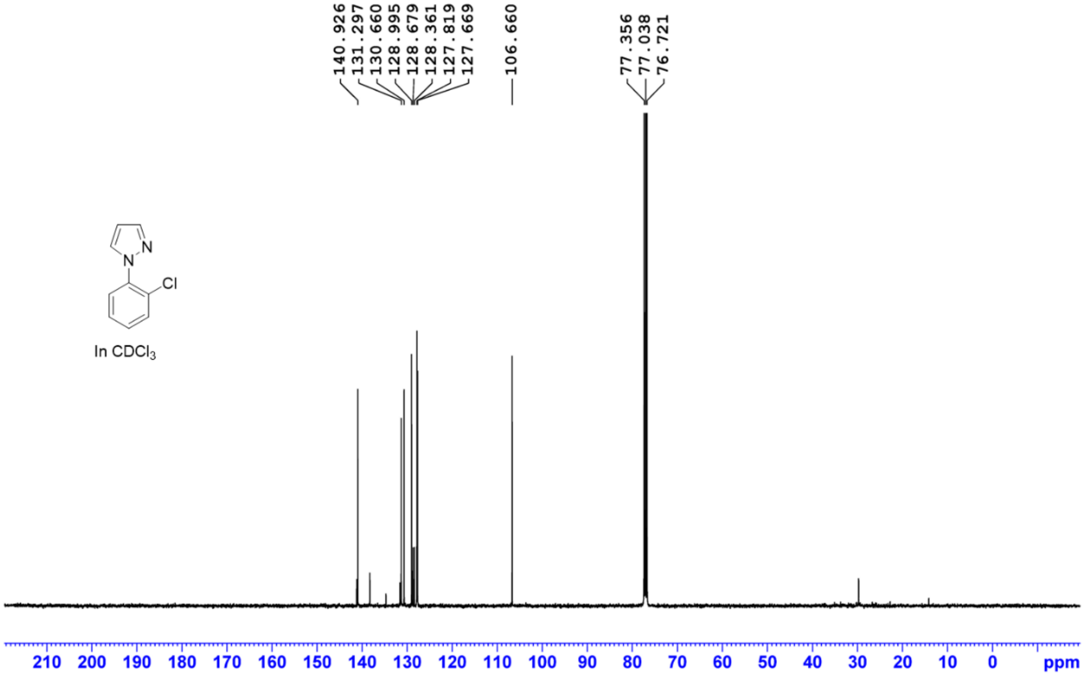
140.926
131.297
130.660
128.995
128.679
128.361
127.819
127.669
106.660
77.356
77.038
76.721
Cl
N
N
In CDCl3
210 200 190 180 170 160 150 140 130 120 110 100 90 80 70 60 50 40 30 20 10 0 ppm

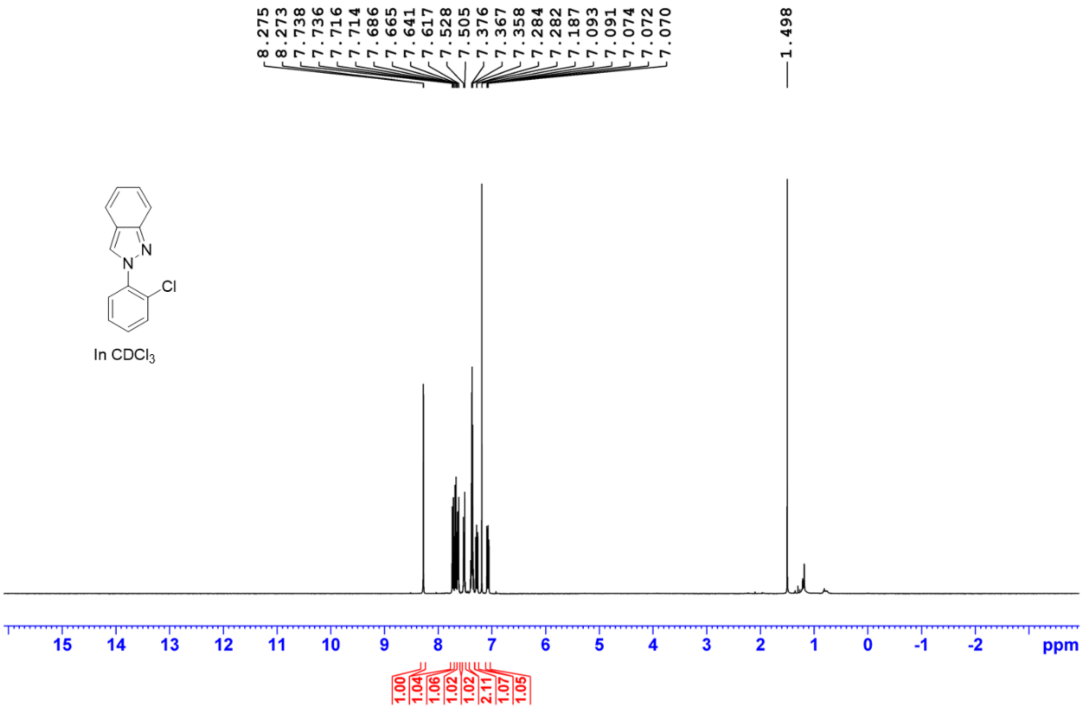

8.275
8.273
7.738
7.736
7.716
7.714
7.686
7.665
7.641
7.617
7.528
7.505
7.376
7.367
7.358
7.284
7.282
7.187
7.093
7.091
7.074
7.072
7.070
1.498
N
N
Cl
In CDCl3
15
14
13
12
11
10
9
8
7
6
5
4
3
2
1
0
-1
-2
ppm
1.00
1.04
1.06
1.02
1.02
2.11
1.07
1.05

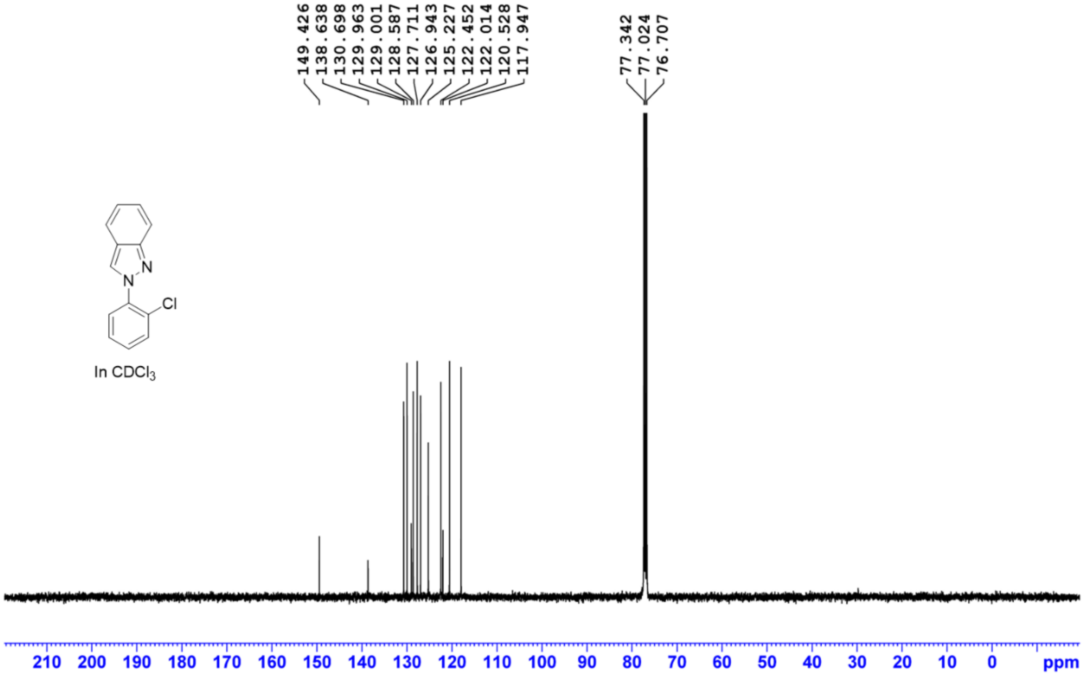

149.426
138.638
130.698
129.963
129.001
128.587
127.711
126.943
125.227
122.452
122.014
120.528
117.947
77.342
77.024
76.707
N
N
Cl
In CDCl3
210
200
190
180
170
160
150
140
130
120
110
100
90
80
70
60
50
40
30
20
10
0
ppm

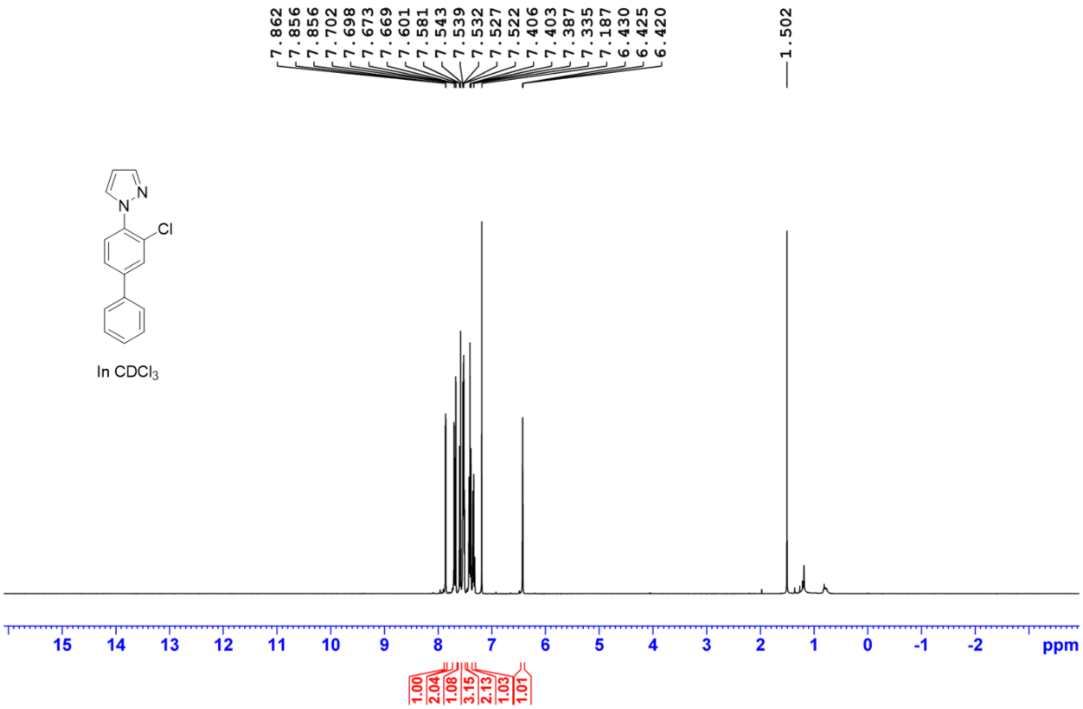
7.862
7.856
7.856
7.702
7.698
7.673
7.669
7.601
7.581
7.543
7.539
7.532
7.527
7.522
7.406
7.403
7.387
7.335
7.187
6.430
6.425
6.420
1.502
N
Cl
In CDCl3
15 14 13 12 11 10 9 8 7 6 5 4 3 2 1 0 -1 -2 ppm
1.00
2.04
1.08
3.15
2.13
1.03
1.01

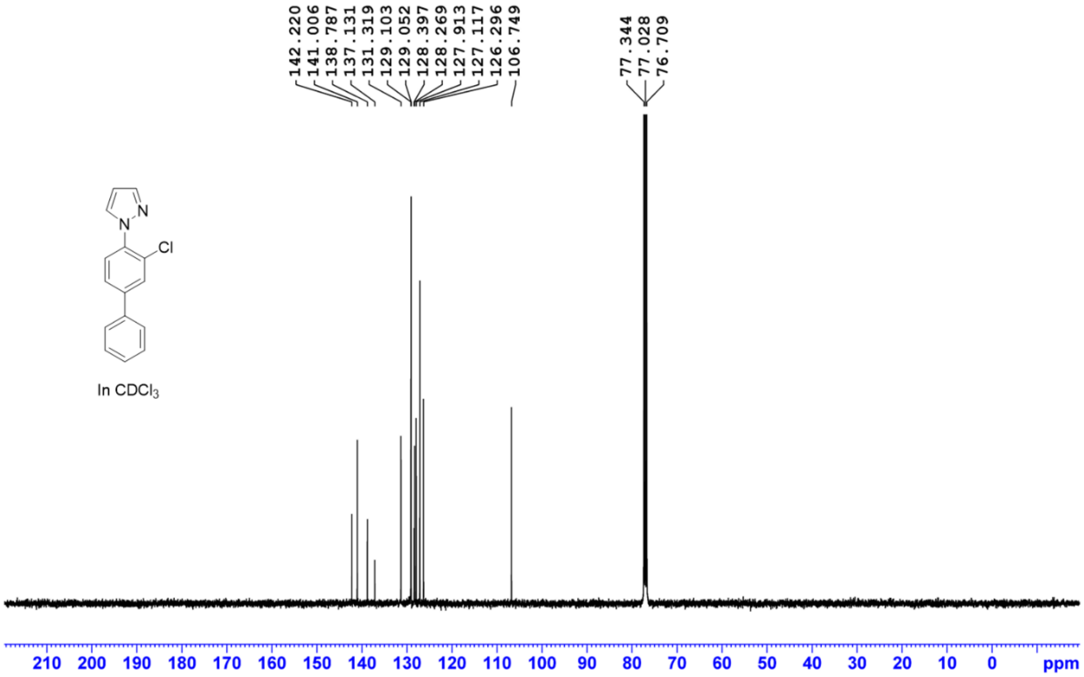
142.220
141.006
138.787
137.131
131.319
129.103
129.052
128.397
128.269
127.913
127.117
126.296
106.749
77.344
77.028
76.709
N
Cl
In CDCl3
210 200 190 180 170 160 150 140 130 120 110 100 90 80 70 60 50 40 30 20 10 0 ppm

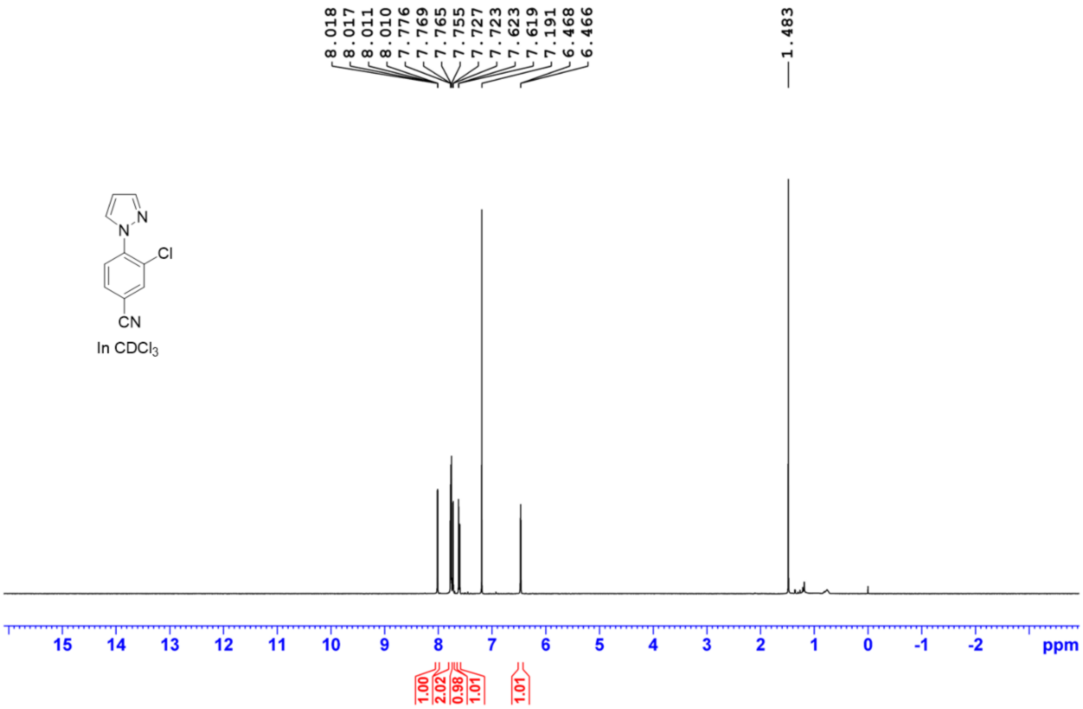
8.018
8.017
8.011
8.010
7.776
7.769
7.765
7.755
7.727
7.723
7.623
7.619
7.191
6.468
6.466
1.483
Cl
N
N
CN
In CDCl$_3$
15 14 13 12 11 10 9 8 7 6 5 4 3 2 1 0 -1 -2 ppm
1.00
2.02
0.98
1.01
1.01

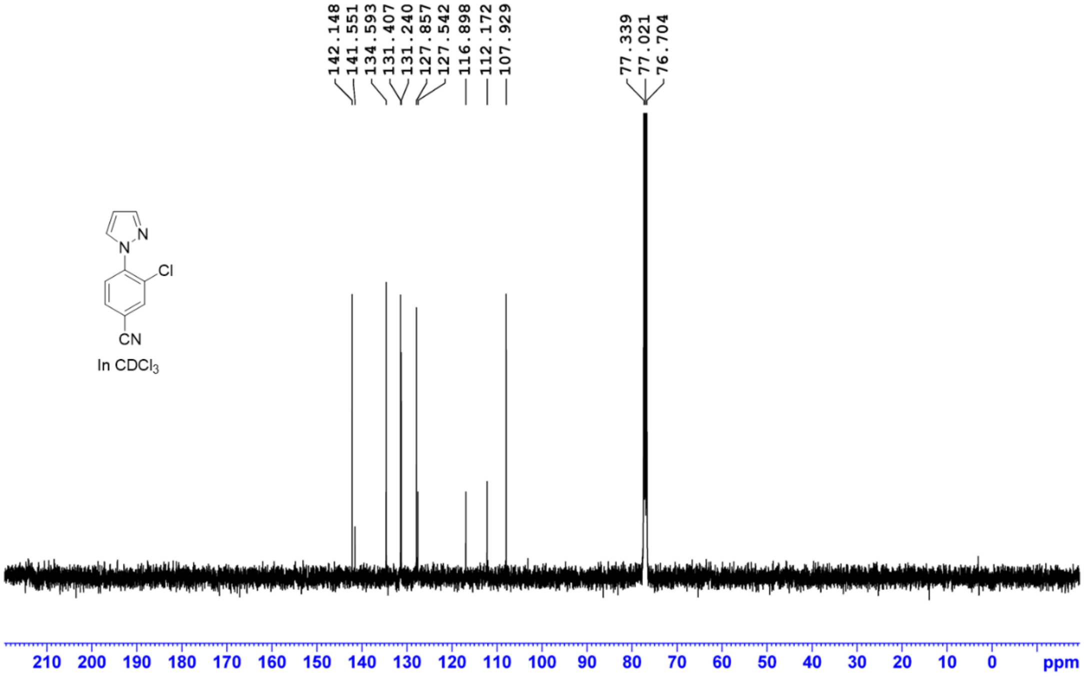
142.148
141.551
134.593
131.407
131.240
127.857
127.542
116.898
112.172
107.929
77.339
77.021
76.704
Cl
N
N
CN
In CDCl$_3$
210 200 190 180 170 160 150 140 130 120 110 100 90 80 70 60 50 40 30 20 10 0 ppm

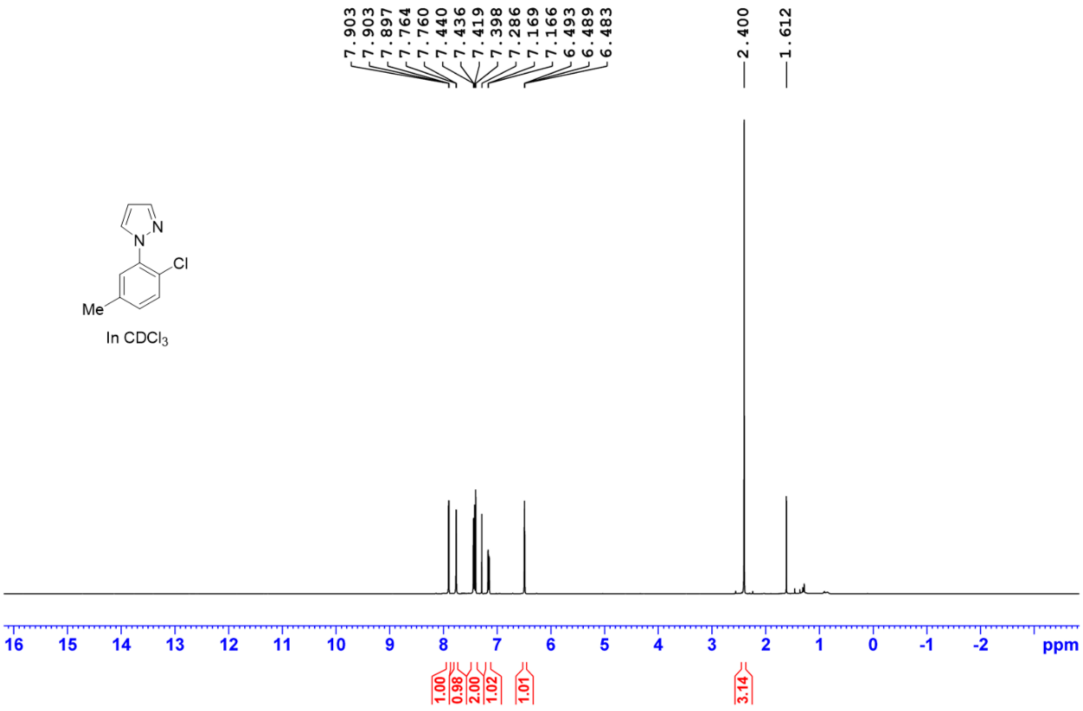
7.903
7.903
7.897
7.764
7.760
7.440
7.436
7.419
7.398
7.286
7.169
7.166
6.493
6.489
6.483
2.400
1.612
Cl
N
N
Me
In CDCl3
16
15
14
13
12
11
10
9
8
7
6
5
4
3
2
1
0
-1
-2
ppm
1.00
0.98
2.00
1.02
1.01
3.14

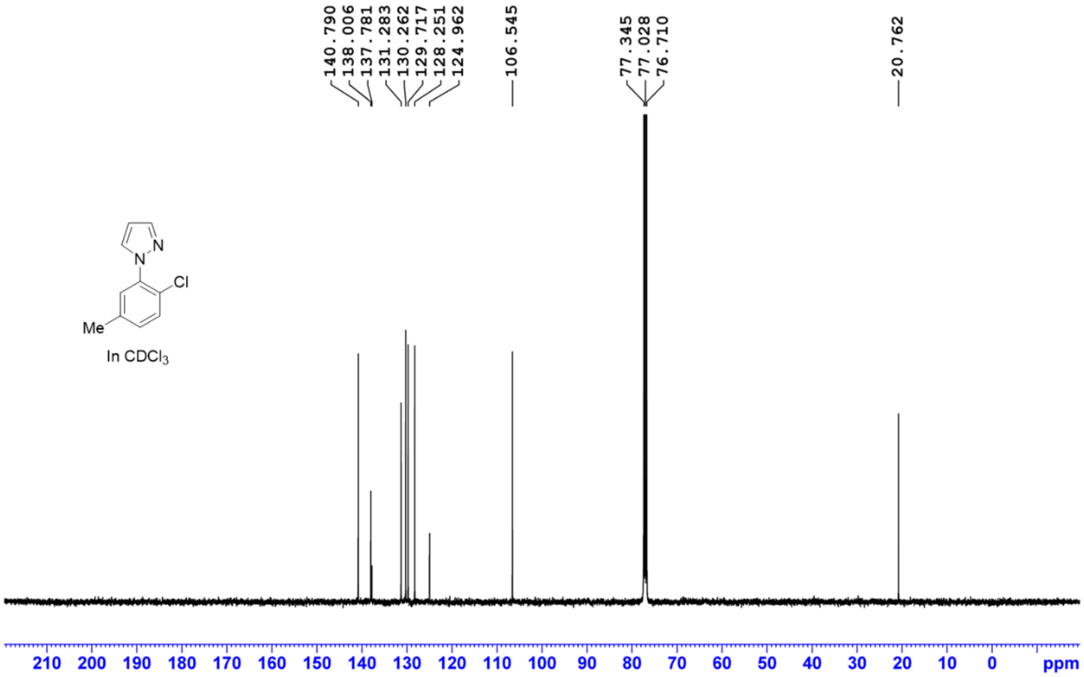
140.790
138.006
137.781
131.283
130.262
129.717
128.251
124.962
106.545
77.345
77.028
76.710
20.762
Cl
N
N
Me
In CDCl3
210
200
190
180
170
160
150
140
130
120
110
100
90
80
70
60
50
40
30
20
10
0
ppm

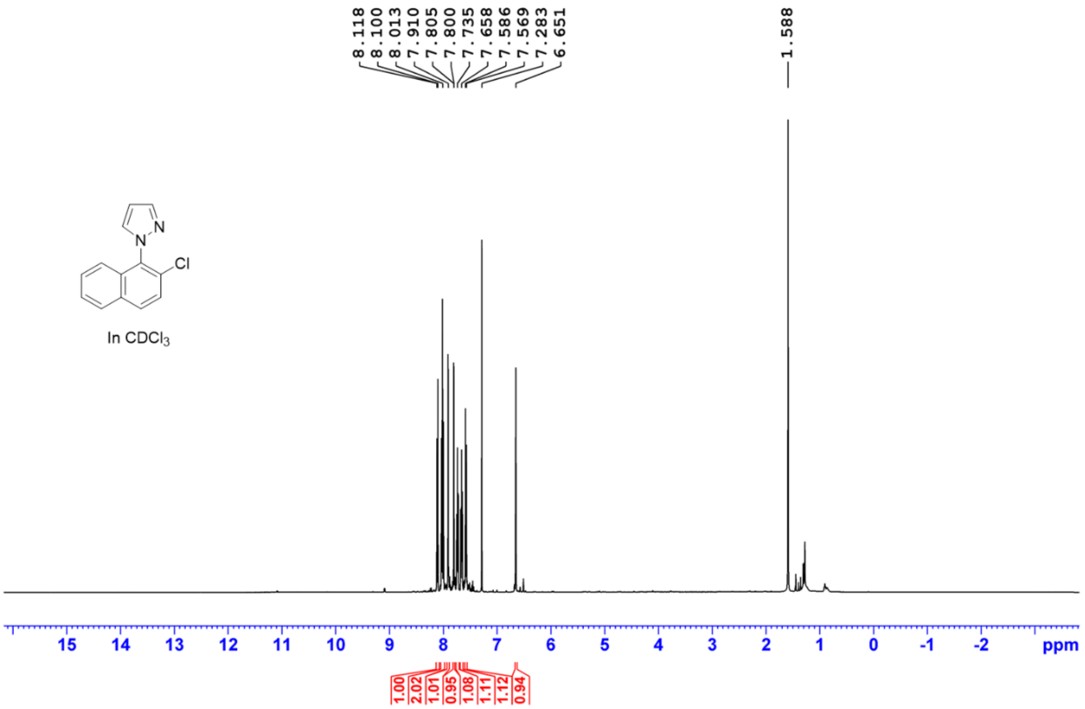

8.118
8.100
8.013
7.910
7.805
7.800
7.735
7.658
7.586
7.569
7.283
6.651
1.588
N
N
Cl
In $CDCl_3$
15 14 13 12 11 10 9 8 7 6 5 4 3 2 1 0 -1 -2 ppm
1.00
2.02
1.01
0.95
1.08
1.11
1.12
0.94

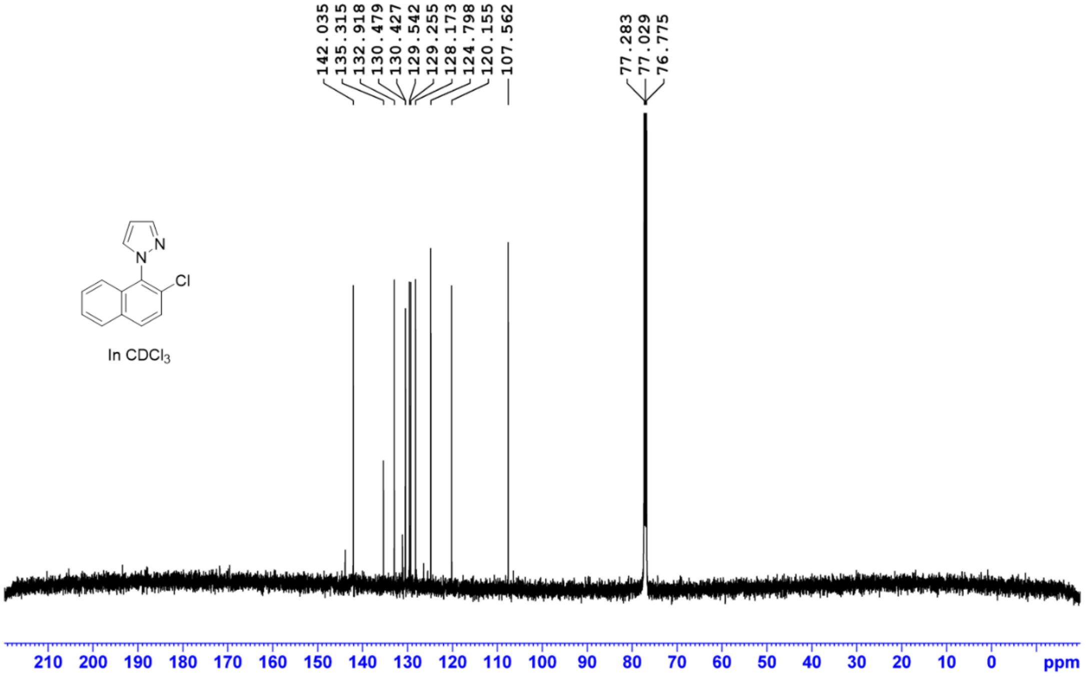

142.035
135.315
132.918
130.479
130.427
129.542
129.255
128.173
124.798
120.155
107.562
77.283
77.029
76.775
N
N
Cl
In $CDCl_3$
210 200 190 180 170 160 150 140 130 120 110 100 90 80 70 60 50 40 30 20 10 0 ppm

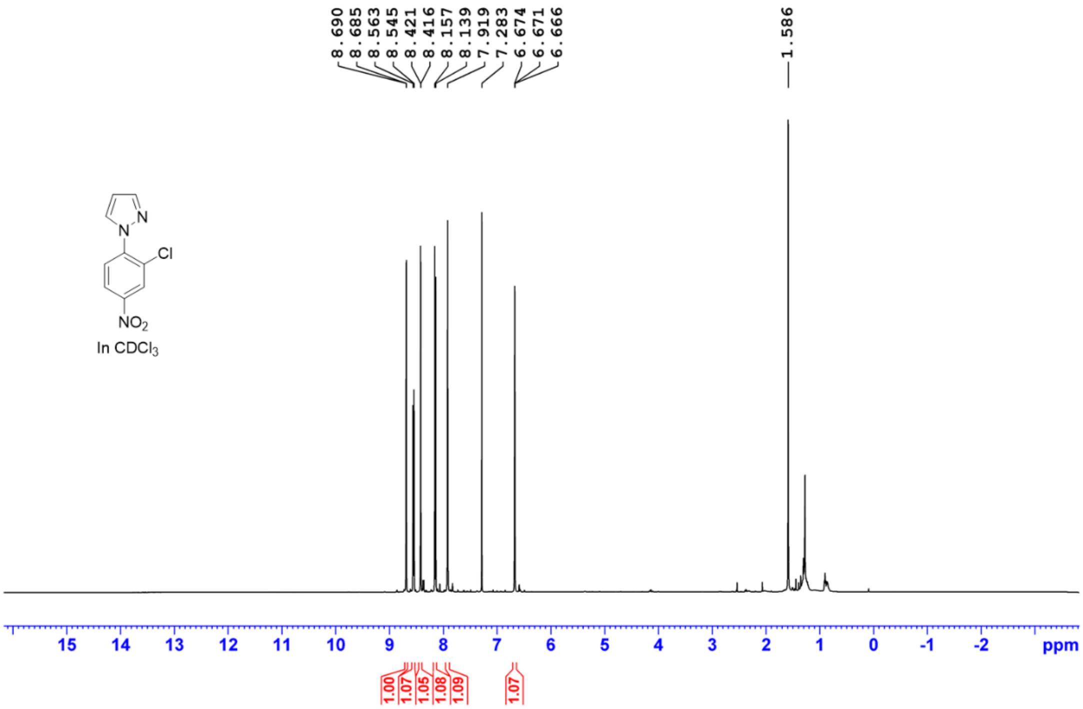
8.690
8.685
8.563
8.545
8.421
8.416
8.157
8.139
7.919
7.283
6.674
6.671
6.666
1.586
Cl
N
N
NO2
In CDCl3
1.00
1.07
1.05
1.08
1.09
1.07
ppm

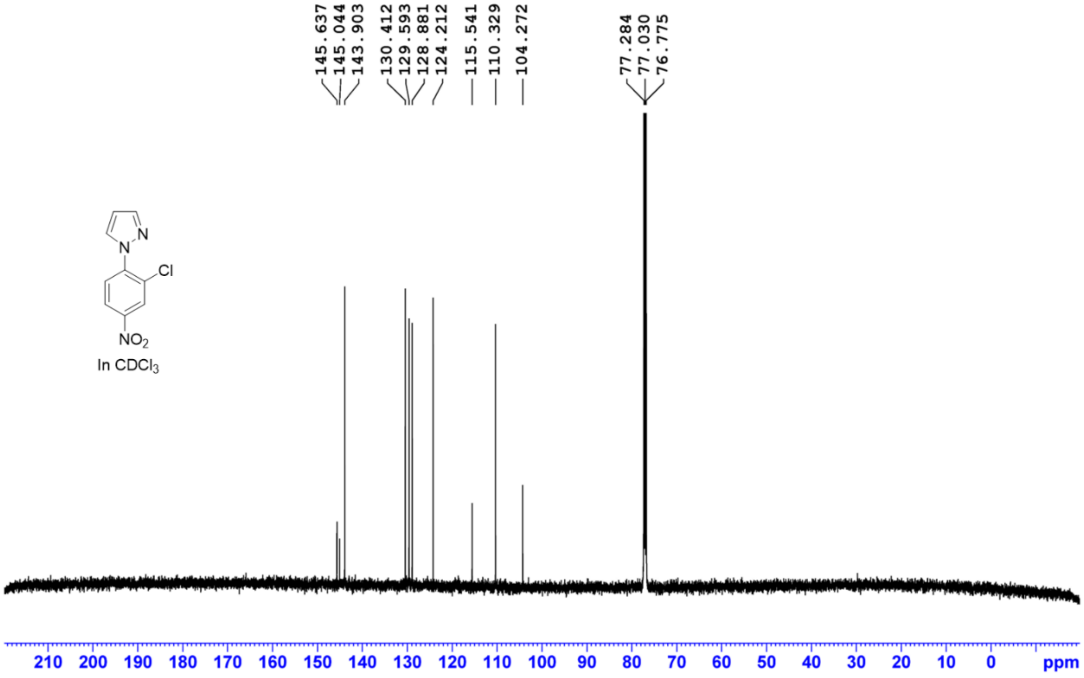
145.637
145.044
143.903
130.412
129.593
128.881
124.212
115.541
110.329
104.272
77.284
77.030
76.775
Cl
N
N
NO2
In CDCl3
ppm

Supplementary Information

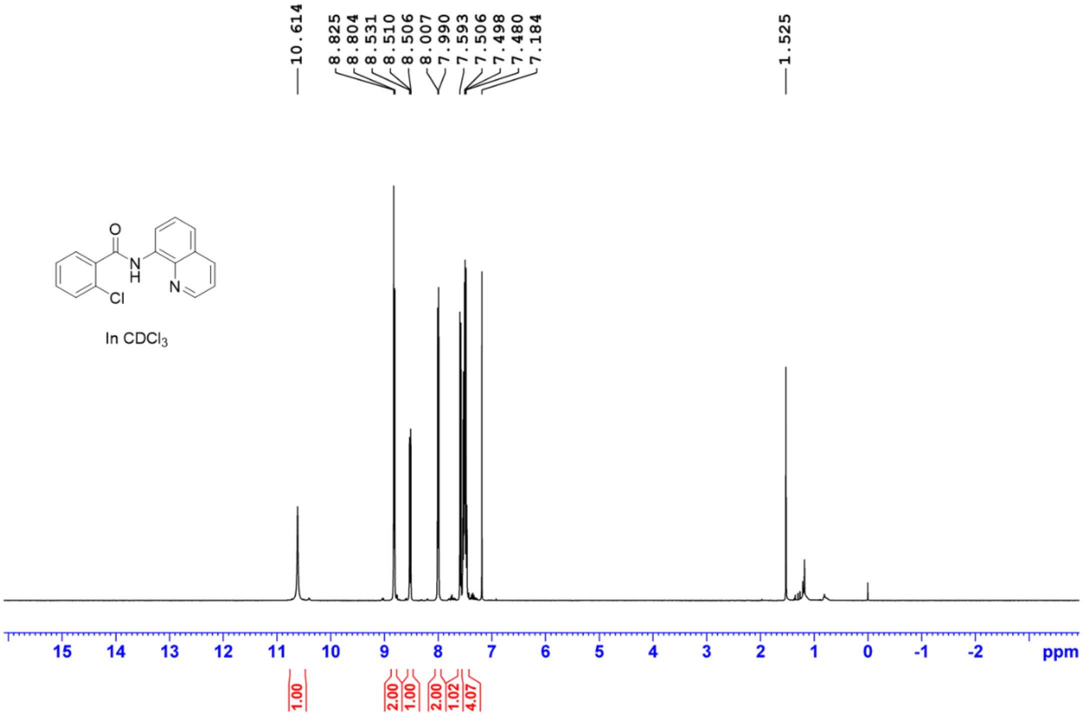

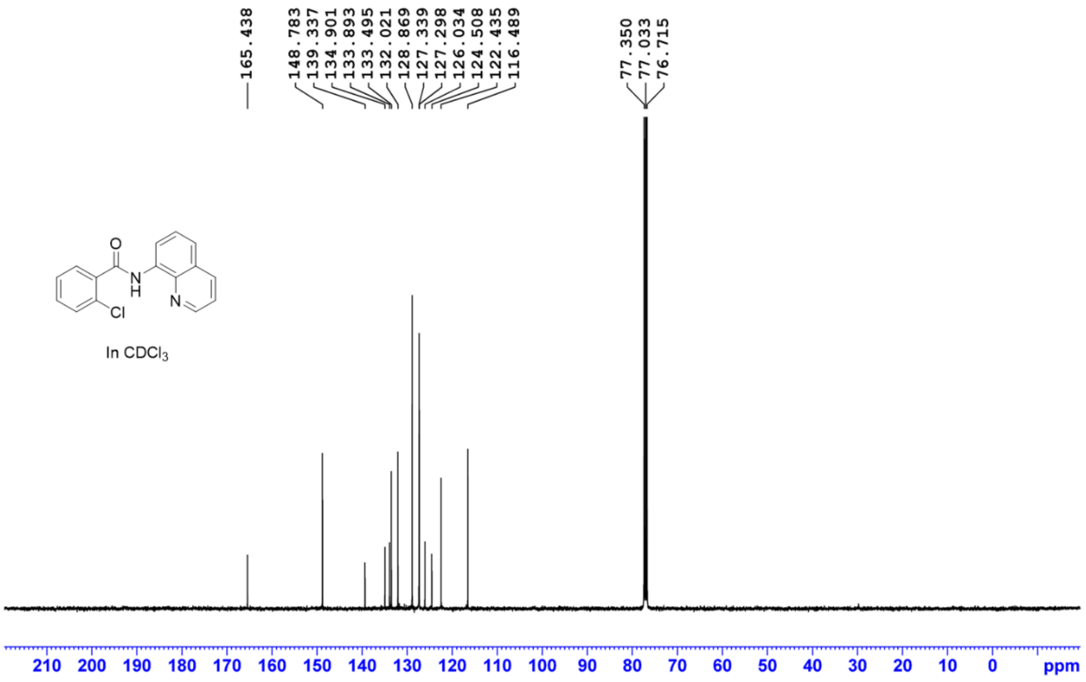

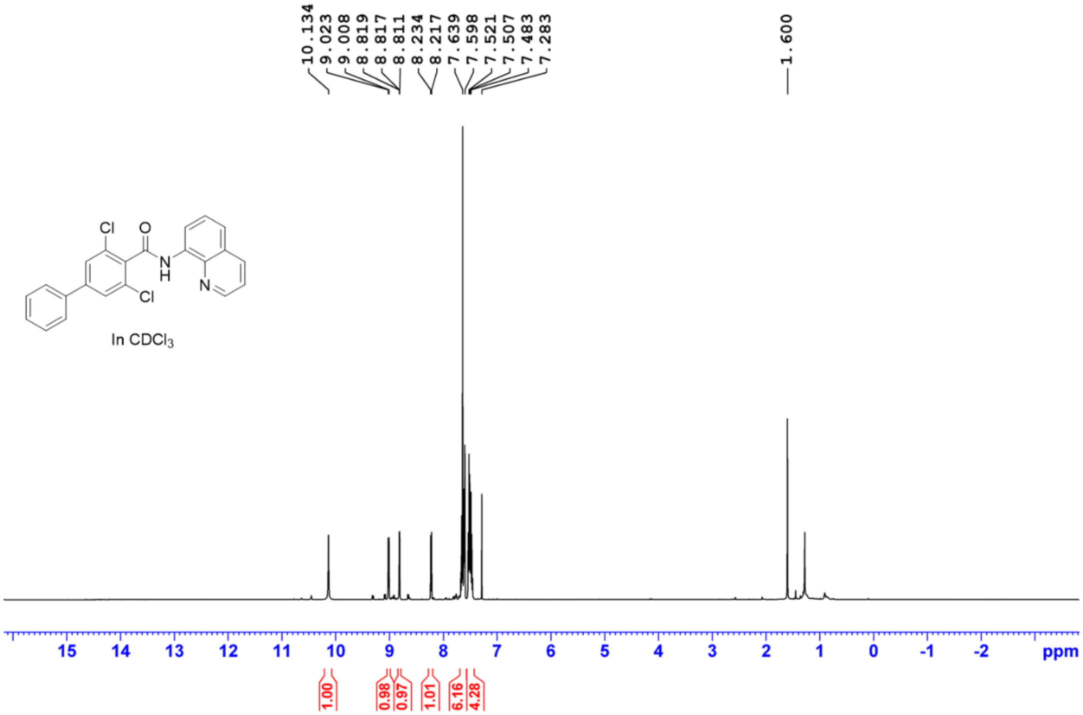
10.134
9.023
9.008
8.819
8.817
8.811
8.234
8.217
7.639
7.598
7.521
7.507
7.483
7.283
1.600
Cl
O
N
H
Cl
N
In CDCl3
15 14 13 12 11 10 9 8 7 6 5 4 3 2 1 0 -1 -2 ppm
1.00
0.98
0.97
1.01
6.16
4.28

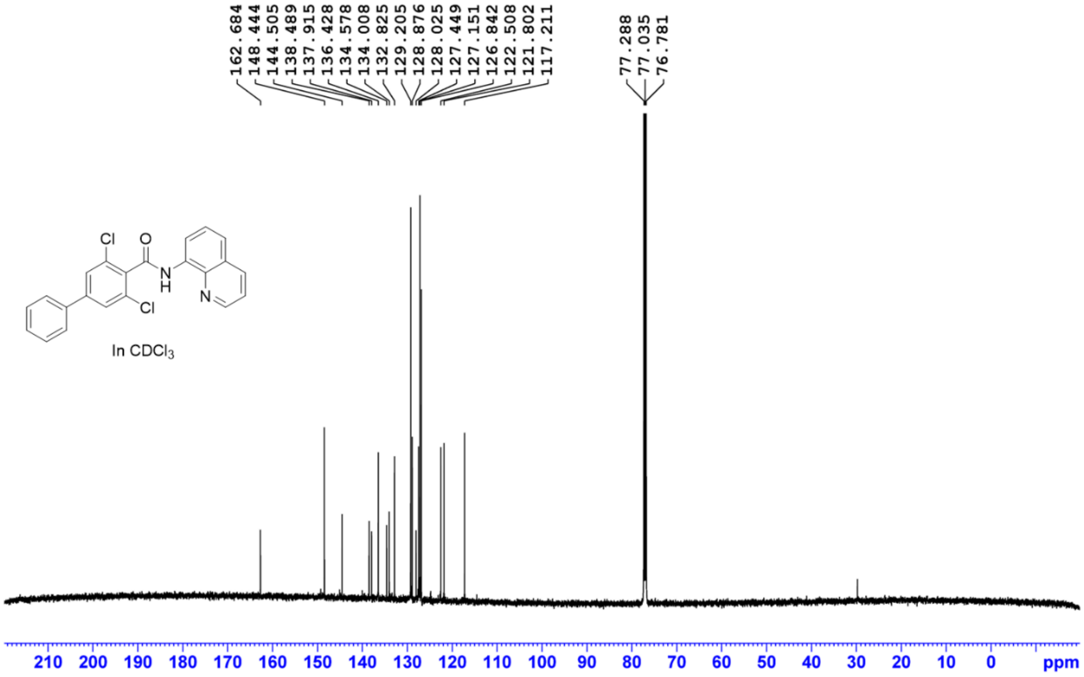
162.684
148.444
144.505
138.489
137.915
136.428
134.578
134.008
132.825
129.205
128.876
128.025
127.449
127.151
126.842
122.508
121.802
117.211
77.288
77.035
76.781
Cl
O
N
H
Cl
N
In CDCl3
210 200 190 180 170 160 150 140 130 120 110 100 90 80 70 60 50 40 30 20 10 0 ppm

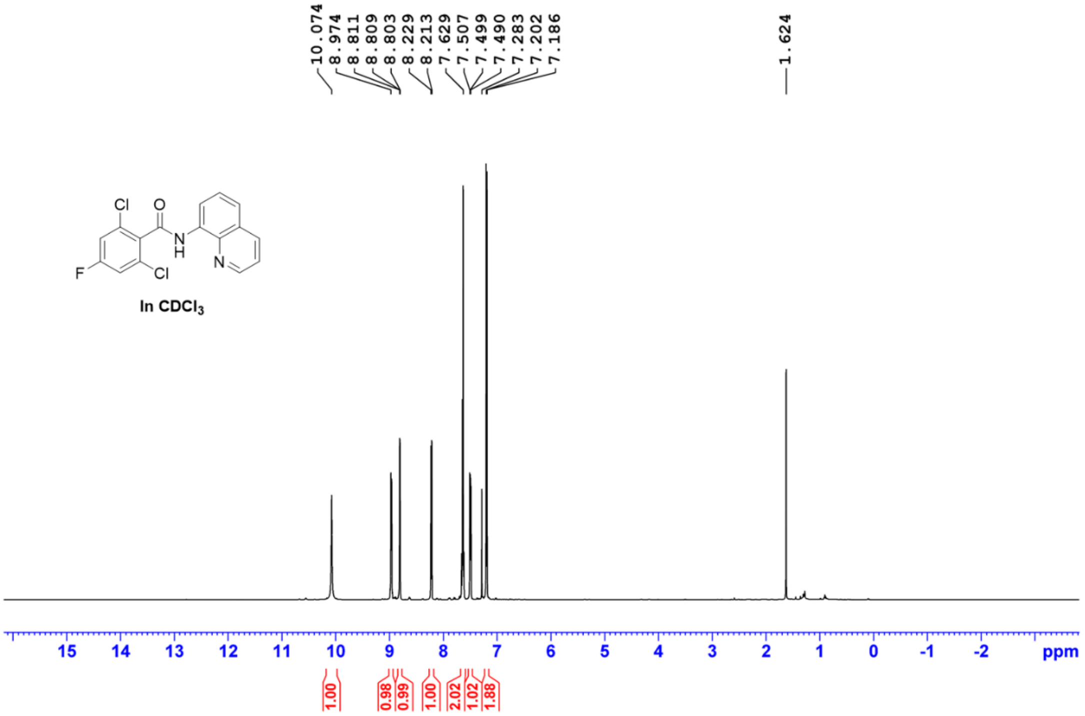
10.074
8.974
8.811
8.809
8.803
8.229
8.213
7.629
7.507
7.499
7.490
7.283
7.202
7.186
1.624
Cl
O
N
H
F
Cl
N
In CDCl3
15
14
13
12
11
10
9
8
7
6
5
4
3
2
1
0
-1
-2
ppm
1.00
0.98
0.99
1.00
2.02
1.02
1.88

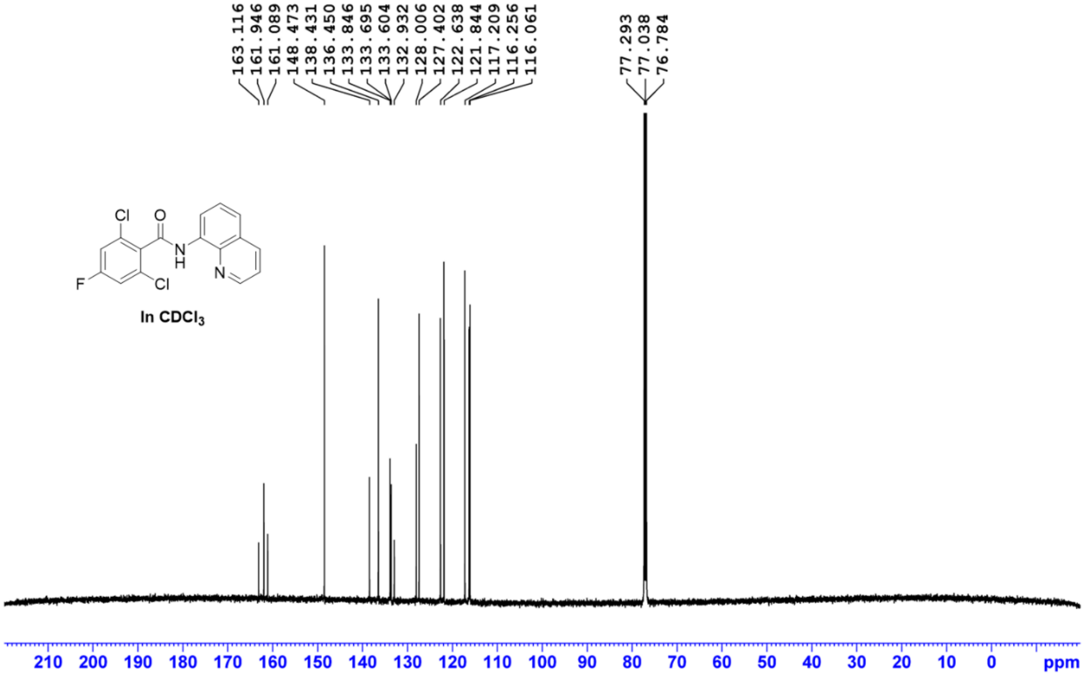
163.116
161.946
161.089
148.473
138.431
136.450
133.846
133.695
133.604
132.932
128.006
127.402
122.638
121.844
117.209
116.256
116.061
77.293
77.038
76.784
Cl
O
N
H
F
Cl
N
In CDCl3
210
200
190
180
170
160
150
140
130
120
110
100
90
80
70
60
50
40
30
20
10
0
ppm

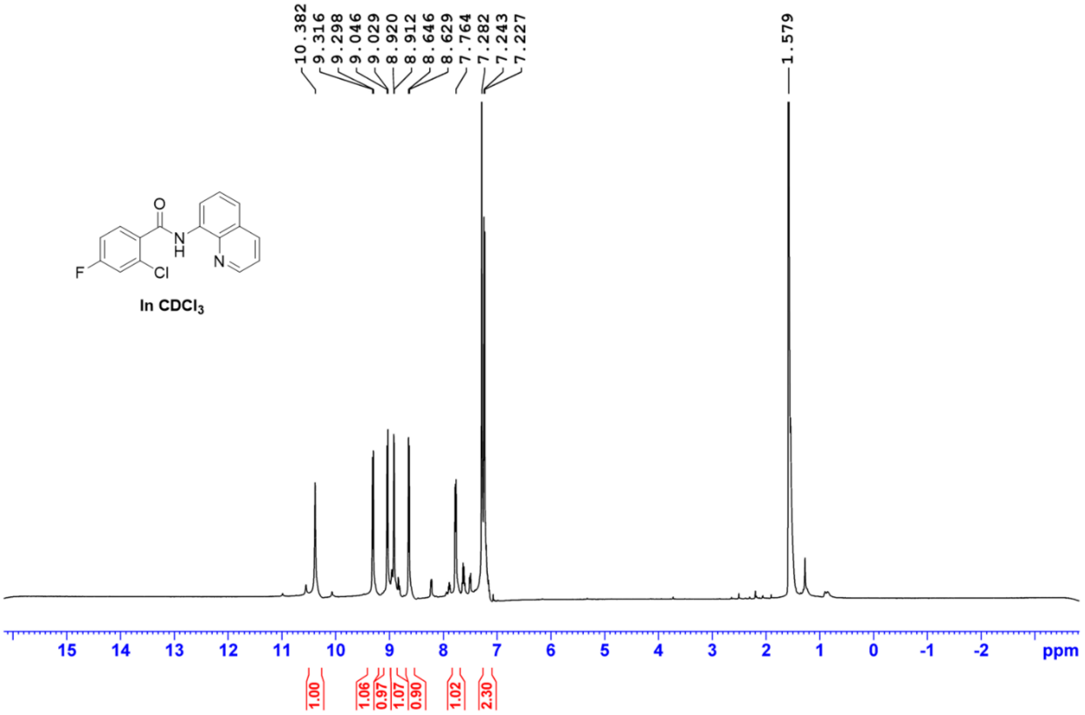
10.382
9.316
9.298
9.046
9.029
8.920
8.912
8.646
8.629
7.764
7.282
7.243
7.227
1.579
O
N
H
F
Cl
N
In $CDCl_3$
15 14 13 12 11 10 9 8 7 6 5 4 3 2 1 0 -1 -2 ppm
1.00
1.06
0.97
1.07
0.90
1.02
2.30

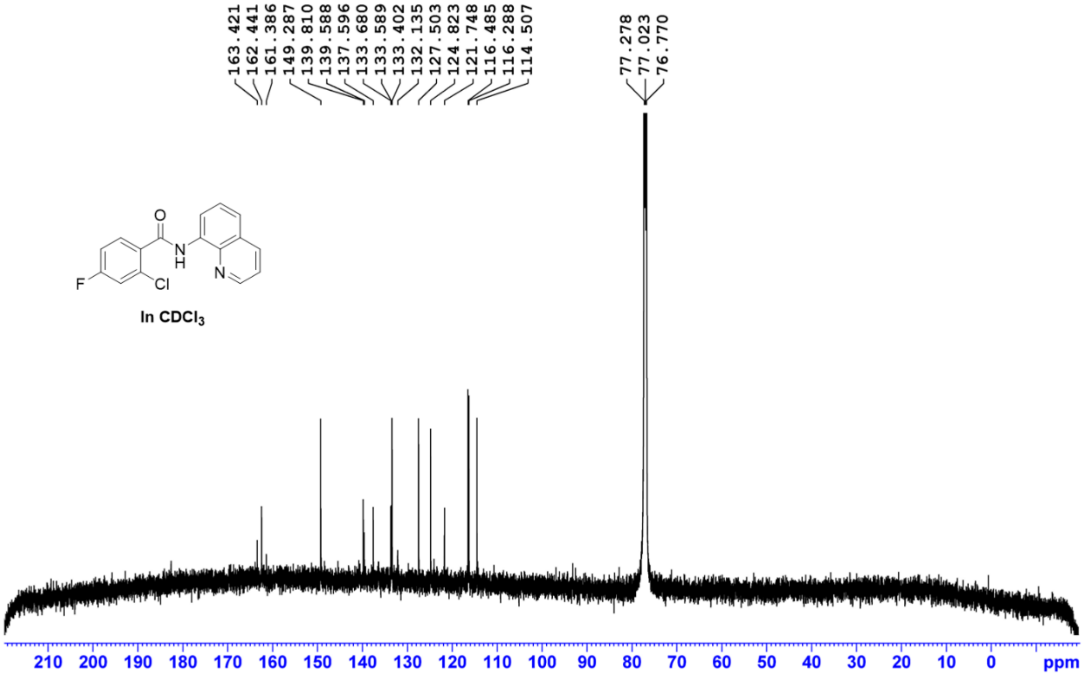
163.421
162.441
161.386
149.287
139.810
139.588
137.596
133.680
133.589
133.402
132.135
127.503
124.823
121.748
116.485
116.288
114.507
77.278
77.023
76.770
O
N
H
F
Cl
N
In $CDCl_3$
210 200 190 180 170 160 150 140 130 120 110 100 90 80 70 60 50 40 30 20 10 0 ppm

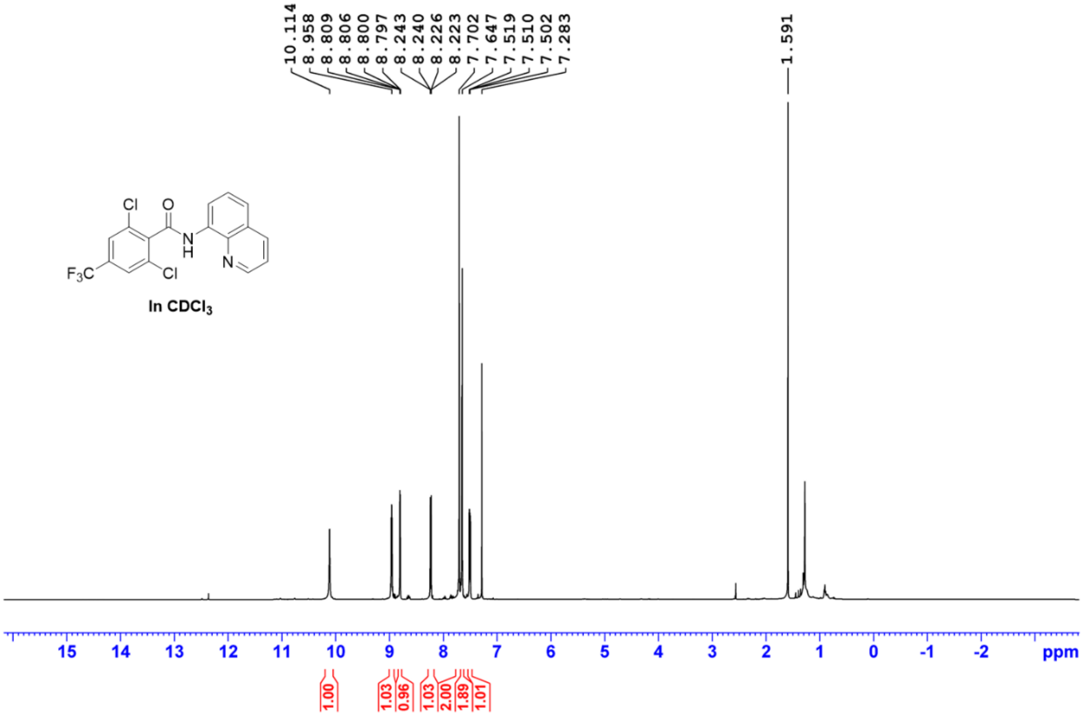
10.114
8.958
8.809
8.806
8.800
8.797
8.243
8.240
8.226
8.223
7.702
7.647
7.519
7.510
7.502
7.283
1.591
Cl
O
N
H
N
F3C
Cl
In CDCl3
15 14 13 12 11 10 9 8 7 6 5 4 3 2 1 0 -1 -2 ppm
1.00
1.03
0.96
1.03
2.00
1.89
1.01

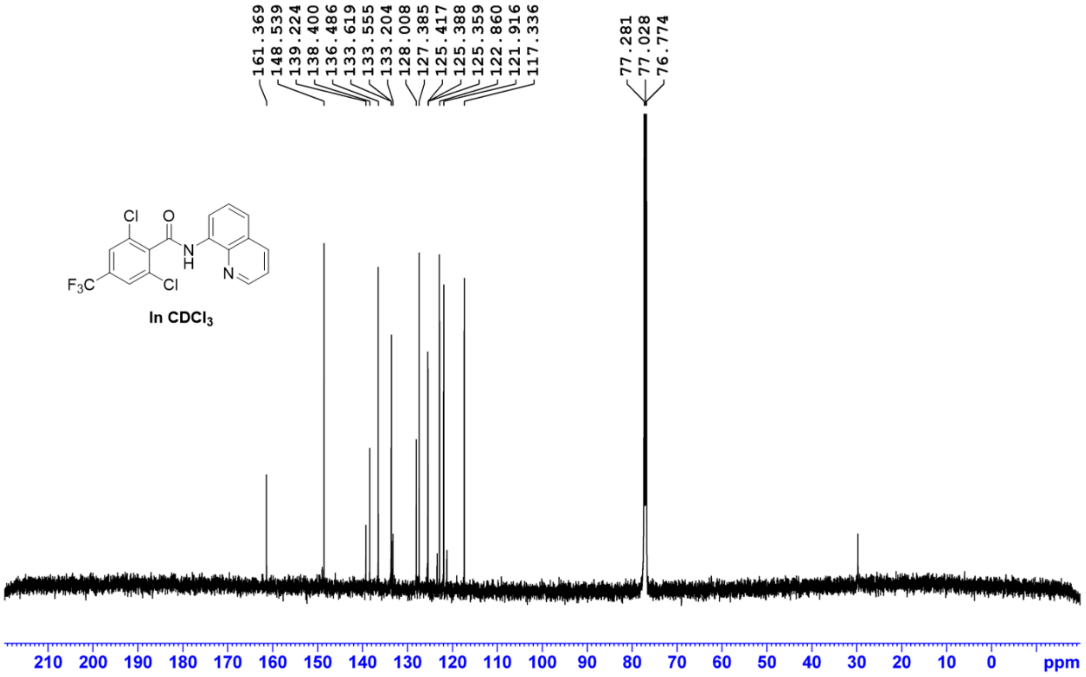
161.369
148.539
139.224
138.400
136.486
133.619
133.555
133.204
128.008
127.385
125.417
125.388
125.359
122.860
121.916
117.336
77.281
77.028
76.774
Cl
O
N
H
N
F3C
Cl
In CDCl3
210 200 190 180 170 160 150 140 130 120 110 100 90 80 70 60 50 40 30 20 10 0 ppm

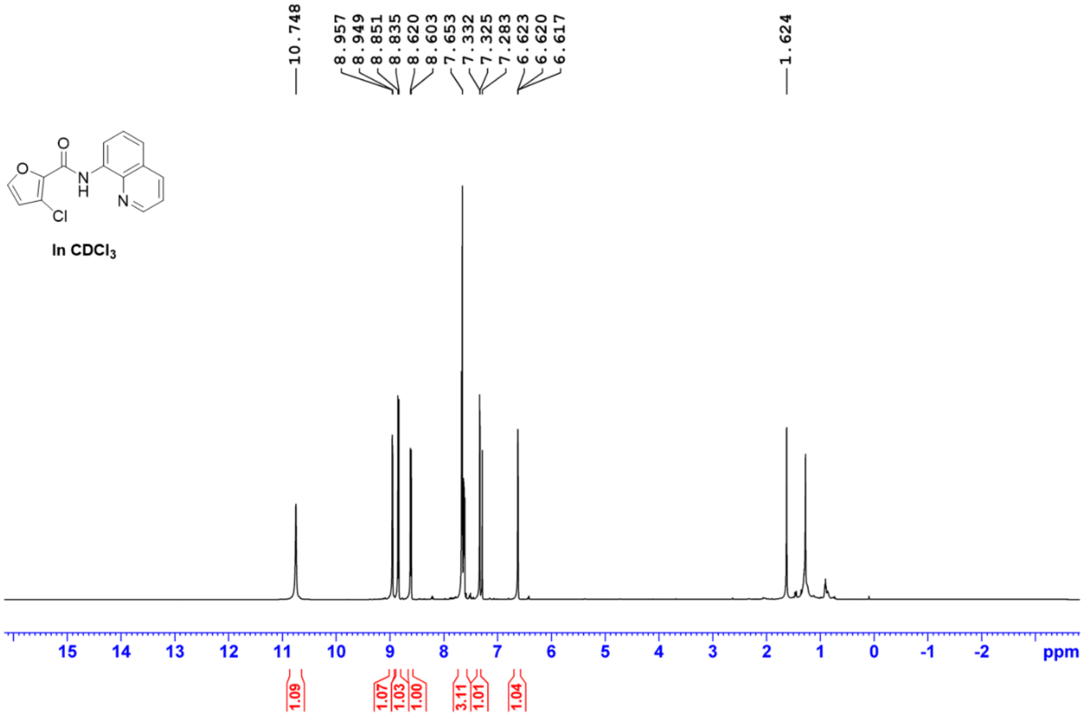
10.748
8.957
8.949
8.851
8.835
8.620
8.603
7.653
7.332
7.325
7.283
6.623
6.620
6.617
1.624
In CDCl3
15 14 13 12 11 10 9 8 7 6 5 4 3 2 1 0 -1 -2 ppm
1.09
1.07
1.03
1.00
3.11
1.01
1.04

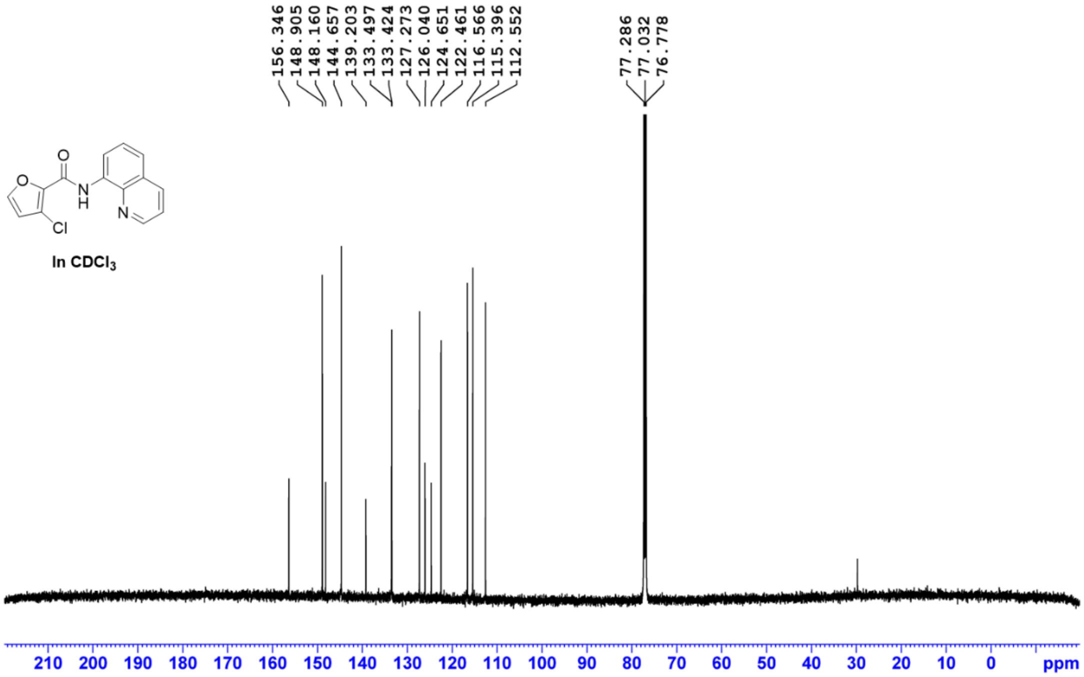
156.346
148.905
148.160
144.657
139.203
133.497
133.424
127.273
126.040
124.651
122.461
116.566
115.396
112.552
77.286
77.032
76.778
In CDCl3
210 200 190 180 170 160 150 140 130 120 110 100 90 80 70 60 50 40 30 20 10 0 ppm

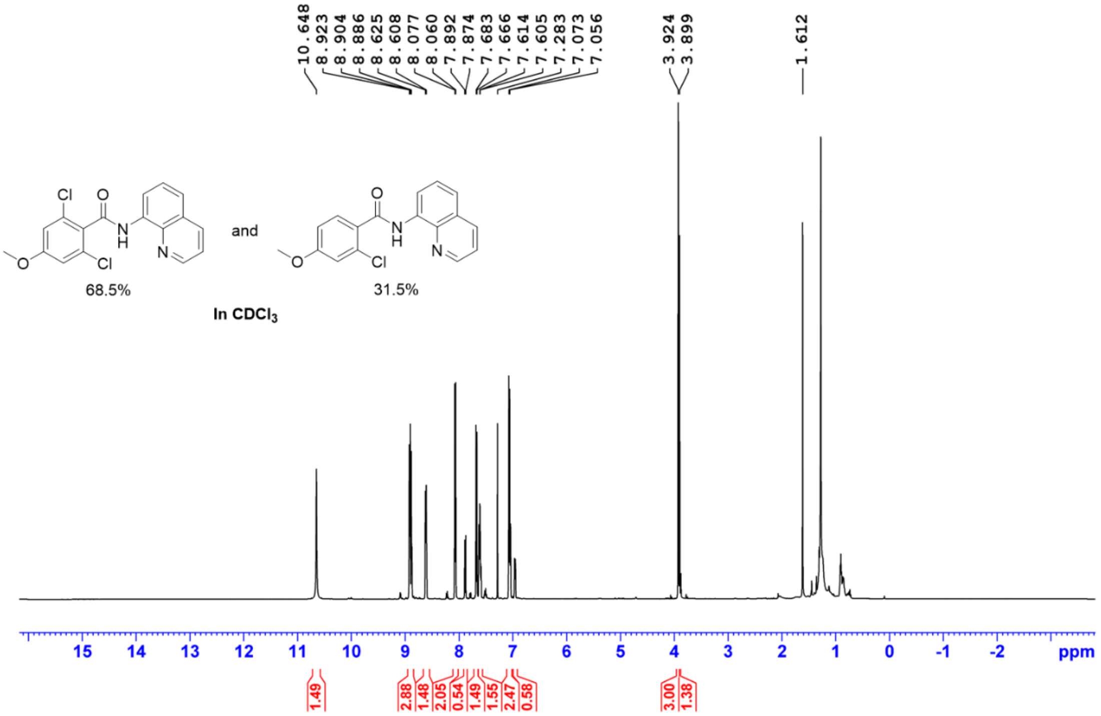
10.648
8.923
8.904
8.886
8.625
8.608
8.077
8.060
7.892
7.874
7.683
7.666
7.614
7.605
7.283
7.073
7.056
3.924
3.899
1.612
Cl
O
N
H
N
Cl
O
and
O
N
H
N
O
Cl
68.5%
31.5%
In CDCl3
15 14 13 12 11 10 9 8 7 6 5 4 3 2 1 0 -1 -2 ppm
1.49
2.88
1.48
2.05
0.54
1.49
1.55
2.47
0.58
3.00
1.38

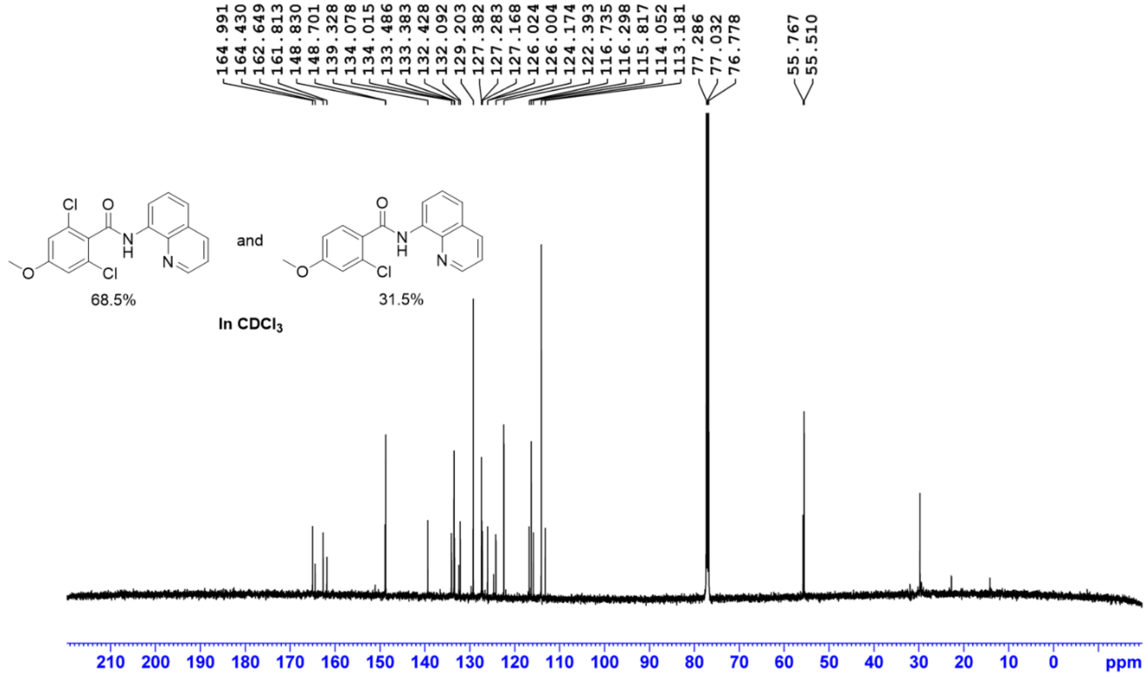
164.991
164.430
162.649
161.813
148.830
148.701
139.328
134.078
134.015
133.486
133.383
132.428
132.092
129.203
127.382
127.283
127.168
126.024
126.004
124.174
122.393
116.735
116.298
115.817
114.052
113.181
77.286
77.032
76.778
55.767
55.510
Cl
O
N
H
N
Cl
O
and
O
N
H
N
O
Cl
68.5%
31.5%
In CDCl3
210 200 190 180 170 160 150 140 130 120 110 100 90 80 70 60 50 40 30 20 10 0 ppm

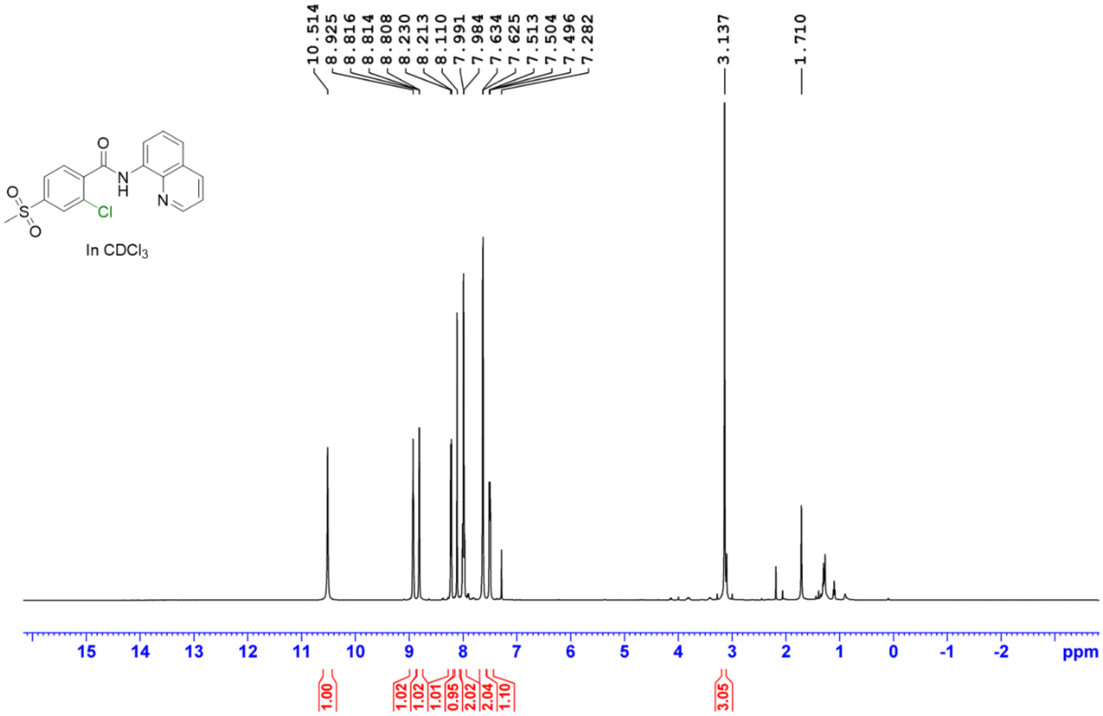
10.514
8.925
8.816
8.814
8.808
8.230
8.213
8.110
7.991
7.984
7.634
7.625
7.513
7.504
7.496
7.282
3.137
1.710
In CDCl$_3$
ppm
1.00
1.02
1.02
1.01
0.95
2.02
2.04
1.10
3.05

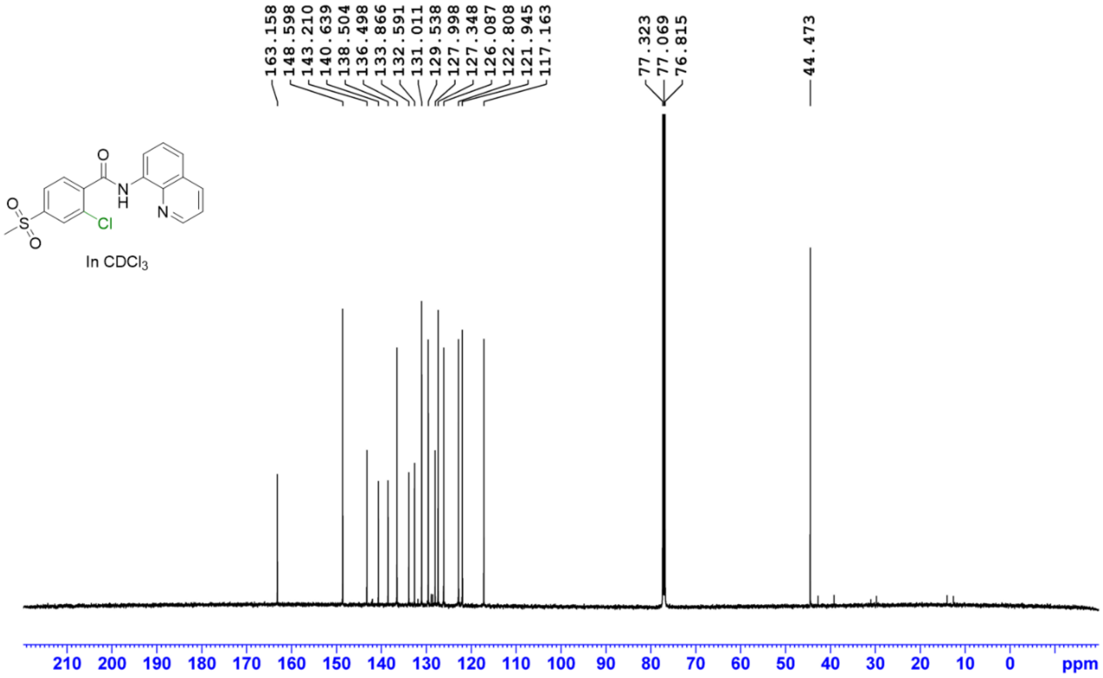
163.158
148.598
143.210
140.639
138.504
136.498
133.866
132.591
131.011
129.538
127.998
127.348
126.087
122.808
121.945
117.163
77.323
77.069
76.815
44.473
In CDCl$_3$
ppm

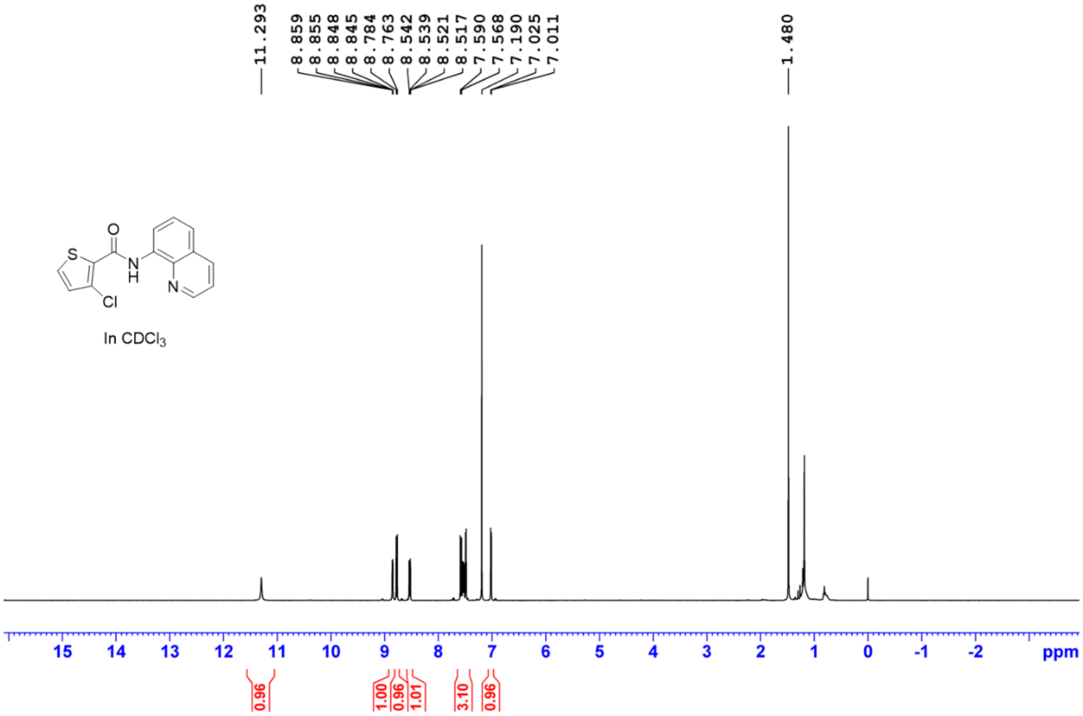
11.293
8.859
8.855
8.848
8.845
8.784
8.763
8.542
8.539
8.521
8.517
7.590
7.568
7.190
7.025
7.011
1.480
S
O
N
H
Cl
N
In CDCl3
15 14 13 12 11 10 9 8 7 6 5 4 3 2 1 0 -1 -2 ppm
0.96
1.00
0.96
1.01
3.10
0.96

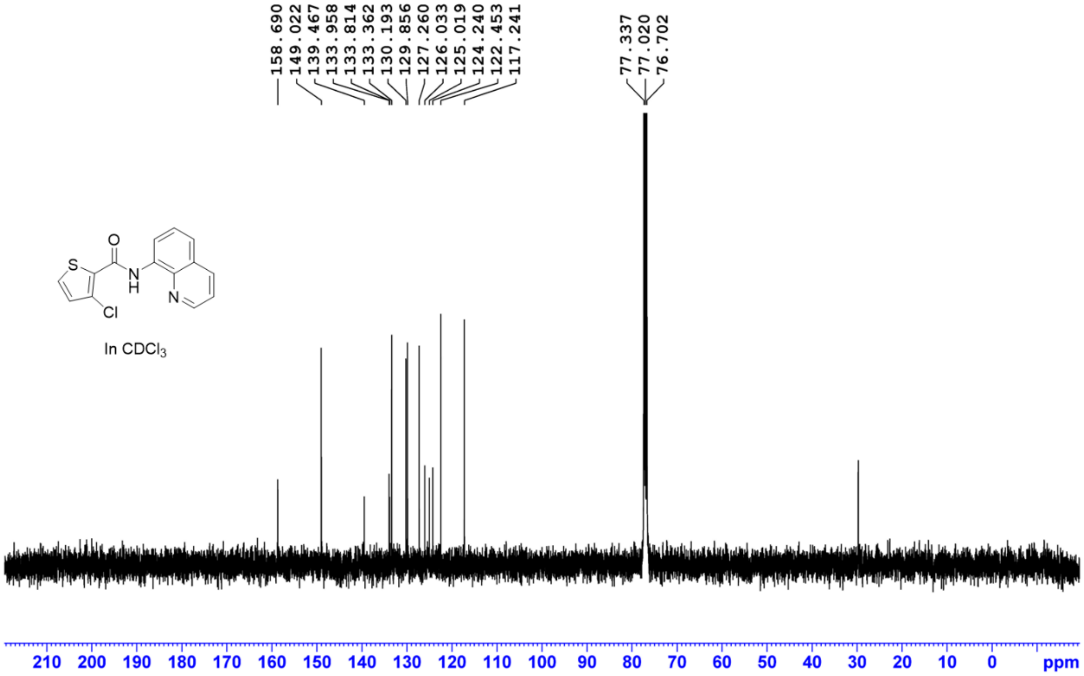
158.690
149.022
139.467
133.958
133.814
133.362
130.193
129.856
127.260
126.033
125.019
124.240
122.453
117.241
77.337
77.020
76.702
S
O
N
H
Cl
N
In CDCl3
210 200 190 180 170 160 150 140 130 120 110 100 90 80 70 60 50 40 30 20 10 0 ppm

References

1. Huy, P. H.; Motsch, S.; Kappler, S. M., Formamides as Lewis Base Catalysts in SN Reactions—Efficient Transformation of Alcohols into Chlorides, Amines, and Ethers. *Angew. Chem., Int. Ed.* **2016**, *55*, 10145-10149.
2. Pritchard, J. G.; Vollmer, R. L., The meso and Racemic Forms of 2,4-Pentanediol and Certain of Their Derivatives. *J. Org. Chem.* **1963**, *28*, 1545-1549.
3. Dong, X.-Y.; Zhang, Y.-F.; Ma, C.-L.; Gu, Q.-S.; Wang, F.-L.; Li, Z.-L.; Jiang, S.-P.; Liu, X.-Y., A general asymmetric copper-catalysed Sonogashira C(sp3)–C(sp) coupling. *Nat. Chem.* **2019**, *11*, 1158-1166.
4. Littke, A. F.; Fu, G. C., A Convenient and General Method for Pd-Catalyzed Suzuki Cross-Couplings of Aryl Chlorides and Arylboronic Acids. *Angew. Chem., Int. Ed.* **1998**, *37*, 3387-3388.
5. Xu, Z.-L.; Li, H.-X.; Ren, Z.-G.; Du, W.-Y.; Xu, W.-C.; Lang, J.-P., Cu(OAc)2·H2O-catalyzed N-arylation of nitrogen-containing heterocycles. *Tetrahedron* **2011**, *67*, 5282-5288.
6. Thrimurtulu, N.; Dey, A.; Maiti, D.; Volla, C. M. R., Cobalt-Catalyzed sp2-C−H Activation: Intermolecular Heterocyclization with Allenes at Room Temperature. *Angew. Chem., Int. Ed.* **2016**, *55*, 12361-12365.
7. Powers, D. C.; Benitez, D.; Tkatchouk, E.; Goddard, W. A., III; Ritter, T., Bimetallic Reductive Elimination from Dinuclear Pd(III) Complexes. *J. Am. Chem. Soc.* **2010**, *132*, 14092-14103.
8. Yoshioka, T.; Furukawa, K.; Okuwaki, A., Chemical recycling of rigid-PVC by oxygen oxidation in NaOH solutions at elevated temperatures. *Polym. Degrad. Stab.* **2000**, *67*, 285-290.
9. Hashimoto, K.; Suga, S.; Wakayama, Y.; Funazukuri, T., Hydrothermal dechlorination of PVC in the presence of ammonia. *J. Mater. Sci.* **2008**, *43*, 2457-2462.
10. Zhao, T.; Zhou, Q.; He, X.-L.; Wei, S.-D.; Wang, L.; van Kasteren, J. M. N.; Wang, Y.-Z., A highly efficient approach for dehydrochlorinating polyvinyl chloride: catalysis by 1-butyl-3-methylimidazolium chloride. *Green Chem.* **2010**, *12*, 1062-1065.
11. Yoshihara, M.; Grause, G.; Kameda, T.; Yoshioka, T., Upgrading of poly(vinyl chloride) by chemical modifications using sodium sulfide. *J. Mater. Cycles Waste Manag.* **2010**, *12*, 264-270.
12. Kameda, T.; Wachi, S.; Grause, G.; Mizoguchi, T.; Yoshioka, T., Dehydrochlorination of poly(vinyl chloride) with Ca(OH)2 in ethylene glycol and the effect of ball milling. *J. Polym. Res.* **2011**, *18*, 1687-1691.
13. Glas, D.; Hulsbosch, J.; Dubois, P.; Binnemans, K.; De Vos, D. E., End-of-Life Treatment of Poly(Vinyl Chloride) and Chlorinated Polyethylene by Dehydrochlorination in Ionic Liquids. *ChemSusChem* **2014**, *7*, 610-617.
14. Oster, K.; Tedstone, A.; Greer, A. J.; Budgen, N.; Garforth, A.; Hardacre, C., Dehydrochlorination of PVC in multi-layered blisterpacks using ionic liquids. *Green Chem.* **2020**, *22*, 5132-5142.
15. Assefa, M. K.; Fieser, M. E., Divergent silylium catalysis enables facile poly(vinyl chloride) upcycling to poly(ethylene-co-styrene) derivatives. *J. Mater. Chem. A* **2023**, *11*, 2128-2132.
16. O'Rourke, G.; Hennebel, T.; Stalpaert, M.; Skorynina, A.; Bugaev, A.; Janssens, K.; Van Emelen, L.; Lemmens, V.; De Oliveira Silva, R.; Colemonts, C.; Gabriels, P.; Sakellariou, D.; De Vos, D., Catalytic tandem dehydrochlorination–hydrogenation of PVC towards valorisation of chlorinated plastic waste. *Chem. Sci.* **2023**, *14*, 4401-4412.
17. Zhou, Q.; Lan, W.; Du, A.; Wang, Y.; Yang, J.; Wu, Y.; Yang, K.; Wang, X., Lanthania promoted MgO: Simultaneous highly efficient catalytic degradation and dehydrochlorination of polypropylene/polyvinyl chloride. *Appl. Catal. B: Environ.* **2008**, *80*, 141-146.
18. Bae, S. Y.; Jeon, S.; Lee, Y. H.; Lee, D. H.; Kye, H.; Bae, J. W., Dehydrochlorination of polyvinylchloride using Al-modified graphitic-C3N4. *RSC Advances* **2016**, *6*, 20728-20733.
19. Chen, Y.; Zhang, S.; Han, X.; Zhang, X.; Yi, M.; Yang, S.; Yu, D.; Liu, W., Catalytic Dechlorination and Charring Reaction of Polyvinyl Chloride by CuAl Layered Double Hydroxide. *Energy Fuels* **2018**, *32*, 2407-2413.

20. Gao, Z.; Lu, L.; Shi, C.; Qian, X.; Wang, X.; Zhang, G.; Zhou, M.; Pan, Y., The study of ZnAl and ZnFe layered double hydroxide on the catalytic dechlorination and fire safety of polyvinyl chloride. *J. Therm. Anal. Calorim.* **2020**, *140*, 115-123.
21. Jin, D.; Khanal, S.; Xu, S., Effects of ZnAl layered double hydroxide and benzimidazole derivative on the dechlorination of poly(vinyl chloride). *Appl. Clay Sci.* **2021**, *208*, 106114.
22. Kots, P. A.; Vance, B. C.; Quinn, C. M.; Wang, C.; Vlachos, D. G., A two-stage strategy for upcycling chlorine-contaminated plastic waste. *Nature Sustainability* **2023**, *6*, 1258-1267.
23. Pearson, D. M.; Conley, N. R.; Waymouth, R. M., Palladium-Catalyzed Carbonylation of Diols to Cyclic Carbonates. *Adv. Synth. Catal.* **2011**, *353*, 3007-3013.
24. Eytel, L. M.; Fargher, H. A.; Haley, M. M.; Johnson, D. W., The road to aryl CH⋯anion binding was paved with good intentions: fundamental studies, host design, and historical perspectives in CH hydrogen bonding. *Chem. Commun.* **2019**, *55*, 5195-5206.
25. Wang, L.; Zhang, R.-z.; Deng, R.; Luo, Y.-h., Oxygen-Induced Enhancement in Low-Temperature Dechlorination of PVC: An Experimental and DFT Study on the Oxidative Pyrolysis Process. *ACS Sustain. Chem. Eng.* **2021**, *9*, 2835-2843.
26. Zhang, R.-z.; Wang, L.-z.; Deng, R.-q.; Luo, Y.-h., Inhibition on Chloroaromatics during thermal conversion processes of Municipal Solid Waste by oxygen-induced low-temperature pre-dechlorination and hydrogen-assisted in-situ Cl capture. *Fuel Process. Technol.* **2022**, *237*, 107445.
27. Song, S.; Li, X.; Wei, J.; Wang, W.; Zhang, Y.; Ai, L.; Zhu, Y.; Shi, X.; Zhang, X.; Jiao, N., DMSO-catalysed late-stage chlorination of (hetero)arenes. *Nat. Catal.* **2020**, *3*, 107-115.
28. Nizovtsev, A. S.; Bogdanchikov, G. A.; Baklanov, A. V., The computational study of the "inversion substitution" reactions CX3Br+O2→CX3O2+Br (X=H, F). *Combust. Flame* **2010**, *157*, 1382-1389.
29. Richard, J. P.; Jencks, W. P., Concerted bimolecular substitution reactions of 1-phenylethyl derivatives. *J. Am. Chem. Soc.* **1984**, *106*, 1383-1396.
30. Hasan, T.; Sims, L. B.; Fry, A., Heavy atom isotope effect studies of elimination reaction mechanisms. 1. A kinetic and carbon-14 kinetic isotope effect study of the base-promoted dehydrochlorination of substituted 1-phenylethyl-2-14C chlorides. *J. Am. Chem. Soc.* **1983**, *105*, 3967-3975.
31. Wang, Z.; Bachman, S.; Dudnik, A. S.; Fu, G. C., Nickel-Catalyzed Enantioconvergent Borylation of Racemic Secondary Benzylic Electrophiles. *Angew. Chem., Int. Ed.* **2018**, *57*, 14529-14532.
32. Mueller, J. A.; Goller, C. P.; Sigman, M. S., Elucidating the Significance of β-Hydride Elimination and the Dynamic Role of Acid/Base Chemistry in a Palladium-Catalyzed Aerobic Oxidation of Alcohols. *J. Am. Chem. Soc.* **2004**, *126*, 9724-9734.
33. Schultz, M. J.; Park, C. C.; Sigman, M. S., A convenient palladium-catalyzed aerobic oxidation of alcohols at room temperature. *Chem. Commun.* **2002**, 3034-3035.
34. Schultz, M. J.; Adler, R. S.; Zierkiewicz, W.; Privalov, T.; Sigman, M. S., Using Mechanistic and Computational Studies To Explain Ligand Effects in the Palladium-Catalyzed Aerobic Oxidation of Alcohols. *J. Am. Chem. Soc.* **2005**, *127*, 8499-8507.
35. Wang, D.; Weinstein, A. B.; White, P. B.; Stahl, S. S., Ligand-Promoted Palladium-Catalyzed Aerobic Oxidation Reactions. *Chem. Rev.* **2018**, *118*, 2636-2679.
36. Liu, M.; Zhang, Z.; Song, J.; Liu, S.; Liu, H.; Han, B., Nitrogen Dioxide Catalyzed Aerobic Oxidative Cleavage of C(OH)–C Bonds of Secondary Alcohols to Produce Acids. *Angew. Chem., Int. Ed.* **2019**, *58*, 17393-17398.
37. Piera, J.; Bäckvall, J.-E., Catalytic Oxidation of Organic Substrates by Molecular Oxygen and Hydrogen Peroxide by Multistep Electron Transfer—A Biomimetic Approach. *Angew. Chem., Int. Ed.* **2008**, *47*, 3506-3523.
38. Whitfield, S. R.; Sanford, M. S., Reactivity of Pd(II) Complexes with Electrophilic Chlorinating Reagents: Isolation of Pd(IV) Products and Observation of C−Cl Bond-Forming Reductive Elimination. *J. Am. Chem. Soc.* **2007**, *129*, 15142-15143.
39. Song, B.; Zheng, X.; Mo, J.; Xu, B., Palladium-Catalyzed Monoselective Halogenation of C□H Bonds: Efficient Access to Halogenated Arylpyrimidines using Calcium Halides. *Adv. Synth. Catal.* **2010**, *352*, 329-335.

40. Phan, N. T. S.; Van Der Sluys, M.; Jones, C. W., On the Nature of the Active Species in Palladium Catalyzed Mizoroki–Heck and Suzuki–Miyaura Couplings – Homogeneous or Heterogeneous Catalysis, A Critical Review. *Adv. Synth. Catal.* **2006**, *348*, 609-679.
41. Chernyshev, V. M.; Astakhov, A. V.; Chikunov, I. E.; Tyurin, R. V.; Eremin, D. B.; Ranny, G. S.; Khrustalev, V. N.; Ananikov, V. P., Pd and Pt Catalyst Poisoning in the Study of Reaction Mechanisms: What Does the Mercury Test Mean for Catalysis? *ACS Catal.* **2019**, *9*, 2984-2995.
42. Ji, Y.; Jain, S.; Davis, R. J., Investigation of Pd Leaching from Supported Pd Catalysts during the Heck Reaction. *J. Phys. Chem. B* **2005**, *109*, 17232-17238.
43. Chen, X.; Hao, X.-S.; Goodhue, C. E.; Yu, J.-Q., Cu(II)-Catalyzed Functionalizations of Aryl C−H Bonds Using O2 as an Oxidant. *J. Am. Chem. Soc.* **2006**, *128*, 6790-6791.
44. Yang, L.; Lu, Z.; Stahl, S. S., Regioselective copper-catalyzed chlorination and bromination of arenes with O2 as the oxidant. *Chem. Commun.* **2009**, 6460-6462.
45. Wang, W.; Pan, C.; Chen, F.; Cheng, J., Copper(ii)-catalyzed ortho-functionalization of 2-arylpyridines with acyl chlorides. *Chem. Commun.* **2011**, *47*, 3978-3980.
46. Pye, D. R.; Mankad, N. P., Bimetallic catalysis for C–C and C–X coupling reactions. *Chem. Sci.* **2017**, *8*, 1705-1718.
47. Kim, U. B.; Jung, D. J.; Jeon, H. J.; Rathwell, K.; Lee, S.-g., Synergistic Dual Transition Metal Catalysis. *Chem. Rev.* **2020**, *120*, 13382-13433.
48. Kakiuchi, F.; Kochi, T.; Mutsutani, H.; Kobayashi, N.; Urano, S.; Sato, M.; Nishiyama, S.; Tanabe, T., Palladium-Catalyzed Aromatic C−H Halogenation with Hydrogen Halides by Means of Electrochemical Oxidation. *J. Am. Chem. Soc.* **2009**, *131*, 11310-11311.
49. Dick, A. R.; Hull, K. L.; Sanford, M. S., A Highly Selective Catalytic Method for the Oxidative Functionalization of C−H Bonds. *J. Am. Chem. Soc.* **2004**, *126*, 2300-2301.
50. Fagnani, D. E.; Kim, D.; Camarero, S. I.; Alfaro, J. F.; McNeil, A. J., Using waste poly(vinyl chloride) to synthesize chloroarenes by plasticizer-mediated electro(de)chlorination. *Nat. Chem.* **2022**.
51. López Granados, M.; Moreno, J.; Alba-Rubio, A. C.; Iglesias, J.; Martín Alonso, D.; Mariscal, R., Catalytic transfer hydrogenation of maleic acid with stoichiometric amounts of formic acid in aqueous phase: paving the way for more sustainable succinic acid production. *Green Chem.* **2020**, *22*, 1859-1872.
52. Escobar-Hernandez, H. U.; Quan, Y.; Papadaki, M. I.; Wang, Q., Life Cycle Assessment of Metal–Organic Frameworks: Sustainability Study of Zeolitic Imidazolate Framework-67. *ACS Sustain. Chem. Eng.* **2023**, *11*, 4219-4225.
